# Mapping HIV prevalence in sub-Saharan Africa between 2000 and 2017

**DOI:** 10.1038/s41586-019-1200-9

**Published:** 2019-05-15

**Authors:** Laura Dwyer-Lindgren, Michael A. Cork, Amber Sligar, Krista M. Steuben, Kate F. Wilson, Naomi R. Provost, Benjamin K. Mayala, John D. VanderHeide, Michael L. Collison, Jason B. Hall, Molly H. Biehl, Austin Carter, Tahvi Frank, Dirk Douwes-Schultz, Roy Burstein, Daniel C. Casey, Aniruddha Deshpande, Lucas Earl, Charbel El Bcheraoui, Tamer H. Farag, Nathaniel J. Henry, Damaris Kinyoki, Laurie B. Marczak, Molly R. Nixon, Aaron Osgood-Zimmerman, David Pigott, Robert C. Reiner, Jennifer M. Ross, Lauren E. Schaeffer, David L. Smith, Nicole Davis Weaver, Kirsten E. Wiens, Jeffrey W. Eaton, Jessica E. Justman, Alex Opio, Benn Sartorius, Frank Tanser, Njeri Wabiri, Peter Piot, Christopher J. L. Murray, Simon I. Hay

**Affiliations:** 1 Institute for Health Metrics and Evaluation, University of Washington, Seattle, WA USA; 2 DHS program, ICF International, Rockville, MD USA; 3 Department of Global Health, University of Washington, Seattle, WA USA; 4 Department of Medicine, University of Washington, Seattle, WA USA; 5 Department of Infectious Disease Epidemiology, Imperial College London, London, UK; 6 ICAP, Mailman School of Public Health, Columbia University, New York, NY USA; 7 Vagelos College of Physicians and Surgeons, Columbia University, New York, NY USA; 8 Medireal Investment Uganda, Entebbe, Uganda; 9 Public Health Medicine, School of Nursing and Public Health, College of Health Sciences, University of KwaZulu-Natal, Durban, South Africa; 10 School of Nursing and Public Health, University of KwaZulu-Natal, Durban, South Africa; 11 Africa Health Research Institute, KwaZulu-Natal, South Africa; 12 Centre for the AIDS Programme of Research in South Africa (CAPRISA), University of KwaZulu-Natal, Durban, South Africa; 13 Research Department of Infection & Population Health, University College London, London, UK; 14 HIV/AIDS, STIs & TB Research Programme, Human Sciences Research Council, Pretoria, South Africa; 15 London School of Hygiene & Tropical Medicine, London, UK

**Keywords:** Epidemiology, HIV infections, Geography

## Abstract

HIV/AIDS is a leading cause of disease burden in sub-Saharan Africa. Existing evidence has demonstrated that there is substantial local variation in the prevalence of HIV; however, subnational variation has not been investigated at a high spatial resolution across the continent. Here we explore within-country variation at a 5 × 5-km resolution in sub-Saharan Africa by estimating the prevalence of HIV among adults (aged 15–49 years) and the corresponding number of people living with HIV from 2000 to 2017. Our analysis reveals substantial within-country variation in the prevalence of HIV throughout sub-Saharan Africa and local differences in both the direction and rate of change in HIV prevalence between 2000 and 2017, highlighting the degree to which important local differences are masked when examining trends at the country level. These fine-scale estimates of HIV prevalence across space and time provide an important tool for precisely targeting the interventions that are necessary to bringing HIV infections under control in sub-Saharan Africa.

## Abstract (web summary)

Fine-scale estimates of the prevalence of HIV in adults across sub-Saharan Africa reveal substantial within-country variation and local differences in both the direction and rate of change in the prevalence of HIV between 2000 and 2017.

## Main

HIV/AIDS is a leading cause of morbidity and mortality in sub-Saharan Africa^1,2^. In the nearly four decades since HIV was first recognized, scientific breakthroughs have transformed the once invariably fatal illness to one that can be successfully managed with lifelong anti-retroviral therapy (ART)^3^. Despite the rapid increase in the use of ART since the mid-2000s and the resulting decline in mortality, 34% of people in east and southern Africa and 60% of people in west and central Africa who are living with HIV are not currently receiving any treatment^4^ and HIV/AIDS remains the most common cause of death in sub-Saharan Africa^2^. The burden of the global HIV epidemic is disproportionately concentrated in sub-Saharan Africa, where—in 2017—75% of deaths and 65% of new infections occurred and where 71% of people living with HIV resided^1,2^.

The global community has repeatedly called for the end of the HIV epidemic. Millennium Development Goal 6 (Combat HIV/AIDS, malaria, and other diseases) included the target: “To halt by 2015 and have started to reverse the spread of HIV/AIDS”^5^. More recently, Sustainable Development Goal 3 (Ensure healthy lives and promote well-being for all at all ages)^6^ explicitly calls for the end of the epidemic by 2030. The Joint United Nations Programme on HIV/AIDS (UNAIDS) fast-track strategy has set diagnosis and treatment targets^7^ for 2020 and 2030, with the goal of markedly reducing both new infections and deaths by 2030. Despite these goals, a recent review of the state of HIV concluded that the world is not on track to end the HIV epidemic^8^. Moreover, global spending on HIV in sub-Saharan Africa peaked in 2013 and has since declined^9^, potentially compromising existing efforts to combat HIV.

Renewed commitment and new tools are required to get the world on track to bring HIV infection under control, in sub-Saharan Africa and globally. Local data on the current prevalence of HIV are such a tool, providing a means to target resources and interventions more efficiently.

## Precision public health and HIV

Country-level estimates of HIV prevalence, produced by both the Global Burden of Disease (GBD) study^1^ and UNAIDS^4^, highlight extensive differences in HIV prevalence between countries within sub-Saharan Africa. Further differences in HIV prevalence within national borders have long been recognized^10^ and recent evidence suggests that there is substantial within-country variation. Both GBD^1^ and UNAIDS^4^ estimate the prevalence of HIV at the first-level administrative subdivisions in select countries and a growing number of studies have examined subnational trends in the prevalence of HIV in a variety of locations and at various levels of granularity^11–19^ (Supplementary Table 1); these studies consistently find extensive within-country geographical variation in HIV prevalence.

Subnational variation in HIV prevalence has important implications for efforts to bring HIV infection under control, related to the treatment of people living with HIV as well as other prevention efforts that are aimed at directly reducing the number of new infections. Local estimates of HIV prevalence—particularly the number of people living with HIV—are useful for estimating the location-specific need for ART and other HIV-related services, and complement routinely collected clinical data that in some locations provide estimates of the number of diagnosed individuals living with HIV. In terms of prevention, areas in which HIV prevalence is high and ART coverage is low are likely to have a high incidence of HIV^20,21^. In the absence of local information on HIV incidence, knowledge of the variation in HIV prevalence can be used to better target prevention efforts to those areas with the greatest need. Recognizing the importance of subnational heterogeneity in the HIV epidemic, UNAIDS and funding agencies—including the US President’s Emergency Plan for AIDS Relief (PEPFAR) and the Global Fund to Fight AIDS, Tuberculosis and Malaria—have called for incorporating local data into strategies for addressing the HIV epidemic^22–24^.

**Extended Data Fig. 1:**
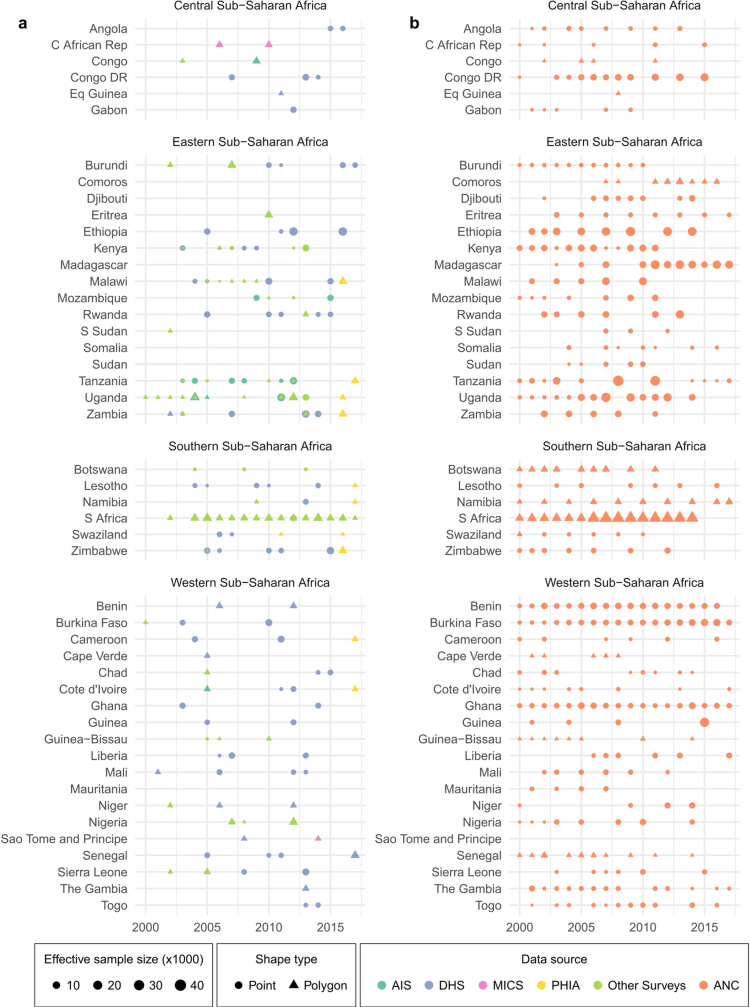
HIV prevalence data by region and country. **a**, **b**, HIV seroprevalence survey data (**a**) and ANC sentinel surveillance data (**b**) used in this analysis, by region and country. Colour indicates the data source. AIS, AIDS Indicator Survey; DHS, Demographic and Health Survey; MICS, Multiple Indicator Cluster Survey; PHIA, Population-based HIV Impact Assessment Survey. Shape type indicates whether a data source has point (GPS) or polygon location information. Size indicates the relative effective sample size for each source. A full list of data sources with additional details about data type (such as survey microdata and survey reports) and geographical details are provided in Supplementary Tables 2, 4.

**Extended Data Fig. 2:**
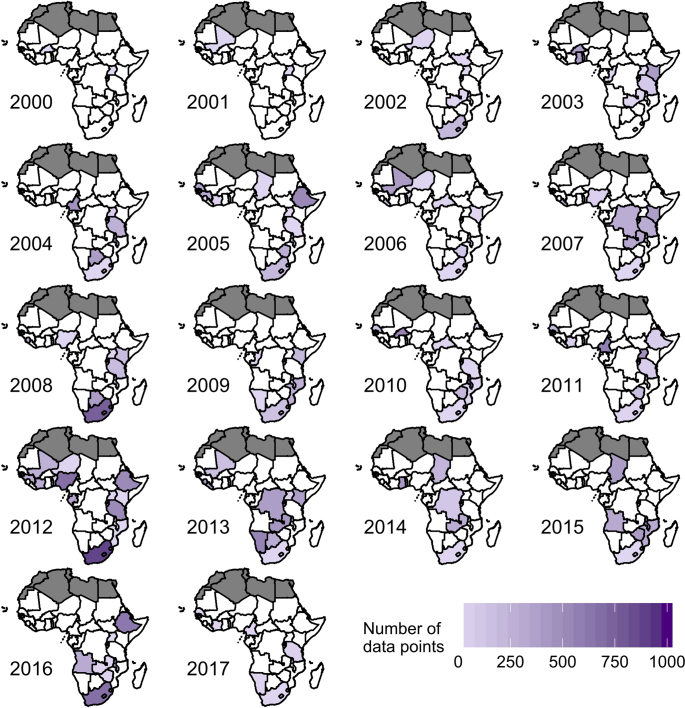
HIV seroprevalence survey data coverage by year. A data point is defined as a cluster or polygon used in the analysis for the given year. There are a total of 29,103 data points for the HIV seroprevalence surveys from 2000 to 2017. Countries in white have no available survey data in the given year. Countries in dark grey were not included in the analysis.

**Extended Data Fig. 3:**
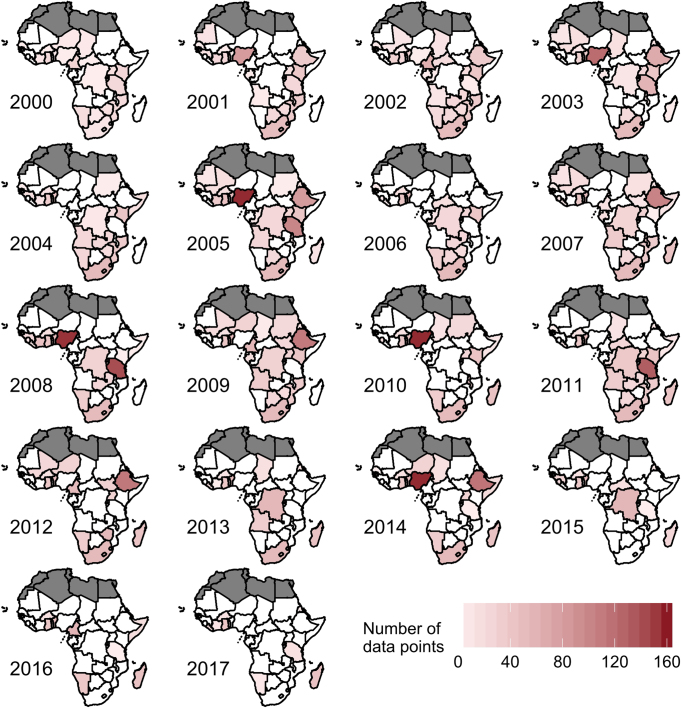
ANC sentinel surveillance data coverage by year. A data point is defined as an ANC sentinel surveillance site used in the analysis for the given year. A site may be a hospital, city or town, or administrative region. There are a total of 9,794 ANC data points from 2000 to 2017. Countries in white have no available ANC data in the given year. Countries in dark grey were not included in the analysis.

Although previous studies have examined subnational variation in HIV prevalence in select countries^11–19^ (Supplementary Table 1), there is—to our knowledge—no comprehensive and comparable set of subnational HIV prevalence estimates for all of sub-Saharan Africa. Moreover, for most countries, existing estimates are for a single year and use a single data source. Here we present comprehensive space–time estimates of HIV prevalence among adults aged 15–49 years who reside in each area on a 5 × 5-km grid across 47 countries in sub-Saharan Africa, annually from 2000 to 2017. For this analysis, we constructed a geolocated database of HIV prevalence data from 134 surveys in 41 countries and 9,794 site-years of sentinel surveillance of antenatal care clinics at 1,858 unique sites in 46 countries (Extended Data Figs. 1–3). We adapted existing Bayesian spatiotemporal methods to analyse these data and produce gridded estimates of HIV prevalence, calibrated to national estimates from the GBD^1^. We additionally combined grid-cell-level estimates of HIV prevalence with grid-cell-level estimates of the population^25,26^ aged 15–49 years to estimate the number of people living with HIV. Finally, for HIV prevalence, we calculated population-weighted averages of the grid-cell-level estimates to generate estimates for first-level administrative subdivisions (for example, provinces or regions) and second-level administrative subdivisions (for example, districts or departments) in each country. All estimates are publicly available from the Global Health Data Exchange (http://ghdx.healthdata.org/ihme-data/africa-hiv-prevalence-geospatial-estimates-2000-2017) and through a user-friendly data visualization tool (https://vizhub.healthdata.org/lbd/hiv).

## Widespread differences in HIV prevalence

**Fig. 1:**
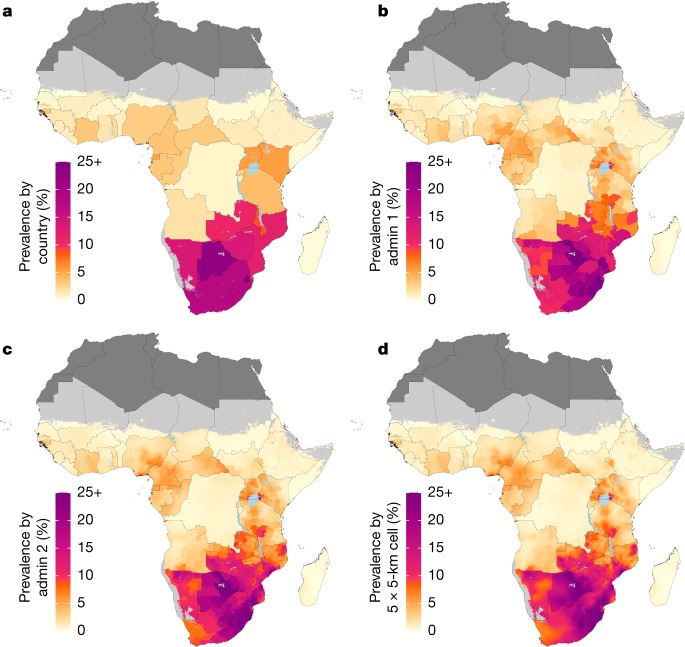
Prevalence of HIV in adults aged 15–49 in 2017. **a**–**d**, Prevalence of HIV among adults aged 15–49 in 2017 at the country level (**a**), first administrative subdivision level (admin 1; **b**), second administrative subdivision level (admin 2; **c**) and 5 × 5-km grid-cell level (**d**). Maps reflect administrative boundaries, land cover, lakes and population; grid cells with fewer than 10 people per 1 × 1 km, and classified as barren or sparsely vegetated, are coloured light grey^25,26,37–40^. Countries in dark grey were not included in the analysis.

**Extended Data Fig. 4:**
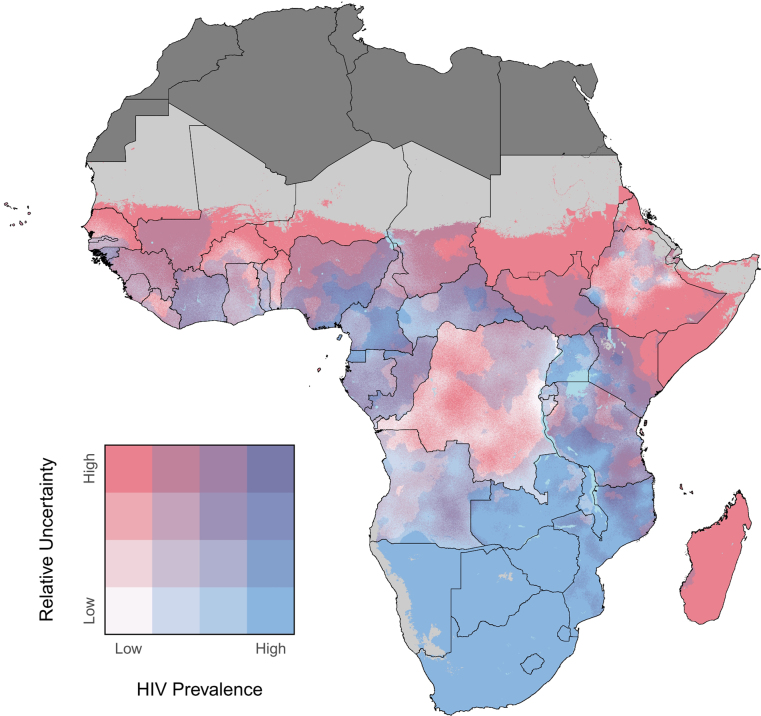
Relative uncertainty in HIV prevalence in adults aged 15–49 in 2017. Overlapping population-weighted quartiles of HIV prevalence and relative 95% uncertainty in 2017 at the 5 × 5-km grid cell level. Relative uncertainty is defined as the ratio of the width of the 95% uncertainty interval to the mean estimate. Maps reflect administrative boundaries, land cover, lakes and population; grid cells with fewer than 10 people per 1 × 1 km, and classified as barren or sparsely vegetated, are coloured light grey^25,26,37–40^. Countries in dark grey were not included in the analysis.

HIV prevalence varied substantially at the grid-cell level as well as among first and second administrative subdivisions throughout sub-Saharan Africa (Fig. 1, Extended Data Fig. 4 and Supplementary Figs. 1–4). This variation was apparent within countries with a relatively high overall HIV prevalence; for example, in Botswana (national prevalence, 22.8% (95% uncertainty interval, 19.8–26.1%)) prevalence among districts ranged from 15.1% (11.5–19.8%) in Ghanzi district to 27.7% (22.3–33.8%) in North-East district in 2017. This variation was also apparent in countries with a more moderate national HIV prevalence; for example, in Tanzania (national prevalence, 3.9% (3.6–4.3%)), prevalence among regions ranged from 0.4% (0.2–0.6%) in Kusini Pemba region to 9.1% (7.1–11.3%) in Njombe region in 2017. In countries in which levels of HIV prevalence are lower overall, the absolute differences among subnational units were necessarily smaller. However, in many instances, relative differences among subnational units remained large—for example, in the Democratic Republic of the Congo, in which national prevalence was 0.7% (0.6–0.9%), prevalence among second-level administrative subdivisions ranged from 0.3% (0.2–0.5%) in Lukaya district to 1.4% (0.8–2.3%) in the city Likasi in 2017. Most countries (36 out of 47) had a more than twofold difference in prevalence between the second-level administrative subdivisions with the lowest and highest estimated prevalence in 2017, and the largest difference was more than fivefold in 14 out of 47 countries.

At the country level (Fig. 1a), there was a clear divide between countries in southern sub-Saharan Africa (Botswana, Lesotho, Mozambique, Namibia, South Africa, Swaziland, Zambia and Zimbabwe), where estimated HIV prevalence exceeded 10% in 2017 and the rest of the continent, where prevalence was generally much lower. At subnational levels, however, there are areas outside of southern sub-Saharan Africa that nonetheless had a very high prevalence of HIV, including second-level administrative subdivisions in Kenya, Malawi, Uganda and Tanzania, where the estimated prevalence of HIV exceeded 10% in 2017 (Fig. 1c). Overall, the highest estimated prevalence observed in 2017 at the country level was 27.2% (23.6–31.1%) in Swaziland, compared to 28.3% (24.2–32.7%) in Lubombo province (Swaziland) at the first administrative level and 30.1% (25.2–35.4%) in Tikhuba constituency (Swaziland) at the second administrative level.

## Local temporal changes in HIV prevalence

**Fig. 2:**
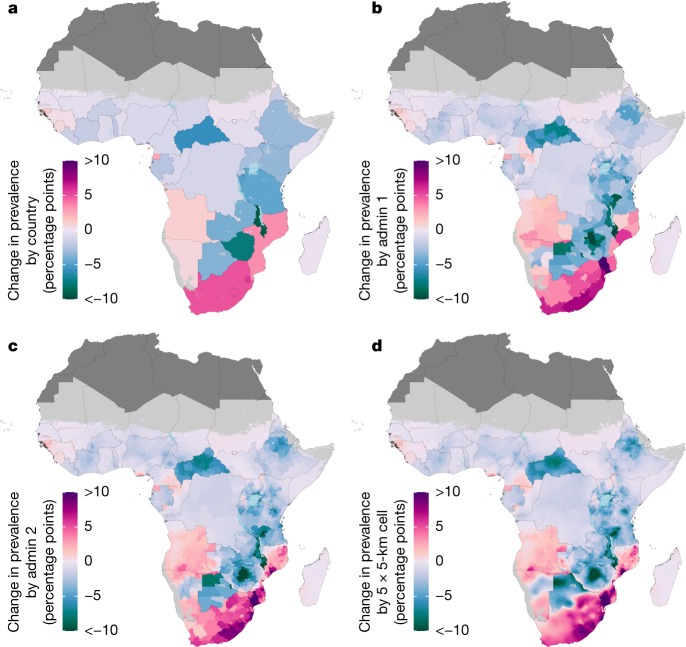
Change in HIV prevalence in adults aged 15–49 from 2000 to 2017. **a**–**d**, Absolute change in HIV prevalence among adults aged 15–49 between 2000 and 2017 at the country level (**a**), first administrative subdivision level (**b**), second administrative subdivision level (**c**) and 5 × 5-km grid-cell level (**d**). Maps reflect administrative boundaries, land cover, lakes and population; grid cells with fewer than 10 people per 1 × 1 km, and classified as barren or sparsely vegetated, are coloured light grey^25,26,37–40^. Countries in dark grey were not included in the analysis.

Between 2000 and 2017, estimated HIV prevalence at the country level increased in 15 out of 47 countries (Fig. 2a). At subnational levels, we estimated an increase in HIV prevalence in 22.9% of first-level administrative subdivisions (located in 24 countries) and in 25.0% of second-level administrative subdivisions (located in 28 countries) across sub-Saharan Africa (Fig. 2b, c; the posterior probability of an increase is shown in Supplementary Fig. 5). Although there was local heterogeneity, broad regional trends were apparent; the largest increases were found primarily in areas in coastal countries in southern sub-Saharan Africa and the largest decreases found primarily in a band stretching from Botswana to Kenya and in Central African Republic. Although in some places the direction and rate of change differed substantially on opposite sides of international borders (for example, between Botswana and South Africa), transnational patterns were also apparent—for example, the region that covered eastern South Africa and southern Mozambique.

There were substantial differences in both the direction and rate of change in HIV prevalence within many countries: 16 (34%) countries had areas in which the estimated HIV prevalence increased and areas in which the estimated HIV prevalence decreased among first-level administrative subdivisions (Fig. 2b). At the second administrative level this was true in 20 (42.6%) countries, and at the grid-cell level this was true in 28 (59.6%) countries (Fig. 2c, d). In some of these countries, the differences were substantial. For example, HIV prevalence declined by 5.8 percentage points (0.2–11.4 percentage points) in Manica district in Mozambique, whereas prevalence increased by 17.2 percentage points (9.3–26.1 percentage points) in Guija district. Similarly, prevalence declined by 14.3 percentage points (10.3–18.2 percentage points) in Chegutu district in Zimbabwe, whereas it increased by 0.6 percentage points (−4.1 to 5.0 percentage points) in Beitbridge district.

Changes in HIV prevalence from 2000 to 2017 in any given location were not generally linear or necessarily consistently in the same direction. Estimates of changes in prevalence over shorter periods within the overall 2000–2017 timeframe of this analysis highlight the variation within this period (Supplementary Figs. 6–8).

## Local trends in the number of people living with HIV

**Fig. 3:**
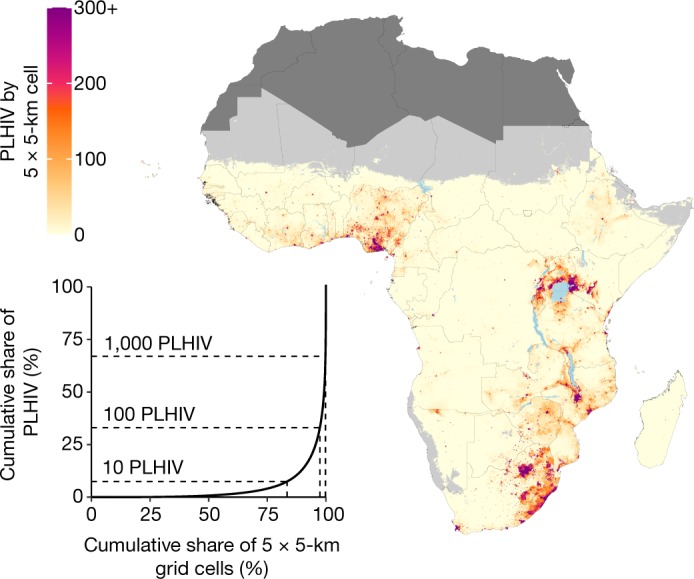
Number of people living with HIV for adults aged 15–49 in 2017. Number of people living with HIV (PLHIV) aged 15–49 in 2017 per 5 × 5-km grid cell (map) and Lorenz curve depicting the cumulative share of people living with HIV compared to the cumulative share of 5 × 5-km grid cells (inset). Maps reflect administrative boundaries, land cover, lakes and population; grid cells with fewer than 10 people per 1 × 1 km, and classified as barren or sparsely vegetated, are coloured light grey^25,26,37–40^. Countries coloured dark grey were not included in the analysis. In the inset, dotted lines indicate the cumulative share of people living with HIV and cumulative share of 5 × 5-km grid cells represented by grid cells with fewer than 10, 100 and 1,000 people living with HIV each.

Figure 3 shows the estimated number of people living with HIV by 5 × 5-km grid cell. As expected, given variation in population density and HIV prevalence, the number of people living with HIV per grid cell was highly variable and skewed: in 2017, we estimate that less than one person lives with HIV in 52.1% (50.6–53.4%) of grid cells, less than 10 people live with HIV in 83.8% (83.3–84.3%) of grid cells, less than 100 people living with HIV in 97.4% (97.2–97.5%) of grid cells and less than 1,000 people live with HIV in 99.8% (99.78–99.81%) of grid cells. Grid cells with large numbers of people living with HIV tend to have large populations in general. Although many of the grid cells that have the largest number of people living with HIV are also grid cells with very high prevalence (which are located primarily in southern and south-eastern sub-Saharan Africa), there are also grid cells with more moderate HIV prevalence but large numbers of people living with HIV; these are located primarily in western Africa.

A large proportion of people who are living with HIV are concentrated in a small number of grid cells with high spatial concentrations of people who are living with HIV. Approximately one-third (34.3% (33.0–35.7%)) of people living with HIV in sub-Saharan Africa live in the 0.2% of grid cells in which it is estimated that there are more than 1,000 people living with HIV. A similarly large proportion of people living with HIV is distributed throughout the larger number of grid cells that have more moderate spatial concentrations of people living with HIV: 32.0% (30.6–33.4%) of people with HIV live in grid cells in which there are estimated to be fewer than 100 people with HIV, and 7.2% (6.7–7.7%) of people with HIV reside in grid cells in which there are estimated to be fewer than 10 people with HIV. The total number of people living with HIV aged 15–49 years in sub-Saharan Africa increased by 3.0 (1.8–4.4) million between 2000 and 2017, from 17.0 million (16.3–17.8) to 20.1 million (19.0–21.2). This increase was due to a corresponding increase in population, as prevalence in sub-Saharan Africa as a whole declined over this same period, from 5.5% (5.2–5.7%) in 2000 to 4.0% (3.8–4.2%) in 2017. The increase in people living with HIV was larger in locations with high spatial concentrations of people with HIV compared to those with fewer people living with HIV: in 2017, the total number of people with HIV in grid cells in which there are estimated to be fewer than 100 people with HIV was nearly identical (6.4 million (6.2–6.6)) to the number in 2000 (6.5 million (6.3–6.6)). However, the number of people living with HIV in grid cells in which there are estimated to be more than 1,000 people increased by 37.5%, from 5.0 million (4.7–5.3) to 6.9 million (6.3–7.5).

## Discussion

This study provides a comprehensive quantification of subnational trends in HIV prevalence and the number of people living with HIV in sub-Saharan Africa. These estimates highlight substantial differences between and within countries in levels and trends in HIV prevalence and the spatial concentration of people living with HIV. For discussion of the advantages of this analysis compared to earlier analyses, important limitations of the present analysis and potential future directions, see Supplementary Discussion.

Subnational estimates of HIV prevalence can be used to more efficiently target resources and interventions. The WHO (World Health Organization) recommends ART for all people living with HIV^27^, and the UNAIDS fast-track strategy emphasizes the importance of treatment and diagnosis^7^. Estimates of the prevalence of HIV and the number of people living with HIV at local levels provide important information about the number of people who are potentially in need of diagnosis and treatment services. Additionally, in the absence of local information on HIV incidence, information about HIV prevalence can be used to target primary prevention strategies: modelling studies that compare geographically targeted to non-geographically targeted prevention strategies have found that geographically targeted strategies are more efficient in preventing new HIV infections under the same budgetary constraints^11,28^. Moreover, previous research has highlighted the potential role of geographical ‘hot spots’ as a source of HIV transmission both locally and further afield, which suggests that targeted prevention strategies may reduce the incidence of HIV not only in targeted areas but also more broadly^29,30^.

Our analysis highlights several challenges to bringing HIV infection under control in Africa. Growing population size coupled with continued high incidence^1,4^ of new HIV infections and increased life expectancy among people living with HIV^31–34^ has led to an increase in the number of people living with HIV in sub-Saharan Africa since 2000. Despite this increase, spending on HIV in sub-Saharan Africa has declined in recent years, largely as a result of a reduction in development assistance for health^9^. Our estimates also highlight the diversity of the HIV epidemic: although a large number of people living with HIV are concentrated in a few select areas (Fig. 3), a similarly large number are living in areas with a relatively low spatial concentration of people living with HIV. The most effective treatment and prevention strategies probably differ between areas in which many people live with HIV and those with a smaller number of people living with HIV, and economies of scale may be harder to realize in the latter case. Nonetheless, it is essential to ensure that people living with HIV have access to appropriate health services regardless of their location.

The results of this analysis describe a multifaceted picture of patterns of changing HIV prevalence across sub-Saharan Africa, with many areas experiencing increases over the same period in which other areas experienced declines. Changes in HIV prevalence are the outcome of a complex interaction between incidence, mortality and migration patterns. Globally, the large-scale expansion of ART coverage has reduced mortality among people living with HIV, offsetting declines in incidence and resulting in an overall increase in HIV prevalence since 2000^1,4,35^. At the region and country levels, trends in mortality and incidence have varied, which has resulted in differing trends in the prevalence of HIV^1,4,35^. Exploration of this dynamic at a subnational level is warranted, although it is complicated by the relative lack of directly observed empirical data on HIV incidence and mortality in sub-Saharan Africa^36^. Nonetheless, existing evidence indicates that subnational increases in prevalence should not be interpreted as inherently alarming without additional consideration of incidence and mortality trends.

Despite progress in recent decades, HIV continues to impose a substantial health burden on countries in sub-Saharan Africa. The estimates from this analysis highlight the degree to which the effect of this epidemic varies, even within countries. These local data provide a new tool for policymakers, programme implementers and researchers to use to assess local needs, efficiently target interventions and ultimately work towards bringing HIV infection under control in Africa.

## Methods

### Data reporting

No statistical methods were used to predetermine sample size. The experiments were not randomized and the investigators were not blinded to allocation during experiments and outcome assessment.

### Overview

**Extended Data Fig. 5:**
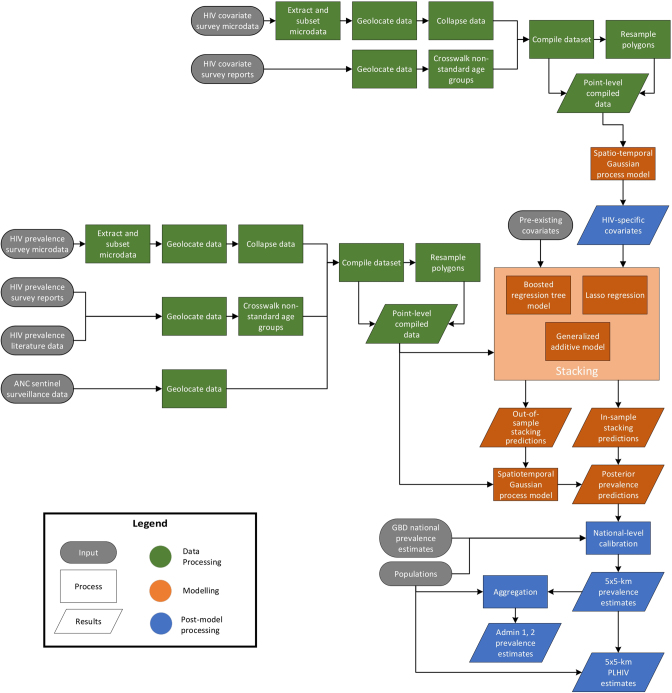
Analytic process overview. The process used to produce HIV prevalence estimates among adults in sub-Saharan Africa involved three main parts. In the data-processing steps (green), data were identified, extracted and prepared for use in the HIV prevalence model and in covariate models. In the modelling phase (orange), we used these data and covariates in a stacked generalization ensemble model and spatiotemporal Gaussian process model. In the post-processing phase (blue), we calibrated the prevalence estimation to match GBD 2017 estimates at the national level, aggregated prevalence estimates to the first- and second-level administrative subdivisions in each country and calculated the number of people living with HIV.

Our study follows the Guidelines for Accurate and Transparent Health Estimates Reporting (GATHER). This analysis provides estimates of HIV prevalence among adults aged 15–49 on a 5 × 5-km grid in 47 countries in sub-Saharan Africa, with annual resolution, from 2000 to 2017. The period of 2000–2017 and the age group of 15–49 years were selected to optimize the contemporaneousness of the estimates and to maximize data availability—there were relatively few large-scale seroprevalence surveys conducted before 2000, and most seroprevalence surveys focus on adults, in which 15–49 years was the most commonly reported age range. The methodology used here is similar to that used for previous analyses of mortality in children under 5 years of age^41^, child growth failure^42^ and education^43^ in Africa. We used a 5 × 5-km grid for consistency with these previous analyses; to align with the resolution available for pre-existing covariates incorporated in this analysis; and for flexibility in aggregating these estimates to other levels of interest (for example, first- and second-order administrative subdivisions). Extended Data Figure 5 provides an overview of the analytic process. Each step is described below and additional details are available in the Supplementary Information, including a discussion of the limitations of this approach.

### HIV data

We compiled a dataset of 29,103 data points from 134 seroprevalence surveys in 41 countries and 9,794 data points from sentinel surveillance of antenatal care clinics (ANC data) in 46 countries. Data from seroprevalence surveys were originally in one of three forms: survey microdata (that is, individual-level survey responses), survey reports or published literature (Supplementary Table 2). For surveys with available microdata, we extracted variables related to age, HIV blood test result, location and survey weights. After subsetting the data to ages 15–49 years and excluding rows with missing information on any of these variables, we collapsed the data by calculating the weighted HIV prevalence at the finest spatial resolution available. Ideally, this was at the level of the GPS coordinates that represent the location of a survey cluster, but in instances for which GPS data were not available, the smallest areal unit (termed a polygon) possible was used instead, typically representing an administrative subdivision. For surveys for which microdata were unavailable but for which estimates with some subnational resolution were provided in a report or published literature, we extracted these estimates along with information about the sample size and location. Where possible, these data were matched to a specific set of GPS coordinates, and otherwise were matched to a polygon, which most-often represented an administrative subdivision. In some instances, estimates extracted from reports or published literature were for age groups other than 15–49 years (34 sources representing 1.76% of the total effective sample size; Supplementary Table 3). In these instances, we used a cross-walking model—that is, an approach for linking disparate data sources (in this case data sources reporting for different age groups)—that leveraged existing microdata and linear regression to translate the prevalence in the reported age range to the standard 15–49 age range (Supplementary Information, section 2.3).

ANC data were primarily derived from national HIV estimate files developed by national teams and compiled and shared via UNAIDS^44^, and supplemented with data derived from sentinel surveillance country reports (Supplementary Table 4). In both instances, we extracted information on HIV prevalence and sample size by site and year. Sites were geolocated to specific GPS coordinates where possible and otherwise to a polygon that represents an administrative subdivision.

In instances in which data were matched to a polygon rather than specific GPS coordinates, we resampled these data to mimic point data. Specifically, for each observation, we randomly sampled 10,000 candidate locations within the associated polygon with a probability proportional to the population and then used *k*-means clustering to generate a reduced set of locations based on the centroid of each *k*-means cluster. Each of these resulting pseudo-points was assigned the HIV prevalence observed for the polygon as a whole, and the sample size was set to the observed sample size for the polygon as a whole multiplied by the fraction of candidate locations that belonged to that *k*-means cluster. Weighting by sample size, 78.0% of all data (including 61.1% of survey data and 83.5% of ANC data) were associated with GPS coordinates, and the remaining data were associated with polygons and were analysed using this approach.

### Covariates

**Extended Data Fig. 6:**
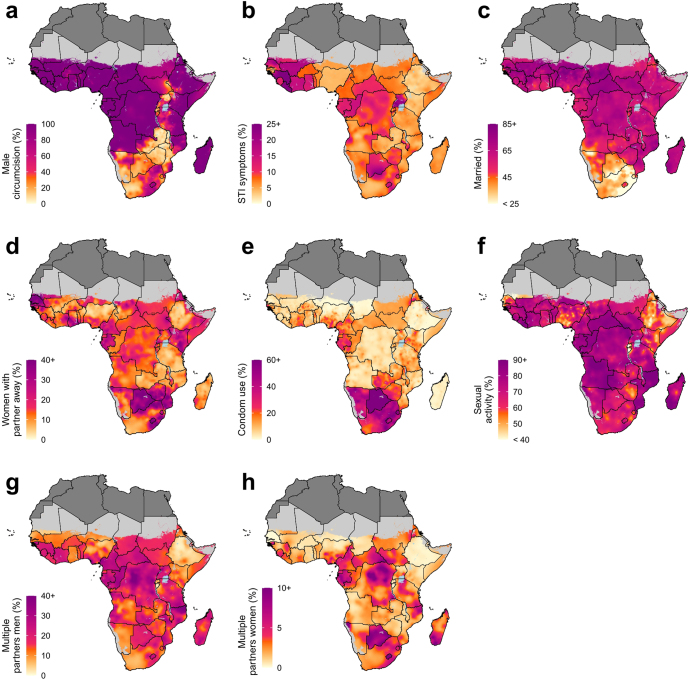
Prevalence of covariates at 5 × 5-km grid cell level in 2017. **a**–**h**, Maps of HIV-specific covariates in 2017 include prevalence of male circumcision (**a**), prevalence of signs and symptoms of sexually transmitted infections (**b**), prevalence of marriage or living as married (**c**), prevalence of partner living elsewhere among women (**d**), prevalence of condom use during the most recent sexual encounter (**e**), prevalence of sexual activity among young women (**f**), prevalence of multiple partners among men in the past year (**g**) and prevalence of multiple partners among women in the past year (**h**). Maps reflect administrative boundaries, land cover, lakes and population; grid cells with fewer than 10 people per 1 × 1 km, and classified as barren or sparsely vegetated, are coloured light grey^25,26,37–40^. Countries in dark grey were not included in the analysis.

This analysis included five pre-existing covariates: (1) travel time to the nearest settlement of more than 50,000 inhabitants; (2) total population; (3) night-time lights; (4) urbanicity; and (5) malaria incidence (Supplementary Table 5). In addition, eight covariates were constructed explicitly for this analysis owing to their known association with HIV prevalence and data availability: (1) prevalence of male circumcision (all forms); (2) prevalence of self-reported STI symptoms; (3) prevalence of marriage or living with a partner as married; (4) prevalence of one’s current partner living elsewhere; (5) prevalence of condom use at last sexual encounter; (6) prevalence of reporting ever having had intercourse among young adults; and (7) and (8) prevalence of multiple partners in the past year for men and for women (Extended Data Fig. 6). These eight covariates were constructed based on survey data collected and analysed analogously to the HIV data (described above), and using geostatistical models similar to those described in the next section (Supplementary Table 6 and Supplementary Figs. 9–16). In addition, calendar year was used as a covariate.

### Statistical model

#### Covariate stacking

**Extended Data Fig. 7:**
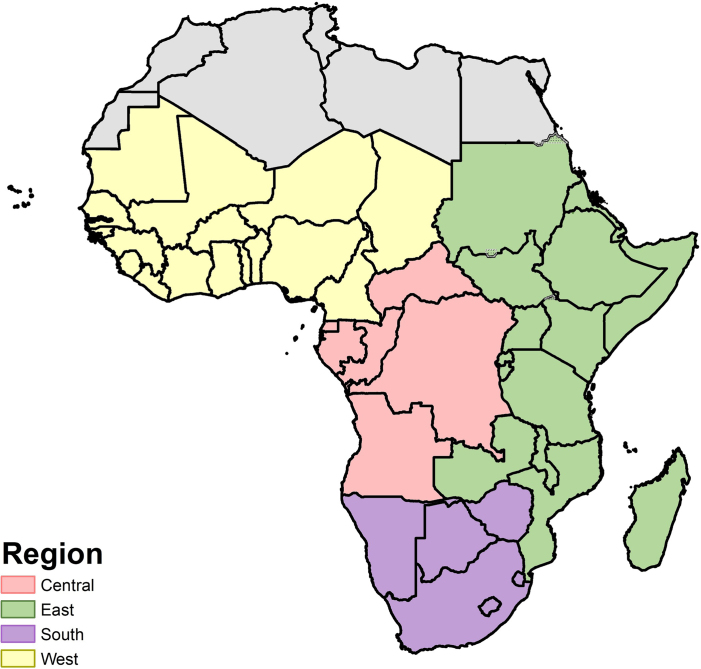
Modelling regions. Modelling regions were defined as the four GBD regions in sub-Saharan Africa: central, east, south and west. Sudan was included in the east sub-Saharan Africa region for this analysis (in GBD, it is included in the North Africa and Middle East region). Countries in grey were not included in the analysis.

An ensemble covariate modelling approach was implemented to capture possible nonlinear effects and complex interactions among these covariates^45^. For each modelling region (Extended Data Fig. 7), three sub-models were fitted to the HIV survey data with the covariates as explanatory predictors: generalized additive models, boosted regression trees and lasso regression. Each sub-model was fitted using fivefold cross-validation to avoid overfitting, and the out-of-sample predictions from across the five folds were compiled into a single set of predictions that were used to fit the geostatistical model described below. In addition, each sub-model was also fitted to the full dataset to generate a complete set of in-sample predictions that were subsequently used when generating predictions from the geostatistical model (Supplementary Figs. 17–19).

#### Geostatistical model

We modelled HIV prevalence using a spatially and temporally explicit generalized linear mixed effects model:

$${Y}_{i,t}\sim {\rm{b}}{\rm{i}}{\rm{n}}{\rm{o}}{\rm{m}}{\rm{i}}{\rm{a}}{\rm{l}}({p}_{i,t},{N}_{i,t})$$

$${\rm{logit}}\left({p}_{i,t}\right)=\;{\beta }_{0}+\;{{\boldsymbol{\beta }}}_{1}{{\boldsymbol{X}}}_{i,t}+{\gamma }_{c\left[i\right]}+\;{Z}_{i,t}+\;{\epsilon }_{i,t}+({\beta }_{2}+\;{U}_{i}){I}_{{\rm{ANC}}}$$

$${\gamma }_{c[i]}\sim {\rm{n}}{\rm{o}}{\rm{r}}{\rm{m}}{\rm{a}}{\rm{l}}(0,\,{\sigma }_{{\rm{c}}{\rm{o}}{\rm{u}}{\rm{n}}{\rm{t}}{\rm{r}}{\rm{y}}}^{2})$$

$${Z}_{i,t}\sim {\rm{G}}{\rm{P}}(0,\,{{\rm{\Sigma }}}_{{\rm{s}}{\rm{p}}{\rm{a}}{\rm{c}}{\rm{e}}}\otimes \,{{\rm{\Sigma }}}_{{\rm{t}}{\rm{i}}{\rm{m}}{\rm{e}}})$$

$${\epsilon }_{i,t}\sim {\rm{n}}{\rm{o}}{\rm{r}}{\rm{m}}{\rm{a}}{\rm{l}}(0,\,{\sigma }_{{\rm{n}}{\rm{u}}{\rm{g}}{\rm{g}}{\rm{e}}{\rm{t}}}^{2})$$

$${U}_{i}\sim {\rm{G}}{\rm{P}}(0,\,{{\rm{\Sigma }}}_{{\rm{s}}{\rm{p}}{\rm{a}}{\rm{c}}{\rm{e}}})$$

in which ∼ denotes ‘distributed as’. We modelled the number of HIV-positive individuals (*Y*_*i*,*t*_) among a sample (*N*_*i*,*t*_) in location *i* and year *t* as a binomial variable. This model specified logit-transformed HIV prevalence (*p*_*i*,*t*_) as a linear combination of a regional intercept (*β*_0_), covariate effects (***β***_1_***X***_*i*,*t*_), country random effects (*γ*_*c*[*i*]_), spatially and temporally correlated random effects (*Z*_*i*,*t*_) and an uncorrelated error term or nugget effect $\left({\epsilon }_{i,t}\right)$. HIV prevalence as measured by sentinel surveillance of antenatal care clinics is known to be biased as a measure of HIV prevalence in the general adult population, because it only covers pregnant women who attend ANC, compared to all adult men and women^46,47^. In instances in which data in our model were derived from ANC sentinel surveillance (*I*_ANC_ = 1), our model allowed for this bias using a fixed term *(β*_2_) that captured the overall mean bias and a spatially varying term (*U*_*i*_) that captured local differences in the extent of this bias. In this model, the spatially and temporally correlated random effect (*Z*_*i*,*t*_) was modelled as a Gaussian process with mean 0 and a covariance matrix given by the Kronecker product of a spatial Matérn covariance function $\left({{\rm{\Sigma }}}_{{\rm{space}}}\right)$ and a temporal first-order autoregressive covariance function $\left({{\rm{\Sigma }}}_{{\rm{time}}}\right)$. *U*_*i*_ was modelled as a Gaussian process with mean 0 and spatial Matérn covariance $\left({{\rm{\Sigma }}}_{{\rm{space}}}\right)$. Sensitivity analyses were carried out to assess sensitivity to hyper-prior specification and are described in detail in the Supplementary Information, section 4.2.

This model was fitted in R-INLA^48^ using the stochastic partial differential equation^49^ approach to approximate the continuous spatial and spatio-temporal Gaussian random fields (*U*_*i*_ and *Z*_*i*,*t*_, respectively). Owing to computational constraints, and to allow for regional differences in the relationship between the covariates and HIV prevalence, as well as differences in the temporal and spatial autocorrelation in HIV prevalence, separate models were fitted for each of the four regions (Extended Data Fig. 7). From each fitted model, we generated 1,000 draws from the approximated joint posterior distribution of all model parameters and used these to construct 1,000 draws of *p*_*i*,*t*_, setting *I*_ANC_ to 0. Fivefold cross-validation was used to assess model performance and to compare among a number of alternative models that use covariates, ANC data and polygon data in a variety of ways (Supplementary Figs. 20–25 and Supplementary Information, section 4.3).

#### Post-estimation

To take advantage of the more structured modelling approach and additional national-level data used by GBD 2017, we performed post hoc calibration of our estimates to the corresponding national-level GBD estimates^1^. For each country and year in our analysis, we defined a raking factor equal to the ratio of the GBD estimate for this country and year to the population-weighted posterior mean HIV prevalence in all grid cells within this country and year (Supplementary Fig. 26). These raking factors were then used to scale each draw of HIV prevalence for each grid cell within that GBD geography and year. Point estimates for each grid cell were calculated as the mean of the scaled draws, and 95% uncertainty intervals were calculated as the 2.5th and 97.5th percentiles of the scaled draws. Grid cells that crossed international borders within modelling regions were fractionally allocated to multiple countries in proportion to the covered area during this process.

In addition to estimates of HIV prevalence on a 5 × 5-km grid, we constructed estimates of HIV prevalence for first- and second-level administrative subdivisions by calculating population-weighted averages of prevalence for all grid cells within a given area. This process was carried out for each of the 1,000 posterior draws (after calibration to GBD) with final point estimates derived from the mean of these draws and uncertainty intervals from the 2.5th and 97.5th percentiles. Additionally, estimates of the number of people living with HIV for each grid cell were derived by multiplying estimated prevalence in each grid cell by the corresponding population estimate from WorldPop^25,26^, which was also calibrated to match GBD 2017^50^ (Supplementary Information, section 4.4). As with calibration, grid cells that crossed borders were fractionally allocated to multiple areas when calculating aggregated prevalence estimates and estimates of people living with HIV.

Although the model makes predictions for all locations that are covered by available covariates, all final model outputs for which the land cover was classified as barren or sparsely vegetated on the basis of MODIS satellite data and for which the total population density was less than 10 individuals per 1 × 1 km in 2015 were masked for improved clarity when communicating with data specialists and policymakers.

### Limitations

This analysis is subject to several limitations (further discussed in the Supplementary Information, section 5.2). Most importantly, the accuracy of our estimates is dependent on the quantity and quality of the underlying data. We have constructed a large database of geolocated HIV prevalence data for the purposes of this analysis. Nonetheless, important gaps in data coverage, both spatial and temporal, remain (Extended Data Figs. 1–3). Data quality is also likely to be variable and may be problematic for some data sources or locations. For HIV seroprevalence surveys, potential non-response bias is a particular concern^51^ and the quality of the underlying data that are used to generate the covariate surfaces may also be suboptimal in some situations—for example, if cultural context influences the interpretation of a survey question or the response to potentially sensitive questions regarding sexual behaviour^52^. The information on locations that is associated with the data used in this analysis is also subject to some error and uncertainty. For example, in most surveys, GPS coordinates are randomly displaced (typically by 2–5 km) to protect the confidentiality of respondents ^53^ and some data sources have relatively non-specific location information (for example, districts or provinces instead of GPS coordinates). Primarily as a consequence of gaps in data coverage as well as the relative sparsity and small sample sizes in existing data sources disaggregated at small subnational levels, our estimates at the grid cell level—and to a lesser extent at the second and first administrative level—are associated with considerable uncertainty (Extended Data Fig. 4 and Supplementary Figs. 1–4). In the future, additional data collection, increased access to existing datasets (including detailed location information) and new strategies for using non-traditional data sources such as routine healthcare facility data^54^ will be needed to improve the precision of these estimates at all levels.

The modelling strategy incorporates a number of assumptions, which—if incorrect—may lead to error. Additionally, the model fitting and prediction strategy used an integrated nested Laplace approximation to the posterior distribution, as implemented in R-INLA^48^, as well as further approximations to generate predictions; these approximations may also introduce error. Although it is difficult to assess the effect of these assumptions and approximations, our validation analyses showed that our final model had minimal bias and a good coverage of the 95% prediction intervals, which provides some reassurance that the approximation method used—as well as other potential sources of error—did not result in appreciable bias or poorly described uncertainty in our reported estimates.

### Reporting summary

Further information on research design is available in the Nature Research Reporting Summary linked to this paper.

## Online content

Any methods, additional references, Nature Research reporting summaries, source data, statements of data availability and associated accession codes are available at 10.1038/s41586-019-1200-9.

## Supplementary information

Supplementary Information This file contains Supplementary Text, Data and Methods, a Supplementary Discussion, Supplementary References, Supplementary Figures 1–27 and Supplementary Tables 1–9.

Reporting Summary

## Data Availability

The findings of this study are supported by data that are available in public online repositories, data that are publicly available upon request from the data provider and data that are not publicly available owing to restrictions by the data provider and that were used under a license for the current study (including select data sources in Burkina Faso, Burundi, Chad, Eritrea, Nigeria, Sierra Leone, Uganda and Zambia, as indicated in Supplementary Tables 2, 6). A detailed description of data sources can be found in Supplementary Tables 2, 4–6. More information about each data source is available on the Global Health Data Exchange (http://ghdx.healthdata.org/), including information about the data provider and links to where the data can be accessed or requested (where available). Administrative boundaries were retrieved from the Global Administrative Unit Layers dataset, implemented by FAO within the CountrySTAT and Agricultural Market Information System projects^37^. Land cover data were retrieved from the online Data Pool, courtesy of the NASA EOSDIS Land Processes Distributed Active Archive Center, USGS/Earth Resources Observation and Science Center^38^. Lakes were retrieved from the Global Lakes and Wetlands Database, courtesy of the World Wildlife Fund and the Center for Environmental Systems Research^39,40^. Populations were retrieved from WorldPop^25,26^. All estimates produced as part of this analysis are publicly available from the Global Health Data Exchange (http://ghdx.healthdata.org/ihme-data/africa-hiv-prevalence-geospatial-estimates-2000-2017) and via a user-friendly data visualization tool (https://vizhub.healthdata.org/lbd/hiv).

## Code availability

All code used for these analyses is publicly available at https://github.com/ihmeuw/lbd/tree/hiv-africa-2019.
