# Supplementary material for: Mapping HIV prevalence in sub-Saharan Africa between 2000 and 2017
Source: Nature. 2019 May 15;570(7760):189–93. doi: 10.1038/s41586-019-1200-9 (PMC6601349; doi:10.1038/s41586-019-1200-9)
Supplement: Supplementary file 1 — This file contains Supplementary Text, Data and Methods, a Supplementary Discussion, Supplementary References, Supplementary Figures 1–27 and Supplementary Tables 1–9. [file 41586_2019_1200_MOESM1_ESM.pdf]

nature

SUPPLEMENTARY INFORMATION

https://doi.org/10.1038/s41586-019-1200-9

In the format provided by the authors and unedited.

Mapping HIV prevalence in sub-Saharan Africa between 2000 and 2017

Laura Dwyer-Lindgren[1], Michael A. Cork[1], Amber Sligar[1], Krista M. Steuben[1], Kate F. Wilson[1], Naomi R. Provost[1], Benjamin K. Mayala[2], John D. VanderHeide[1], Michael L. Collison[1], Jason B. Hall[1], Molly H. Biehl[1], Austin Carter[1], Tahvi Frank[1], Dirk Douwes-Schultz[1], Roy Burstein[1], Daniel C. Casey[1], Aniruddha Deshpande[1], Lucas Earl[1], Charbel El Bcheraoui[1], Tamer H. Farag[1], Nathaniel J. Henry[1], Damaris Kinyoki[1], Laurie B. Marczak[1], Molly R. Nixon[1], Aaron Osgood-Zimmerman[1], David Pigott[1], Robert C. Reiner Jr[1], Jennifer M. Ross[1,3,4], Lauren E. Schaeffer[1], David L. Smith[1], Nicole Davis Weaver[1], Kirsten E. Wiens[1], Jeffrey W. Eaton[1,5], Jessica E. Justman[6,7], Alex Opio[8], Benn Sartorius[9], Frank Tanser[10,11,12,13], Njeri Wabiri[14], Peter Piot[15], Christopher J. L. Murray[1] & Simon I. Hay[1]*

[1]Institute for Health Metrics and Evaluation, University of Washington, Seattle, WA, USA. [2]DHS program, ICF International, Rockville, MD, USA. [3]Department of Global Health, University of Washington, Seattle, WA, USA. [4]Department of Medicine, University of Washington, Seattle, WA, USA. [5]Department of Infectious Disease Epidemiology, Imperial College London, London, UK. [6]ICAP, Mailman School of Public Health, Columbia University, New York, NY, USA. [7]Vagelos College of Physicians and Surgeons, Columbia University, New York, NY, USA. [8]Medireal Investment Uganda, Entebbe, Uganda. [9]Public Health Medicine, School of Nursing and Public Health, College of Health Sciences, University of KwaZulu-Natal, Durban, South Africa. [10]School of Nursing and Public Health, University of KwaZulu-Natal, Durban, South Africa. [11]Africa Health Research Institute, KwaZulu-Natal, South Africa. [12]Centre for the AIDS Programme of Research in South Africa (CAPRISA), University of KwaZulu-Natal, Durban, South Africa. [13]Research Department of Infection & Population Health, University College London, London, UK. [14]HIV/AIDS, STIs & TB Research Programme, Human Sciences Research Council, Pretoria, South Africa. [15]London School of Hygiene & Tropical Medicine, London, UK. *e-mail: sihay@uw.edu

Supplementary Information:
Mapping HIV prevalence in sub-Saharan Africa between 2000 and 2017

Contents

1 Compliance with the Guidelines for Accurate and Transparent Health Estimates Reporting (GATHER) .. 5
2 HIV data sources and data processing 7
2.1 Seroprevalence surveys 7
2.1.1 Data identification strategy 7
2.1.2 Data processing for microdata 7
2.1.3 Data processing for reports 7
2.2 Literature review 8
2.2.1 Search strategy and exclusion criteria 8
2.2.2. Data processing 9
2.3 Age cross-walk 9
2.4 Antenatal care (ANC) sentinel surveillance 10
2.4.1 Data sources 10
2.4.2 Data processing 11
2.5 Polygon resampling 11
3 Covariate and auxiliary data 12
3.1 Pre-existing covariates 12
3.2 Covariates constructed for this analysis 12
3.2.1 Covariate selection criteria and definitions 12
3.2.2 Covariate data 14
3.2.3 Covariate modelling 16
3.3 Administrative boundaries 16
3.4 Gridded population 16
4 Statistical model 17
4.1 Covariate stacking 17
4.2 Geostatistical model 17
4.2.1 Model description 17
4.2.2 Prior sensitivity analysis 19
4.2.3 Model fitting and prediction 21
4.3 Model validation 22
4.3.1 Validation strategy 22
4.3.2 Sensitivity analyses 22
4.4 Post-estimation 24

4.4.1 Calibration to Global Burden of Disease 2017 24
4.4.2 Aggregation to first- and second-level administrative subdivisions 24
4.4.3 Calculating people living with HIV (PLHIV) 25
5 Supplementary Discussion 25
5.1 Advances compared to previous analyses 25
5.2 Limitations 25
5.3 Future directions 27
References 29
Supplementary Figures 33
Supplementary Figure 1: HIV prevalence, adults ages 15-49, 2000 33
Supplementary Figure 2: HIV prevalence, adults ages 15-49, 2005 34
Supplementary Figure 3: HIV prevalence, adults ages 15-49, 2010 35
Supplementary Figure 4: HIV prevalence, adults ages 15-49, 2017 36
Supplementary Figure 5: Posterior probability of an increase in HIV prevalence, adults ages 15-49, 2000–2017 37
Supplementary Figure 6: Change in HIV prevalence, adults ages 15-49, 2000–2005 39
Supplementary Figure 7: Change in HIV prevalence, adults age 15-49, 2005–2010 40
Supplementary Figure 8: Change in HIV prevalence, adults ages 15-49, 2010–2017 41
Supplementary Figure 9: Prevalence of male circumcision in 2000, 2005, 2010, and 2017 42
Supplementary Figure 10: Prevalence of signs and symptoms of sexually transmitted infections in 2000, 2005, 2010, and 2017 43
Supplementary Figure 11: Prevalence of marriage or living as married in 2000, 2005, 2010, and 2017 44
Supplementary Figure 12: Prevalence of partner living elsewhere among women in 2000, 2005, 2010, and 2017 45
Supplementary Figure 13: Prevalence of condom use during most recent sexual encounter in 2000, 2005, 2010, and 2017 46
Supplementary Figure 14: Prevalence of sexual activity among young adults in 2000, 2005, 2010, and 2017 47
Supplementary Figure 15: Prevalence of multiple partners among men in the past year in 2000, 2005, 2010, and 2017 48
Supplementary Figure 16: Prevalence of multiple partners among women in the past year in 2000, 2005, 2010, and 2017 49
Supplementary Figure 17: HIV prevalence predictions from the boosted regression tree model in 2000, 2005, 2010, and 2017 50

Supplementary Figure 18: HIV prevalence predictions from the generalized additive model in 2000, 2005, 2010, and 2017 51
Supplementary Figure 19: HIV prevalence predictions from the lasso regression model in 2000, 2005, 2010, and 2017 52
Supplementary Figure 20: Validation metrics from the covariate sensitivity analysis 53
Supplementary Figure 21: Predictions comparison from the covariate sensitivity analysis 54
Supplementary Figure 22: Validation metrics from the ANC data and bias correction sensitivity analysis 56
Supplementary Figure 23: Predictions comparison from the ANC data and bias correction sensitivity analysis 57
Supplementary Figure 24: Validation metrics from the polygon data sensitivity analysis 58
Supplementary Figure 25: Predictions comparison from the polygon data analysis 59
Supplementary Figure 26: HIV prevalence raking factors 61
Supplementary Figure 27: Space mesh for geostatistical models 62
Supplementary Tables 63
Supplementary Table 1: Previous publications of subnational estimates of HIV prevalence in sub-Saharan Africa 64
Supplementary Table 2: HIV seroprevalence survey data 71
Supplementary Table 3: Data requiring age cross-walk 97
Supplementary Table 4: ANC sentinel surveillance data 100
Supplementary Table 5: Sources for pre-existing covariates 113
Supplementary Table 6: HIV covariate survey data 114
Supplementary Table 7: Surveys excluded from this analysis 199
Supplementary Table 8: Model prior sensitivity analyses 206
Supplementary Table 9: Fitted model parameters 208

1 Compliance with the Guidelines for Accurate and Transparent Health Estimates Reporting (GATHER)

Item #	Checklist item	Description of Compliance
**Objectives and funding**		
1	Define the indicator(s), populations (including age, sex, and geographic entities), and time period(s) for which estimates were made.	Precision public health and HIV section
2	List the funding sources for the work.	Acknowledgments section
**Data Inputs**		
For all data inputs from multiple sources that are synthesized as part of the study:		
3	Describe how the data were identified and how the data were accessed.	Methods section; SI sections 2.1, 2.2, 2.4, 3.2
4	Specify the inclusion and exclusion criteria. Identify all ad-hoc exclusions.	SI sections 2.2, 2.4, 3.2; Supplementary Table 7
5	Provide information on all included data sources and their main characteristics. For each data source used, report reference information or contact name/institution, population represented, data collection method, year(s) of data collection, sex and age range, diagnostic criteria or measurement method, and sample size, as relevant.	Supplementary Tables 2-7, http://ghdx.healthdata.org/ihme-data/africa-hiv-prevalence-geospatial-estimates-2000-2017
6	Identify and describe any categories of input data that have potentially important biases (e.g., based on characteristics listed in item 5).	Methods section; SI section 5.2
For data inputs that contribute to the analysis but were not synthesized as part of the study:		
7	Describe and give sources for any other data inputs.	SI sections 3.1, 3.3, 3.4; Supplementary Table 5
For all data inputs:		
8	Provide all data inputs in a file format from which data can be efficiently extracted (e.g., a spreadsheet rather than a PDF), including all relevant meta-data listed in item 5. For any data inputs that cannot be shared because of ethical or legal reasons, such as third-party ownership, provide a contact name or the name of the institution that retains the right to the data.	Available through http://ghdx.healthdata.org/ihme-data/africa-hiv-prevalence-geospatial-estimates-2000-2017

**Data analysis**		
9	Provide a conceptual overview of the data analysis method. A diagram may be helpful.	Methods section, Extended data Figure 5
10	Provide a detailed description of all steps of the analysis, including mathematical formulae. This description should cover, as relevant, data cleaning, data pre-processing, data adjustments and weighting of data sources, and mathematical or statistical model(s).	Methods section, SI sections 2-4
11	Describe how candidate models were evaluated and how the final model(s) were selected.	SI section 4.3
12	Provide the results of an evaluation of model performance, if done, as well as the results of any relevant sensitivity analysis.	SI section 4.3
13	Describe methods for calculating uncertainty of the estimates. State which sources of uncertainty were, and were not, accounted for in the uncertainty analysis.	Methods section; SI sections 2.3, 4.2, 4.4
14	State how analytic or statistical source code used to generate estimates can be accessed.	Methods section
**Results and Discussion**		
15	Provide published estimates in a file format from which data can be efficiently extracted.	Available through http://ghdx.healthdata.org/ihme-data/africa-hiv-prevalence-geospatial-estimates-2000-2017
16	Report a quantitative measure of the uncertainty of the estimates (e.g. uncertainty intervals).	Results section; Extended data figure 4; Supplementary figures 1-4
17	Interpret results in light of existing evidence. If updating a previous set of estimates, describe the reasons for changes in estimates.	Precision public health and HIV section
18	Discuss limitations of the estimates. Include a discussion of any modelling assumptions or data limitations that affect interpretation of the estimates.	Methods section; SI section 5.2

2 HIV data sources and data processing

2.1 Seroprevalence surveys

2.1.1 Data identification strategy

We identified HIV seroprevalence surveys in sub-Saharan Africa through a review of all surveys in the Demographic and Health Survey (DHS), AIDS Indicator Survey (AIS), and Multiple Indicator Cluster Survey (MICS) series; surveys listed in the Global Health Data Exchange[1]; surveys included in the national HIV estimates files from UNAIDS[2]; and surveys listed in the US Census Bureau HIV/AIDS Surveillance Database[3]. For a survey to be considered for this analysis, we required that the survey reported HIV blood test results, sampled from the general adult population, and contained geographic information more refined than country-level. For surveys with no microdata available we used reports if they included sample size or uncertainty intervals. Our desired age range was 15-49 years, but we also included survey reports that recorded prevalence for an expanded or overlapping age range. The surveys used in this analysis are listed in Supplementary Table 2 and visualized in Extended Data Figures 1 and 2.

2.1.2 Data processing for microdata

To prepare survey microdata for analysis, we first subset the data to the age range of interest, 15-49 years. We then dropped rows for individuals explicitly listed as not tested or where the blood samples were marked as lost or rejected (insufficient sample volume, tip broken, etc.). Inconclusive and indeterminate test results were coded as a negative test result. After subsetting on these conditions, a further 0.07% of the microdata were missing an HIV test result or a survey weight and were dropped from the analysis. A further 0.93% of the microdata were dropped due to missing geographic information or due to the GPS coordinates being located more than 10 km outside of the country border; coordinates within 10 km of the country border were snapped to be approximately 1 km inside the nearest border of the specified country.

We then aggregated the individual-level microdata to calculate HIV prevalence at the finest possible spatial resolution available, ideally a latitude and longitude pair representing the location of the survey cluster (point-level data). The interview date for each specific location was calculated as the median of the individual-level interview dates. Where point-level referencing was not available, we geolocated survey microdata to the smallest geographical area (polygon) possible. Individual-level sample weights were used when calculating prevalence, and the effective sample size for each prevalence estimate was estimated via the Kish approximation[4], which accounts for differences in the underlying selection probability within a sample.

2.1.3 Data processing for reports

In instances where individual-level microdata were not available, we used summary reports, given that the estimates reported were similar in nature to what we would calculate from the microdata. We used the median months of the reported data collection periods (ranging from 2 to 13 months in length) as the interview dates to align with the extracted microdata. If the sample sizes were not included in the report, we estimated the sample sizes from the reported confidence intervals, assuming that a Normal approximation was used to generate 95% confidence intervals. In both instances, sample sizes were further adjusted by multiplying the median design effect (ratio of effective sample size to observed sample size) calculated in the microdata as described above. For prevalence estimates provided for an age range other than 15-49 years, we cross-walked the prevalence to estimate prevalence for the 15-49 age range (see section 2.3 for details).

2.2 Literature review

2.2.1 Search strategy and exclusion criteria

We searched for literature reporting HIV prevalence based on seroprevalence surveys in Africa using the following search string in PubMed on February 14, 2018:

> *("hiv"[Title] OR "hiv1"[Title] OR "hiv2"[Title] OR "hiv-1"[Title] OR "hiv-2"[Title] OR "human immunodeficiency virus"[Title]) AND ("prevalence"[Title/Abstract] OR "seroprevalence"[Title/Abstract] OR "sero-prevalence"[Title/Abstract]) AND ("survey"[Title/Abstract] OR "surveys"[Title/Abstract] OR "cross-sectional"[Title/Abstract] OR "cohort"[Title/Abstract]) AND ("English"[LA]) AND ("Africa"[Title/Abstract] OR "African"[Title/Abstract] OR "Algeria"[Title/Abstract] OR "Angola"[Title/Abstract] OR "Benin"[Title/Abstract] OR "Botswana"[Title/Abstract] OR "Burkina Faso"[Title/Abstract] OR "Burundi"[Title/Abstract] OR "Cameroon"[Title/Abstract] OR "Cape Verde"[Title/Abstract] OR "Central African Republic"[Title/Abstract] OR "Chad"[Title/Abstract] OR "Comoros"[Title/Abstract] OR "Democratic Republic of the Congo"[Title/Abstract] OR "Republic of the Congo"[Title/Abstract] OR "Congo"[Title/Abstract] OR "Cote d'Ivoire"[Title/Abstract] OR "Djibouti"[Title/Abstract] OR "Egypt"[Title/Abstract] OR "Equatorial Guinea"[Title/Abstract] OR "Eritrea"[Title/Abstract] OR "Ethiopia"[Title/Abstract] OR "Gabon"[Title/Abstract] OR "Gambia"[Title/Abstract] OR "Ghana"[Title/Abstract] OR "Guinea"[Title/Abstract] OR "Guinea-Bissau"[Title/Abstract] OR "Kenya"[Title/Abstract] OR "Lesotho"[Title/Abstract] OR "Liberia"[Title/Abstract] OR "Libya"[Title/Abstract] OR "Madagascar"[Title/Abstract] OR "Malawi"[Title/Abstract] OR "Mali"[Title/Abstract] OR "Mauritania"[Title/Abstract] OR "Mauritius"[Title/Abstract] OR "Morocco"[Title/Abstract] OR "Mozambique"[Title/Abstract] OR "Namibia"[Title/Abstract] OR "Niger"[Title/Abstract] OR "Nigeria"[Title/Abstract] OR "Rwanda"[Title/Abstract] OR "Sao Tome and Principe"[Title/Abstract] OR "Senegal"[Title/Abstract] OR "Seychelles"[Title/Abstract] OR "Sierra Leone"[Title/Abstract] OR "Somalia"[Title/Abstract] OR "South Africa"[Title/Abstract] OR "South Sudan"[Title/Abstract] OR "Sudan"[Title/Abstract] OR "Swaziland"[Title/Abstract] OR "Tanzania"[Title/Abstract] OR "Togo"[Title/Abstract] OR "Tunisia"[Title/Abstract] OR "Uganda"[Title/Abstract] OR "Zambia"[Title/Abstract] OR "Zimbabwe"[Title/Abstract]) NOT ("adolescents"[Title] OR "adolescent"[Title] OR "infant"[Title] OR "infants"[Title] OR "child"[Title] OR "children"[Title] OR "patients"[Title] OR "hospitalized"[Title] OR "blood donors" OR "pregnant"[Title] OR "sex worker"[Title] OR "sex workers"[Title] OR "migrant"[Title] OR "migrants"[Title] OR "prison"[Title] OR "prisoners"[Title] OR "men who have sex with men"[Title] OR "MSM"[Title] OR "African-American"[Title/Abstract]) .*

There were 1,846 results from this search. We then completed a title/abstract review where articles were excluded that met the following criteria:

- Used a data source for which we already had microdata;
- Location outside of Africa;
- No measurement of HIV prevalence;
- Facility- or school-based studies;
- Special populations (migrants, workers, blood donors, patients, pregnant women, men who have sex with men, female sex workers, refugees, etc.).

668 articles remained after we excluded the articles that met our initial exclusion criteria. We then performed a full text review, in which we added these additional exclusion criteria:

- Only national-level results;
- Data collected prior to 2000;
- Sample size and uncertainty intervals not included;
- Convenience sampling or other non-probability type sampling;
- Impossible to geolocate due to lack of location information;
- Articles that had duplicate information from the same cohort study.

30 articles remained after the full text review. One additional article that was not captured by our PubMed search but was identified by other means was also included, for a total of 31 articles that were ultimately extracted.

2.2.2. Data processing

For all 31 articles, we extracted information on location, sex, age range, year, sample size, HIV prevalence, HIV type (HIV-1, HIV-2, dual-infections, HIV and combined), response rate, case definition (type of blood test used), case diagnostics (sampling methods and exclusion criteria), and any additional notes deemed relevant.

After extracting the data from each article, we geolocated each observation to a specific latitude and longitude or to a polygon as follows:

1. If the location information provided described an administrative subdivision at the first, second, third, or fourth level, we geolocated these data to a polygon corresponding to this area.
2. If the location information provided described an administrative area below the fourth level, we searched for the location on Google Maps and OpenStreetMaps and measured the area of the location: if the area was less than 5 km in diameter, we geolocated it to the central GPS coordinate of the area; if the area was greater than 5 km in diameter, we created a polygon shape file using QGIS based on the shape in Google Maps or OpenStreetMaps.
3. If the data extracted were from a demographic surveillance site or other cohort, we attempted to locate a map of the study site (these were provided in the article in some instances; in other instances, we did additional internet searches to locate this information) and then used ArcGIS to create a shape file to accurately represent the area.

2.3 Age cross-walk

In some instances, HIV prevalence estimates extracted from literature or survey reports referred to age ranges other than the standard 15-49 age range, which we denote below as A_1-A_2 where $A_1 \neq 15$ and/or $A_2 \neq 49$. In these instances, we used linear regression to transform the estimates of HIV prevalence from the reported age range A_1-A_2 to HIV prevalence for the standard 15-49 age range.

To do this, for each A_1-A_2 we first identified surveys with microdata available that spanned both the A_1-A_2 and 15-49 age ranges, i.e., $\mathrm{minimum}(A_1, 15)$-$\mathrm{maximum}(A_2, 49)$, and produced HIV prevalence estimates for both age ranges by first-level administrative subdivisions. Then for each non-standard age range we used these data to fit the following linear regression model:

$$\mathrm{logit}(p_{15-49}) = \beta_0 + \beta_1 \cdot \mathrm{logit}\left(p_{A_1-A_2}\right) + \epsilon$$

where:

- p_{15-49} is the observed HIV prevalence among individuals age 15-49 years;
- $p_{A_1-A_2}$ is the observed HIV prevalence among individuals age A_1-A_2 years;
- β_0 is an intercept;
- β_1 is the slope on $\text{logit}(p_{A_1-A_2})$;
- ϵ is a normally distributed error term with mean 0.

In this model, a logit transform was applied to prevalence to ensure the predicted prevalence was restricted to between 0 and 1. For values that were equal to 0 and 1, a transformation was employed to preserve the shape of the logistic curve. For these values, an ε was selected to be half of the smallest non-zero value, and all instances of 0 were replaced with ε and instances of 1 replaced with $1 - \varepsilon$. Linear regression was chosen over more complex algorithms due to the high proportion of variance explained with this relatively simple method (R^2 ranged from 0.84 to 0.99).

Uncertainty in prevalence for ages 15-49 as a result of this cross-walk was estimated by simulating 1,000 draws of the slope, intercept, and error term from the fitted linear model, and using these to construct 1,000 draws of HIV prevalence among ages 15-49[5]. Then, for each draw of HIV prevalence, we simulated a random draw from a binomial distribution with *p* equal to prevalence from this draw and *N* equal to the reported sample size and then divided this simulated count by the reported sample size to again calculate HIV prevalence. These draws of HIV prevalence reflected both the sampling uncertainty as well as the additional uncertainty introduced by cross-walking from non-standard age ranges to 15-49. Finally, a new effective sample size was calculated by finding the variance of the prevalence draws and employing the relationship between variance and sample size of a binomial distribution. This new sample size reflected our confidence in the estimate of HIV prevalence for ages 15-49 and is a function of the uncertainty in our linear model, the original sample size, and level of HIV prevalence.

Surveys where this age cross-walk was required are listed in Supplementary Table 3.

2.4 Antenatal care (ANC) sentinel surveillance

2.4.1 Data sources

In addition to general population surveys, we used antenatal care sentinel surveillance data, which measure HIV prevalence among pregnant women attending antenatal care clinics. Most of these data came from national Spectrum files that were developed by a country team of experts and compiled and shared by the UNAIDS secretariat[2]. These files include the HIV prevalence and sample size of ANC sentinel surveillance and routine testing for various sites and years. We only used the sentinel surveillance estimates for our analysis.

We supplemented this data with ANC sentinel surveillance country reports. In general, the reports contained the same information as the Spectrum files, but there was some additional information in the reports and some discrepancies compared to the Spectrum files. The additional information included additional sites, additional years for given sites, and more precise prevalence estimates. In instances where there were discrepancies for a given site-year, we elected to use the source where HIV prevalence was closest to the average prevalence of surrounding years for the same site.

Five countries had a notably large number of discrepancies between the Spectrum files and the ANC reports. The Senegal Spectrum files recorded only HIV-1 positive results so we used the reports to extract

both HIV-1 and HIV-2 positive results to correspond with other countries' estimates. The Zambia Spectrum files recorded prevalence for the 15-39 age range, while the reports recorded prevalence for the 15-44 age range. In this case, we elected to use the Spectrum files because they had better data coverage in terms of number of site-years. There were also many discrepancies in Central African Republic, Côte d'Ivoire, and Zimbabwe; we were unable to identify a specific reason for these discrepancies and elected to use data from the Spectrum files only for these countries.

We investigated the ANC reports to determine if site names in the Spectrum files represented hospitals, cities, or administrative subdivisions. We then used various mapping websites to find geographic information related to these sites. For hospitals and cities/towns that are less than 25 km^2 in area, we used a central GPS coordinate, and for administrative subdivisions we used a polygon of the area. Some hospital sites had a city or town name rather than a hospital name. In those instances, we searched for a hospital in the given city or town and used that hospital's GPS coordinates. If there were multiple hospitals in the area but they were less than 5 km apart, we used the GPS coordinates of the midpoint of the hospitals. If no hospitals were found in the area but the corresponding region was less than 25 km^2 in area, we used the central GPS coordinate. 5.2% of the sites could not be geolocated because none of these conditions were met.

The ANC data included in this analysis are listed in Supplementary Table 4 and visualized in Extended Data Figures 1 and 3.

2.4.2 Data processing

To prepare the ANC data for analysis, we compiled the HIV prevalence and sample size data from the Spectrum files and the ANC reports, and the site geographic information – either GPS coordinates or polygons for administrative subdivisions – into one dataset. After thoroughly inspecting the data, we decided to exclude the following data from our analysis:

- Hospital-level sites were dropped from Congo in 2011 (23 site-years) and Guinea-Bissau in 2003, 2005, 2010, 2014 (10 site-years) because the data aggregated by administrative subdivisions had better temporal coverage.
- We dropped administrative subdivisions that were masked by a different level of administrative subdivisions (8 site-years), defaulting to the level that would give better temporal coverage.
- We determined that 4 site-years were outliers based on inspection of site-level time trends, and these were dropped from the analysis.
- Data from sites that could not be geolocated were also dropped (245 site-years).
- Additionally, in the Spectrum files, data from 12 site-years labeled as sentinel surveillance we suspect are actually routine testing, and we excluded these from the analysis.

2.5 Polygon resampling

Wherever possible, HIV prevalence data were matched to a specific latitude and longitude. In instances where this was not possible, we matched data to the smallest areal unit (termed a polygon) possible. In most instances, these polygons represented administrative subdivisions. The statistical model we employed requires point-referenced data, so data matched to polygons were resampled to generate pseudo-point data based on the underlying population distribution within the polygon. The methods for the resampling are consistent with those previously used in geospatial modelling of under-5 mortality[6].

Specifically, for each polygon-level observation, we randomly sampled 10,000 locations among grid cells in the given polygon with probability proportional to grid cell population. Grid cells were defined to be contained within the polygon if their centroid fell within the geographic boundary. We performed k-means clustering (with k set to 1 per 40 grid cells) on the sampled points to generate a reduced set of locations to be used in modelling based on the k-means cluster centroids. Weights were assigned to each pseudo-point proportional to the number of sampled points contained in each of the k-means clusters, i.e., the number of sampled points divided by 10,000. Each pseudo-point generated by this process was assigned the HIV prevalence observed for the polygon as a whole, and a sample size equal to the sample size for the polygon as a whole multiplied by the weight derived for each point.

3 Covariate and auxiliary data

3.1 Pre-existing covariates

This analysis included five pre-existing covariates: travel time to the nearest settlement of more than 50,000 inhabitants, total population, night-time lights, urbanicity, and malaria incidence. These variables were selected from among available gridded datasets for sub-Saharan Africa because they are factors or proxies for factors previous literature has identified to be associated (not necessarily causally) with HIV prevalence. The first four variables were included as measures or proxies for connectedness and urbanicity as HIV historically spread through sub-Saharan Africa along travel routes[7,8] and is typically found to be higher in more urban compared to more rural locations. Malaria incidence was selected based on prior evidence relating higher malaria incidence rates to higher prevalence of HIV at the population level[9,10]. Sources for these data are given in Supplementary Table 5. These covariates underwent spatial and temporal processing in preparation for their inclusion in analysis.

Spatial processing involved resampling the input covariate raster to align the spatial resolution of the covariate to the 5 x 5-km resolution used in modelling. For covariates that were originally at a finer resolution, we resampled the raster by taking the neighborhood average (travel time to the nearest settlement of more than 50,000 inhabitants, night-time lights, and urbanicity) or sum (total population) of the finer covariate raster to produce one at a 5 x 5-km resolution. Malaria incidence was natively at a 5 x 5-km resolution and thus did not require additional spatial processing.

Temporal processing was required in instances where the original temporal resolution of the covariate was anything other than annual. To resolve from a coarser time period to an annual time period, we filled the intervening years with the value from the nearest neighboring year (urbanicity) or using an exponential growth rate model (total population). Night-time lights and malaria incidence were provided at a one-year temporal resolution and did not require interpolation. As travel time to the nearest settlement of more than 50,000 inhabitants was available only for a single representative year (2015), this covariate was set to be unchanged over time. After interpolation, night-time lights and urbanicity were still missing the most recent years of the 2000–2017 analysis period, and in these instances we filled out the end of the time-series carrying forward the most recent year without modification.

3.2 Covariates constructed for this analysis

3.2.1 Covariate selection criteria and definitions

In addition to the five pre-existing covariates, additional covariates were constructed specifically for this analysis. Numerous studies have been conducted in sub-Saharan Africa on risk and protective factors for HIV infection, and these factors commonly include sexual behaviour and factors that are thought to

influence the transmission of HIV during sexual intercourse[11]. Potential covariates were informed by past literature and required to have a demonstrated association with HIV prevalence, though not necessarily a causal relationship. Furthermore, our selection of covariates depended on having adequate data coverage from data sources that could be readily extracted. In total, eight covariates were constructed:

- Prevalence of male circumcision, including medical or traditional circumcision ('male circumcision');
- Prevalence of self-reported STI symptoms (genital discharge and/or genital ulcer/sore) in the last 12 months ('STI symptoms');
- Prevalence of marriage or living with a partner as married ('in union');
- Prevalence of one's current partner living elsewhere ('partner away');
- Prevalence of condom use at last sexual encounter within the last 12 months ('condom last time');
- Prevalence of reporting ever had sexual intercourse among young adults ('had intercourse');
- Prevalence of men reporting multiple sexual partners within the last year ('multiple partners in year');
- Prevalence of women reporting multiple sexual partners within the last year ('multiple partners in year').

The notion that male circumcision has a protective effect against acquiring HIV was first proposed in 1986, and since then more than 30 cross-sectional studies have found the prevalence of HIV to be significantly higher in uncircumcised men as well as numerous prospective studies that have shown a protective effect ranging from 48% to 88%[12,13]. In 2005, following the interruption of a randomized controlled trial of male circumcision in South Africa that showed a 60% protective effect of circumcision, WHO and UN agencies first acknowledged evidence of male circumcision's protective effect[14,15]. Following these declarations, voluntary medical male circumcision clinics (VMMC) emerged as an HIV prevention strategy in 14 countries in Eastern and Southern Africa with high HIV prevalence and low levels of male circumcision[16]. Given male circumcision's linkage to HIV in the scientific literature, many surveys record men's self-reported circumcision status.

Coinfection of HIV with viral and bacterial sexually transmitted infections (STIs), most notably herpes simplex virus type 2, is a well-studied mechanistic factor associated with higher risk of HIV acquisition[17]. STIs are thought to have been especially important risk factors during the early stages of the epidemic when infections were concentrated in high-risk groups, though researchers have since argued STIs are also critical in advanced stages[18]. Due to the association between STI prevalence, sexual behaviour, and HIV, most survey series detail the self-reported presence of STI symptoms, facilitating its inclusion as an HIV covariate in this analysis.

Marital status represents a structural factor that, while distal to HIV exposure, has been associated with the number and type of sexual partners, as well as with HIV status[19,20]. It has been postulated that the relationship between an individual's marital status and the number of sexual relationships regulates the protective effect of marriage on the risk of HIV infection, especially in older populations[21]. Marital status is a readily available indicator in household surveys more generally.

The frequency with which a partner has slept away from home during the past year is an indicator of the mobility of male partners, and studies have found that mobility confers an increased risk for HIV[22]. Part of

the rapid spread of HIV in sub-Saharan Africa has been attributed to occupations that consist of geographical mobility, especially truck drivers, who are identified as high risk for acquiring and spreading HIV[23]. Many surveys ask women if their partner has lived away from home in the past year, and we use these responses as a proxy for occupational mobility.

Condom use is a sexual behaviour factor that is protective against acquiring HIV. Condoms are often presented as the most effective HIV prevention method of sexual transmission of the disease[24]. Though difficult to measure accurately how often condoms are used in sexual encounters, most surveys report on the use of condoms in last sexual intercourse, a readily available proxy for overall condom use.

An early age at sexual debut may be associated with the number of lifetime sexual partners, which is considered a key risk factor for contracting HIV[21]. Furthermore, early age at sexual debut has been shown to be associated with numerous other risk factors for HIV acquisition, such as STI prevalence, decreased condom use, and increased number of sexual partners[25]. For young women, the initiation of sexual activity is the first important determinant of potential viral exposure, and delayed sexual debut has been associated with decreased risk of HIV acquisition[26]. Given these relationships between HIV and age of sexual debut, and the relative ease of acquiring self-reported sexual status, we constructed an indicator for whether young (ages 15-24) men and women have had intercourse.

An individual's number of sexual partners correlates with HIV risk, and past studies have found an increasing linear relationship between the number of sexual partners and HIV prevalence[27]. The number of sexual partners is thought to have been an especially important factor in the early stages of an epidemic, though past research has determined it remains a key risk factor in advanced stages[18]. Surveys often ask men and women their number of partners in the past year, and we used these responses to construct a proxy for multiple concurrent sexual relationships. Separate covariates were constructed for men and women given the well-documented discrepancy in the number of partners reported by men as compared to women[28].

3.2.2 Covariate data

3.2.2.1 Covariate data identification strategy

We reviewed major survey series (Demographic and Health Surveys [DHS]; Multiple Indicator Cluster Surveys [MICS]; AIDS Indicator Surveys [AIS]; Malaria Indicator Surveys [MIS]; Performance, Monitoring, and Accountability Surveys [PMA]; Reproductive Health Surveys [RHS]; and Living Standards Measurement Surveys [LSMS]) to identify surveys in sub-Saharan Africa that contained relevant variables. We supplemented this initial list of surveys with country-specific surveys identified in the Global Health Data Exchange[1] and with a cross-check of all surveys extracted for HIV prevalence. In order for a survey to be included, we required that it include variables related to one or more of the covariate indicators (including any time restrictions inherent to the indicator definition) and to contain geographic information at a subnational level.

For all indicators except for 'had intercourse,' we required a survey to sample the general adult (ages 15-49) population. For 'had intercourse', a survey only had to sample the general young adult (ages 15-24) population to be included.

Because of variations we identified in the way these questions were asked across surveys, we tracked the skip logic and question format for all surveys including STI symptoms and/or the sexual activity indicators. This helped us identify surveys for which the question format was so substantively different from others

as to require special handling or exclusion (e.g., questions asked without a time restriction for indicators that require a response from the last 12 months). We excluded select surveys because of these irreconcilable question variations, incomplete sampling (e.g., a specific age range or subpopulation), or untrustworthy or outlier data (as determined by the survey administrator or by inspection). The surveys used for these covariates are listed in Supplementary Table 6, and surveys identified as outliers and subsequently excluded from this analysis are listed in Supplementary Table 7.

3.2.2.2 Covariate data processing for microdata

To prepare the survey microdata for analysis, we first constructed final indicators from the raw variables included in the survey data:

- For 'STI symptoms,' we constructed a symptoms indicator that was true if a respondent reported either genital discharge or a genital sore/ulcer in the last 12 months, missing if either individual symptom was missing, and false if both symptoms were reported in the negative.
- For 'in union,' we constructed an indicator that was true for all respondents who reported being either currently married or living with a partner, false for any other marital status response, and missing if the marital status response was missing.
- For 'multiple partners in year', we used the reported number of sexual partners within the last 12 months to construct a binary indicator that was true for any respondent reporting two or more partners and false for any respondent with 0 or 1 partners (including respondents who had never had intercourse).
- The other indicators were extracted from the survey microdata in their final form and required no additional construction.

For each indicator, we subset the data to the desired age range (15-24 years for 'had intercourse', 15-49 years for all other indicators). For 'STI symptoms' we additionally restricted the sample to respondents who reported having had intercourse, while for 'partner away' we additionally restricted the sample to respondents currently 'in union'. We dropped any rows with missing responses or sample weights. For indicators where we model men and women together ('STI symptoms,' 'in union,' 'condom last time'), we dropped any surveys that did not interview both men and women. Any observations missing geographic information or with inconsistent geographic information (i.e., points more than 10 km from the nearest specified country border) were also dropped.

Finally, we aggregated the weighted individual-level microdata for each indicator to the finest possible spatial resolution available, in the same way as was done for the HIV prevalence data. Data were geolocated to latitude and longitude at the survey cluster level wherever possible, and to the smallest possible polygon available otherwise. As with the HIV prevalence data, we calculated the effective sample size for each spatial aggregation using the Kish approximation[4].

For three of the sexual activity indicators ('condom last time', 'multiple partners in past year', and 'had intercourse'), we initially prepared the data separately for men, women, and both sexes combined to evaluate any sex-specific patterns or trends. To investigate the sex-specific trends, three models were fit with sex-specific data and both sexes combined. The results were interrogated to see how correlated the indicators were over space and time. For 'condom last time', prevalence reported among men was generally higher than among women, but levels among men and women were highly correlated over time and space. We decided to include both sexes combined for the final model to maximize data availability.

Likewise, for ‘had intercourse,’ the estimates from the men- and women-specific models showed high correlation, but a women-specific model was selected due to existence of a number of surveys that only asked this question of women. In contrast, for ‘multiple partners per year,’ men- and women-specific models showed different trends over space and time, so separate sex-specific covariates were created. This was reinforced by literature that suggested discrepancies between men and women in the reported number of lifetime partners[28].

3.2.2.3 Covariate data processing for reports

For ‘male circumcision,’ we additionally included summary reports for surveys where individual-level microdata were not available. As with HIV prevalence report extractions, we used the median month of data collection as the interview date. We cross-walked the circumcision prevalence from any surveys with an age range other than 15-49 to estimate the prevalence for that age range in alignment with the microdata, again using the same methods as for HIV prevalence. Surveys where this age cross-walk was required are listed in Supplementary Table 3.

We chose not to include summary reports for other covariates. For ‘STI symptoms,’ the estimates included in reports used a different construction of the variable than that which we built from the microdata, making the reports incompatible with the microdata. For the sexual activity indicators, we decided against summary report extraction due to the significant number of surveys we were able to extract at the microdata level and the scarcity of reports for most of these indicators.

3.2.3 Covariate modelling

Each of these covariates was estimated using the same modelling framework used for HIV prevalence as described in Section 4, with the key exceptions of not including any covariates in the geospatial model, and not including any correction for data derived from ANC sentinel surveillance (which are used only for modelling HIV prevalence). Therefore, these models were fit including only the intercept, Gaussian process term, country random effect, and nugget effect. All priors and hyper-priors were otherwise the same. Maps of each constructed covariate in 2000, 2005, 2010, and 2017 are displayed in Supplementary Figures 9-16.

3.3 Administrative boundaries

For this analysis we used the Global Administrative Units Layers (GAUL) shape files maintained for the Food and Agriculture Organization (FAO) to define country boundaries and first- and second-level administrative subdivisions[29]. For Kenya, GADM shape files were used instead for first- and second-level administrative subdivisions and subsequently manually updated to address known discrepancies[30].

3.4 Gridded population

The gridded population data used for this analysis were obtained from WorldPop[31,32]. Because WorldPop provides data at a 1 x 1-km spatial resolution at five-year intervals, we processed these data as described in Section 3.1 to aggregate to a 5 x 5-km spatial resolution and interpolate to annual time periods. When we use population as a covariate (described in Section 3.1), we use total population. In all other instances (as described in Section 4.3) we use the population for ages 15-49.

4 Statistical model

4.1 Covariate stacking

Stacked generalization/regression, or stacking, is an ensemble modelling method that combines multiple prediction methods to increase predictive validity relative to a single modelling approach. This ensemble modelling method relies on a variety of sub-models that are then combined by a secondary learner to produce a meta-model that fuses multiple algorithmic methods to capture nonlinear effects and complex interactions[33]. Our implementation of stacking largely follows the approach described by Bhatt and colleagues[34] and previously implemented for mapping under-5 mortality, child growth failure, educational attainment, and diarrheal diseases in Africa[6,35–37].

We fit three sub-models – a generalized additive model, boosted regression trees, and lasso regression – to the HIV survey data described above with the five pre-existing and eight constructed covariates as well as calendar year included as explanatory variables. ANC data were excluded from the sub-models because of known biases (described below). We selected these three sub-models based on ease of implementation through existing software packages, the fundamental differences in their approaches, and a proven track record of predictive accuracy[34]. Sub-models were fit in R using the mgcv, xgboost, glmnet, and caret packages.

Each sub-model was fit using five-fold cross-validation to avoid overfitting, and hyper-parameter fitting was done to maximize predictive power. For each sub-model, we produced two sets of predictions: out-of-sample and in-sample. Out-of-sample predictions for each model were generated by compiling the predictions from the five holdouts from each cross-validation fold, and in-sample predictions were generated by re-fitting the sub-models using all available data. The out-of-sample sub-model predictions were used as explanatory covariates when fitting the geostatistical model described below, and the in-sample predictions were used when generating predictions from the geostatistical model in order to maximize data use. In both instances, the logit-transformation of the predictions was used to put these predictions on the same scale as the linear predictor in the geostatistical model. Maps of in-sample predictions from each stacker are presented in Supplementary Figures 17-19.

4.2 Geostatistical model

4.2.1 Model description

We modelled HIV prevalence using the following spatially and temporally explicit generalized linear mixed effects model:

$$\begin{aligned} Y_{i,t} &\sim \text{Binomial}\left(p_{i,t}, N_{i,t}\right) \\ \text{logit}\left(p_{i,t}\right) &= \beta_0 + \boldsymbol{\beta}_1 \boldsymbol{X}_{i,t} + \gamma_{c[i]} + Z_{i,t} + \epsilon_{i,t} + (\beta_2 + U_i) \cdot I_{ANC} \end{aligned}$$

$$\begin{aligned} \gamma_{c[i]} &\sim \text{Normal}\left(0, \sigma^2_{country}\right) \\ Z_{i,t} &\sim \text{GP}(0, \Sigma_{space} \otimes \Sigma_{time}) \\ \epsilon_{i,t} &\sim \text{Normal}\left(0, \sigma^2_{nugget}\right) \\ U_i &\sim \text{GP}(0, \Sigma_{space}) \end{aligned}$$

where:

- $N_{i,t}$ and $Y_{i,t}$ are the number of individuals sampled and the number of individuals who are HIV+ among those sampled, respectively, in location *i* and year *t*;
- $p_{i,t}$ is the underlying HIV prevalence in location *i* and year *t*;
- β_0 is an intercept;
- $\boldsymbol{X}_{i,t}$ is a vector of logit-transformed stacked covariates for location *i* and year *t,* and $\boldsymbol{\beta}_1$ is the corresponding vector of regression coefficients;
- $\gamma_{c[i]}$ is a country-level random effect for country *c* containing location *i*;
- $Z_{i,t}$ is a spatially and temporally correlated random effect for location *i* and year *t*;
- $\epsilon_{i,t}$ is an independent and identically distributed random effect for location *i* and year *t*;
- I_{ANC} is an indicator variable that is 1 for data derived from antenatal care clinic sentinel surveillance and 0 otherwise;
- β_2 is a fixed offset for data derived from antenatal care clinic sentinel surveillance;
- and U_i is a spatially correlated random offset for data derived from antenatal care clinic sentinel surveillance for location *i*.

Descriptively, this model specifies logit-transformed HIV prevalence as a linear combination of a regional intercept (β_0), covariate effects ($\boldsymbol{\beta}_1\boldsymbol{X}_{i,t}$), country random effects ($\gamma_{c[i]}$), spatially and temporally correlated random effects ($Z_{i,t}$), and an uncorrelated error term or nugget effect ($\epsilon_{i,t}$). The intercept captures the overall mean level of HIV prevalence while the covariate effects capture the spatial and temporal variation in HIV prevalence that can be described as a function of spatial and temporal variation in the included covariates. The country random effects capture additional variation between countries, while the spatially and temporally correlated random effects capture additional variation by location (within and between countries) and time that varies smoothly in space and time. Finally, the uncorrelated error term (or nugget effect) captures any additional, non-structured variation by location and time.

HIV prevalence as measured by sentinel surveillance of antenatal care (ANC) clinics is known to be biased as a measure of HIV prevalence in the general adult population because it captures pregnant women who attend ANC only, as compared to all adult men and women[38,39]. This bias may be either positive or negative: the fact that all pregnant women are sexually active tends to elevate their risk of having acquired HIV prevalence compared to the general adult population (some of whom are not sexually active), while HIV-related sub-fertility tends to reduce the prevalence of HIV-positive women among the population of pregnant women[40,41]. However, ANC data have better temporal and spatial coverage in many countries than survey data alone (Extended Data Figure 1). We incorporated ANC data to capitalize on this additional data coverage, but also attempted to correct for the known biases. In instances where data in our model were derived from ANC sentinel surveillance ($I_{ANC} = 1$), our model allows for this bias via a fixed term (β_2) that captures the overall mean bias, and a spatially varying term (U_i) that captures local differences in the extent of this bias. This approach is conceptually similar to previously described approaches for spatial modelling using non-randomized (and therefore potentially biased) data and randomized survey data[42,43]. Although the bias associated with ANC sentinel surveillance may also vary over time in addition to varying spatially, we felt there was insufficient data to estimate both spatial and temporal variation in this bias, and so the bias associated with ANC sentinel surveillance was assumed to be time-invariant over the period of this analysis.

The spatially and temporally correlated random effect ($Z_{i,t}$) is modelled as a Gaussian process with mean 0 and a covariance matrix given by the Kronecker product of a spatial Matérn covariance function

(Σ_{space}) and a temporal first-order autoregressive (AR1) covariance function (Σ_{time}). U_i is a spatially correlated random effect and is modelled as a Gaussian process with mean 0 and spatial Matérn covariance (Σ_{space}). The Matérn covariance function is given by:

$$\Sigma_{space} = \sigma^2 \frac{2^{1-\nu}}{\Gamma(\nu)} \times (\kappa \boldsymbol{D})^{\nu} \times \mathrm{K}_{\nu}(\kappa \boldsymbol{D})$$

In this analysis ν (the smoothness parameter) was fixed at 1. For both $Z_{i,t}$ and U_i, a penalized complexity (PC) prior was used for the Matérn covariance function and specified via two hyper-parameters: the spatial range, ρ_s (where $\rho_s = \sqrt{8\nu}/\kappa$ and is equal to the distance at which correlation is approximately 0.1; the subscript *s* for space is used as to not confuse with the temporal correlation parameter), and marginal standard deviation, σ. PC priors shrink towards a more simplistic base model – in this case, one where the marginal variance is 0 and the spatial range is infinite – and are specified via setting the tail probabilities on each hyper-parameter[44,45]. We followed the guidance provided by Fugulstad et al., who recommend selecting priors that satisfy $\mathrm{P}(\sigma > \sigma_0) = 0.05$ and $\mathrm{P}(\rho_s < \rho_{s_0}) = 0.05$, where σ_0 is between 2.5 to 40 times the expected true marginal standard deviation and ρ_{s_0} is between $1/10$ to $1/2.5$ of the expected true range[46]. Specifically, we set:

$$\sigma_0 = 2;\ \mathrm{P}(\sigma > \sigma_0) = 0.05$$
$$\rho_{s_0} = 0.1;\ \mathrm{P}(\rho_s < \rho_{s_0}) = 0.05$$

In addition, for $Z_{i,t}$, the AR1 covariance function is associated with a temporal correlation parameter ρ (not be confused with ρ_s). We used the following hyper-prior in this case, which corresponds to a prior mean of 0.76 with a 95% range of -0.17 to 0.97 for ρ:

$$\log((1+\rho)/(1-\rho)) \sim \mathrm{Normal}(2, 1.2^2)$$

PC priors were also used for the standard deviation of $\gamma_{c[i]}$ and $\epsilon_{i,t}$ and were set to:

$$\sigma_{country_0} = 2;\ \mathrm{P}(\sigma_{country} > \sigma_{country_0}) = 0.05$$
$$\sigma_{nugget_0} = 2;\ \mathrm{P}(\sigma_{nugget} > \sigma_{nugget_0}) = 0.05$$

Finally, priors for fixed effects were set as:

$$\beta_0 \sim \mathrm{Normal}(0, 3^2)$$
$$\boldsymbol{\beta}_1 \sim^{iid} \mathrm{Normal}(0, 3^2)$$
$$\beta_2 \sim \mathrm{Normal}(0, 3^2)$$

4.2.2 Prior sensitivity analysis

Sensitivity analyses were undertaken to assess the impact of the hyper-priors for all random effects on the model predictions. In addition to the model described above, we considered five alternate models with different hyper-prior specifications, as outlined below.

- Model 1 (Less informative PC priors)

  In this model, we retained the PC priors for ρ_s and σ and the hyper-parameters for $Z_{i,t}$ and U_i, but made these hyper-priors less informative by specifying a larger value of the standard deviation and smaller value of the spatial range when setting the tail probabilities:

$$\sigma_0 = 4;\ \mathrm{P}(\sigma > \sigma_0) = 0.05$$
$$\rho_{s_0} = 0.05;\ \mathrm{P}(\rho_s < \rho_{s_0}) = 0.05$$

We similarly updated the PC priors for the standard deviation of $\gamma_{c[i]}$ and $\epsilon_{i,t}$:

$$\sigma_{country_0} = 4;\ \mathrm{P}(\sigma_{country} > \sigma_{country_0}) = 0.05$$
$$\sigma_{nugget_0} = 4;\ \mathrm{P}(\sigma_{nugget} > \sigma_{nugget_0}) = 0.05$$

The hyper-prior for the temporal correlation, ρ, was the same as our selected model.

- Model 2 (Less informative PC priors with informative ρ)

  This model has the same less-informative PC priors as Model 1 on the spatial parameters and other random effects, but the hyper-prior for the temporal correlation parameter ρ was adjusted to be more heavily weighted toward a high temporal correlation:

$$\log((1+\rho)/(1-\rho)) \sim \mathrm{Normal}(4, 1.2^2)$$

  This hyper-prior corresponds to a prior mean of 0.96 with a 95% range of 0.68 to 1 for ρ.

- Model 3 (INLA default)

  This model used the default hyper-priors in INLA implemented in the function inla.spde2.matern()[47]. These hyper-priors are defined in terms of τ (where $\tau = \sqrt{1/(4\pi\kappa^2\sigma^2)}$) and κ, and were set as:

$$\theta_1 = \log(\tau) \sim \mathrm{Normal}(\mu_{\theta_1}, \sigma^2_{\theta_1})$$
$$\theta_2 = \log(\kappa) \sim \mathrm{Normal}(\mu_{\theta_2}, \sigma^2_{\theta_2})$$

  with μ_{θ_1}, σ_{θ_1}, μ_{θ_2}, and σ_{θ_2} automatically determined by INLA.

  INLA default hyper-priors were also used for the precision parameter of the remaining random effects:

$$1/\sigma^2_{country} \sim \mathrm{gamma}(\mathrm{rate} = 1, \mathrm{shape} = 0.00005)$$
$$1/\sigma^2_{nugget} \sim \mathrm{gamma}(\mathrm{rate} = 1, \mathrm{shape} = 0.00005)$$

  The hyper-prior for the temporal correlation parameter, ρ, was the same as our selected model.

- Model 4 (INLA default with informative ρ):

  This model had the same hyper-priors for τ, κ, $\sigma^2_{country}$, and σ^2_{nugget} as model 3, but the more informative hyper-prior for the temporal correlation parameter, ρ, described for model 2.

- Model 5 (default PC prior with informative ρ)

  This model used the same PC priors as our selected model but used the more informative hyper-prior for the temporal correlation parameter, ρ, described for model 2.

All six models (five alternative models outlined here and selected PC prior model described in the previous section) were fit for each covariate model and the HIV prevalence model to enable comparison

of the corresponding model predictions. The less-informative PC prior (models 1 or 2) failed to converge in at least one region for three covariates ('partner away', 'had intercourse', and 'condom last time'), which is a known problem in INLA when the prior is insufficiently informative. For these indicators, the model that failed to converge was excluded from the comparison.

The predictions from each of the five alternate models were highly correlated with the predictions from the selected model at the grid cell level and at the first- and second-order administrative subdivision level, with the pairwise correlation above 0.999 in most cases (Supplementary Table 8). Additionally, in all but one case (Model 4 for 'male circumcision' at the first-order administrative subdivision level), there was 100% overlap in the uncertainty intervals between the selected model and any alternate model. The mean absolute difference between predictions was also generally low. These comparisons suggest that the predictions are relatively robust to different hyper-prior specifications.

4.2.3 Model fitting and prediction

This model was fit in R-INLA[48] using the stochastic partial differential equations (SPDE)[49] approach to approximate the continuous spatial and spatial-temporal Gaussian random fields (U_i and $Z_{i,t}$, respectively). We constructed a finite elements mesh for the SPDE approximation to the Gaussian process regression using a simplified polygon boundary (Supplementary Figure 27). We set the inner mesh triangle maximum edge length (the mesh size for areas over land) to be 0.25 decimal degrees, and the buffer maximum edge length (the mesh size for areas over the ocean) to be 5.0 decimal degrees. Estimated model parameters are listed in Supplementary Table 9.

Due to computational constraints and to allow for regional differences in the relationship between covariates and HIV prevalence as well as the strength of spatial and temporal auto-correlation in HIV prevalence, separate models were fit for four regions (Extended Data Figure 7). Specifically, we used the regional classifications for sub-Saharan Africa from the Global Burden of Disease (GBD) study[50] which group countries by location and epidemiological profile. We made one modification to this classification, grouping Sudan as part of the Eastern sub-Saharan Africa region rather than the North Africa and the Middle East region.

After fitting each model, we generated 1,000 draws of all model parameters from the approximated joint posterior distribution using the inla.posterior.sample() function in R-INLA. For each draw *s* of the model parameters we constructed a draw of $p_{i,t}$ as:

$$p_{i,t}^{(s)} = \text{logit}^{-1}\left(\beta_0^{(s)} + \boldsymbol{\beta}_1^{(s)}\boldsymbol{X}_{i,t} + \gamma_{c[i]}^{(s)} + Z_{i,t}^{(s)} + \epsilon_{i,t}^{(s)}\right)$$

I_{ANC} is set to 0 for the purposes of generating estimates, so draws of β_2 and U_i are not incorporated when generating draws of $p_{i,t}$. Additional processing of the output from inla.posterior.sample() is required for the spatial-temporal random effect ($Z_{i,t}^{(s)}$) and the nugget effect ($\epsilon_{i,t}^{(s)}$) prior to constructing $p_{i,t}^{(s)}$ according to the equation above. Specifically, for $Z_{i,t}^{(s)}$, draws are generated initially only at vertices of the finite element mesh, so we project from this mesh to each combination of (i,t) desired for prediction, i.e., the centroid of each grid cell on a 5 × 5-km grid as well as all years from 2000 to 2017. For the nugget effect, we generate $\epsilon_{i,t}^{(s)}$ for each combination of (i,t) by sampling from $\text{Normal}\left(0, \sigma_{nugget}^{2}{}^{(s)}\right)$. At the end of this process, we have 1,000 draws of $p_{i,t}$ for each grid cell and year combination.

4.3 Model validation

4.3.1 Validation strategy

We used five-fold cross-validation in order to assess the performance of the modelling framework described above with respect to predicting HIV prevalence. To do so, we first split all survey data into five groups by randomly sorting a list of unique identifiers for each survey, calculating the cumulative effective sample size represented by the surveys in this list, and then dividing the list into five parts at the point where this cumulative sample size was closest to 20%, 40%, 60%, and 80% of the total. This results in five groups that are approximately equal in terms of the total effective sample size and which contain entire surveys (i.e., all of the data points derived from each survey are contained exclusively within only one fold). We then fit the model described above five times, excluding each of the five groups of data in turn. All ANC data were included in all models and were not used to assess model performance given the known biases in these data.

After fitting the model five times, the data withheld from each model were matched with predictions from that model, and then these data-prediction pairs were compiled across all five models, resulting in a complete dataset of out-of-sample predictions corresponding to all survey data included in the analysis. HIV prevalence estimates based on single survey clusters are generally quite noisy due to very small sample sizes, and are consequently insufficient as a 'gold standard' for evaluating the model predictions[6]. To address this issue, we aggregated both the observed data and the corresponding out-of-sample predictions within countries and within first- and second-level administrative subdivisions, by calculating a weighted mean of each using the effective sample sizes as the weights. Then, across all data-estimate pairs, we calculated two summary measures: the mean error (ME, a measure of bias) and the root-mean-square error (RMSE: a measure of total variance).

In addition, for each data-estimate pair, we constructed 95% prediction intervals from the 2.5th and 97.5th percentiles of 1,000 draws from a binomial distribution corresponding to each of the 1,000 posterior draws of HIV prevalence with p equal to HIV prevalence in a given posterior draw and N equal to the effective sample size for the data point. We then calculated coverage as the percentage of data-estimate pairs where the data point was contained within this 95% prediction interval.

Finally, to complement the out-of-sample predictive validity metrics, we also calculated in-sample predictive validity metrics using the same process but matching each data point to predictions from a model fit using all data.

4.3.2 Sensitivity analyses

We used this validation strategy to assess model performance of the final model (as described in sections 4.1-4.2) and to compare to a number of alternatives, considering in particular the impact of the choices and assumptions we make with regards to incorporating covariates, using data from ANC data, and integrating data with polygon rather than point location information[51].

First, we assessed the contribution of the covariates and the stacking algorithm to the overall modelling strategy by considering models with no covariates and models with covariates included in the geostatistical model as-is ('raw') rather than first going through the stacking algorithm. We additionally considered models with and without the Gaussian process (GP) spatial-temporal smoothing term ($Z_{i,t}$). This leads to five models:

1. No covariates, with GP;

2. Raw covariates, no GP;
3. Raw covariates, with GP;
4. Stacked covariates, no GP;
5. Stacked covariates, with GP (this is the full model described in Sections 4.1-4.2)

The summary error measures for these five models are shown in Supplementary Figure 20 and a comparison of the estimates derived from these models is shown in Supplementary Figure 21. Across all three levels of aggregation and all three validation metrics, the models with a Gaussian process outperformed those without, with mean error closer to zero, smaller RMSE, and coverage closer to 95%. Among the three models with a Gaussian process, performance was more similar, although the mean error for models with covariates (either raw or stacked) was noticeably closer to zero than models without covariates, both in- and out-of-sample. For all models with a Gaussian process, coverage of the prediction intervals was between 96% and 97% in-sample, and between 90% and 93% out-of-sample.

Second, to assess the contribution of including ANC data in addition to survey data as well as several alternate approaches to including these data, we considered models with and without the ANC data included, and three different approaches to the bias-correction for these models: no correction, U_i as a non-structured (independently and identically distributed (IID)) random effect, and U_i as a spatially structured random effect. This leads to four models:

1. No ANC data (survey data only);
2. ANC data with no bias correction;
3. ANC data with IID correction;
4. ANC data with GP correction (this is the full model described in Sections 4.1-4.2)

The summary error measures for these four models are shown in Supplementary Figure 22, and a comparison of the estimates derived from these models is shown in Supplementary Figure 23. The model including ANC data but no bias correction was consistently the worst in terms of mean error and RMSE both in- and out-of-sample. The models including ANC data with some type of bias correction outperformed the model with no ANC data, with a noticeably lower out-of-sample RMSE. Comparing the two bias corrections, the model with the Gaussian process correction was slightly superior to the model with the IID correction in terms of out-of-sample mean error (closer to zero), and out-of-sample coverage (closer to 95%), although the RMSE both in- and out-of-sample was comparable between these two models.

Third, to assess the sensitivity of the model to including polygon data processed via polygon resampling in addition to point data, we considered models with and without polygon data included. In both cases, we assessed the performance of the model in terms of in-sample and out-of-sample predictions for the point data only. The summary error measures for these two models are shown in Supplementary Figure 24, and a comparison of the estimates derived from these models is shown in Supplementary Figure 25. In-sample RMSE was relatively constant between the two models, while in-sample mean error was slightly larger in the model with point and polygon data compared to the model with point data only. Out-of-sample, however, the model with both point and polygon data performed noticeably better – smaller mean error and lower RMSE – than the model with point data only.

4.4 Post-estimation

4.4.1 Calibration to Global Burden of Disease 2017

To take advantage of the more epidemiologically structured modelling approach and additional national-level data used by GBD 2017, we performed post hoc calibration of our estimates to the GBD estimates[52]. As a preliminary step to this calibration and the aggregation described in the next section, we first intersected each grid cell with the second-level administrative subdivision shape file to determine what fraction of the area of each grid cell fell within each administrative unit. Since all second-level subdivisions nest within first-level subdivisions, which in turn nest within countries, this strategy assigned the cell fractions to an administrative area at each level of the administrative hierarchy. We assumed that population density within each cell was uniform, and for cells that were split across multiple subdivisions, allocated the WorldPop population estimate in proportion to area. This process was carried out separately for each modelling region, so cells that cross international borders that are also regional borders were allocated in their entirety to the country that contained the centroid of the grid cell.

Using this assignment of cells and cell fractions to the administrative hierarchy, we first scaled the grid-cell-level WorldPop estimates for the 15-49 age group to match the corresponding GBD population estimates for each country and year[53]. To do so, for each country and year, we defined a population raking factor as the ratio of the GBD population estimate to the sum of the WorldPop population estimates for all cells and fractional cells within the country, and then multiplied the WorldPop population estimates for all cells and fractional cells within the country by this raking factor.

We then similarly adjusted our HIV prevalence estimates. Specifically, for each country and year, we defined a prevalence raking factor as the ratio of the GBD prevalence estimate to the population-weighted mean of estimates for all cells and fractional cells within the country, and then multiplied each HIV prevalence draw for all cells and fractional cells within the country by this raking factor. At this point, the prevalence estimates for cells that had been fractionally allocated to multiple countries were recombined by calculating a weighted average with weights determined by the relative area of each fraction. Final point estimates for each grid cell were calculated as the mean of the scaled draws, and 95% uncertainty intervals were calculated as the 2.5th and 97.5th percentiles of the scaled draws. The impact of this calibration procedure is depicted in Supplementary Figure 26, which compares the pre-calibration estimates to the post-calibration estimates, both aggregated at the national level. The corresponding prevalence raking factors had a mean of 1.09 and an interquartile range of 0.88–1.14.

4.4.2 Aggregation to first- and second-level administrative subdivisions

In addition to estimates of HIV prevalence on a grid, we also constructed estimates of HIV prevalence for first- and second-level administrative subdivisions. These estimates were derived by calculating population-weighted averages of HIV prevalence for each grid cell or fractional grid cell within a given first- or second-level administrative subdivision. Grid cell fractions were assigned through intersection as described above, and this process used the calibrated population estimates and prevalence draws also described above. This was carried out for each of the 1,000 posterior draws at the grid cell level, generating 1,000 posterior draws for each administrative subdivision. Final point estimates and uncertainty intervals for each subdivision at each level of the administrative hierarchy were derived from the mean, 2.5th percentile, and 97.5th percentile of these draws, respectively.

4.4.3 Calculating people living with HIV (PLHIV)

We estimated the number of people living with HIV (PLHIV) in each grid cell and year by combining estimated population and HIV prevalence after calibration to GBD as described above. Specifically, for each cell and fractional cell, we multiplied the estimated population by each of the 1,000 prevalence draws to generate 1,000 draws of PLHIV. Fractional cells were then recombined by summing PLHIV for each draw within each cell. Final point estimates and uncertainty intervals for PLHIV were calculated as the mean, 2.5th percentile, and 97.5th percentile of these draws, respectively.

5 Supplementary Discussion

5.1 Advances compared to previous analyses

This study is not the first to consider subnational variation in HIV prevalence (Supplementary Table 1). However, this analysis builds upon and expands beyond previously published analyses in several important ways.

First, this is, to our knowledge, the only study to date to investigate subnational variation in HIV prevalence with a high degree of spatial granularity (second-level administrative subdivisions or lower) on a continental scale. This enables multinational comparisons and exploration of cross-border trends, but more importantly ensures that estimates are available for all countries in sub-Saharan Africa, including those that may have previously been missed entirely or analyzed only at a coarser subnational level (e.g., first-level administrative subdivisions).

Second, this analysis makes use of a wider array of data sources than are typically considered, with the aim of producing more robust and precise estimates. Most previous analyses used a single data source per country, whereas this analysis incorporated all identified and available data from seroprevalence surveys and ANC surveillance in each country and incorporated additional data in the form of covariates. Additionally, by calibrating our estimates to those from the GBD, we are able to capitalize on the more structured modelling approach and additional national-level data used in that study.

Third, this study considers not only differences in HIV prevalence at a single point in time, but also temporal trends over an 18-year period. While estimates for the most recent year are likely the most useful from a programmatic or policy standpoint, information about recent trends can also provide some suggestion of what to expect in the near future.

Finally, to our knowledge this is the first study to make estimates readily available via a web-based visualization tool, which we believe will make these estimates more accessible. We intend to regularly update this analysis so that contemporary, comprehensive data continue to be available. Subnational estimates of HIV prevalence or PLHIV are already in use in some contexts, for example, for geographic priority setting in annual PEPFAR country operational plans[54]. The underlying data sources and methodologies used to produce these estimates are often unclear based on publicly available documentation.

5.2 Limitations

This methodology and the resulting estimates are subject to a number of limitations.

Most importantly, the accuracy of our estimates is critically dependent on the quantity and quality of the underlying data. We have constructed a large database of geo-located HIV prevalence data for the purposes of this analysis; nonetheless important gaps in data coverage, both spatial and temporal, remain (Extended Data Figures 1-3). In addition, there are several factors related to data quality that should be

acknowledged. Survey data are subject to non-response bias. This is potentially a particular concern for HIV prevalence data, as participation rates in the blood test portion of household surveys are typically lower than response rates for the questionnaire portion[55]. We carried out a complete-case analysis and have made no explicit adjustment for potential non-response bias. Future research should consider the impact of non-response on HIV prevalence estimates and potentially include adjustments alongside geostatistical methods for mapping prevalence. Survey data used to construct covariates may also be subject to biases. In addition to non-response bias, recall bias and social desirability bias are potential concerns for these self-reported outcomes[56]. If these biases impact our covariate estimates, particularly in a manner that varies spatially and temporally, these errors could potentially be propagated to the HIV prevalence estimates. However, our out-of-sample validation analysis found no evidence of increased bias or total error (as measured by RMSE) in HIV prevalence as estimated from models using covariates compared to models that do not use covariates.

The location information associated with the data compiled for this analysis is subject to some error. In order to protect respondent confidentiality, most surveys that collect GPS coordinates perform some type of random displacement on those coordinates prior to releasing data for secondary analysis: for example, GPS coordinates for Demographic and Health Surveys (DHS) are displaced by up to 2 km for urban clusters, up to 5 km for most rural clusters, and up to 10 km in a random 1% of rural clusters[57]. Past research has found that displacement can degrade the predictive power of a geostatistical model; however, this effect was found to be modest, and researchers concluded that relatively accurate mapping can be undertaken at a 5 x 5-km resolution even with GPS displacement[58]. ANC data and survey data extracted from reports and scientific literature were manually matched to location information, a process that is inherently associated with some uncertainty and potentially human error. Moreover, ANC data are collected at clinics. We attempted to match these data to the clinic location, but clinic location is only a proxy for where the women attending this clinic reside. Finally, data associated with polygons rather than GPS coordinates were resampled so that they could be included in the geostatistical model, but this process effectively assumes that HIV prevalence is constant over the polygon and may result in overly homogenous estimates in locations with a preponderance of polygon data. This may be the case, for example, in the Southern sub-Saharan Africa region, where an unusually large proportion of the ANC data are associated with polygon rather than point location data and the posterior estimate of the spatial range parameter is noticeably larger than in other regions (Supplemental Table 9). Research on scalable methods for better integration of polygon data in geostatistical models similar to those used in this analysis is currently ongoing.

With respect to the modelling strategy, the primary limitation is the difficulty in assessing model performance at the grid cell level. We used cross-validation to assess model performance, but due to the substantial impact of sampling error on estimates derived from single survey clusters, it was necessary to aggregate both the data and predictions when assessing error. Additionally, while we have attempted to propagate uncertainty from various sources through the different modelling stages, there are some sources of uncertainty that have not been propagated. In particular, it was not computationally feasible to propagate uncertainty from the covariates (all of which are associated with some uncertainty, including those that we modeled specifically for this analysis) or the sub-models in stacking through the geostatistical model. Similarly, although the WorldPop population raster is also composed of estimates associated with some uncertainty, this uncertainty is difficult to quantify and not currently reported, and so we were unable to propagate this uncertainty into our estimates of the number of PLHIV or estimates

of HIV prevalence for administrative subdivisions that were created using population-weighted averages of grid cell estimates. The modelling strategy also incorporates a number of assumptions, which are difficult to check and, if incorrect, may lead to error. In particular, due to data sparsity, we elected to assume that the bias associated with estimates of HIV prevalence derived from ANC sentinel surveillance varied spatially but was constant over time, though it is possible that this bias varied temporally as well. Finally, our model 'borrows strength' spatially and temporally as well as from covariates in order to stabilize estimates in locations and time periods with very small sample sizes and to interpolate in locations and time periods with no directly observed HIV prevalence data. While we think this is broadly appropriate, there may be specific places and times where this methodology fails; for example, abrupt discontinuities in HIV prevalence in neighboring locations or time periods that cannot be described as a function of the covariates we included are unlikely to be reflected in our estimates unless they are directly observed in the underlying HIV data and those data have substantial sample sizes.

Model fitting was carried out using an integrated nested Laplace approximation to the posterior distribution, as implemented in the R-INLA package[48]. Prediction from fitted models was subsequently carried out using the inla.posterior.sample() function, which generates samples from the approximated posterior of the fitted model. Both model fitting and prediction thus require approximations, and these approximations may introduce error. While it is difficult to assess the impact of these approximations in this particular use case, our validation analysis found that our final model has minimal bias and good coverage of the 95% prediction intervals, which provides some reassurance that the approximation method used – as well as other potential sources of error – are not resulting in appreciable bias or poorly described uncertainty in our reported estimates.

5.3 Future directions

There are opportunities and a need to expand this analysis further.

In particular, information on HIV prevalence by age and sex and covering a larger age range would be valuable, given the well-known differences in HIV prevalence by age and sex, the unique needs of children and adolescents who are living with HIV, and the increasing number of PLHIV who are age 50 years or older[52,59].

Even more so, mapping diagnosis rates, treatment rates, incidence, and mortality in addition to prevalence would provide more nuanced tools to target primary prevention, testing, and health care delivery strategies, and would also allow for local tracking of progress toward international targets including SDG3 and the 90-90-90 targets[60,61]. Comprehensively mapping incidence and mortality in sub-Saharan Africa is likely to be considerably more difficult than mapping prevalence, as there are substantially fewer sources of directly observed data[62]. Nonetheless, there is an opportunity to combine the geostatistical methods described here with methods akin to those used by the GBD study and by UNAIDS to estimate incidence and mortality largely on the basis of observed prevalence and the epidemiological relationships between prevalence, incidence, and mortality[52,59]. Moreover, HIV incidence assays – which distinguish between recent and non-recent infections, allowing for direct measurement of population-level HIV incidence – are increasingly common in seroprevalence surveys[63] and in routine health care settings, and could provide an important input into future incidence mapping efforts. Similarly, survey-based data on diagnosis and treatment are also increasingly common and could support future efforts to map diagnosis and treatment rates[63].

This analysis does not speak to the underlying mechanisms driving geographic differences in HIV prevalence or differences in changes in HIV prevalence over time. Future analyses focusing on geographic

differences in HIV incidence and mortality, as well as consideration of migration patterns, will be necessary to assess the extent to which local changes in HIV prevalence observed in this study are due to changes in incidence, mortality, and/or migration. Similarly, future analyses of diagnosis and treatment patterns, potentially coupled with information about structural and behavioural factors related to HIV incidence (including many of the variables constructed as covariates in this analysis), would enable an even more nuanced exploration of the root causes of the geographic differences observed here.

Finally, this analysis generated estimates on a 5 x 5-km grid and then aggregated these estimates to produce estimates for first- and second-order administrative subdivisions, but other levels of analysis are possible. Future research should consider the optimal level of analysis, which likely requires attention to data availability, data quality, and the specificity of the location information associated with available data sources, in addition to consideration of the level of policy action, implementation, and monitoring.

References

1. Global Health Data Exchange | GHDx. Available at: http://ghdx.healthdata.org/. (Accessed: 22nd August 2018)

2. Joint United Nations Programme on HIV/AIDS (UNAIDS). National HIV estimates file. Available at: http://www.unaids.org/en/dataanalysis/datatools/spectrum-epp. (Accessed: 26th August 2017)

3. United States Census Bureau. HIV/AIDS Database. Available at: https://www.census.gov/programs-surveys/international-programs/about/hiv.html. (Accessed: 12th January 2018)

4. Wiegand, H. Kish, L.: Survey Sampling. John Wiley & Sons, Inc., New York, London 1965, IX + 643 S., 31 Abb., 56 Tab., Preis 83 s. *Biom. Z.* **10**, 88–89 (1968).

5. King, G., Tomz, M. & Wittenberg, J. Making the most of statistical analyses: improving interpretation and presentation. *Am. J. Polit. Sci.* **44**, 341–355 (2000).

6. Golding, N. *et al.* Mapping under-5 and neonatal mortality in Africa, 2000–15: a baseline analysis for the Sustainable Development Goals. *The Lancet* **390**, 2171–2182 (2017).

7. Tatem, A. J., Hemelaar, J., Gray, R. R. & Salemi, M. Spatial accessibility and the spread of HIV-1 subtypes and recombinants. *AIDS* **26**, 2351 (2012).

8. Gray, R. R. *et al.* Spatial phylodynamics of HIV-1 epidemic emergence in east Africa. *AIDS* **23**, F9–F17 (2009).

9. Abu-Raddad, L. J., Patnaik, P. & Kublin, J. G. Dual infection with HIV and malaria fuels the spread of both diseases in sub-Saharan Africa. *Science* **314**, 1603–1606 (2006).

10. Cuadros, D. F., Branscum, A. J. & Crowley, P. H. HIV-malaria co-infection: effects of malaria on the prevalence of HIV in East sub-Saharan Africa. *Int. J. Epidemiol.* **40**, 931–939 (2011).

11. Auvert, B. *et al.* Ecological and individual level analysis of risk factors for HIV infection in four urban populations in sub-Saharan Africa with different levels of HIV infection. *AIDS* **15 Suppl 4**, S15-30 (2001).

12. Bailey, R. C., Plummer, F. A. & Moses, S. Male circumcision and HIV prevention: current knowledge and future research directions. *Lancet Infect. Dis.* **1**, 223–231 (2001).

13. Bailey, R. C. *et al.* Male circumcision for HIV prevention in young men in Kisumu, Kenya: a randomised controlled trial. *The Lancet* **369**, 643–656 (2007).

14. World Health Organization. UNAIDS statement on South African trial findings regarding male circumcision and HIV. *WHO* (2005). Available at: https://www.who.int/mediacentre/news/releases/2005/pr32/en/. (Accessed: 22nd February 2019)

15. Joint United Nations Programme on HIV/AIDS (UNAIDS), UNICEF, UNFPA & World Health Organization. *Male Circumcision and HIV Fact Sheet*. 3 (2005).

16. Sgaier, S. K., Reed, J. B., Thomas, A. & Njeuhmeli, E. Achieving the HIV prevention impact of voluntary medical male circumcision: lessons and challenges for managing programs. *PLOS Med.* **11**, e1001641 (2014).

17. Freeman, E. E. *et al.* Herpes simplex virus 2 infection increases HIV acquisition in men and women: systematic review and meta-analysis of longitudinal studies. *AIDS* **20**, 73–83 (2006).

18. Chen, L. *et al.* Sexual risk factors for HIV infection in early and advanced HIV epidemics in sub-Saharan Africa: systematic overview of 68 epidemiological studies. *PLOS ONE* **2**, e1001 (2007).

19. Glynn, J. R. *et al.* Why do young women have a much higher prevalence of HIV than young men? A study in Kisumu, Kenya and Ndola, Zambia. *AIDS* **15 Suppl 4**, S51-60 (2001).

20. Johnson, K. & Way, A. Risk factors for HIV infection in a national adult population: evidence from the 2003 Kenya Demographic and Health Survey. *J. Acquir. Immune Defic. Syndr.* **42**, 627–636 (2006).

21. Pettifor, A. E., van der Straten, A., Dunbar, M. S., Shiboski, S. C. & Padian, N. S. Early age of first sex: a risk factor for HIV infection among women in Zimbabwe. *AIDS* **18**, 1435–1442 (2004).

22. Coffee, M. P. *et al.* Patterns of movement and risk of HIV infection in rural Zimbabwe. *J. Infect. Dis.* **191 Suppl 1**, S159-167 (2005).

23. Bwayo, J. *et al.* Human immunodeficiency virus infection in long-distance truck drivers in east Africa. *Arch. Intern. Med.* **154**, 1391–1396 (1994).

24. Davis, K. R. & Weller, S. C. The effectiveness of condoms in reducing heterosexual transmission of HIV. *Fam. Plann. Perspect.* **31**, 272–279 (1999).

25. Duncan, M. E. *et al.* First coitus before menarche and risk of sexually transmitted disease. *Lancet* **335**, 338–340 (1990).

26. Stöckl, H., Kalra, N., Jacobi, J. & Watts, C. Is early sexual debut a risk factor for HIV infection among women in sub-Saharan Africa? A systematic review. *Am. J. Reprod. Immunol.* **69 Suppl 1**, 27–40 (2013).

27. Carswell, J. W., Lloyd, G. & Howells, J. Prevalence of HIV-1 in east African lorry drivers. *AIDS* **3**, 759–761 (1989).

28. Brown, N. R. & Sinclair, R. C. Estimating number of lifetime sexual partners: Men and women do it differently. *J. Sex Res.* **36**, 292–297 (1999).

29. GeoNetwork. GeoNetwork opensource portal to spatial data and information. (2015). Available at: http://www.fao.org/geonetwork/srv/en/metadata.show?id=%2012691. (Accessed: 26th January 2016)

30. GADM. Available at: https://gadm.org/. (Accessed: 6th May 2018)

31. WorldPop. Worldpop dataset. Available at: http://www.worldpop.org.uk/data/get_data/. (Accessed: 7th July 2017)

32. Tatem, A. J. WorldPop, open data for spatial demography. *Sci. Data* **4**, 170004 (2017).

33. Breiman, L. Stacked regressions. *Mach. Learn.* **24**, 49–64 (1996).

34. Bhatt, S. *et al.* Improved prediction accuracy for disease risk mapping using Gaussian process stacked generalization. *J. R. Soc. Interface* **14**, (2017).

35. Osgood-Zimmerman, A. *et al.* Mapping child growth failure in Africa between 2000 and 2015. *Nature* **555**, 41–47 (2018).

36. Graetz, N. *et al.* Mapping local variation in educational attainment across Africa. *Nature* **555**, 48–53 (2018).

37. Reiner, R. C. *et al.* Variation in Childhood Diarrheal Morbidity and Mortality in Africa, 2000–2015. *N. Engl. J. Med.* **379**, 1128–1138 (2018).

38. Gouws, E., Mishra, V. & Fowler, T. B. Comparison of adult HIV prevalence from national population-based surveys and antenatal clinic surveillance in countries with generalised epidemics: implications for calibrating surveillance data. *Sex. Transm. Infect.* **84**, i17–i23 (2008).

39. Marsh, K., Mahy, M., Salomon, J. A. & Hogan, D. R. Assessing and adjusting for differences between HIV prevalence estimates derived from national population-based surveys and antenatal care surveillance, with applications for Spectrum 2013. *AIDS* **28 Suppl 4**, S497-505 (2014).

40. Kongnyuy, E. J. & Wiysonge, C. S. Association between fertility and HIV status: what implications for HIV estimates? *BMC Public Health* **8**, 309 (2008).

41. Zaba, B. & Gregson, S. Measuring the impact of HIV on fertility in Africa. *AIDS* **12 Suppl 1**, S41-50 (1998).

42. Giorgi, E., Sesay, S. S. S., Terlouw, D. J. & Diggle, P. J. Combining data from multiple spatially referenced prevalence surveys using generalized linear geostatistical models. *J. R. Stat. Soc.* **178**, 445–464 (2015).

43. Diggle, P. J. & Giorgi, E. Model-Based Geostatistics for Prevalence Mapping in Low-Resource Settings. *J. Am. Stat. Assoc.* **111**, 1096–1120 (2016).

44. Simpson, D. P., Rue, H., Martins, T. G., Riebler, A. & Sørbye, S. H. Penalising model component complexity: A principled, practical approach to constructing priors. *Stat. Sci.* (2014).

45. Franco-Villoria, M., Ventrucci, M. & Rue, H. Bayesian varying coefficient models using PC priors. *arXiv:1806.02084* (2018).

46. Fuglstad, G.-A., Simpson, D., Lindgren, F. & Rue, H. Constructing Priors that Penalize the Complexity of Gaussian Random Fields. *J. Am. Stat. Assoc.* (2015).

47. Blangiardo, M. & Cameletti, M. *Spatial and spatio-temporal Bayesian models with R-INLA*. (John Wiley and Sons, Inc, 2015).

48. Rue, H., Martino, S. & Chopin, N. Approximate Bayesian inference for latent Gaussian models by using integrated nested Laplace approximations. *J. R. Stat. Soc.* **71**, 319–392 (2009).

49. Lindgren, F., Rue, H. & Lindström, J. An explicit link between Gaussian fields and Gaussian Markov random fields: the stochastic partial differential equation approach. *J. R. Stat. Soc. Ser. B Stat. Methodol.* **73**, 423–498 (2011).

50. Murray, C. J. *et al.* GBD 2010: design, definitions, and metrics. *The Lancet* **380**, 2063–2066 (2012).

51. Waller, L. A. Estimate suggests many infant deaths in sub-Saharan Africa attributable to air pollution. *Nature* **559**, 188 (2018).

52. James, S. L. *et al.* Global, regional, and national incidence, prevalence, and years lived with disability for 354 diseases and injuries for 195 countries and territories, 1990–2017: a systematic analysis for the Global Burden of Disease Study 2017. *The Lancet* **392**, 1789–1858 (2018).

53. Murray, C. J. L. *et al.* Population and fertility by age and sex for 195 countries and territories, 1950–2017: a systematic analysis for the Global Burden of Disease Study 2017. *The Lancet* **392**, 1995–2051 (2018).

54. The United States President's Emergency Plan for AIDS Relief (PEPFAR). FY 2018 Country Operational Plans. Available at: https://www.pepfar.gov/countries/cop/fy2018/index.htm. (Accessed: 21st November 2018)

55. Mishra, V., Hong, R., Khan, S., Gu, Y. & Liu, L. Evaluating HIV estimates from national population-based surveys for bias resulting from non-response. (2008). Available at: http://dhsprogram.com/publications/publication-as12-analytical-studies.cfm. (Accessed: 22nd August 2018)

56. Curtis, S. & Sutherland, E. Measuring sexual behaviour in the era of HIV/AIDS: the experience of Demographic and Health Surveys and similar enquiries. *Sex. Transm. Infect.* **80**, ii22–ii27 (2004).

57. Burgert, C. R., Colston, J., Roy, T. & Zachary, B. Geographic displacement procedure and georeferenced data release policy for the Demographic and Health Surveys. (2013).

58. Gething, P., Tatem, A., Bird, T. & Burgert-Brucker, C. R. Creating spatial interpolation surfaces with DHS data DHS Spatial Analysis Reports No. 11. *ICF Int.* (2015).

59. Joint United Nations Programme on HIV/AIDS (UNAIDS). AIDSinfo | UNAIDS. Available at: http://aidsinfo.unaids.org/. (Accessed: 24th August 2018)

60. Transforming our World: The 2030 Agenda for Sustainable Development. Available at: https://sustainabledevelopment.un.org/post2015/transformingourworld/publication. (Accessed: 24th August 2018)

61. Joint United Nations Programme on HIV/AIDS (UNAIDS). Fast-Track - Ending the AIDS epidemic by 2030. (2014). Available at: http://www.unaids.org/en/resources/documents/2014/JC2686_WAD2014report. (Accessed: 24th August 2018)

62. Ghys, P. D., Williams, B. G., Over, M., Hallett, T. B. & Godfrey-Faussett, P. Epidemiological metrics and benchmarks for a transition in the HIV epidemic. *PLOS Med.* **15**, e1002678 (2018).

63. Justman, J. E., Mugurungi, O. & El-Sadr, W. M. HIV Population Surveys – Bringing Precision to the Global Response. *N. Engl. J. Med.* **378**, 1859–1861 (2018).

64. Land Processes Distributed Active Archive Center. Combined MODIS 5.1 dataset. *MCD12Q1 | LP DAAC :: NASA Land Data Products and Services* Available at: https://lpdaac.usgs.gov/dataset_discovery/modis/modis_products_table/mcd12q1. (Accessed: 1st June 2017)

65. Lehner, B. & Doll, P. Development and validation of a global database of lakes, reservoirs and wetlands. *J. Hydrol.* (2004).

66. World Wildlife Fund. Global Lakes and Wetlands Database Level 3. *World Wildlife Fund* (2004). Available at: https://www.worldwildlife.org/pages/global-lakes-and-wetlands-database. (Accessed: 1st June 2017)

Supplementary Figures

Supplementary Figure 1: HIV prevalence, adults ages 15-49, 2000

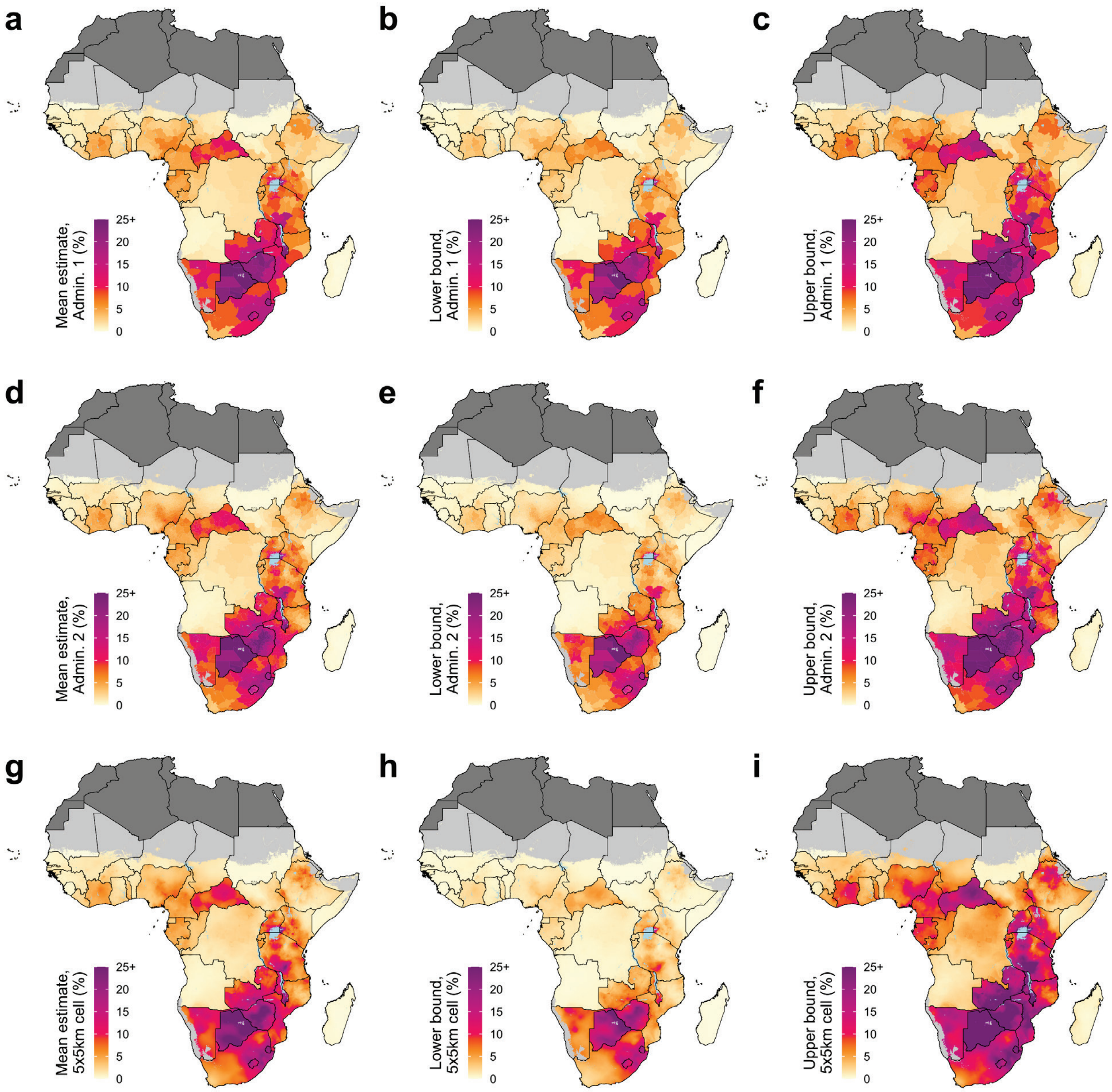

Supplementary Figure 1: HIV prevalence, adults ages 15-49, 2000. HIV prevalence among adults age 15-49 in 2000 at the first administrative subdivision level (**a-c**), second administrative subdivision level (**d-f**) and 5 x 5-km grid cell level (**g-i**). Mean estimates and lower and upper bounds of the 95% uncertainty intervals are shown in the left, middle, and right columns, respectively. Maps reflect administrative boundaries, land cover, lakes, and population; grid cells with fewer than 10 people per 1 x 1-km and classified as "barren or sparsely vegetated" are coloured in light grey[29,31,32,64–66]. Countries in dark grey were not included in the analysis.

Supplementary Figure 2: HIV prevalence, adults ages 15-49, 2005

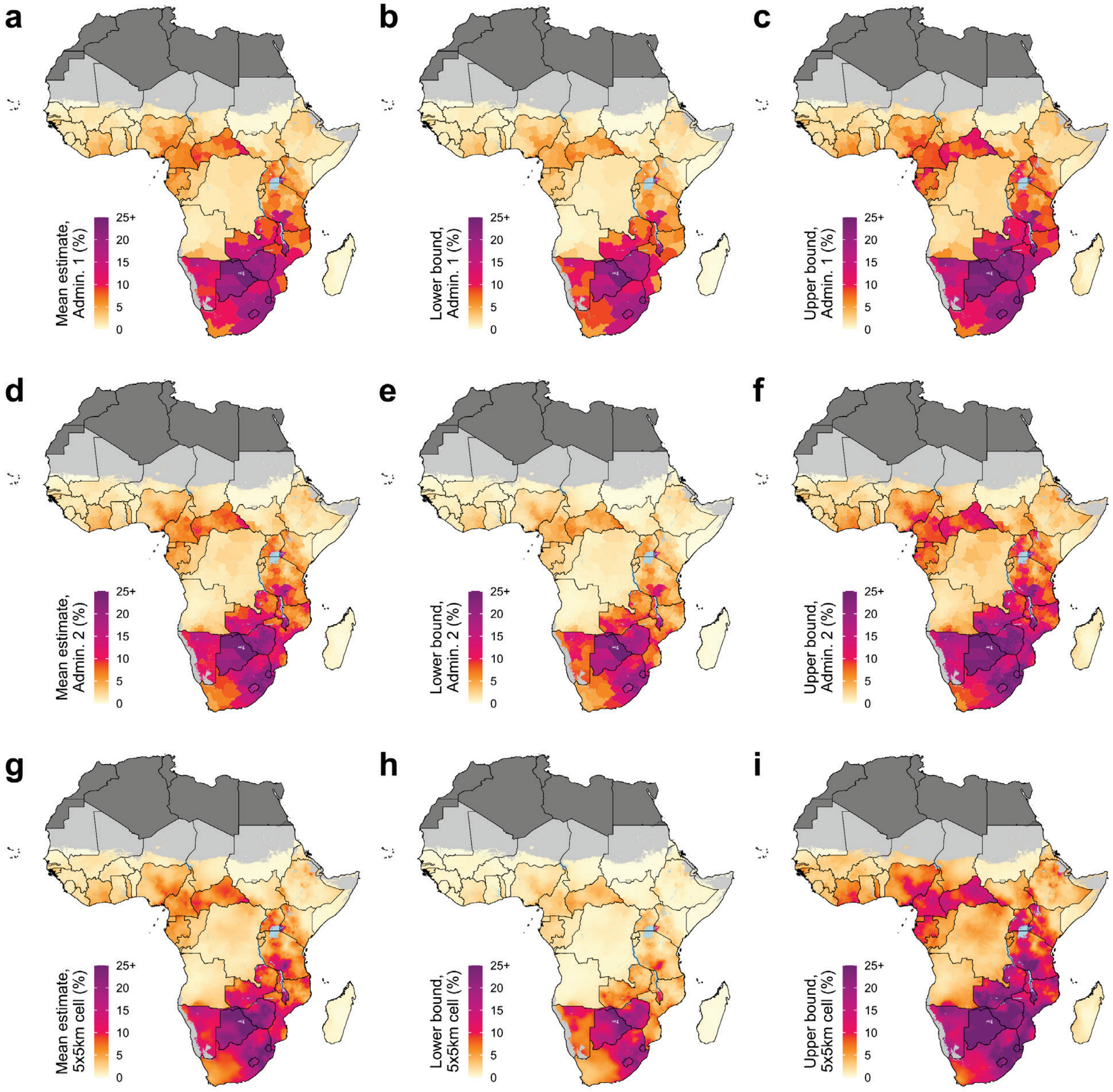

Supplementary Figure 2: HIV prevalence, adults ages 15-49, 2005. HIV prevalence among adults age 15-49 in 2005 at the first administrative subdivision level (**a-c**), second administrative subdivision level (**d-f**), and 5 x 5-km grid cell level (**g-i**). Mean estimates and lower and upper bounds of the 95% uncertainty intervals are shown in the left, middle, and right columns, respectively. Maps reflect administrative boundaries, land cover, lakes, and population; grid cells with fewer than 10 people per 1 x 1-km and classified as "barren or sparsely vegetated" are coloured in light grey[29,31,32,64–66]. Countries in dark grey were not included in the analysis.

Supplementary Figure 3: HIV prevalence, adults ages 15-49, 2010

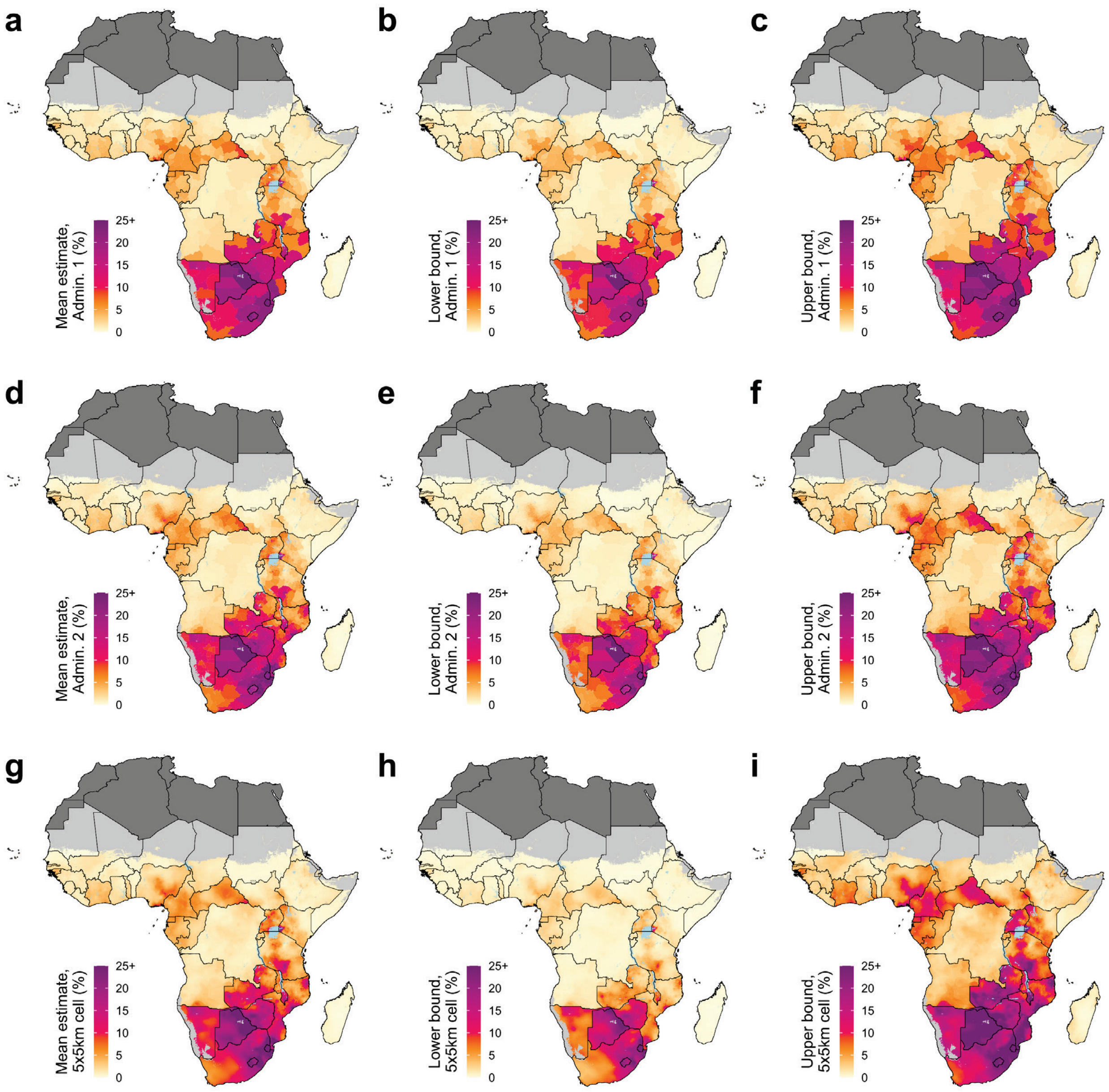

Supplementary Figure 3: HIV prevalence, adults ages 15-49, 2010. HIV prevalence among adults age 15-49 in 2010 at the first administrative subdivision level (**a-c**), second administrative subdivision level (**d-f**), and 5 x 5-km grid cell level (**g-i**). Mean estimates and lower and upper bounds of the 95% uncertainty intervals are shown in the left, middle, and right columns, respectively. Maps reflect administrative boundaries, land cover, lakes, and population; grid cells with fewer than 10 people per 1 x 1-km and classified as "barren or sparsely vegetated" are coloured in light grey[29,31,32,64–66]. Countries in dark grey were not included in the analysis.

Supplementary Figure 4: HIV prevalence, adults ages 15-49, 2017

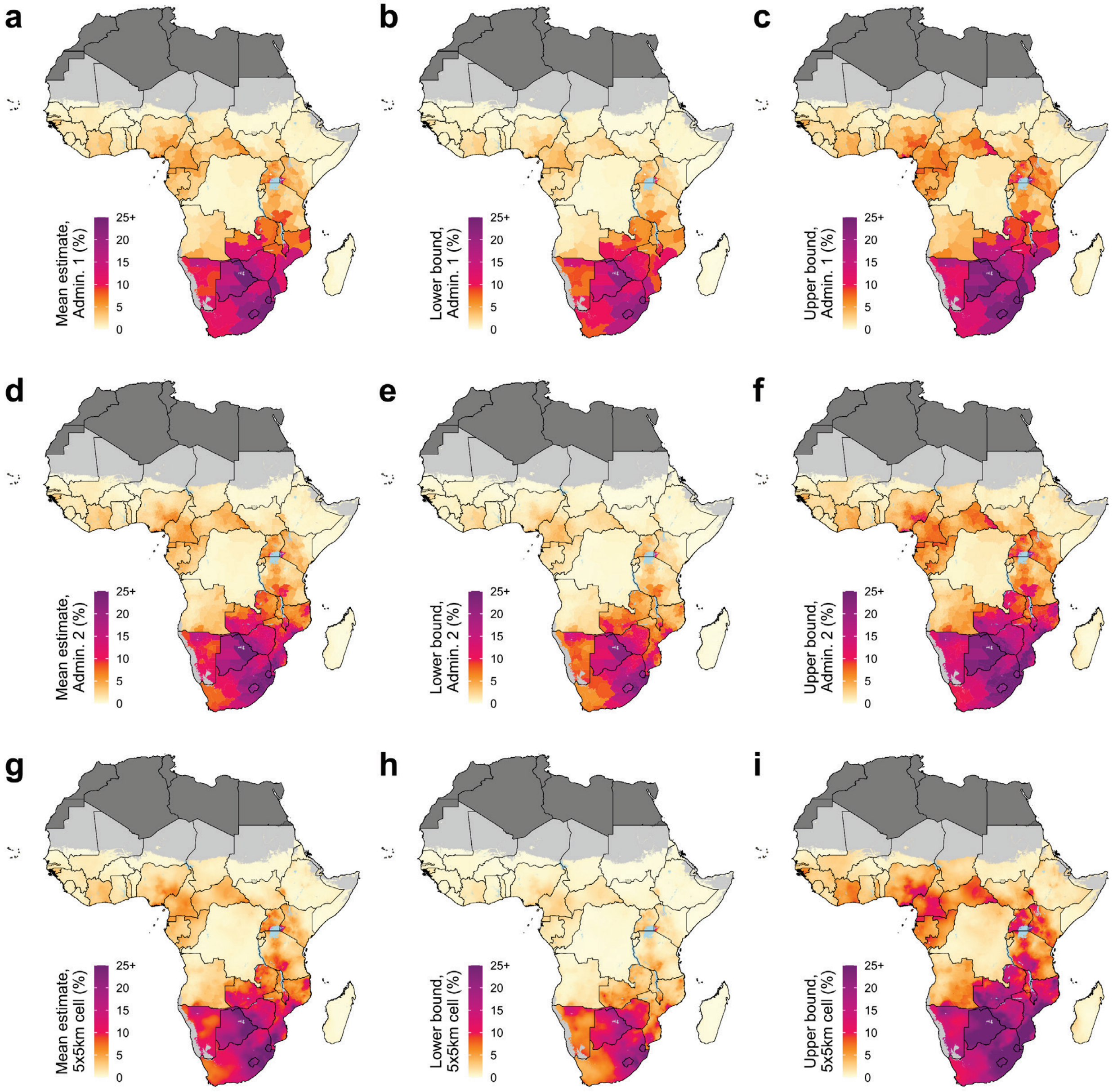

Supplementary Figure 4: HIV prevalence, adults ages 15-49, 2017. HIV prevalence among adults age 15-49 in 2017 at the first administrative subdivision level (**a-c**), second administrative subdivision level (**d-f**), and 5 x 5-km grid cell level (**g-i**). Mean estimates and lower and upper bounds of the 95% uncertainty intervals are shown in the left, middle, and right columns, respectively. Maps reflect administrative boundaries, land cover, lakes, and population; grid cells with fewer than 10 people per 1 x 1-km and classified as "barren or sparsely vegetated" are coloured in light grey[29,31,32,64–66]. Countries in dark grey were not included in the analysis.

Supplementary Figure 5: Posterior probability of an increase in HIV prevalence, adults ages 15-49, 2000–2017

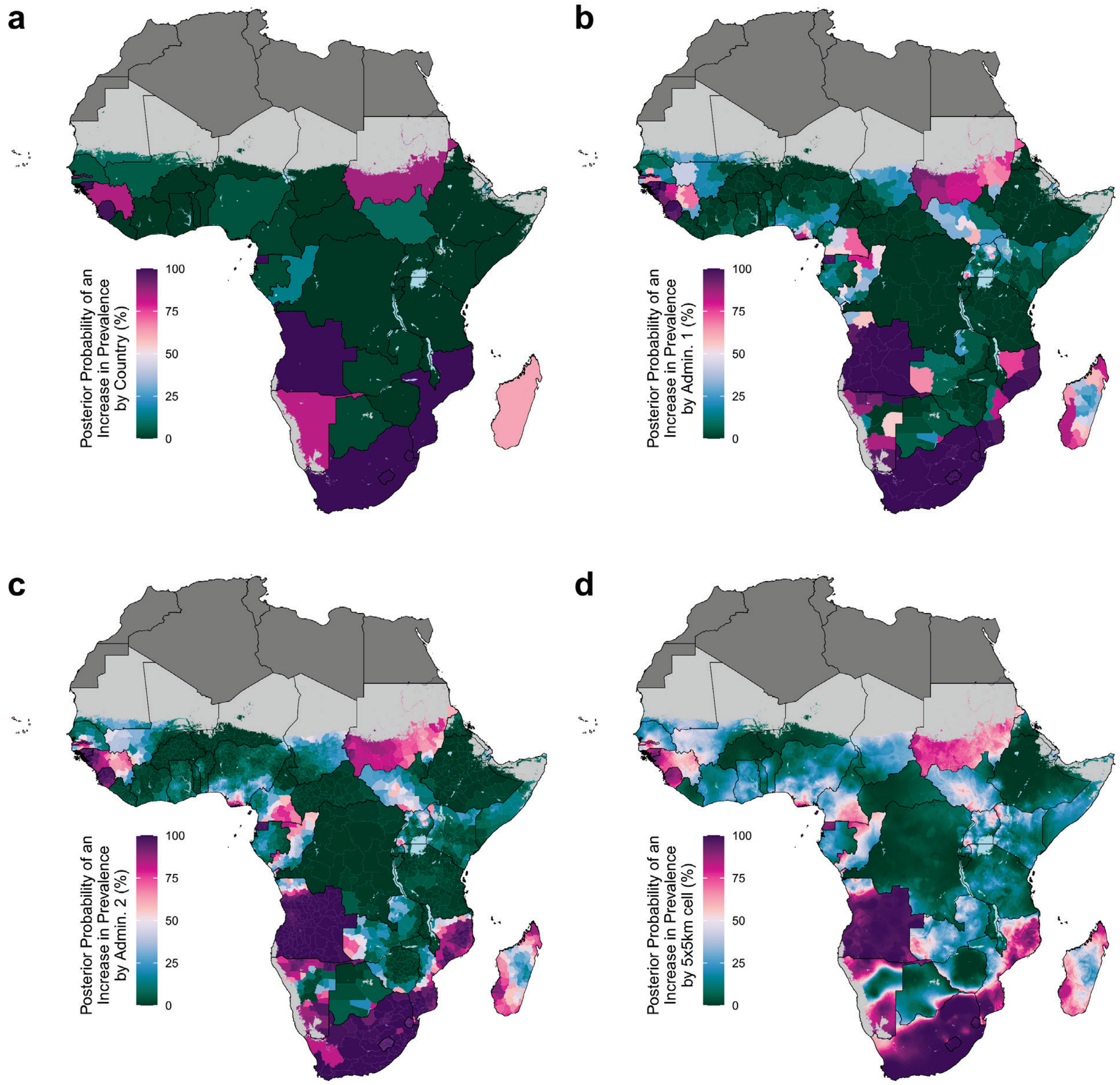

Supplementary Figure 5: Posterior probability of an increase in HIV prevalence, adults ages 15-49, 2000–2017. Posterior probability of an increase in HIV prevalence among adults ages 15-49 from 2000 to 2017, calculated as the percentage of draws from the approximated posterior distribution in which HIV prevalence for a given area increased from 2000 to 2017 at the country level (**a**), first administrative subdivision level (**b**), second administrative subdivision level (**c**), and 5 x 5-km grid cell level (**d**). High posterior probability of an increase in HIV prevalence indicates a high degree of confidence that HIV prevalence increased in a given area, while low posterior probability of an increase in HIV prevalence

indicates a high degree of confidence that HIV prevalence decreased in a given area. Maps reflect administrative boundaries, land cover, lakes, and population; grid cells with fewer than 10 people per 1 x 1-km and classified as “barren or sparsely vegetated” are coloured in light grey[29,31,32,64–66]. Countries in dark grey were not included in the analysis.

Supplementary Figure 6: Change in HIV prevalence, adults ages 15-49, 2000–2005

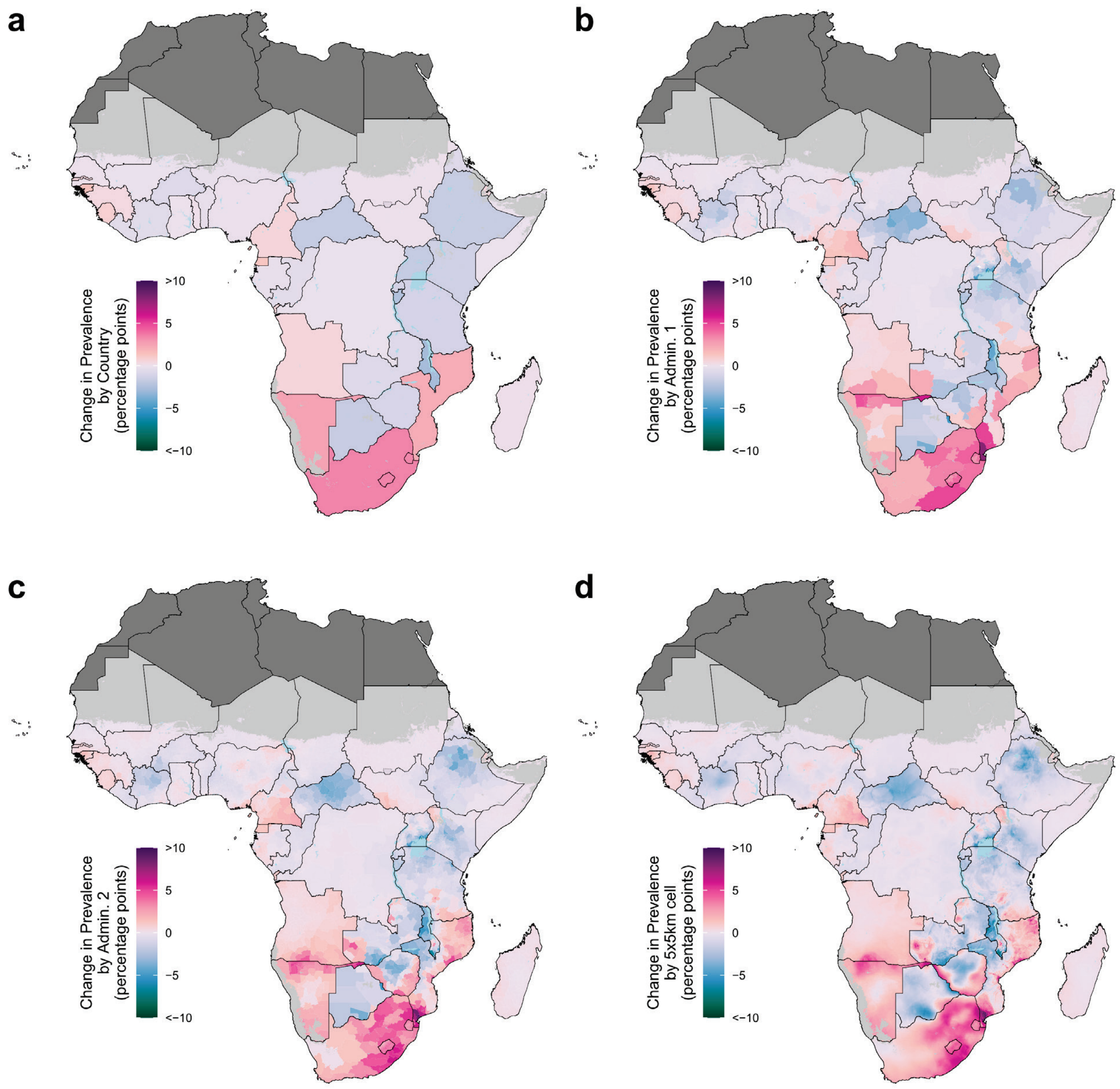

Supplementary Figure 6: Change in HIV prevalence, adults 15-49, 2000–2005. Absolute change in HIV prevalence among adults age 15-49 between 2000 and 2005 at the country level (**a**), first administrative subdivision level (**b**), second administrative subdivision level (**c**), and 5 × 5-km grid cell level (**d**). Maps reflect administrative boundaries, land cover, lakes, and population; grid cells with fewer than 10 people per 1 x 1-km and classified as “barren or sparsely vegetated” are coloured in light grey[29,31,32,64–66]. Countries in dark grey were not included in the analysis.

Supplementary Figure 7: Change in HIV prevalence, adults age 15-49, 2005–2010

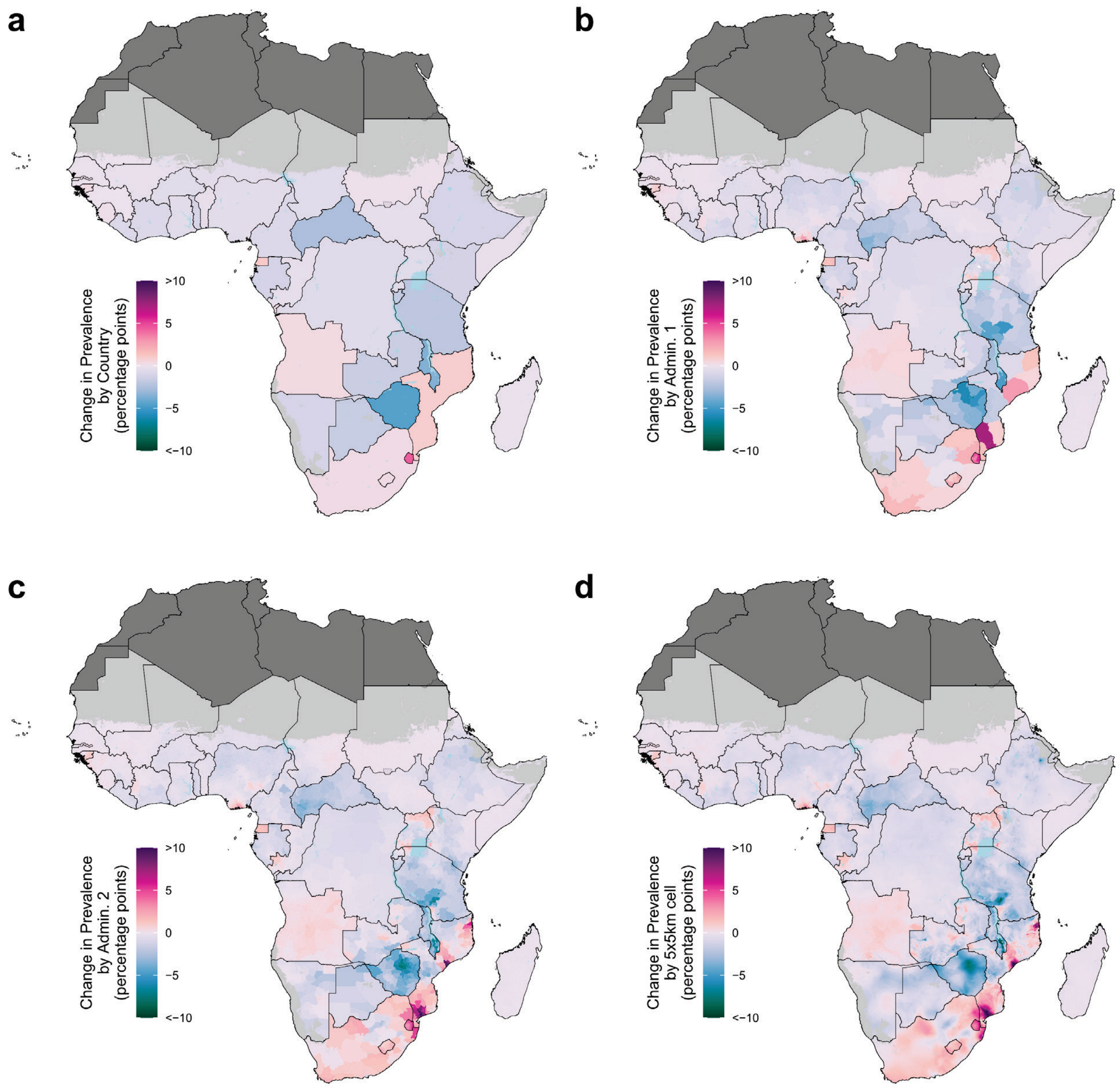

Supplementary Figure 7: Change in HIV prevalence, adults age 15-49, 2005–2010. Absolute change in HIV prevalence among adults age 15-49 between 2005 and 2010 at the country level (**a**), first administrative subdivision level (**b**), second administrative subdivision level (**c**), and 5 × 5-km grid cell level (**d**). Maps reflect administrative boundaries, land cover, lakes, and population; grid cells with fewer than 10 people per 1 x 1-km and classified as “barren or sparsely vegetated” are coloured in light grey[29,31,32,64–66]. Countries in dark grey were not included in the analysis.

Supplementary Figure 8: Change in HIV prevalence, adults ages 15-49, 2010–2017

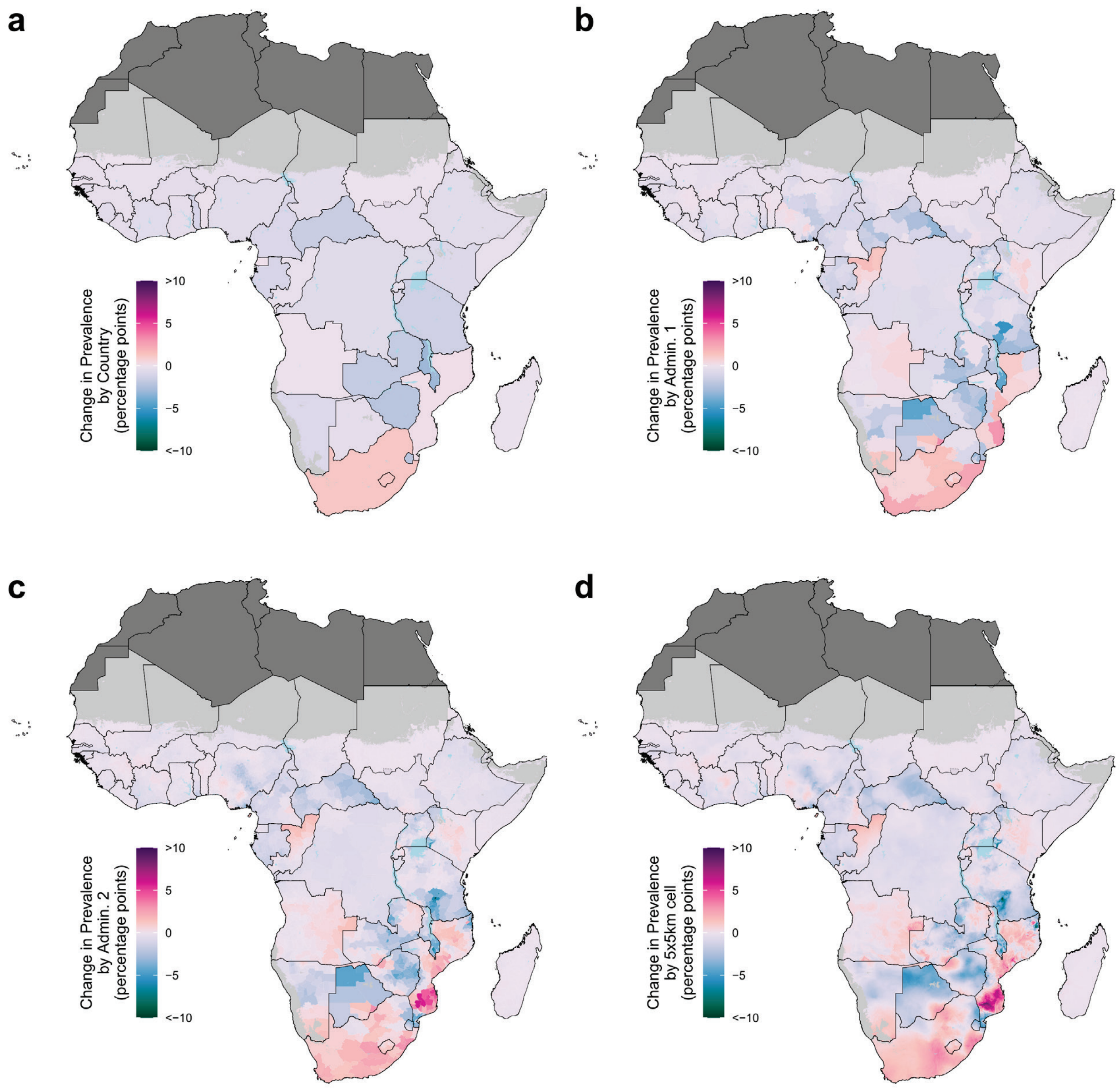

Supplementary Figure 8: Change in HIV prevalence, adults 15-49, 2010–2017. Absolute change in HIV prevalence among adults ages 15-49 between 2010 and 2017 at the country level (**a**), first administrative subdivision level (**b**), second administrative subdivision level (**c**), and 5 × 5-km grid cell level (**d**). Maps reflect administrative boundaries, land cover, lakes, and population; grid cells with fewer than 10 people per 1 x 1-km and classified as “barren or sparsely vegetated” are coloured in light grey[29,31,32,64–66]. Countries in dark grey were not included in the analysis.

Supplementary Figure 9: Prevalence of male circumcision in 2000, 2005, 2010, and 2017

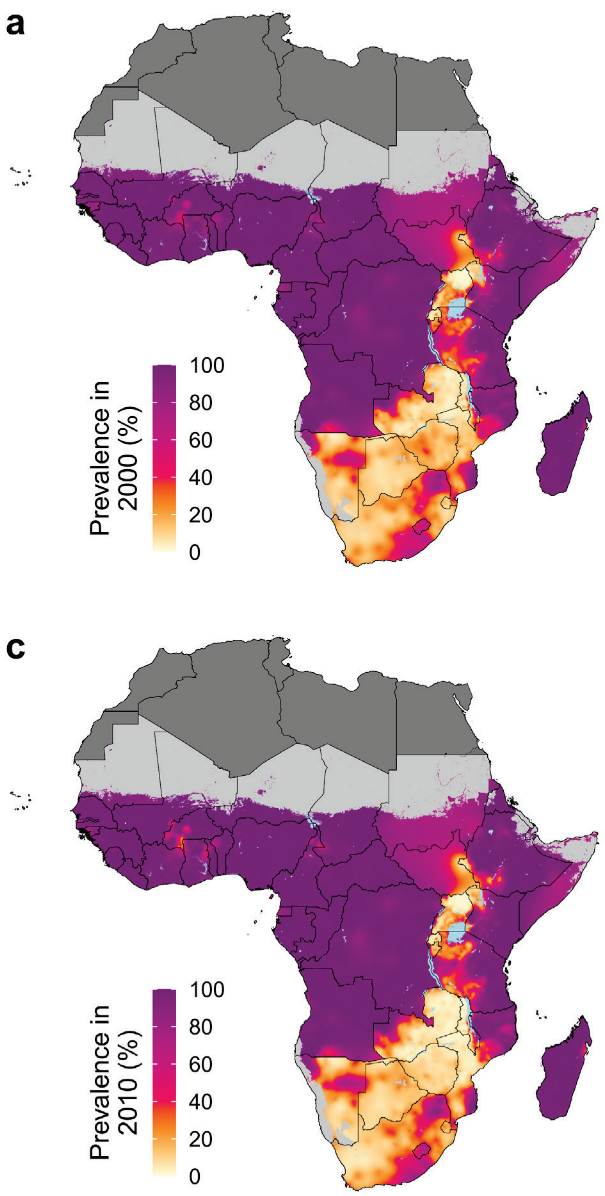

Supplementary Figure 9: Prevalence of male circumcision in 2000, 2005, 2010, and 2017. Prevalence of male circumcision, including medical or traditional circumcision, among men ages 15-49 at the 5 x 5-km grid cell level in 2000 (**a**), 2005 (**b**), 2010 (**c**), and 2017 (**d**). Maps reflect administrative boundaries, land cover, lakes, and population; grid cells with fewer than 10 people per 1 x 1-km and classified as "barren or sparsely vegetated" are coloured in light grey[29,31,32,64–66]. Countries in dark grey were not included in the analysis.

Supplementary Figure 10: Prevalence of signs and symptoms of sexually transmitted infections in 2000, 2005, 2010, and 2017

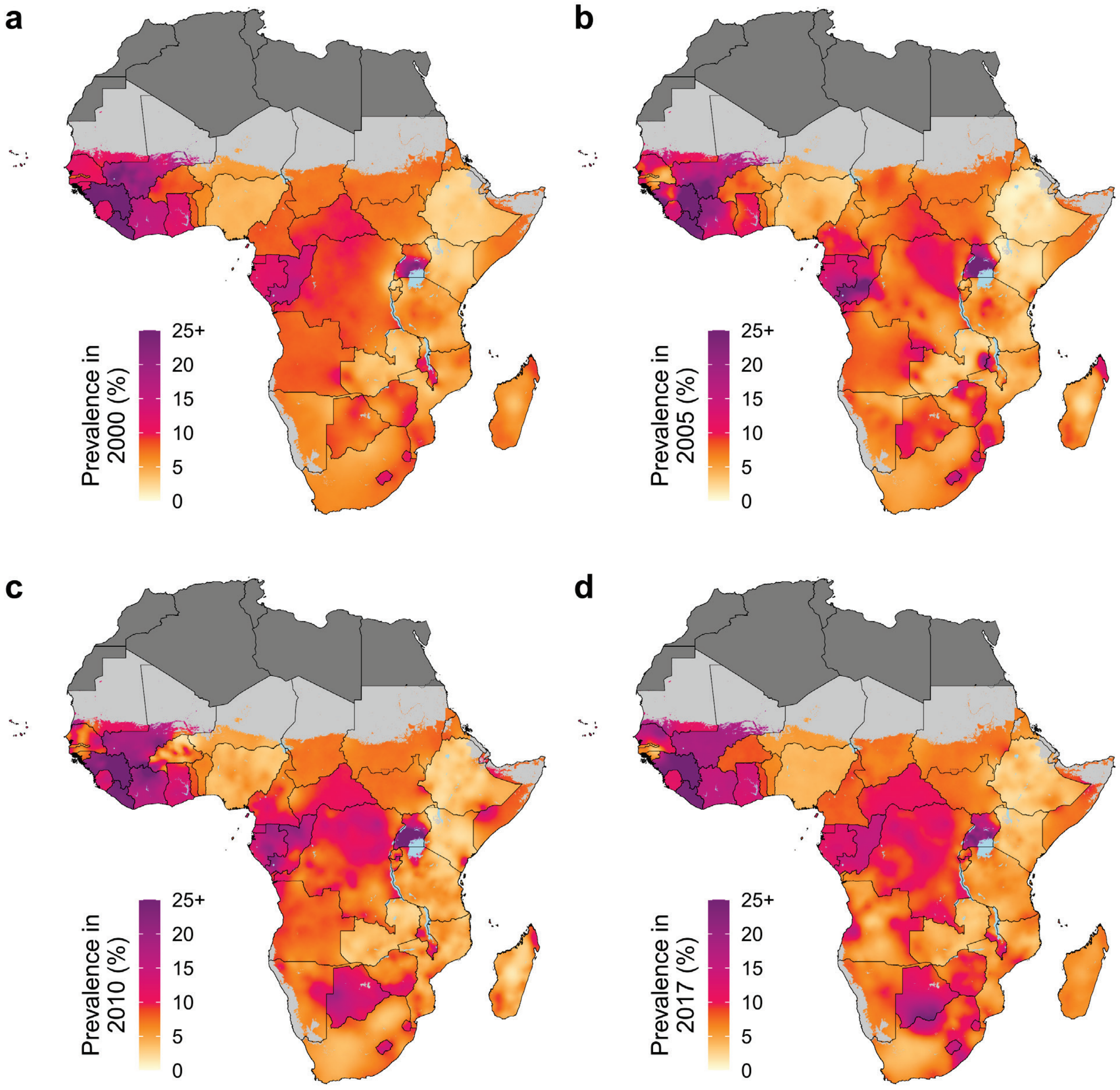

Supplementary Figure 10: Prevalence of signs and symptoms of sexually transmitted infections in 2000, 2005, 2010, and 2017. Prevalence of self-reported STI symptoms (genital discharge and/or genital ulcer/sore) in the last 12 months among adults ages 15-49 at the 5 x 5-km grid cell level in 2000 (**a**), 2005 (**b**), 2010 (**c**), and 2017 (**d**). Maps reflect administrative boundaries, land cover, lakes, and population; grid cells with fewer than 10 people per 1 x 1-km and classified as "barren or sparsely vegetated" are coloured in light grey[29,31,32,64–66]. Countries in dark grey were not included in the analysis.

Supplementary Figure 11: Prevalence of marriage or living as married in 2000, 2005, 2010, and 2017

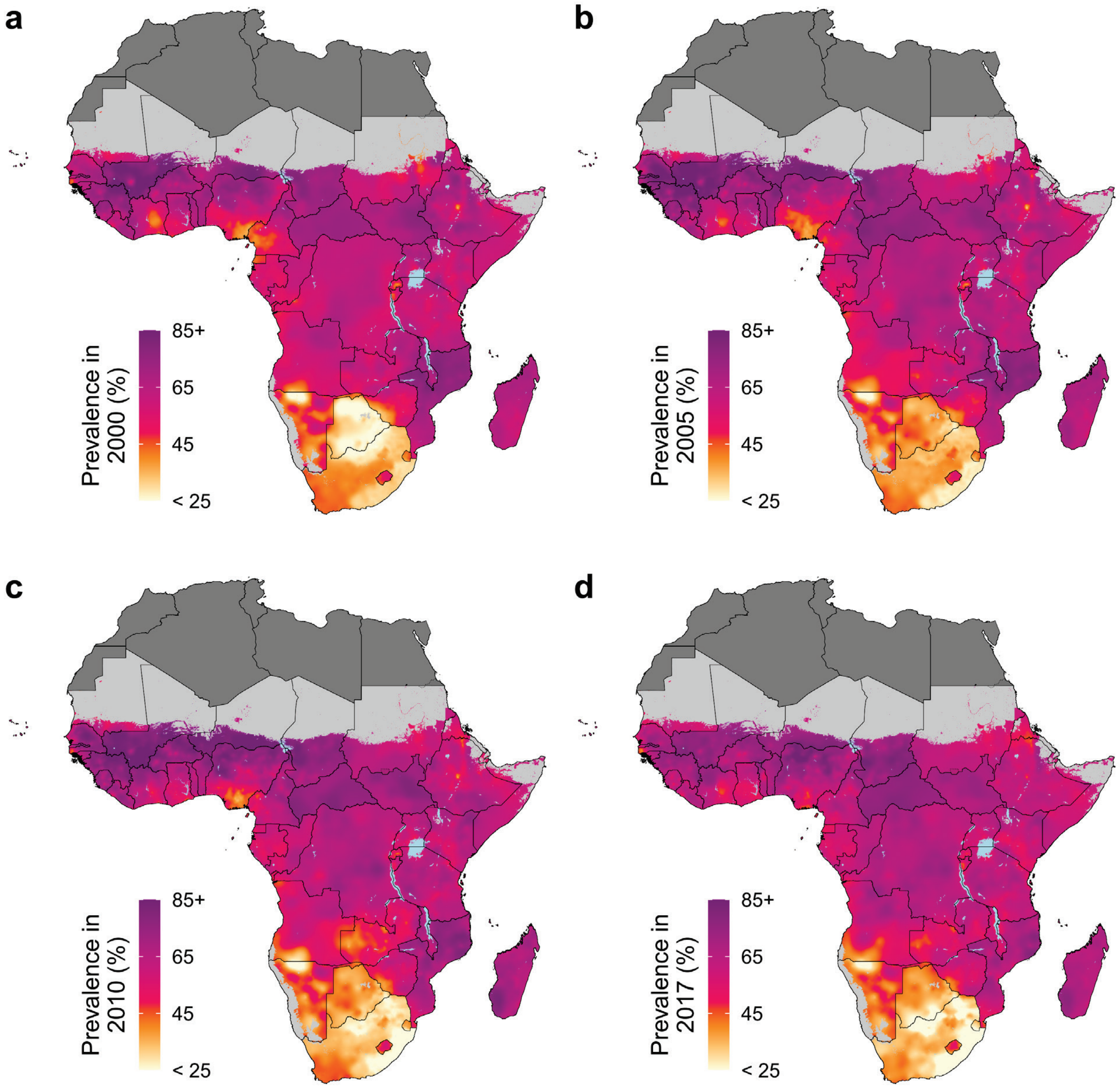

Supplementary Figure 11: Prevalence of marriage or living as married in 2000, 2005, 2010, and 2017. Prevalence of marriage or living as married among adults ages 15-49 at the 5 x 5-km grid cell level in 2000 (**a**), 2005 (**b**), 2010 (**c**), and 2017 (**d**). Maps reflect administrative boundaries, land cover, lakes, and population; grid cells with fewer than 10 people per 1 x 1-km and classified as "barren or sparsely vegetated" are coloured in light grey[29,31,32,64–66]. Countries in dark grey were not included in the analysis.

Supplementary Figure 12: Prevalence of partner living elsewhere among women in 2000, 2005, 2010, and 2017

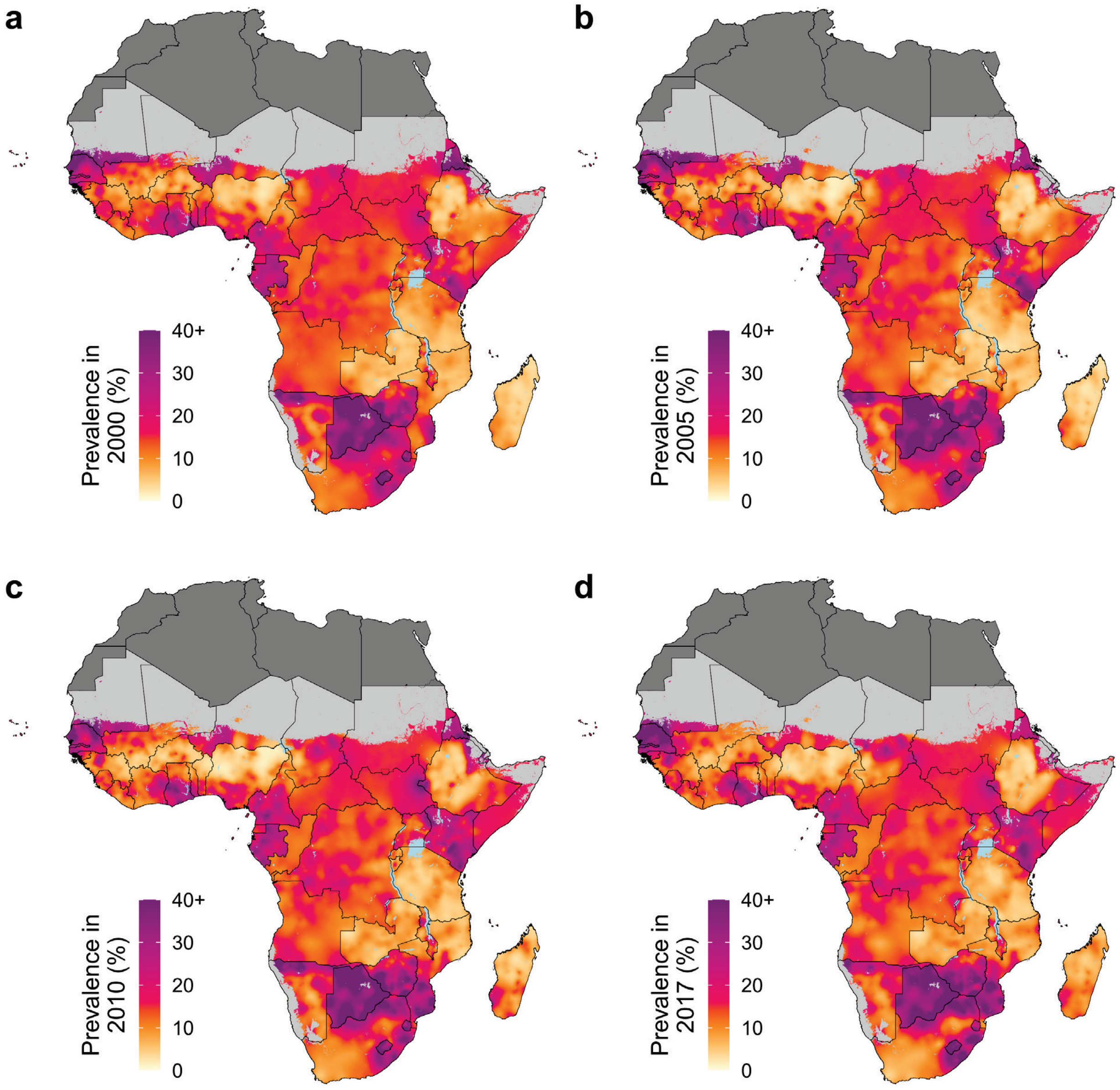

Supplementary Figure 12: Prevalence of partner living elsewhere among women in 2000, 2005, 2010, and 2017. Prevalence of partner living elsewhere among women ages 15-49 at the 5 x 5-km grid cell level in 2000 (**a**), 2005 (**b**), 2010 (**c**), and 2017 (**d**). Maps reflect administrative boundaries, land cover, lakes, and population; grid cells with fewer than 10 people per 1 x 1-km and classified as "barren or sparsely vegetated" are coloured in light grey[29,31,32,64–66]. Countries in dark grey were not included in the analysis.

Supplementary Figure 13: Prevalence of condom use during most recent sexual encounter in 2000, 2005, 2010, and 2017

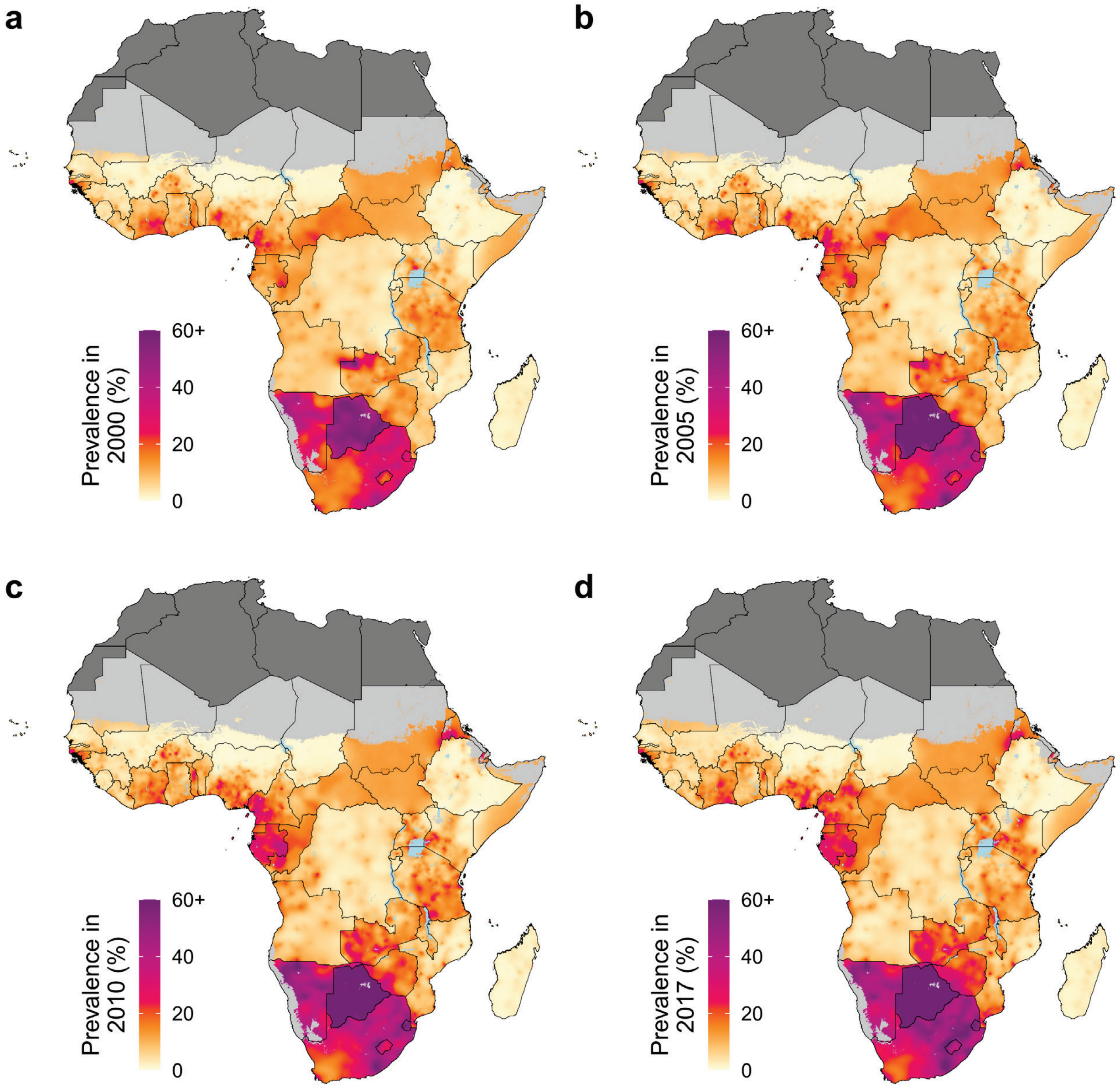

Supplementary Figure 13: Prevalence of condom use during most recent sexual encounter in 2000, 2005, 2010, and 2017. Prevalence of condom use during most recent sexual encounter within the last 12 months among adults ages 15-49 at the 5 x 5-km grid cell level in 2000 (**a**), 2005 (**b**), 2010 (**c**), and 2017 (**d**). Maps reflect administrative boundaries, land cover, lakes, and population; grid cells with fewer than 10 people per 1 x 1-km and classified as "barren or sparsely vegetated" are coloured in light grey[29,31,32,64–66]. Countries in dark grey were not included in the analysis.

Supplementary Figure 14: Prevalence of sexual activity among young adults in 2000, 2005, 2010, and 2017

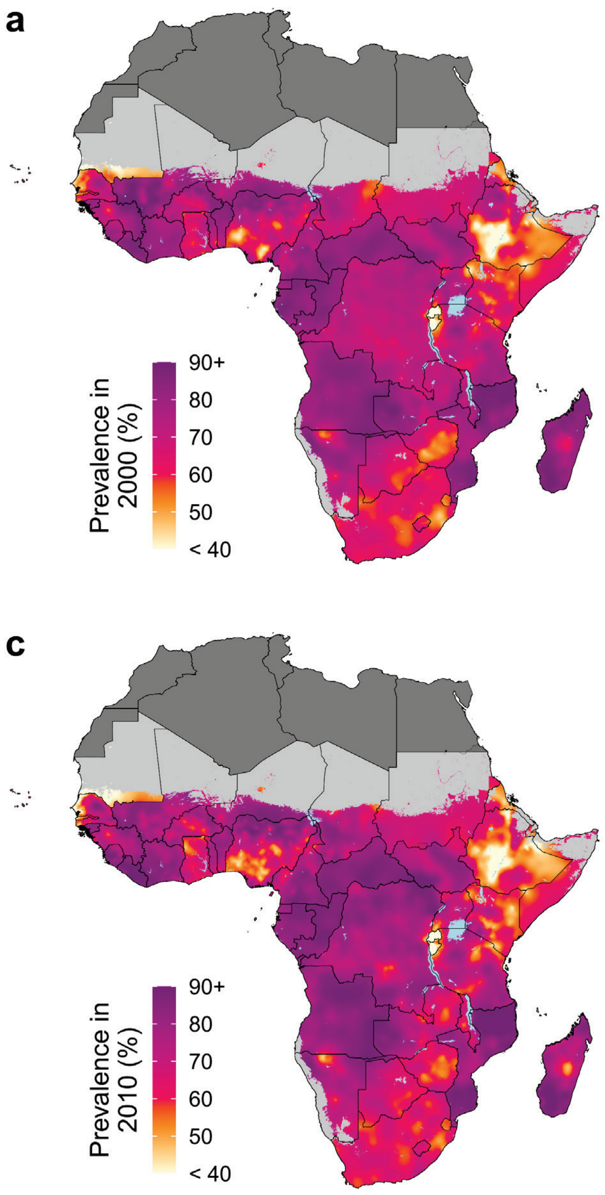

Supplementary Figure 14: Prevalence of sexual activity among young adults in 2000, 2005, 2010, and 2017. Prevalence of sexual activity among young adults ages 15-24 at the 5 x 5-km grid cell level in 2000 (**a**), 2005 (**b**), 2010 (**c**), and 2017 (**d**). Maps reflect administrative boundaries, land cover, lakes, and population; grid cells with fewer than 10 people per 1 x 1-km and classified as "barren or sparsely vegetated" are coloured in light grey[29,31,32,64–66]. Countries in dark grey were not included in the analysis.

Supplementary Figure 15: Prevalence of multiple partners among men in the past year in 2000, 2005, 2010, and 2017

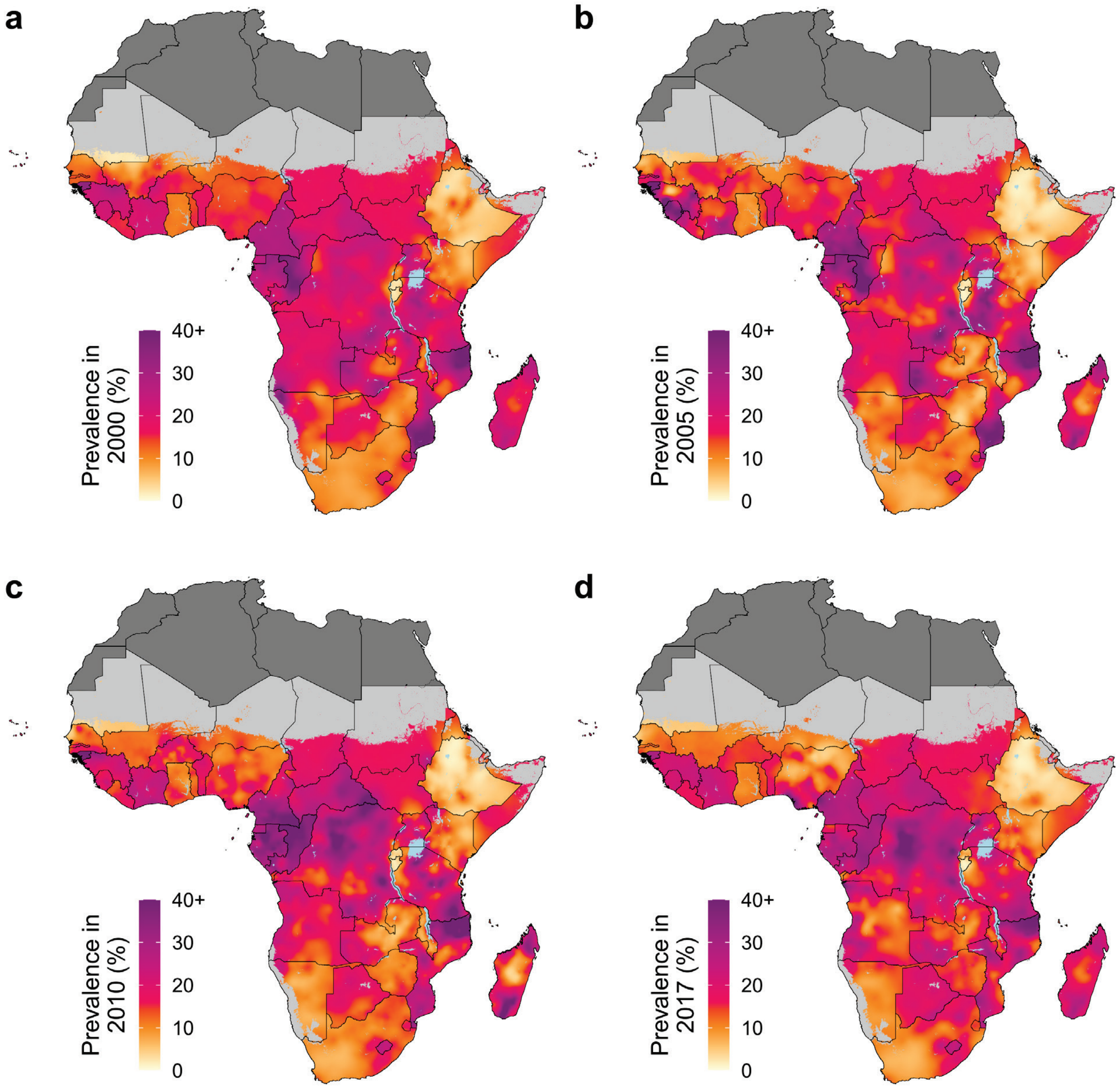

Supplementary Figure 15: Prevalence of multiple partners among men in the past year in 2000, 2005, 2010, and 2017. Prevalence of multiple partners among men ages 15-49 at the 5 x 5-km grid cell level in 2000 (**a**), 2005 (**b**), 2010 (**c**), and 2017 (**d**). Maps reflect administrative boundaries, land cover, lakes, and population; grid cells with fewer than 10 people per 1 x 1-km and classified as "barren or sparsely vegetated" are coloured in light grey[29,31,32,64–66]. Countries in dark grey were not included in the analysis.

Supplementary Figure 16: Prevalence of multiple partners among women in the past year in 2000, 2005, 2010, and 2017

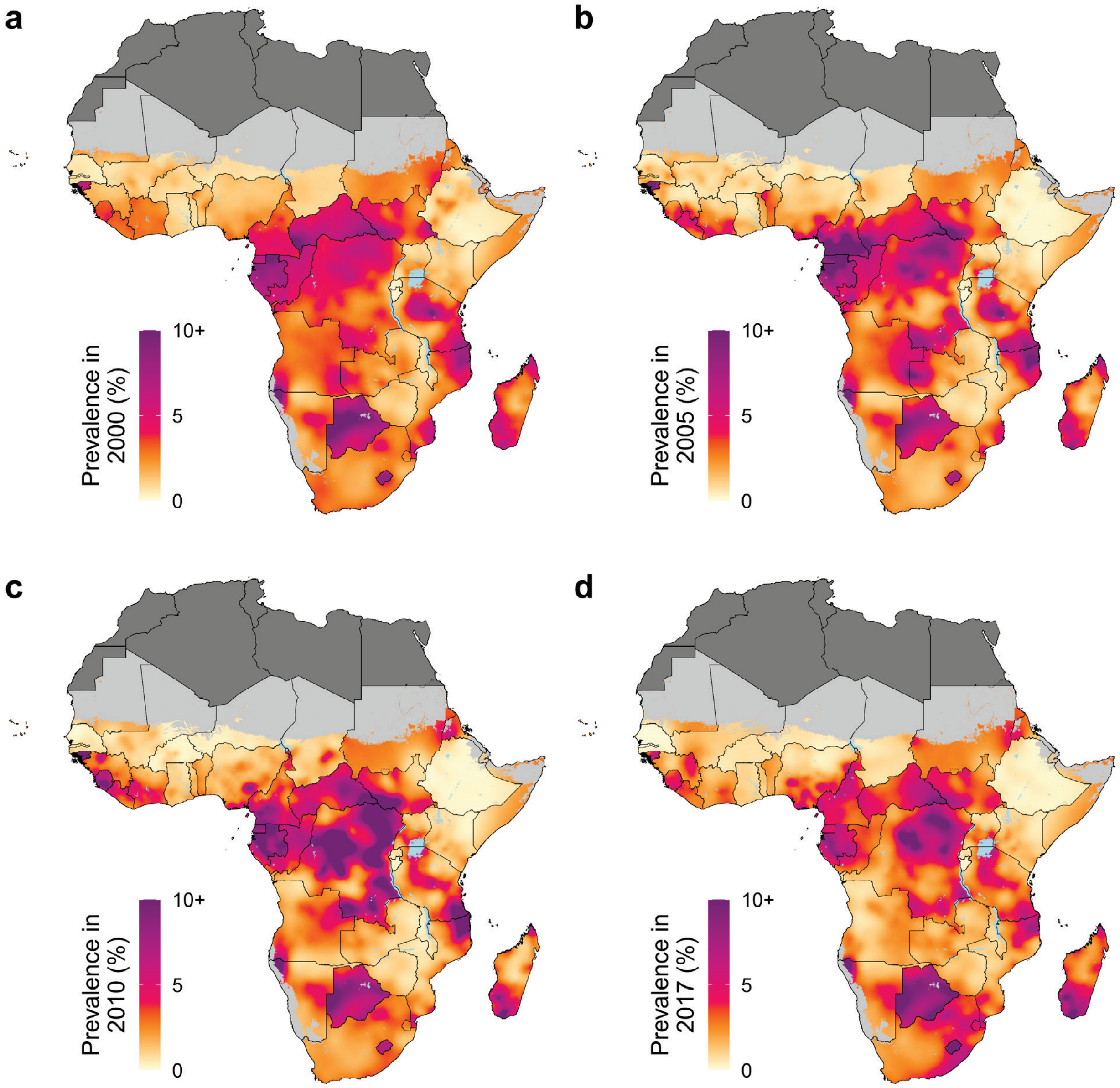

Supplementary Figure 16: Prevalence of multiple partners among women in the past year in 2000, 2005, 2010, and 2017. Prevalence of multiple partners among women ages 15-49 at the 5 x 5-km grid cell level in 2000 (**a**), 2005 (**b**), 2010 (**c**), and 2017 (**d**). Maps reflect administrative boundaries, land cover, lakes, and population; grid cells with fewer than 10 people per 1 x 1-km and classified as "barren or sparsely vegetated" are coloured in light grey[29,31,32,64–66]. Countries in dark grey were not included in the analysis.

Supplementary Figure 17: HIV prevalence predictions from the boosted regression tree model in 2000, 2005, 2010, and 2017

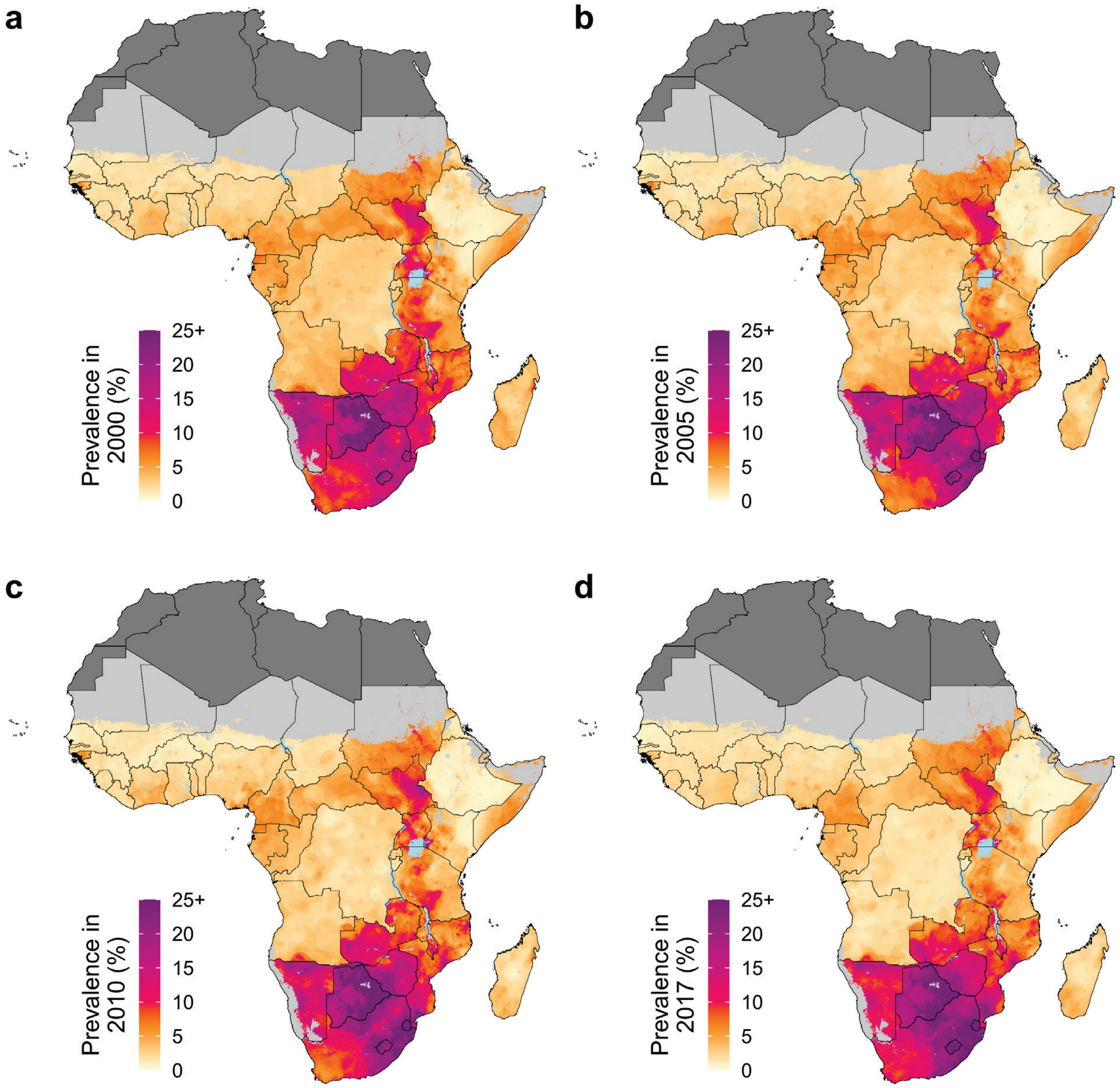

Supplementary Figure 17: HIV prevalence predictions from the boosted regression tree model in 2000, 2005, 2010, and 2017. HIV prevalence among adults ages 15-49 at the 5 x 5-km grid cell level in 2000 (**a**), 2005 (**b**), 2010 (**c**), and 2017 (**d**) as estimated in the boosted regression tree model. Maps reflect administrative boundaries, land cover, lakes, and population; grid cells with fewer than 10 people per 1 x 1-km and classified as "barren or sparsely vegetated" are coloured in light grey[29,31,32,64–66]. Countries in dark grey were not included in the analysis.

Supplementary Figure 18: HIV prevalence predictions from the generalized additive model in 2000, 2005, 2010, and 2017

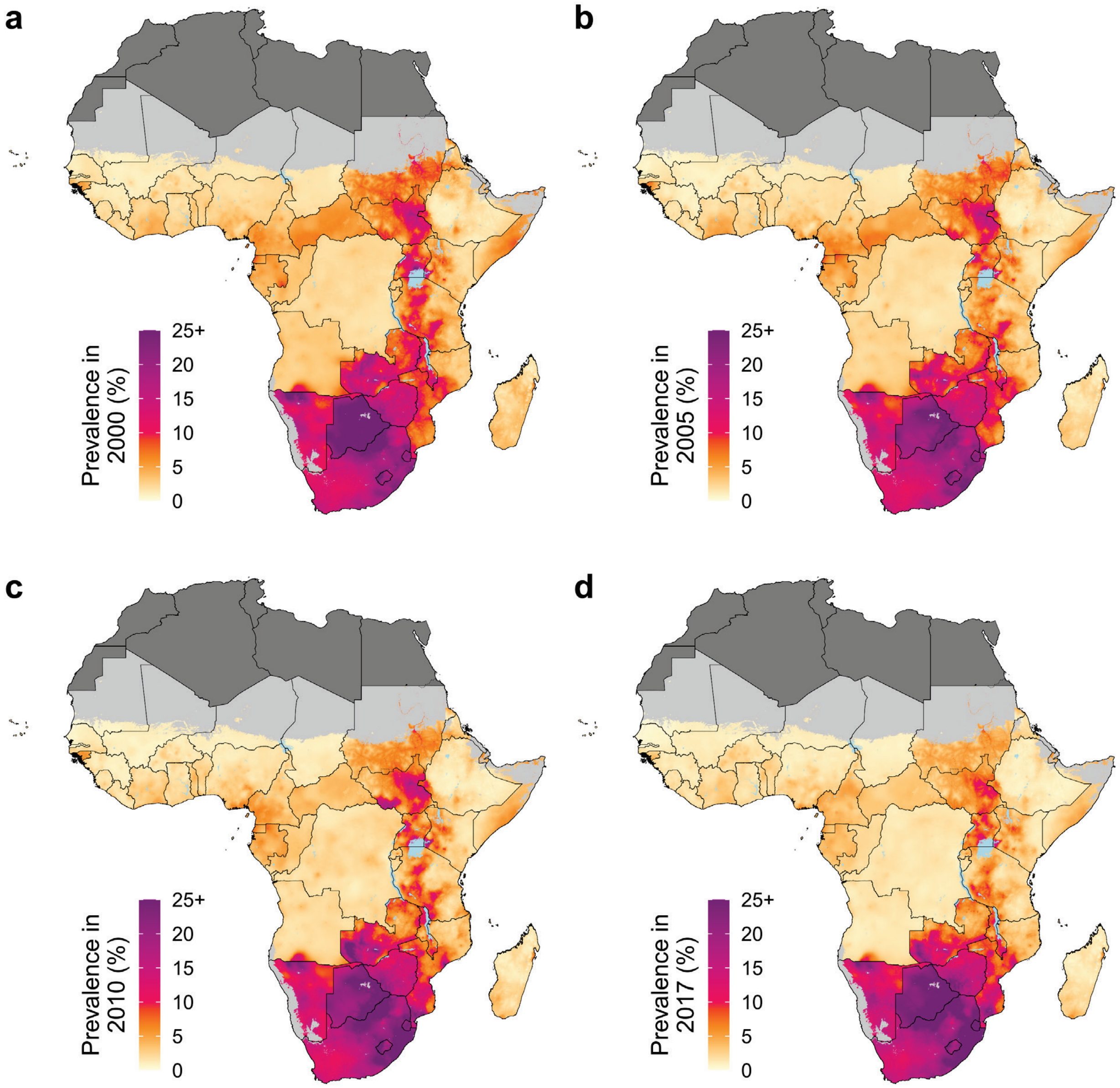

Supplementary Figure 18: HIV prevalence predictions from the generalized additive model in 2000, 2005, 2010, and 2017. HIV prevalence among adults ages 15-49 at the 5 x 5-km grid cell level in 2000 (**a**), 2005 (**b**), 2010 (**c**), and 2017 (**d**) as estimated in the generalized additive model. Maps reflect administrative boundaries, land cover, lakes, and population; grid cells with fewer than 10 people per 1 x 1-km and classified as "barren or sparsely vegetated" are coloured in light grey[29,31,32,64–66]. Countries in dark grey were not included in the analysis.

Supplementary Figure 19: HIV prevalence predictions from the lasso regression model in 2000, 2005, 2010, and 2017

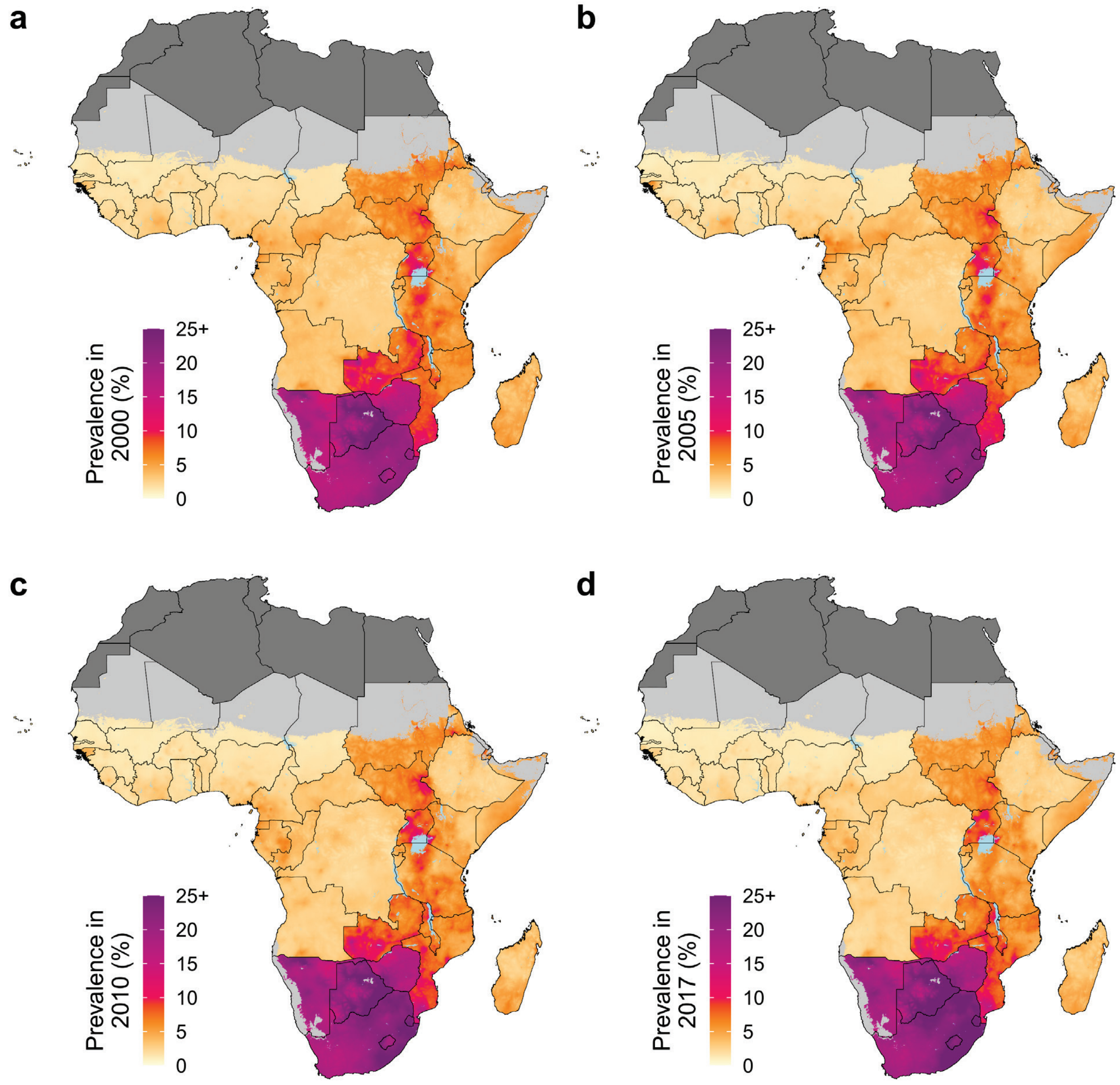

Supplementary Figure 19: HIV prevalence predictions from the lasso regression model in 2000, 2005, 2010, and 2017. HIV prevalence among adults ages 15-49 at the 5 x 5-km grid cell level in 2000 (**a**), 2005 (**b**), 2010 (**c**), and 2017 (**d**) as estimated in the lasso regression model. Maps reflect administrative boundaries, land cover, lakes, and population; grid cells with fewer than 10 people per 1 x 1-km and classified as “barren or sparsely vegetated” are coloured in light grey[29,31,32,64–66]. Countries in dark grey were not included in the analysis.

Supplementary Figure 20: Validation metrics from the covariate sensitivity analysis

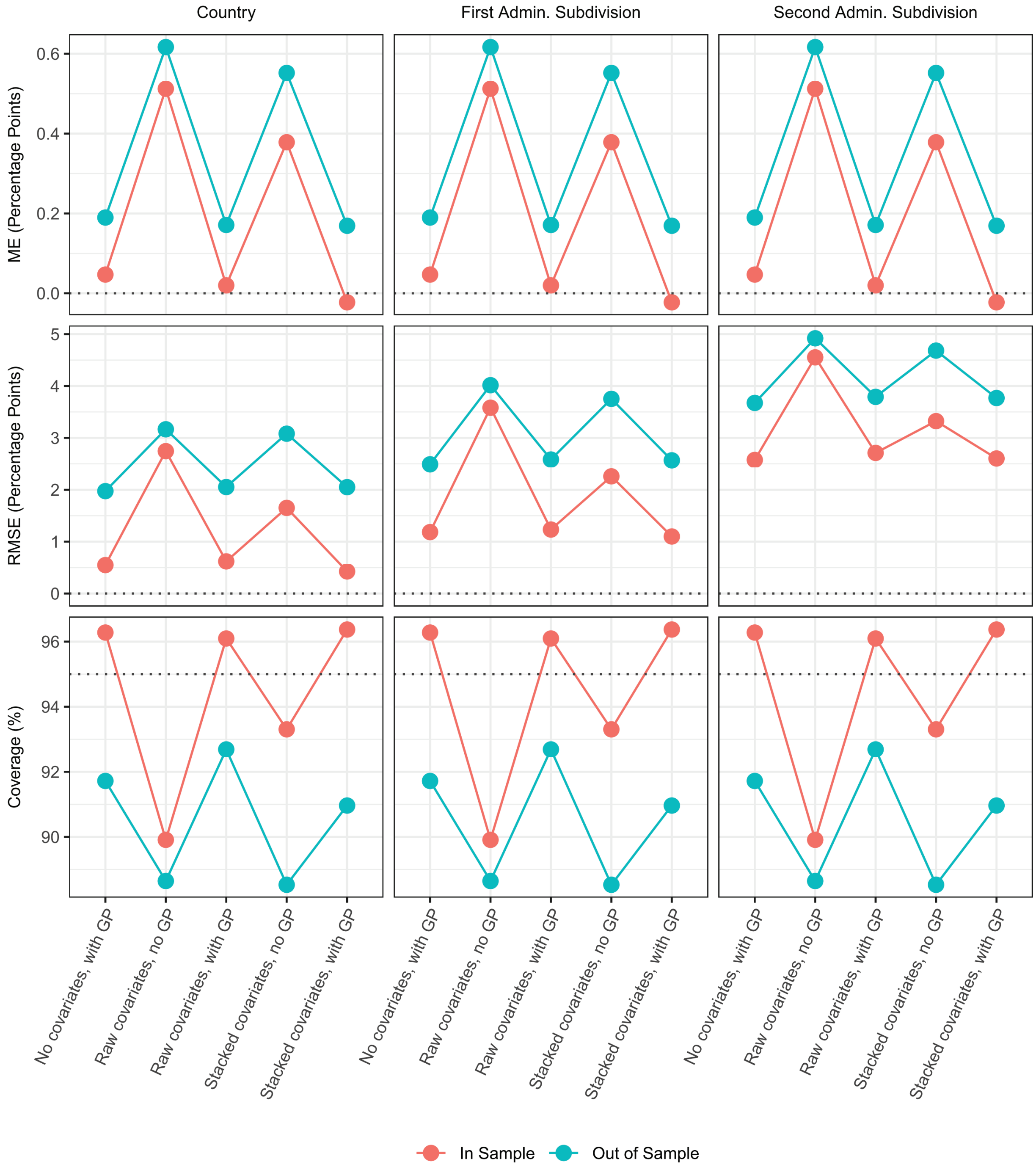

Supplementary Figure 20: Validation metrics from the covariate sensitivity analysis. Mean error (ME; first row), root-mean-square error (RMSE: second row), and 95% prediction interval coverage ('coverage'; third row), by level of aggregation. In- versus out-of-sample results are indicated by colour.

Supplementary Figure 21: Predictions comparison from the covariate sensitivity analysis

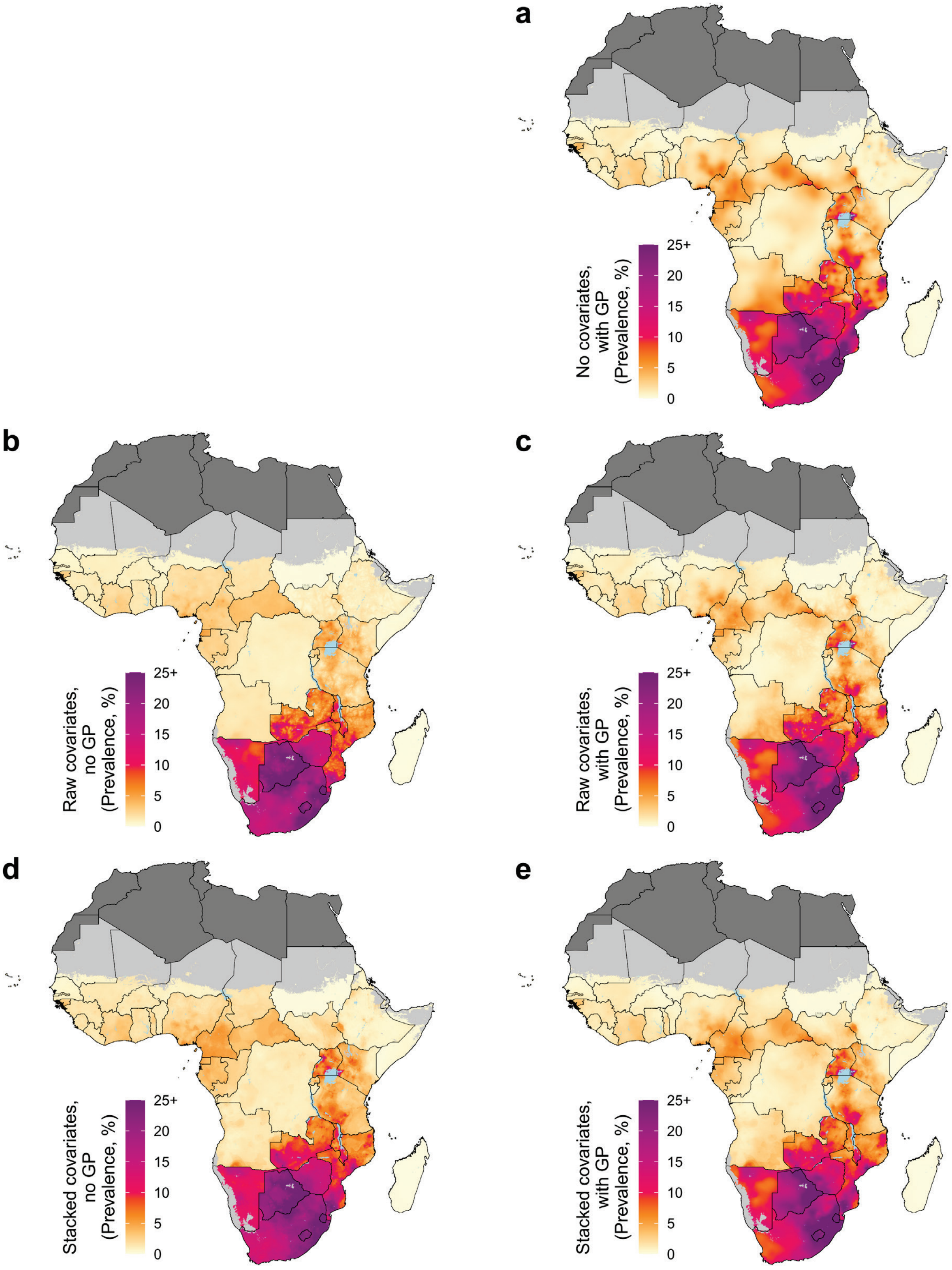

Supplementary Figure 21: Predictions comparison from the covariate sensitivity analysis. HIV prevalence among adults ages 15-49 in 2017 at the 5 x 5-km grid cell level based on models with no covariates and

including a Gaussian process (GP) (**a**), raw covariates with no GP (**b**), raw covariates with a GP (**c**), stacked covariates no a GP (**d**), and stacked covariates with a GP (**e**; the final model). Estimates are shown without calibration to GBD 2017 to better highlight the differences between the models. Maps reflect administrative boundaries, land cover, lakes, and population; grid cells with fewer than 10 people per 1 x 1-km and classified as “barren or sparsely vegetated” are coloured in light grey[29,31,32,64–66]. Countries in dark grey were not included in the analysis.

Supplementary Figure 22: Validation metrics from the ANC data and bias correction sensitivity analysis

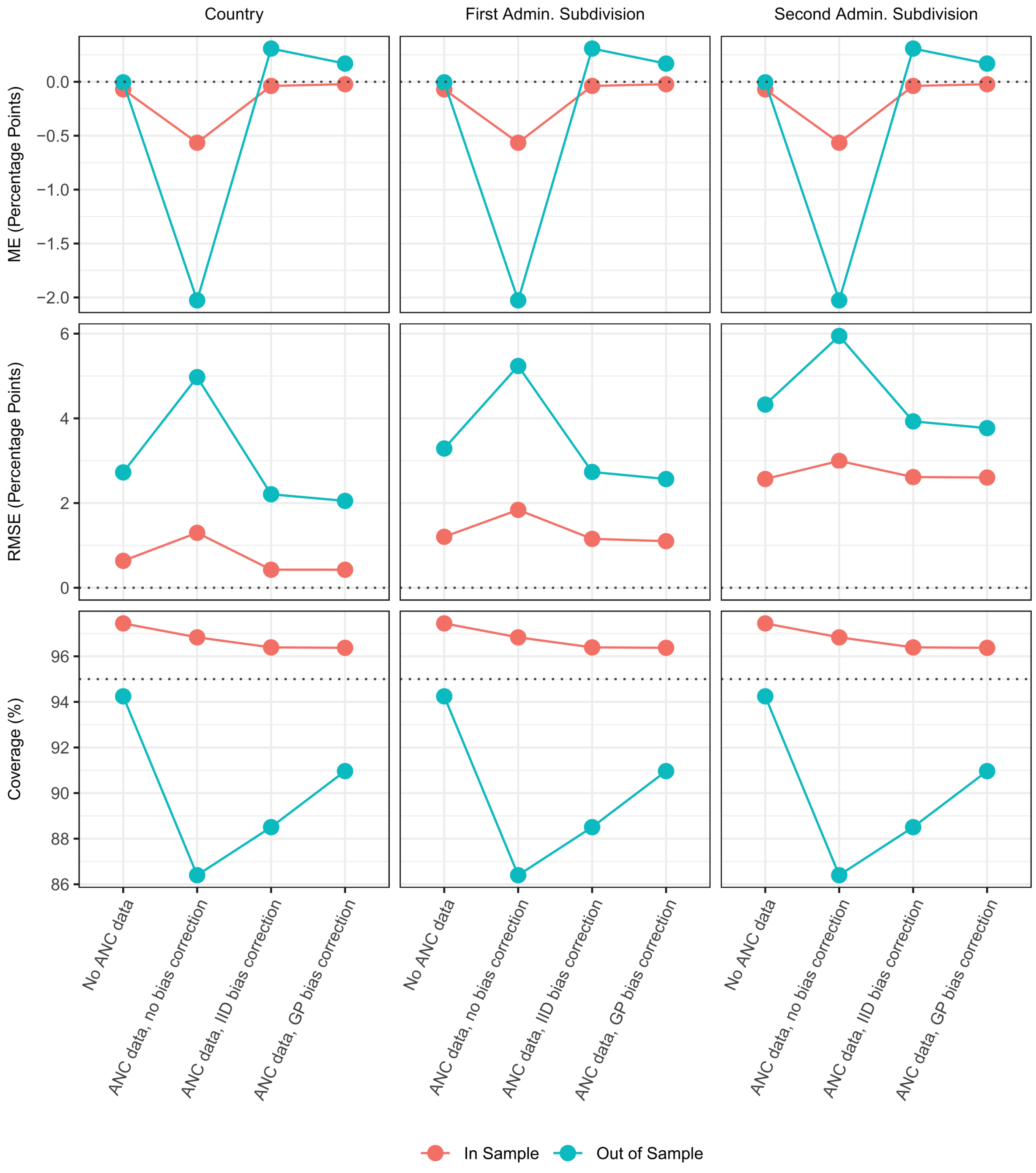

Supplementary Figure 22: Validation metrics from the ANC data and bias correction sensitivity analysis. Mean error (ME; first row), root-mean-square error (RMSE: second row), and 95% prediction interval coverage ('coverage'; third row), by level of aggregation. In- versus out-of-sample results are indicated by colour.

Supplementary Figure 23: Predictions comparison from the ANC data and bias correction sensitivity analysis

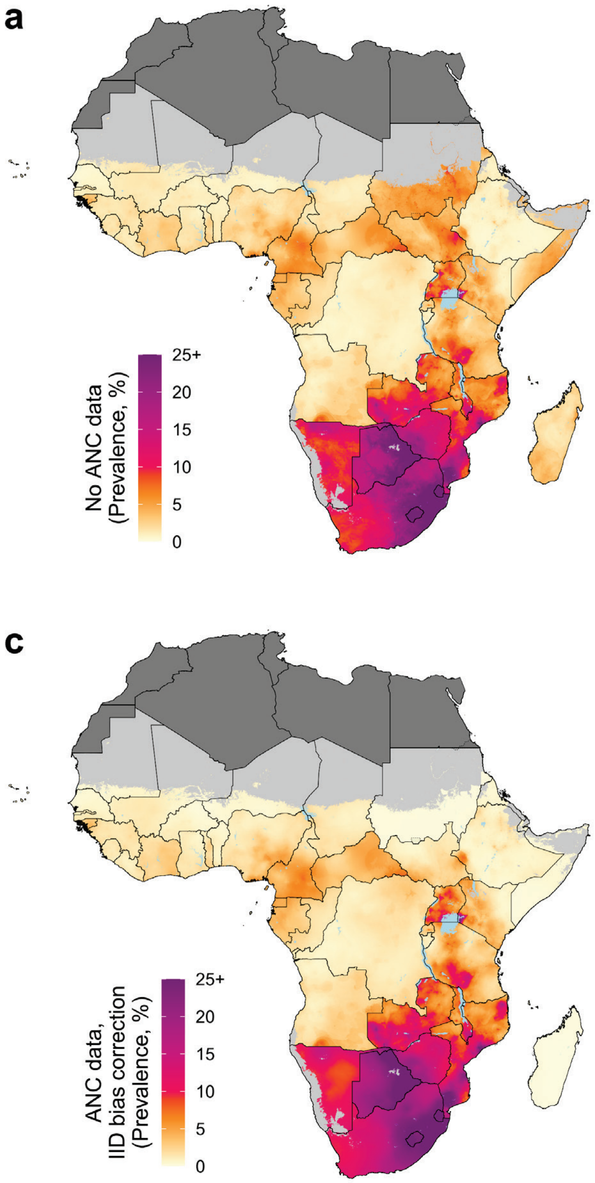

Supplementary Figure 23: Predictions comparison from the ANC data and bias correction sensitivity analysis. HIV prevalence among adults ages 15-49 in 2017 at the 5 x 5-km grid cell level based on models with no ANC data (**a**), including ANC data with no bias correction (**b**), including ANC data with an IID bias correction (**c**), and including ANC data with a GP bias correction (**d**; the final model). Estimates are shown without calibration to GBD 2017 to better highlight the differences between the models. Maps reflect administrative boundaries, land cover, lakes, and population; grid cells with fewer than 10 people per 1 x 1-km and classified as "barren or sparsely vegetated" are coloured in light grey[29,31,32,64–66]. Countries in dark grey were not included in the analysis.

Supplementary Figure 24: Validation metrics from the polygon data sensitivity analysis

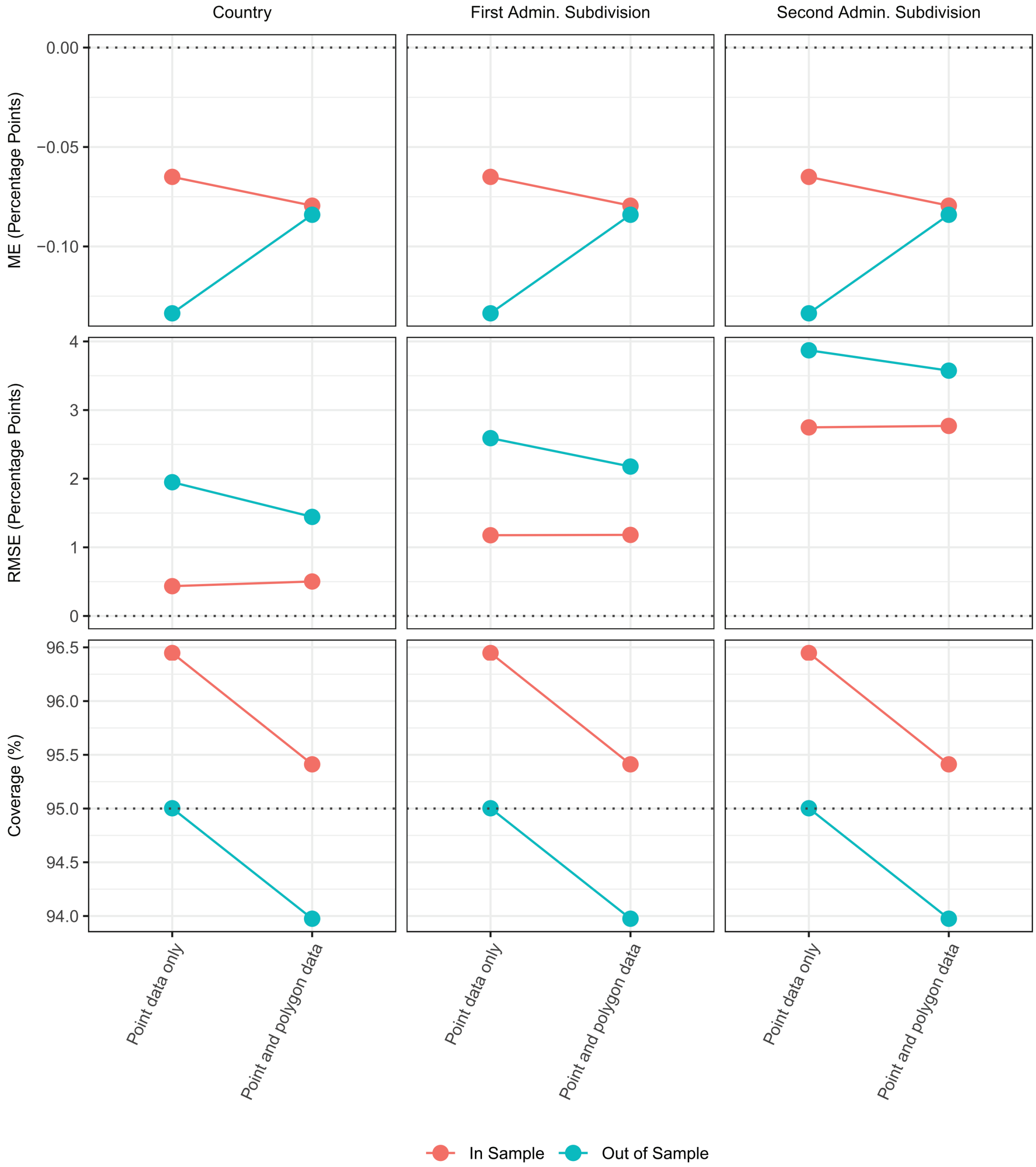

Supplementary Figure 24: Validation metrics from the polygon data sensitivity analysis. Mean error (ME; first row), root-mean-square error (RMSE: second row), and 95% prediction interval coverage ('coverage'; third row), by level of aggregation. In- versus out-of-sample results are indicated by colour.

Supplementary Figure 25: Predictions comparison from the polygon data analysis

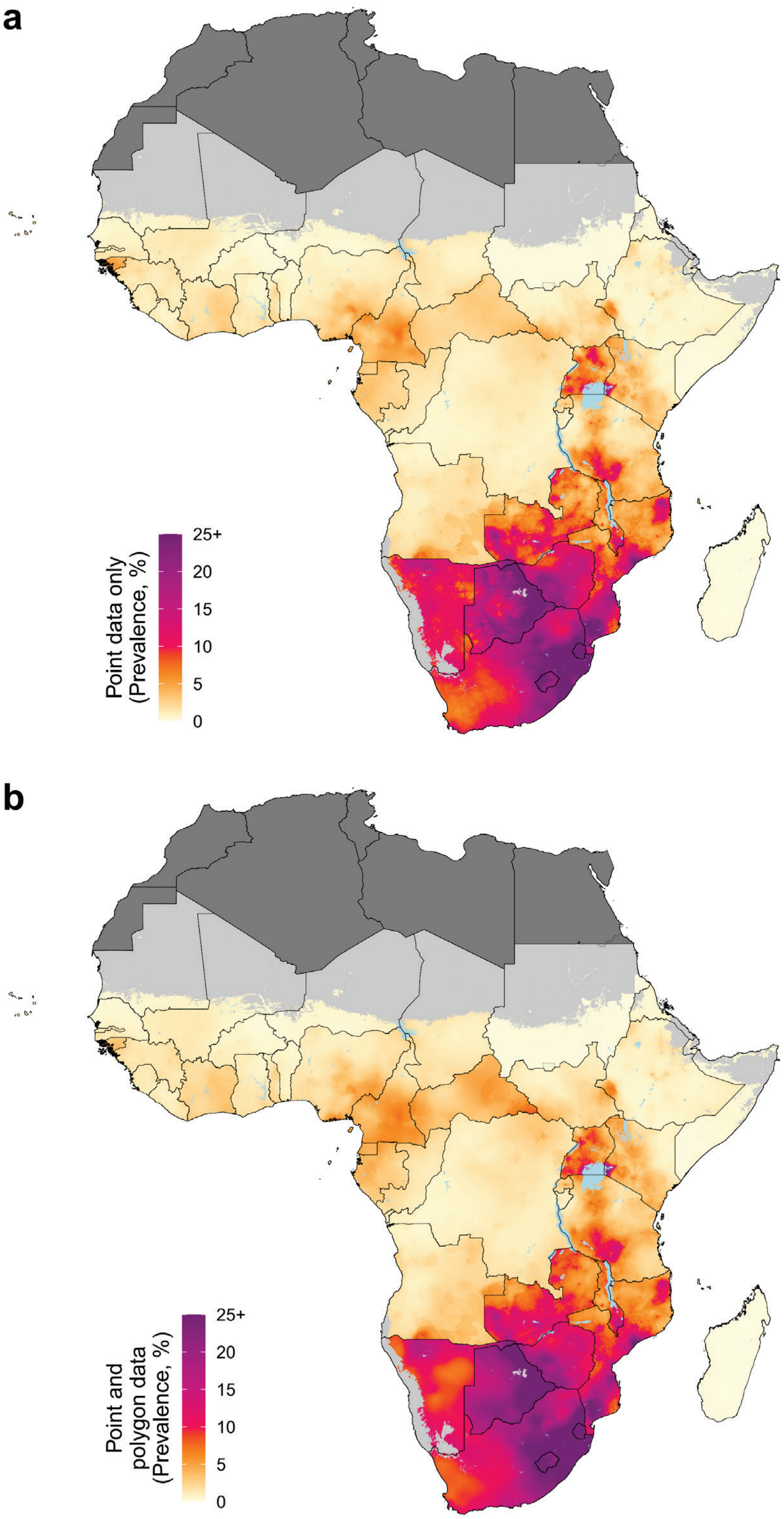

Supplementary Figure 25: Predictions comparison from the polygon data sensitivity analysis. HIV prevalence among adults ages 15-49 in 2017 at the 5 x 5-km grid cell level based on models including

point data only (**a**) and including both point and polygon data (**b**; the final model). Estimates are shown without calibration to GBD 2017 to better highlight the differences between the models. Maps reflect administrative boundaries, land cover, lakes, and population; grid cells with fewer than 10 people per 1 x 1-km and classified as “barren or sparsely vegetated” are coloured in light grey[29,31,32,64–66]. Countries in dark grey were not included in the analysis.

Supplementary Figure 26: HIV prevalence raking factors

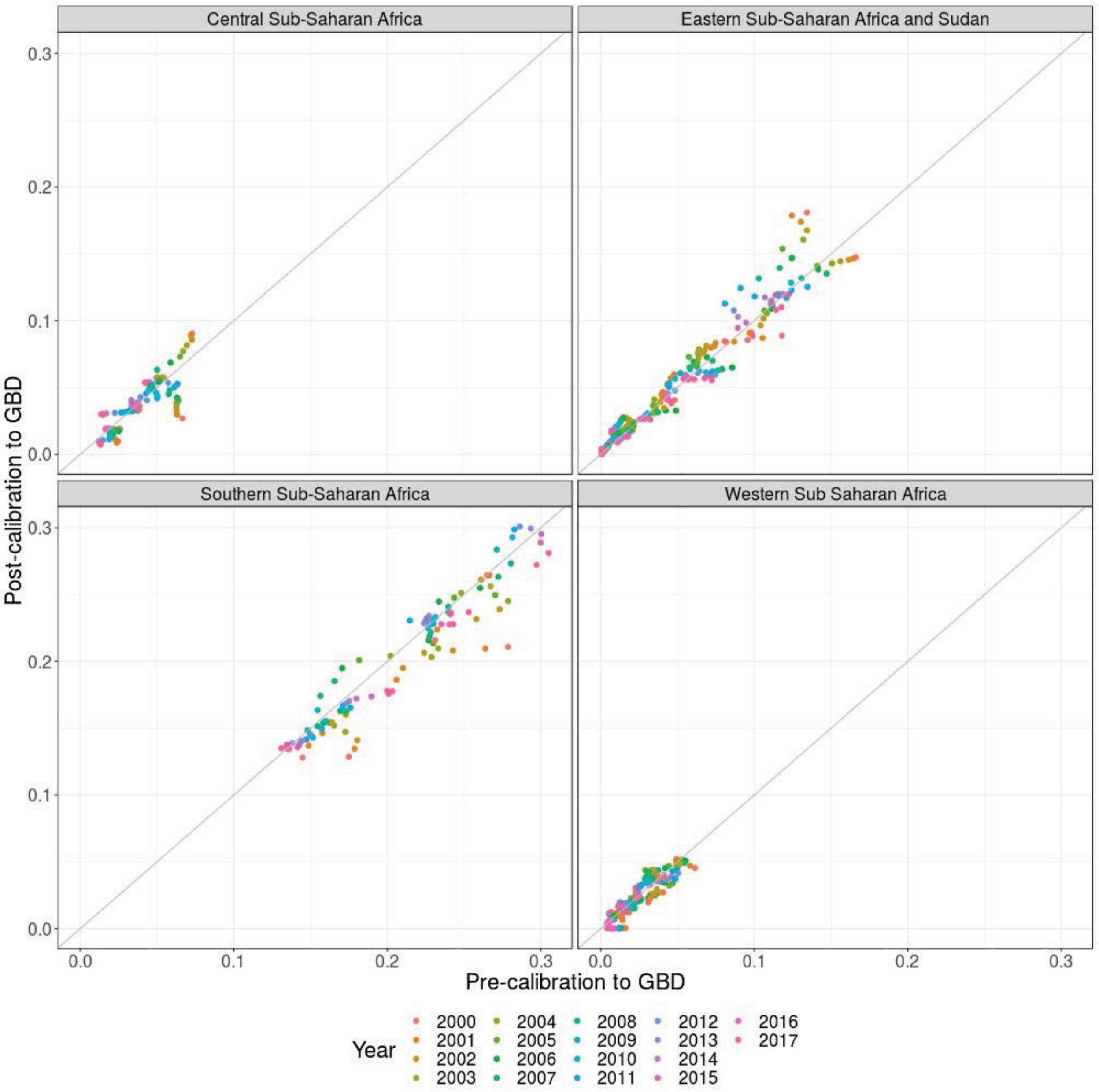

Supplementary Figure 26: HIV prevalence raking factors. Comparison of country-level HIV prevalence estimates derived by population-weighting 5 x 5-km grid cell estimates before (x-axis) and after (y-axis) calibration to GBD 2017 by year (colour) and country.

Supplementary Figure 27: Space mesh for geostatistical models

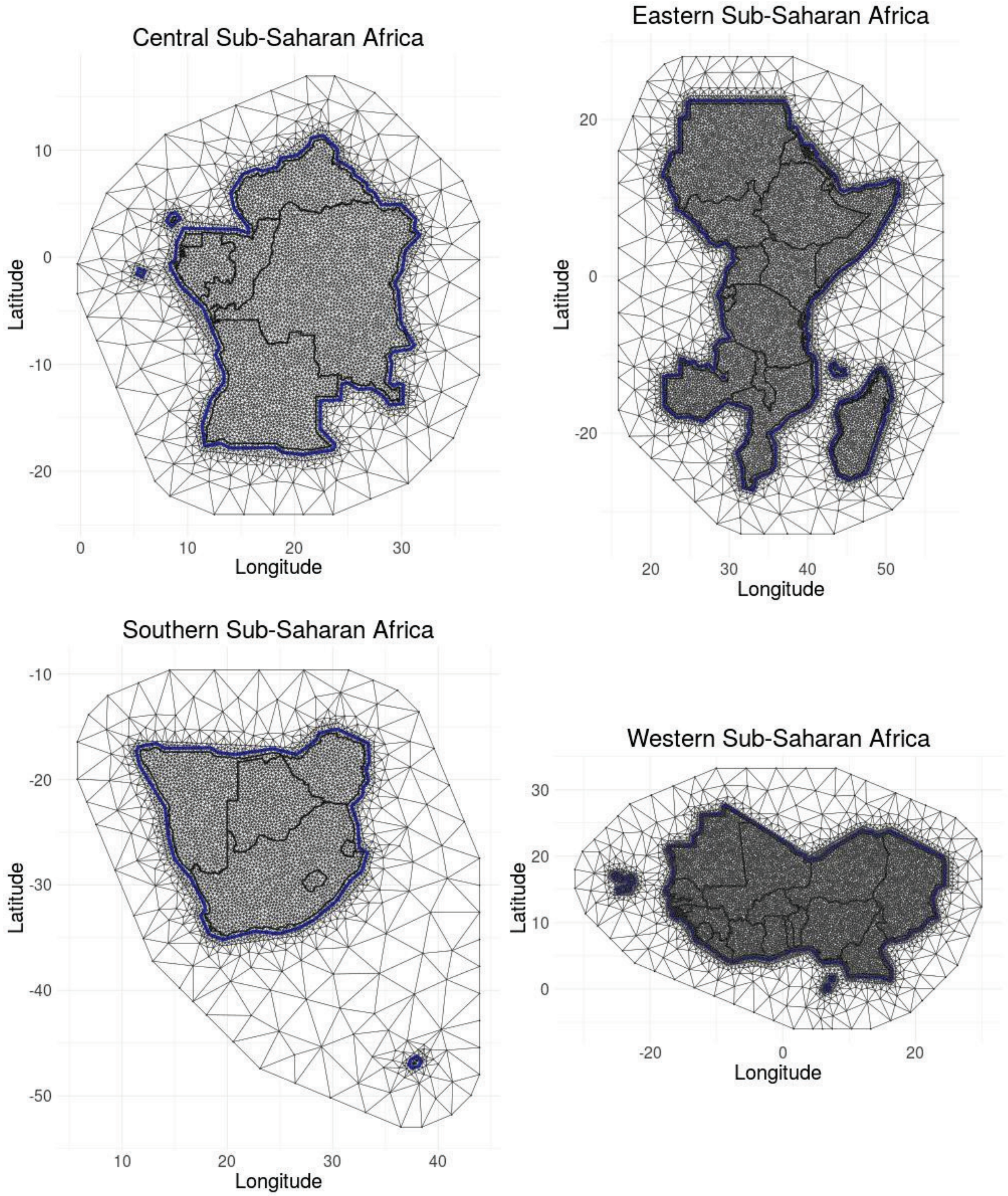

Supplementary Figure 27: Space mesh for geostatistical models. The finite elements mesh used to fit the space-time correlated error for each region overlaid on the countries in that region. Both the fine-scale mesh over land in the modelling region and the coarser buffer region mesh are shown. The simplified region polygon used to determine the boundary for the modelling region is shown in blue.

Supplementary Tables

Supplementary Table 1: Previous publications of subnational estimates of HIV prevalence in sub-Saharan Africa

Citation	Location(s)	Years estimated	Data source	Prediction resolution
Anderson, S.-J. et al. Maximising the effect of combination HIV prevention through prioritisation of the people and places in greatest need: a modelling study. The Lancet 384, 249–256 (2014).	Burundi, Ethiopia, Kenya, Lesotho, Malawi, Mozambique, Rwanda, Swaziland, Tanzania, Uganda, Zambia, Zimbabwe	2006-12 (pooled)	Most recent DHS in each country.	Grid (resolution not specified)
Barankanira, E., Molinari, N., Niyongabo, T. & Laurent, C. Spatial analysis of HIV infection and associated individual characteristics in Burundi: indications for effective prevention. BMC Public Health 16, (2015).	Burundi	2010	2010 DHS	Provinces (Admin. 1), Grid (resolution not specified)
Brdar, S., Gavrić, K., Ćulibrk, D. & Crnojević, V. Unveiling spatial epidemiology of HIV with mobile phone data. Sci. Rep. 6, (2016).	Ivory Coast	2012	2011-2012 DHS	Departments (Admin. 3)
Carrel, M. et al. Changing spatial patterns and increasing rurality of HIV prevalence in the Democratic Republic of the Congo between 2007 and 2013. Health Place 39, 79–85 (2016).	Democratic Republic of the Congo	2007, 2013	2007 DHS, 2013 DHS	Grid (resolution not specified)
Coburn, B. J. & Blower, S. Mapping HIV epidemics in sub-Saharan Africa with use of GPS data. Lancet Glob. Health 1, e251–e253 (2013).	1 healthcare district in Lesotho	2009-10 (pooled)	2009-10 DHS	100 x 100-m grid
Coburn, B. J., Okano, J. T. & Blower, S. Current drivers and geographic patterns of HIV in Lesotho: implications for treatment and prevention in Sub-Saharan Africa. BMC Med. 11, 224 (2013).	Lesotho	2009-10 (pooled)	2009-10 DHS	Ecological zones, Districts (Admin. 1)

Citation	Location(s)	Years estimated	Data source	Prediction resolution
Coburn, B. J., Okano, J. T. & Blower, S. Using geospatial mapping to design HIV elimination strategies for sub-Saharan Africa. Sci. Transl. Med. 9, eaag0019 (2017).	Lesotho	2009-10 (pooled)	2009-10 DHS	Grid (resolution not specified)
Cuadros, D. F. & Abu-Raddad, L. J. Spatial variability in HIV prevalence declines in several countries in sub-Saharan Africa. Health Place 28, 45–49 (2014).	Cameroon, Ethiopia, Kenya, Lesotho, Malawi, Mali, Rwanda, Senegal, Tanzania, Zimbabwe	2004, 2011 (Cameroon) ; 2005, 2011 (Ethiopia); 2003, 2008-09 (Kenya); 2004, 2009 (Lesotho); 2004, 2010 (Malawi); 2001, 2006 (Mali); 2005, 2010 (Rwanda); 2005, 2010-11 (Senegal); 2003-04, 2007-08, 2011-12 (Tanzania); 2005-06, 2010-11 (Zimbabwe)	DHS in Cameroon (2004, 2011), Ethiopia (2005, 2011), Kenya (2003, 2008-09), Lesotho (2004, 2009), Malawi (2004, 2010), Mali (2001, 2006), Rwanda (2005, 2010), Senegal (2005, 2010-11), Tanzania (2003-04, 2007-08, 2011-12), and Zimbabwe (2005-06, 2010-11)	Grid (resolution not specified)
Cuadros, D. F., Branscum, A. J., Miller, F. D., Awad, S. F. & Abu-Raddad, L. J. Are geographical “cold spots” of male circumcision driving differential HIV dynamics in Tanzania? Front. Public Health 3, (2015).	Tanzania	2003-04, 2007-08, and 2011-12	2003-04, 2007-08, and 2011-12 DHS	1 x 1-km grid
Cuadros, D. F. et al. Mapping the spatial variability of HIV infection in Sub-Saharan Africa: Effective information for localized HIV prevention and control. Sci. Rep. 7, 9093 (2017).	Kenya, Malawi, Mozambique, Tanzania	2008-09 (Kenya), 2010 (Malawi), 2009 (Mozambique), and 2011-2012 (Tanzania)	DHS in Kenya (2008-09), Malawi (2010), Mozambique (2009), and Tanzania (2011-12)	5 x 5-km grid

Citation	Location(s)	Years estimated	Data source	Prediction resolution
Cuadros, D. F. et al. Capturing the spatial variability of HIV epidemics in South Africa and Tanzania using routine healthcare facility data. Int. J. Health Geogr. 17, 27 (2018).	South Africa, Tanzania	2011 (Tanzania) and 2014 (South Africa)	South Africa: ACDIS demographic surveillance system 2014, district health information system (DHIS) 2014.<br><br>Tanzania: 2011 – 2012 Tanzania DHS. 2010 ANC data. Data from 132 healthcare facilities	Grid (resolution not specified)
Cuadros, D. F., Branscum, A. J. & Mukandavire, Z. Temporal stability of HIV prevalence in high-burden areas regardless of declines in national HIV prevalence in Malawi and Zimbabwe: AIDS 32, 1381–1383 (2018).	Malawi, Zimbabwe	2004, 2010, 2015-16 (Malawi); 2005-06, 2010-11, 2015 (Zimbabwe)	DHS in Malawi (2004, 2010, 2015-16) and Zimbabwe (2005-06, 2010-11, 2015)	Grid (resolution not specified)
Kalipeni, E. & Zulu, L. Using GIS to Model and Forecast HIV/AIDS Rates in Africa, 1986–2010. Prof. Geogr. 60, 33–53 (2008).	Africa	1986-2010	ANC sentinel surveillance from the U.S. Census Bureau HIV Surveillance Database (1985-2003)	1 x 1-km grid
Kandala, N.-B., Campbell, E. K., Rakgoasi, S. D., Madi-Segwagwe, B. C. & Fako, T. T. The geography of HIV/AIDS prevalence rates in Botswana. HIVAIDS Auckl. NZ 4, 95–102 (2012).	Botswana	2008	2008 Botswana AIDS Impact Survey III	Districts (Admin. 1)
Kayeyi, N., Fylkesnes, K., Michelo, C., Makasa, M. & Sandøy, I. Decline in HIV prevalence among young women in Zambia: national-level estimates of trends mask geographical and socio-demographic differences. PLOS ONE 7, e33652 (2012).	Zambia	1994-2008	2001-02 and 2007 DHS, 1994-2008 ANC sentinel surveillance	Provinces (Admin. 1) and urban vs rural

Citation	Location(s)	Years estimated	Data source	Prediction resolution
Kleinschmidt, I., Pettifor, A., Morris, N., MacPhail, C. & Rees, H. Geographic distribution of human immunodeficiency virus in South Africa. Am. J. Trop. Med. Hyg. 77, 1163–1169 (2007).	South Africa	2003	2003 South African national household youth (15-24) survey of HIV infection and sexual behavior	25 x 25-km grid
Larmarange, J. & Bendaud, V. HIV estimates at second subnational level from national population-based surveys. AIDS 28, S469–S476 (2014).	Burkina Faso, Burundi, Ivory Coast, Cameroon, Ethiopia, Gabon, Guinea, Haiti, Lesotho, Mozambique, Malawi, Rwanda, Senegal, Sierra Leone, Tanzania, Uganda, Zimbabwe	2008 (Sierra Leone), 2009 (Lesotho, Mozambique), 2010 (Burkina Faso, Burundi, Malawi), 2010-2011 pooled (Rwanda, Senegal, Zimbabwe), 2011 (Cameroon, Ethiopia, Uganda), 2011-2012 pooled (Ivory Coast, Tanzania), 2012 (Gabon, Haiti)	2010 Burkina Faso DHS MICS, 2010 Burundi DHS, 2011 – 2012 Ivory Coast DHS MICS, 2011 Cameroon DHS MICS, 2011 Ethiopia DHS, 2012 Gabon DHS, 2012 Guinea DHS MICS, 2012 Haiti EMMUS, 2009 Lesotho DHS, 2009 Mozambique INSIDA, 2010 Malawi DHS, 2010-2011 Rwanda DHS, 2010-2011 Senegal DHS MICS, 2008 Sierra Leone DHS, 2011-2012 Tanzania HMIS, 2011 Uganda AIS, and 2010-2011 Zimbabwe DHS	Grid (resolution not specified)
Larmarange, J., Vallo, R., Yaro, S., Msellati, P. & Méda, N. Methods for mapping regional trends of HIV prevalence from Demographic and Health Surveys (DHS). Cybergeo Eur. J. Geogr. (2011). doi:10.4000/cybergeo.24606	Burkina Faso	2003	2003 Burkina Faso DHS	Grid (resolution not specified)
Mahy, M. et al. Redefining the HIV epidemic in Nigeria: from national to state level. AIDS 28, S461–S467 (2014).	Nigeria	1990s-2013	1990s-2010 Nigeria ANC sentinel surveillance, 2007 and 2012 National HIV/AIDS	States (Admin. 1)

Citation	Location(s)	Years estimated	Data source	Prediction resolution
			Reproductive Health Surveys	
Manda, S., Masenyetse, L., Cai, B. & Meyer, R. Mapping HIV prevalence using population and antenatal sentinel-based HIV surveys: a multi-stage approach. Popul. Health Metr. 13, 22 (2015).	Malawi	2010	2010 DHS, 2010 ANC sentinel surveillance	Districts (Admin. 2)
Marra, G., Radice, R., Bärnighausen, T., Wood, S. & McGovern, M. A unified modeling approach to estimating HIV prevalence in Sub-Saharan African countries. (2015).	Zambia, Zimbabwe, Swaziland.	2007-08	DHS in Zambia (2007), Zimbabwe (2008), and Swaziland (2008)	Admin. 1
McGillen, J. B., Anderson, S.-J., Dybul, M. R. & Hallett, T. B. Optimum resource allocation to reduce HIV incidence across sub-Saharan Africa: a mathematical modelling study. Lancet HIV 3, e441–e448 (2016).	Benin, Botswana, Burkina Faso, Cameroon, Republic of the Congo, Democratic Republic of the Congo, Ethiopia, Kenya, Mali, Mozambique, Nigeria, Rwanda, Sierra Leone, South Africa, Swaziland, Tanzania, Zambia, Zimbabwe	1985, 1995, 2005, 2015, 2025	Most recent DHS, AIS, or other survey (Botswana and South Africa) in each country; and ANC sentinel surveillance for available years from UNAIDS Spectrum files.	Admin. 1
Messina, J. et al. Spatial and socio-behavioral patterns of HIV prevalence in the Democratic Republic of Congo. Soc. Sci. Med. 1982 71, 1428–1435 (2010).	Democratic Republic of the Congo	2007	2007 DHS	1 x 1-km
Ngesa, O., Mwambi, H. & Achia, T. Bayesian spatial semi-parametric modeling of HIV variation in Kenya. PLOS ONE 9, e103299 (2014).	Kenya	2007	2007 AIDS Indicator Survey	Counties (Admin. 1)
Niragire, F., Achia, T. N. O., Lyambabaje, A. & Ntaganira, J. Bayesian mapping of HIV infection among women of reproductive age in Rwanda. PLOS ONE 10, e0119944 (2015).	Rwanda	2010	2010 DHS	Districts (Admin. 2)

Citation	Location(s)	Years estimated	Data source	Prediction resolution
Ntirampeba, D., Neema, I. & Kazembe, L. N. Joint spatial modelling of disease risk using multiple sources: an application on HIV prevalence from antenatal sentinel and demographic and health surveys in Namibia. Glob. Health Res. Policy 2, (2017).	Namibia	2013-14	2013 DHS, 2014 ANC sentinel surveillance	Constituencies (Admin. 2), Health districts
Okano, J. T. & Blower, S. Sex-specific maps of HIV epidemics in sub-Saharan Africa. Lancet Infect. Dis. 16, 1320–1322 (2016).	Lesotho	2010	2010 DHS	Grid (resolution not specified)
Schaefer, R. et al. Spatial patterns of HIV prevalence and service use in East Zimbabwe: implications for future targeting of interventions. J. Int. AIDS Soc. 20, (2017).	Portions of three districts in Manicaland, Zimbabwe	2013	Manicaland HIV/ STD Prevention Project (2012-13)	Grid (resolution not specified)
Shaikh, N. et al. Masking through averages - intraprovincial heterogeneity in HIV prevalence within the Western Cape. S. Afr. Med. J. 96, 538 (2008).	Western Cape, South Africa	2001, 2004	ANC sentinel surveillance (2001, 2004)	Health districts
Tanser, F., Bärnighausen, T., Cooke, G. S. & Newell, M.-L. Localized spatial clustering of HIV infections in a widely disseminated rural South African epidemic. Int. J. Epidemiol. 38, 1008–1016 (2009).	Hlabisa sub-district, KwaZulu-Natal, South Africa	2003	2003-04 Africa Centre Demographic system (ACDIS) and HIV surveillance	30 x 30-m grid
Tanser, F., LeSueur, D., Solarsh, G. & Wilkinson, D. HIV heterogeneity and proximity of homestead to roads in rural South Africa: an exploration using a geographical information system. Trop. Med. Int. Health 5, 40–46 (2018).	1 health district in northern KwaZulu-Natal, South Africa	1997	1997 ANC sentinel surveillance	Clinic catchment areas
The Subnational Estimates Working Group of the HIV Modelling Consortium. Evaluation of geospatial methods to generate subnational HIV prevalence estimates for local level planning: AIDS 30, 1467–1474 (2016).	Kenya, Malawi, Tanzania,	2008 – 2009 pooled (Kenya), 2010 (Malawi), 2012 (Tanzania)	2008-2009 Kenya DHS, 2010 Malawi DHS, 2012 Tanzania DHS	Grid (resolution not specified), Malawi Districts (Admin. 2)
Wabiri, N., Shisana, O., Zuma, K. & Freeman, J. Assessing the spatial nonstationarity in relationship between local patterns of HIV infections and the covariates in South Africa: A geographically weighted regression analysis. Spat. Spatio-Temporal Epidemiol. 16, 88–99 (2016).	South Africa	2008	2008 South African National HIV prevalence, Incidence, Behavior and Communication Survey	District municipalities (Admin. 2)

Citation	Location(s)	Years estimated	Data source	Prediction resolution
Wand, H., Whitaker, C. & Ramjee, G. Geoadditive models to assess spatial variation of HIV infections among women in Local communities of Durban, South Africa. Int. J. Health Geogr. 10, 28 (2011).	eThekwini Metropolitan Municipality, South Africa	2003-06 (pooled)	2003-06 Methods for Improving Reproductive Health in Africa (MIRA) clinical trial	Grid (resolution not specified)
Zulu, L. C., Kalipeni, E. & Johannes, E. Analyzing spatial clustering and the spatiotemporal nature and trends of HIV/AIDS prevalence using GIS: the case of Malawi, 1994-2010. BMC Infect. Dis. 14, (2014).	Malawi	1994-2010	ANC sentinel surveillance (1994, 1996, 1999, 2001, 2003, 2005, 2007, 2010)	1 x 1-km grid

Supplementary Table 2: HIV seroprevalence survey data

Country	Years	Name	Type	Geographic Detail	Citation	NID*
Angola	2015-16	Angola Demographic and Health Survey 2015-2016	Survey microdata	GPS	ICF International, Ministry of Health (Angola), National Institute of Statistics (Angola), United Nations Children's Fund (UNICEF). Angola Demographic and Health Survey 2015-2016. Fairfax, United States: ICF International, 2017.	218555
Benin	2006	Benin Demographic and Health Survey 2006	Survey report	Admin 1	Macro International, Inc, National Institute of Statistics and Economic Analysis (INSAE) (Benin), National Program Against AIDS (PNLS) (Benin). Benin Demographic and Health Survey 2006. Fairfax, United States: ICF International.	18959
Benin	2012	Benin Demographic and Health Survey 2011-2012	Survey report	Admin 1	ICF International, National Institute of Statistics and Economic Analysis (INSAE) (Benin), National Program Against AIDS (PNLS) (Benin). Benin Demographic and Health Survey 2011-2012. Fairfax, United States: ICF International, 2014.	79839
Botswana	2004	Botswana AIDS Impact Survey 2004	Survey microdata	GPS	Central Statistics Office (Botswana). Botswana AIDS Impact Survey 2004. Gaborone, Botswana: Statistics Botswana.	22114
Botswana	2008	Botswana AIDS Impact Survey 2008	Survey microdata	GPS	Central Statistics Office (Botswana), National AIDS Coordinating Agency (Botswana). Botswana AIDS Impact Survey 2008. Gaborone, Botswana: Statistics Botswana, 2015.	22116
Botswana	2013	Botswana AIDS Impact Survey 2013	Survey microdata	GPS	Ministry of Health (Botswana), National AIDS Coordinating Agency (Botswana), Statistics Botswana. Botswana AIDS Impact Survey 2013. Gaborone, Botswana: Statistics Botswana, 2015.	134753
Burkina Faso	2000	Epidemiology of HIV	Literature review	Admin 3	Lagarde E, Congo Z, Meda N, Baya B, Yaro S, Sangli G,	127572

Country	Years	Name	Type	Geographic Detail	Citation	NID*
		infection in urban Burkina Faso			Traoré Y, Van Renthergem H, Caraël M, Study Group on HIV Dynamic Among Young Adults in Burkina Faso. Epidemiology of HIV infection in urban Burkina Faso. Int J STD AIDS. 2004; 15(6): 395-402.	
Burkina Faso	2003	Burkina Faso Demographic and Health Survey 2003	Survey microdata	GPS	Macro International, Inc, National Institute of Statistics and Demography (Burkina Faso). Burkina Faso Demographic and Health Survey 2003. Fairfax, United States: ICF International.	19088
Burkina Faso	2010	Burkina Faso Demographic and Health Survey 2010-2011	Survey microdata	GPS	ICF Macro, Ministry of Health (Burkina Faso), National Institute of Statistics and Demography (Burkina Faso). Burkina Faso Demographic and Health Survey 2010-2011. Fairfax, United States: ICF International.	19133
Burundi	2002	Burundi National HIV Infection Seroprevalence Survey 2001	Survey report	Admin 1	Training Center for Medical and Infectious Diseases (CEFORMI) (Burundi). Burundi National HIV Infection Seroprevalence Survey 2001.	150378
Burundi	2007	Burundi Combined HIV/AIDS/STI Behavioral Surveillance and HIV/AIDS Seroprevalence Survey 2007	Survey report	Admin 1	National Council for the Fight Against AIDS (CNLS) (Burundi). Burundi Combined HIV/AIDS/STI Behavioral Surveillance and HIV/AIDS Seroprevalence Survey 2007.	335814†
Burundi	2010-11	Burundi Demographic and Health Survey 2010-2011	Survey microdata	GPS	Burundi Institute of Statistics and Economic Studies, ICF International, Ministry of Public Health and the Fight Against AIDS (Burundi). Burundi Demographic and Health Survey 2010-2011. Fairfax, United States: ICF International, 2012.	30431
Burundi	2016-17	Burundi Demographic and Health Survey 2016-2017	Survey microdata	GPS	Burundi Institute of Statistics and Economic Studies, ICF International, Ministry of Public Health and the Fight Against AIDS (Burundi). Burundi Demographic and	286766

Country	Years	Name	Type	Geographic Detail	Citation	NID*
					Health Survey 2016-2017. Fairfax, United States: ICF International, 2018.	
Cameroon	2004	Cameroon Demographic and Health Survey 2004	Survey microdata	GPS	Macro International, Inc, National Institute of Statistics (Cameroon). Cameroon Demographic and Health Survey 2004. Fairfax, United States: ICF International.	19211
Cameroon	2011	Cameroon Demographic and Health Survey 2011	Survey microdata	GPS	ICF International, Ministry of Economy, Planning and Regional Development (Cameroon), Ministry of Public Health (Cameroon), National Institute of Statistics (Cameroon), Pasteur Center of Cameroon. Cameroon Demographic and Health Survey 2011. Fairfax, United States: ICF International.	19274
Cameroon	2017	Cameroon Population-based HIV Impact Assessment 2017-2018	Survey report	Admin >2	Ministry of Health, Cameroon, Centers for Disease Control and Prevention (CDC), and ICAP at Columbia University. Cameroon Population-based HIV Impact Assessment (CAMPHIA) 2015-16: Summary Sheet. Yaoundé, Cameroon, Atlanta, Georgia and New York, New York, USA: Ministry of Health, CDC and ICAP. July 2018.	358376
Cape Verde	2005	Cape Verde Demographic and Health Survey 2005	Survey report	Admin 1	Macro International, Inc, Ministry of Health (Cape Verde), National Institute of Statistics (Cape Verde). Cape Verde Demographic and Health Survey 2005.	21442
Central African Republic	2006	Central African Republic Multiple Indicator Cluster Survey 2006	Survey report	Admin 1	United Nations Children's Fund (UNICEF). Central African Republic Multiple Indicator Cluster Survey 2006. New York, United States: United Nations Children's Fund (UNICEF).	2223
Central African Republic	2010	Central African Republic Multiple Indicator	Survey report	Admin 1	Central African Institute of Statistics, Economic and Social Studies (ICASEES) (Central African Republic), ICF International. Central African	82832

Country	Years	Name	Type	Geographic Detail	Citation	NID*
		Cluster Survey 2010-2011			Republic Multiple Indicator Cluster Survey 2010-2011. Fairfax, United States: ICF International, 2013.	
Chad	2005	Chad National HIV/AIDS Seroprevalence Survey 2005	Survey report	Admin >1	Conseil Santé (France), National Institute for Statistics, Economic and Demographic Studies (INSEED) (Chad). Chad National HIV/AIDS Seroprevalence Survey 2005.	2248†
Chad	2014-15	Chad Demographic and Health Survey 2014-2015	Survey microdata	GPS	ICF International, National Institute of Statistical, Economic and Demographic Studies (Chad). Chad Demographic and Health Survey 2014-2015. Fairfax, United States: ICF International, 2016.	157025
Congo	2009	Congo AIDS Indicator Survey 2009	Survey microdata	Admin 1	ICF Macro, National Center for Statistics and Economic Studies (Congo, Rep.). Congo AIDS Indicator Survey 2009. Fairfax, United States: ICF International.	3133
Congo, Niger	2002-03	HIV and AIDS Surveillance Database Version July 2017	Survey report	Admin 1	United States Census Bureau. HIV and AIDS Surveillance Database Version July 2017. Washington, D.C., United States: United States Census Bureau, 2017.	313360
Cote d'Ivoire	2005	Côte d'Ivoire AIDS Indicator Survey 2005	Survey microdata	Admin >1	CDC Retro-CI, Ministry of the Fight Against AIDS (Côte d'Ivoire), National Institute of Statistics (Côte d'Ivoire), ORC Macro. Côte d'Ivoire AIDS Indicator Survey 2005. Fairfax, United States: ICF International.	56148
Cote d'Ivoire	2011-12	Côte d'Ivoire Demographic and Health Survey 2011-2012	Survey microdata	GPS	ICF International, Ministry of the Fight Against AIDS (Côte d'Ivoire), National Institute of Statistics (Côte d'Ivoire). Côte d'Ivoire Demographic and Health Survey 2011-2012. Fairfax, United States: ICF International.	18533
Cote d'Ivoire	2017	Côte d'Ivoire Population-based HIV Impact	Survey report	Admin 1	Ministry of Health and Public Hygiene, Cote d'Ivoire, Centers for Disease Control and Prevention (CDC), and ICAP at	365441

Country	Years	Name	Type	Geographic Detail	Citation	NID*
		Assessment 2017-2018			Columbia University. Cote d'Ivoire Population-based HIV Impact Assessment (CIPHIA) 2015-16: Summary Sheet. Abidjan, Cote d'Ivoire, Atlanta, Georgia and New York, New York, USA: Ministry of Health, CDC and ICAP. July 2018.	
Democratic Republic of the Congo	2007	Democratic Republic of the Congo Demographic and Health Survey 2007	Survey microdata	GPS	Macro International, Inc, Ministry of Planning (Congo, DR). Democratic Republic of the Congo Demographic and Health Survey 2007. Fairfax, United States: ICF International.	19381
Democratic Republic of the Congo	2013-14	Democratic Republic of the Congo Demographic and Health Survey 2013-2014	Survey microdata	GPS	ICF International, Ministry of Planning and Monitoring Implementation of the Revolution of Modernity (Congo, DR), Ministry of Public Health (Congo, DR), National Institute of Statistics (Congo, DR). Democratic Republic of the Congo Demographic and Health Survey 2013-2014. Fairfax, United States: ICF International, 2014.	76878
Equatorial Guinea	2011	Equatorial Guinea Demographic and Health Survey 2011	Survey report	Admin 1	ICF International, Ministry of Health and Social Welfare (Equatorial Guinea), Ministry of Planning, Economic Development and Public Investment (Equatorial Guinea). Equatorial Guinea Demographic and Health Survey 2011. Fairfax, United States: ICF International, 2012.	76884
Eritrea	2010	Eritrea Population and Health Survey 2010	Survey report	Admin 1	Kenya Medical Research Institute (KEMRI), National Statistics Office (Eritrea), The Fafo Research Foundation. Eritrea Population and Health Survey 2010.	249999
Ethiopia	2005	Ethiopia Demographic and Health Survey 2005	Survey microdata	GPS	Macro International, Inc, Population and Housing Census Commissions Office (PHCCO). Ethiopia Demographic and Health Survey 2005. Fairfax, United States: ICF International.	19557

Country	Years	Name	Type	Geographic Detail	Citation	NID*
Ethiopia	2011-12	Ethiopia Demographic and Health Survey 2010-2011	Survey microdata	GPS	Central Statistical Agency (Ethiopia), ICF Macro, Ministry of Health (Ethiopia). Ethiopia Demographic and Health Survey 2010-2011. Fairfax, United States: ICF International.	21301
Ethiopia	2016	Ethiopia Demographic and Health Survey 2016	Survey microdata	GPS	Central Statistical Agency (Ethiopia), ICF International. Ethiopia Demographic and Health Survey 2016. Fairfax, United States: ICF International, 2017.	218568
Gabon	2012	Gabon Demographic and Health Survey 2012	Survey microdata	GPS	General Directorate of Statistics (Gabon), ICF International, Ministry of Economy, Employment and Sustainable Development (Gabon), Ministry of Health (Gabon). Gabon Demographic and Health Survey 2012. Fairfax, United States: ICF International, 2013.	76706
Ghana	2003	Ghana Demographic and Health Survey 2003	Survey microdata	GPS	Ghana Statistical Service, Macro International, Inc. Ghana Demographic and Health Survey 2003. Fairfax, United States: ICF International.	19627
Ghana	2014	Ghana Demographic and Health Survey 2014	Survey microdata	GPS	Ghana Health Service, Ghana Statistical Service, ICF International. Ghana Demographic and Health Survey 2014. Fairfax, United States: ICF International, 2016.	157027
Guinea	2005	Guinea Demographic and Health Survey 2005	Survey microdata	GPS	Macro International, Inc, National Statistics Directorate (Guinea). Guinea Demographic and Health Survey 2005. Fairfax, United States: ICF International.	19683
Guinea	2012	Guinea Demographic and Health Survey 2012	Survey microdata	GPS	ICF Macro, Ministry of Health and Public Hygiene (Guinea), National Institute of Statistics (Guinea). Guinea Demographic and Health Survey 2012. Fairfax, United States: ICF International.	69761
Guinea-Bissau	2005	Changes in prevalence	Literature review	Site	da Silva ZJ, Oliveira I, Andersen A, Dias F, Rodrigues A,	353416

Country	Years	Name	Type	Geographic Detail	Citation	NID*
		and incidence of HIV-1, HIV-2 and dual infections in urban areas of Bissau, Guinea-Bissau: is HIV-2 disappearing?			Holmgren B, Andersson S, Aaby P. Changes in prevalence and incidence of HIV-1, HIV-2 and dual infections in urban areas of Bissau, Guinea-Bissau: is HIV-2 disappearing?. AIDS. 2008; 22(10): 1195-202.	
Guinea-Bissau	2006	Two distinct epidemics: the rise of HIV-1 and decline of HIV-2 infection between 1990 and 2007 in rural Guinea-Bissau	Literature review	Admin 2	Tienen Cv, van der Loeff MS, Zaman SM, Vincent T, Sarge-Njie R, Peterson I, Leligdowicz A, Jaye A, Rowland-Jones S, Aaby P, Whittle H. Two distinct epidemics: the rise of HIV-1 and decline of HIV-2 infection between 1990 and 2007 in rural Guinea-Bissau. J Acquir Immune Defic Syndr. 2010; 53(5): 640-7.	353418
Guinea-Bissau	2010	Guinea-Bissau HIV Prevalence Survey 2010	Survey report	Admin 1	Institute of Hygiene and Tropical Medicine, Nova University of Lisbon (Portugal). Guinea-Bissau HIV Prevalence Survey 2010.	340936
Kenya	2003	HIV prevalence and associated risk factors among individuals aged 13-34 years in Rural Western Kenya	Literature review	City	Amornkul PN, Vandenhoudt H, Nasokho P, Odhiambo F, Mwaengo D, Hightower A, Buvé A, Misore A, Vulule J, Vitek C, Glynn J, Greenberg A, Slutsker L, De Cock KM. HIV prevalence and associated risk factors among individuals aged 13-34 years in Rural Western Kenya. PLoS One. 2009; 4(7): e6470.	222209
Kenya	2003	Kenya Demographic and Health Survey 2003	Survey microdata	GPS	Centers for Disease Control and Prevention (CDC), Central Bureau of Statistics (Kenya), Macro International, Inc, Ministry of Health (Kenya), National Council for Population and Development (Kenya). Kenya Demographic and Health Survey 2003. Fairfax, United States: ICF International.	20145
Kenya	2006	Association of attitudes and beliefs	Literature review	Admin 2	Cohen CR, Montandon M, Carrico AW, Shiboski S, Bostrom A, Obure A, Kwena Z,	353420

Country	Years	Name	Type	Geographic Detail	Citation	NID*
		towards antiretroviral therapy with HIV-seroprevalence in the general population of Kisumu, Kenya			Bailey RC, Nguti R, Bukusi EA. Association of attitudes and beliefs towards antiretroviral therapy with HIV-seroprevalence in the general population of Kisumu, Kenya. PLoS One. 2009; 4(3): e4573.	
Kenya	2006	The effect of participant nonresponse on HIV prevalence estimates in a population-based survey in two informal settlements in Nairobi city	Literature review	Site	Ziraba AK, Madise NJ, Matilu M, Zulu E, Kebaso J, Khamadi S, Okoth V, Ezeh AC. The effect of participant nonresponse on HIV prevalence estimates in a population-based survey in two informal settlements in Nairobi city. Popul Health Metr. 2010; 8: 22.	353424
Kenya	2007	Kenya AIDS Indicator Survey 2007	Survey microdata	GPS	Centers for Disease Control and Prevention (CDC), Kenya Medical Research Institute (KEMRI), Kenya National Bureau of Statistics, Ministry of Public Health and Sanitation (Kenya), National AIDS Control Council (Kenya), National AIDS and STI Control Program (Kenya), National Coordinating Agency for Population and Development (Kenya), National Public Health Laboratory Services, Ministry of Public Health and Sanitation (Kenya), United States Agency for International Development (USAID). Kenya AIDS Indicator Survey 2007. Nairobi, Kenya: Kenya National Bureau of Statistics.	133219
Kenya	2008-09	Kenya Demographic and Health Survey 2008-2009	Survey microdata	GPS	ICF Macro, Kenya Medical Research Institute (KEMRI), Kenya National Bureau of Statistics, Ministry of Public Health and Sanitation (Kenya), National AIDS and STI Control Program (Kenya), National Aids Control Council (NACC),	21365

Country	Years	Name	Type	Geographic Detail	Citation	NID*
					National Coordinating Agency for Population and Development (Kenya). Kenya Demographic and Health Survey 2008-2009. Fairfax, United States: ICF International.	
Kenya	2012	Cascade of HIV care and population viral suppression in a high-burden region of Kenya	Literature review	Town	Maman D, Zeh C, Mukui I, Kirubi B, Masson S, Opolo V, Szumilin E, Riche B, Etard JF. Cascade of HIV care and population viral suppression in a high-burden region of Kenya. AIDS. 2015; 29(12): 1557-65.	353428
Kenya	2013	Kenya AIDS Indicator Survey 2012-2013	Survey microdata	GPS	Kenya National Bureau of Statistics, Ministry of Devolution and Planning (Kenya), Ministry of Health (Kenya), National AIDS and STI Control Program (Kenya). Kenya AIDS Indicator Survey 2012-2013. Nairobi, Kenya: Kenya National Bureau of Statistics.	133304
Kenya, Uganda	2013	Population levels and geographical distribution of HIV RNA in rural Ugandan and Kenyan communities, including serodiscordant couples: a cross-sectional analysis	Literature review	Town	Jain V, Petersen ML, Liegler T, Byonanebye DM, Kwarisiima D, Chamie G, Sang N, Black D, Clark TD, Ladai A, Plenty A, Kabami J, Ssemmondo E, Bukusi EA, Cohen CR, Charlebois ED, Kamya MR, Havlir DV, SEARCH Collaboration. Population levels and geographical distribution of HIV RNA in rural Ugandan and Kenyan communities, including serodiscordant couples: a cross-sectional analysis. Lancet HIV. 2017; 4(3): e122-33.	332689
Lesotho	2004-05	Lesotho Demographic and Health Survey 2004-2005	Survey microdata	GPS	Bureau of Statistics (Lesotho), Macro International, Inc, Ministry of Health and Social Welfare (Lesotho). Lesotho Demographic and Health Survey 2004-2005. Fairfax, United States: ICF International.	20167

Country	Years	Name	Type	Geographic Detail	Citation	NID*
Lesotho	2009-10	Lesotho Demographic and Health Survey 2009-2010	Survey microdata	GPS	ICF Macro, Ministry of Health and Social Welfare (Lesotho). Lesotho Demographic and Health Survey 2009-2010. Fairfax, United States: ICF International.	21382
Lesotho	2014	Lesotho Demographic and Health Survey 2014	Survey microdata	GPS	ICF International, Ministry of Health and Social Welfare (Lesotho). Lesotho Demographic and Health Survey 2014. Fairfax, United States: ICF International.	157058
Lesotho	2017	Lesotho Population-based HIV Impact Assessment 2016-2017	Survey report	Admin 1	Ministry of Health, Lesotho, Centers for Disease Control and Prevention (CDC), and ICAP at Columbia University. Lesotho Population-based HIV Impact Assessment (LePHIA) 2015-16: Summary Sheet. Maseru, Lesotho, Atlanta, Georgia and New York, New York, USA: Ministry of Health, CDC and ICAP. November 2017.	327582
Liberia	2006-07	Liberia Demographic and Health Survey 2006-2007	Survey microdata	GPS	Liberia Institute for Statistics and Geo-information Services (LISGIS), Macro International, Inc. Liberia Demographic and Health Survey 2006-2007. Fairfax, United States: ICF International.	20191
Liberia	2013	Liberia Demographic and Health Survey 2013	Survey microdata	GPS	ICF International, Liberia Institute for Statistics and Geo-information Services (LISGIS), National AIDS and STI Control Program (NACP), Ministry of Health and Social Welfare (Liberia). Liberia Demographic and Health Survey 2013. Fairfax, United States: ICF International.	77385
Malawi	2004-05	Malawi Demographic and Health Survey 2004-2005	Survey microdata	GPS	Macro International, Inc, National Statistical Office of Malawi. Malawi Demographic and Health Survey 2004-2005. Fairfax, United States: ICF International.	20263
Malawi	2005-09	Underestimation of HIV prevalence in	Literature review	Site	Floyd S, Molesworth A, Dube A, Crampin AC, Houben R, Chihana M, Price A, Kayuni N,	353430

Country	Years	Name	Type	Geographic Detail	Citation	NID*
		surveys when some people already know their status, and ways to reduce the bias			Saul J, French N, Glynn JR. Underestimation of HIV prevalence in surveys when some people already know their status, and ways to reduce the bias. AIDS. 2013; 27(2): 233-42.	
Malawi	2006	Sexual network structure and the spread of HIV in Africa: evidence from Likoma Island, Malawi	Literature review	Village	Helleringer S, Kohler HP. Sexual network structure and the spread of HIV in Africa: evidence from Likoma Island, Malawi. AIDS. 2007; 21(17): 2323-32.	353434
Malawi	2010	Malawi Demographic and Health Survey 2010	Survey microdata	GPS	ICF Macro, National Statistical Office of Malawi. Malawi Demographic and Health Survey 2010. Fairfax, United States: ICF International.	21393
Malawi	2015-16	Malawi Demographic and Health Survey 2015-2016	Survey microdata	GPS	Emory University and Centers for Disease Control & Prevention Collaboration, ICF International, Ministry of Health (Malawi), National Statistical Office of Malawi. Malawi Demographic and Health Survey 2015-2016. Fairfax, United States: ICF International, 2017.	218581
Malawi	2016	Malawi Population-based HIV Impact Assessment 2015-2016	Survey report	Admin >1	Ministry of Health, Malawi, Centers for Disease Control and Prevention (CDC), and ICAP at Columbia University. Malawi Population-based HIV Impact Assessment (MPHIA) 2015-16: First Report. Lilongwe, Malawi, Atlanta, Georgia and New York, New York, USA: Ministry of Health, CDC and ICAP. December 2016.	287629
Mali	2001	Mali Demographic and Health Survey 2001	Survey microdata	Admin 1	Macro International, Inc, National Directorate of Statistics and Informatics (DNSI) (Mali), Planning and Statistics Unit, Ministry of Health (Mali). Mali Demographic and Health	20315

Country	Years	Name	Type	Geographic Detail	Citation	NID*
					Survey 2001. Fairfax, United States: ICF International.	
Mali	2006	Mali Demographic and Health Survey 2006	Survey microdata	GPS	Macro International, Inc, Ministry of Health (Mali), National Directorate of Statistics and Informatics (DNSI) (Mali). Mali Demographic and Health Survey 2006. Fairfax, United States: ICF International.	20274
Mali	2012-13	Mali Demographic and Health Survey 2012-2013	Survey microdata	GPS	ICF International, INFO-STAT (Mali), Ministry of Health (Mali), National Institute of Statistics (INSTAT) (Mali), Planning and Statistics Unit, Ministry of Health (Mali). Mali Demographic and Health Survey 2012-2013. Fairfax, United States: ICF International, 2014.	77388
Mozambique	2009	Mozambique AIDS Indicator Survey 2009	Survey microdata	GPS	ICF Macro, Ministry of Health (Mozambique), National Institute of Statistics (INE) (Mozambique). Mozambique AIDS Indicator Survey 2009. Fairfax, United States: ICF International, 2010.	8906
Mozambique	2010-12	HIV Incidence and Spatial Clustering in a Rural Area of Southern Mozambique	Literature review	Site	Gonzalez R, Augusto OJ, Munguambe K, Pierrat C, Pedro EN, Sacoor C, De Lazzari E, Aponte JJ, Macete E, Alonso PL, Menendez C, Naniche D. HIV Incidence and Spatial Clustering in a Rural Area of Southern Mozambique. PLoS One. 2015; 10(7): e0132053.	353436
Mozambique	2015	Mozambique AIDS Indicator Survey 2015	Survey microdata	GPS	Centers for Disease Control and Prevention (CDC), ICF International, Ministry of Health (Mozambique), National Institute of Health (Mozambique), National Institute of Statistics (INE) (Mozambique). Mozambique AIDS Indicator Survey 2015. Fairfax, United States: ICF International, 2018.	157060
Namibia	2009	Incidence of HIV in Windhoek,	Literature review	Admin 2	Aulagnier M, Janssens W, De Beer I, van Rooy G, Gaeb E, Hesp C, van der Gaag J, Rinke	325204

Country	Years	Name	Type	Geographic Detail	Citation	NID*
		Namibia: demographic and socio-economic associations			de Wit TF. Incidence of HIV in Windhoek, Namibia: demographic and socio-economic associations. PLoS One. 2011; 6(10).	
Namibia	2013	Namibia Demographic and Health Survey 2013	Survey microdata	GPS	ICF International, Ministry of Health and Social Services (Namibia), Namibia Institute of Pathology, Namibia Statistics Agency. Namibia Demographic and Health Survey 2013. Fairfax, United States: ICF International.	150382
Namibia	2017	Namibia Population-based HIV Impact Assessment 2017	Survey report	Admin 1	Ministry of Health, Namibia, Centers for Disease Control and Prevention (CDC), University of California, San Francisco, and ICAP at Columbia University. Namibia Population-based HIV Impact Assessment (NAMPHIA) 2015-16: Summary Sheet. Windhoek, Namibia, Atlanta, Georgia and New York, New York, USA: Ministry of Health, CDC and ICAP. July 2018.	359089
Niger	2006	Niger Demographic and Health Survey 2006	Survey microdata	Admin 1	Department of Statistics and National Accounts (Niger), Macro International, Inc. Niger Demographic and Health Survey 2006. Fairfax, United States: ICF International.	20499
Niger	2012	Niger Demographic and Health Survey 2012	Survey microdata	Admin 1	ICF International, Ministry of Public Health (Niger), National Institute of Statistics (Niger). Niger Demographic and Health Survey 2012. Fairfax, United States: ICF International.	74393
Nigeria	2007-08	Nigeria National HIV/AIDS and Reproductive Health Survey 2007	Survey microdata	Admin 1	Federal Ministry of Health (Nigeria), National Bureau of Statistics (Nigeria), National Population Commission (NPC), Society for Family Health (Nigeria), University College Hospital, Ibadan. Nigeria National HIV/AIDS and Reproductive Health Survey 2007.	325046†
Nigeria	2012	Nigeria National	Survey microdata	Admin 2	Expanded Social Marketing Project in Nigeria (ESMPIN),	324443†

Country	Years	Name	Type	Geographic Detail	Citation	NID*
		HIV/AIDS and Reproductive Health Survey 2012			Federal Ministry of Health (Nigeria), Joint United Nations Program on HIV/AIDS (UNAIDS), National Population Commission (NPC), Society for Family Health (Nigeria), University College Hospital, Ibadan, World Health Organization (WHO). Nigeria National HIV/AIDS and Reproductive Health Survey 2012.	
Rwanda	2005	Rwanda Demographic and Health Survey 2005	Survey microdata	GPS	Macro International, Inc, National Institute of Statistics of Rwanda. Rwanda Demographic and Health Survey 2005. Calverton, United States: Macro International, Inc.	20740
Rwanda	2010-11	Rwanda Demographic and Health Survey 2010-2011	Survey microdata	GPS	ICF Macro, Ministry of Health (Rwanda), National Institute of Statistics of Rwanda. Rwanda Demographic and Health Survey 2010-2011. Calverton, United States: ICF Macro.	56040
Rwanda	2013	Rwanda AIDS Indicator and HIV Incidence Survey 2013	Survey report	Admin 1	Ministry of Health (Rwanda), Rwanda Biomedical Center. Rwanda AIDS Indicator and HIV Incidence Survey 2013.	343466
Rwanda	2014-15	Rwanda Demographic and Health Survey 2014-2015	Survey microdata	GPS	ICF International, Ministry of Health (Rwanda), National Institute of Statistics of Rwanda. Rwanda Demographic and Health Survey 2014-2015. Fairfax, United States: ICF International, 2016.	157063
Sao Tome and Principe	2008	Sao Tome and Principe Demographic and Health Survey 2008-2009	Survey microdata	Admin 2	ICF Macro, Ministry of Health (Sao Tome and Principe), National Institute of Statistics (Sao Tome and Principe). Sao Tome and Principe Demographic and Health Survey 2008-2009. Calverton, United States: ICF Macro.	26866
Sao Tome and Principe	2014	Sao Tome and Principe Multiple Indicator Cluster Survey:	Survey report	Admin 2	Global Fund to Fight Aids Tuberculosis and Malaria (GFATM), ICF International, National Center for Endemic Diseases (CNE) (Sao Tome and	233903

Country	Years	Name	Type	Geographic Detail	Citation	NID*
		Biomarker Data 2014			Principe), National Institute of Statistics (Sao Tome and Principe), United Nations Children's Fund (UNICEF), United Nations Development Programme (UNDP). Sao Tome and Principe Multiple Indicator Cluster Survey: Biomarker Data 2014. Fairfax, United States: ICF International.	
Senegal	2005	Senegal Demographic and Health Survey 2005	Survey microdata	GPS	Ministry of Health and Prevention (Senegal), Research Center for Human Development (Senegal). Senegal Demographic and Health Survey 2005. Calverton, United States: Macro International, Inc.	26855
Senegal	2010-11	Senegal Demographic and Health Survey 2010-2011	Survey microdata	GPS	Center for Research in Human Development (CRDH), Cheikh Anta Diop University, Hospital Aristide Le Dantec, ICF Macro, National Agency of Statistics and Demography (Senegal). Senegal Demographic and Health Survey 2010-2011. Fairfax, United States: ICF International.	56063
Senegal	2017	Senegal Continuous Demographic and Health Survey 2017	Survey microdata	Admin 1	ICF International, Ministry of Health and Social Action (Senegal), National Agency of Statistics and Demography (Senegal), Unit for the Fight Against Malnutrition (Senegal). Senegal Continuous Demographic and Health Survey 2017. Fairfax, United States: ICF International, 2018.	353526
Sierra Leone	2002	Sierra Leone HIV/AIDS Seroprevalence and Behavioral Risk Factor Survey 2002	Survey report	Admin >1, Admin >2	Centers for Disease Control and Prevention (CDC), Government of Sierra Leone, World Bank. Sierra Leone HIV/AIDS Seroprevalence and Behavioral Risk Factor Survey 2002.	356788†
Sierra Leone	2005	Sierra Leone National Population-based HIV	Survey report	Admin 2	Ministry of Health and Sanitation (Sierra Leone), National HIV/AIDS Secretariat (NAS) (Sierra Leone), Nimba Research and Consulting	325150

Country	Years	Name	Type	Geographic Detail	Citation	NID*
		Seroprevalence Survey 2005			(NiRC) (Ghana), Statistics Sierra Leone. Sierra Leone National Population-Based HIV Seroprevalence Survey 2005.	
Sierra Leone	2008	Sierra Leone Demographic and Health Survey 2008	Survey microdata	GPS	Macro International, Inc, Statistics Sierra Leone. Sierra Leone Demographic and Health Survey 2008. Fairfax, United States: ICF International.	21258
Sierra Leone	2013	Sierra Leone Demographic and Health Survey 2013	Survey microdata	GPS	ICF International, Ministry of Health and Sanitation (Sierra Leone), Statistics Sierra Leone. Sierra Leone Demographic and Health Survey 2013. Fairfax, United States: ICF International, 2014.	131467
South Africa	2002	Can highly active antiretroviral therapy reduce the spread of HIV?: A study in a township of South Africa	Literature review	Admin 3	Auvert B, Males S, Puren A, Taljaard D, Carael M, Williams B. Can highly active antiretroviral therapy reduce the spread of HIV?: A study in a township of South Africa. J Acquir Immune Defic Syndr. 2004; 36(1): 613-21.	353457
South Africa	2002	South Africa HIV/AIDS Behavioral Risks, Sero-Status, and Mass Media Impact Survey 2002	Survey microdata	Admin 3	Center for AIDS Development, Research and Evaluation (CADRE) (South Africa), Department of Social Development (South Africa), Family Health International, Geospace International (South Africa), Human Sciences Research Council, Joint United Nations Program on HIV/AIDS (UNAIDS), Medical University of Southern Africa (MEDUNSA), National Agency for AIDS Research (ANRS) (France), National Health Laboratory Service (NHLS) (South Africa), South African Medical Research Council, University of Natal, Wits Health Consortium. South Africa HIV/AIDS Behavioral Risks, Sero-Status, and Mass Media Impact Survey 2002. Pretoria, South Africa: Human	12102

Country	Years	Name	Type	Geographic Detail	Citation	NID*
					Sciences Research Council, 2011.	
South Africa	2004-16	South Africa - ACDIS Health and Demographic Surveillance System	Survey microdata	Admin <1	Africa Centre for Population Studies and Reproductive Health, INDEPTH, South African Medical Research Council, University of Kwazulu-Natal. South Africa - ACDIS Health and Demographic Surveillance System.	11780
South Africa	2005	South Africa National HIV Prevalence, Incidence, Behavior and Communication Survey 2004-2005	Survey microdata	Admin 3	Center for AIDS Development, Research and Evaluation (CADRE) (South Africa), Centers for Disease Control and Prevention (CDC), Global Clinical and Viral Laboratory (South Africa), Human Sciences Research Council, Maphume Research Services, National Institute for Communicable Diseases (South Africa). South Africa National HIV Prevalence, Incidence, Behavior and Communication Survey 2004-2005. Pretoria, South Africa: Human Sciences Research Council, 2011.	313074
South Africa	2008-09	South Africa National HIV Prevalence, Incidence, Behavior and Communication Survey 2008-2009	Survey microdata	Admin 5, GPS	Center for AIDS Development, Research and Evaluation (CADRE) (South Africa), Geospace International (South Africa), Global Clinical and Viral Laboratory (South Africa), Human Sciences Research Council, Maphume Research Services, National Institute for Communicable Diseases (South Africa), South African Medical Research Council. South Africa National HIV Prevalence, Incidence, Behavior and Communication Survey 2008-2009. Pretoria, South Africa: Human Sciences Research Council, 2014.	228102
South Africa	2010	Prevalence of HIV among those 15 and	Literature review	Site	Gomez-Olive FX, Angotti N, Houle B, Klipstein-Grobusch K, Kabudula C, Menken J, Williams J, Tollman S, Clark SJ.	353443

Country	Years	Name	Type	Geographic Detail	Citation	NID*
		older in rural South Africa			Prevalence of HIV among those 15 and older in rural South Africa. AIDS Care. 2013; 25(9): 1122-8.	
South Africa	2011-12	South Africa National HIV Prevalence, Incidence, and Behavior Survey 2011-2012	Survey microdata	Admin 5, GPS	Centers for Disease Control and Prevention (CDC), Global Clinical and Viral Laboratory (South Africa), Human Sciences Research Council, National Institute for Communicable Diseases (South Africa), South African Medical Research Council, University of Cape Town. South Africa National HIV Prevalence, Incidence, and Behavior Survey 2011-2012. Pretoria, South Africa: Human Sciences Research Council, 2016.	313076
South Africa	2012	Level of viral suppression and the cascade of HIV care in a South African semi-urban setting in 2012	Literature review	Admin 3	Jean K, Puren A, Cutler E, Singh B, Bouscaillou J, Rain-Taljaard R, Taljaard D, Gouws E, Lissouba P, Lewis DA, Peytavin G, Auvert B. Level of viral suppression and the cascade of HIV care in a South African semi-urban setting in 2012. AIDS. 2016; 30(13): 2107-16.	353455
South Africa	2013	Socio-economic and demographic factors related to HIV status in urban informal settlements in the Eastern Cape, South Africa	Literature review	Township	Steenkamp L, Venter D, Walsh C, Dana P. Socio-economic and demographic factors related to HIV status in urban informal settlements in the Eastern Cape, South Africa. Afr J AIDS Res. 2014; 13(3): 271-9.	353447
South Africa	2014	Attrition and Opportunities Along the HIV Care Continuum: Findings From a Population-based Sample, North West	Literature review	Admin 2	Lippman SA, Shade SB, El Ayadi AM, Gilvydis JM, Grignon JS, Liegler T, Morris J, Naidoo E, Prach LM, Puren A, Barnhart S. Attrition and Opportunities Along the HIV Care Continuum: Findings From a Population-Based Sample, North West Province,	353451

Country	Years	Name	Type	Geographic Detail	Citation	NID*
		Province, South Africa			South Africa. J Acquir Immune Defic Syndr. 2016; 73(1): 91-9.	
South Africa	2014	Transmission networks and risk of HIV infection in KwaZulu-Natal, South Africa: a community-wide phylogenetic study	Literature review	Site	de Oliveira T, Kharsany AB, Graf T, Cawood C, Khanyile D, Grobler A, Puren A, Madurai S, Baxter C, Karim QA, Karim SS. Transmission networks and risk of HIV infection in KwaZulu-Natal, South Africa: a community-wide phylogenetic study. Lancet HIV. 2017; 4(1): e41-e50.	353449
South Africa	2016	South Africa Demographic and Health Survey 2016	Survey microdata	GPS	Department of Health (South Africa), ICF International, South African Medical Research Council, Statistics South Africa. South Africa Demographic and Health Survey 2016. Fairfax, United States: ICF International, 2019.	157064
South Africa	2017	South Africa National HIV Prevalence, Incidence, Behavior and Communication Survey 2017	Survey report	Admin 1	Centers for Disease Control and Prevention (CDC), Human Sciences Research Council, National Institute for Communicable Diseases (South Africa), President's Emergency Plan for AIDS Relief (PEPFAR), South African Medical Research Council. South Africa National HIV Prevalence, Incidence, Behavior and Communication Survey 2017.	357200
South Sudan	2002	HIV, syphilis, herpes simplex virus 2, and behavioral surveillance among conflict-affected populations in Yei and Rumbek, southern Sudan	Literature review	City	Kaiser R, Kedamo T, Lane J, Kessia G, Downing R, Handzel T, Marum E, Salama P, Mermin J, Brady W, Spiegel P. HIV, syphilis, herpes simplex virus 2, and behavioral surveillance among conflict-affected populations in Yei and Rumbek, southern Sudan. AIDS. 2006; 20(6): 942-4.	138559
Swaziland	2006-07	Swaziland Demographic and Health	Survey microdata	GPS	Central Statistical Office (Swaziland), Macro International, Inc. Swaziland Demographic and Health	20829

Country	Years	Name	Type	Geographic Detail	Citation	NID*
		Survey 2006-2007			Survey 2006-2007. Fairfax, United States: ICF International.	
Swaziland	2011	Swaziland HIV Incidence Measurement Survey (SHIMS): Descriptive Data Tables, August 2014	Survey report	Admin 1	Centers for Disease Control and Prevention (CDC), ICAP, Columbia University Mailman School of Public Health, Ministry of Health (Swaziland). Swaziland HIV Incidence Measurement Survey (SHIMS): Descriptive Data Tables, August 2014. New York, New York: ICAP, Columbia University Mailman School of Public Health, 2014.	334391
Swaziland	2016	Swaziland HIV Incidence Measurement Survey Summary Sheet: Preliminary Findings November 2017	Survey report	Admin 1	Government of the Kingdom of eSwatini, Centers for Disease Control and Prevention (CDC), and ICAP at Columbia University. Swaziland HIV Incidence Measurement Survey (SHIMS2) 2016-17: Summary Sheet. Mbabane, eSwatini, Atlanta, Georgia and New York, New York, USA: Government of the Kingdom of eSwatini, CDC and ICAP. November 2017.	327588
Tanzania	2003	HIV prevalence and incidence in rural Tanzania: results from 10 years of follow-up in an open-cohort study	Literature review	Admin 3	Wambura M, Urassa M, Isingo R, Ndege M, Marston M, Slaymaker E, Mngara J, Changalucha J, Boerma TJ, Zaba B. HIV prevalence and incidence in rural Tanzania: results from 10 years of follow-up in an open-cohort study. J Acquir Immune Defic Syndr. 2007; 46(5): 616-23.	353475
Tanzania	2003-04	Tanzania AIDS Indicator Survey 2003-2004	Survey microdata	GPS	National Bureau of Statistics (Tanzania), ORC Macro, Tanzania Commission for AIDS (TACAIDS). Tanzania AIDS Indicator Survey 2003-2004. Calverton, United States: ORC Macro.	12630
Tanzania	2005	Trends in HIV-1 prevalence and risk behaviours	Literature review	Village	Mmbaga EJ, Hussain A, Leyna GH, Holm-Hansen C, Mnyika KS, Sam NE, Klouman E, Klepp KI. Trends in HIV-1 prevalence	353479

Country	Years	Name	Type	Geographic Detail	Citation	NID*
		over 15 years in a rural population in Kilimanjaro region of Tanzania			and risk behaviours over 15 years in a rural population in Kilimanjaro region of Tanzania. AIDS Res Ther. 2007; 4: 23.	
Tanzania	2007-08	Tanzania HIV/AIDS and Malaria Indicator Survey 2007-2008	Survey microdata	GPS	Macro International, Inc, National Bureau of Statistics (Tanzania), Office of Chief Government Statistician (OCGS-Zanzibar), Tanzania Commission for AIDS (TACAIDS), Zanzibar AIDS Commission (ZAC). Tanzania HIV/AIDS and Malaria Indicator Survey 2007-2008. Calverton, United States: Macro International, Inc.	12644
Tanzania	2010	Access to social capital and risk of HIV infection in Bukoba urban district, Kagera region, Tanzania	Literature review	Admin 2	Frumence G, Emmelin M, Eriksson M, Kwesigabo G, Killewo J, Moyo S, Nystrom L. Access to social capital and risk of HIV infection in Bukoba urban district, Kagera region, Tanzania. Arch Public Health. 2014; 72(1): 38.	353471
Tanzania	2011-12	Tanzania AIDS Indicator Survey 2011-2012	Survey microdata	GPS	ICF International, National Bureau of Statistics (Tanzania), Office of Chief Government Statistician (OCGS-Zanzibar), Tanzania Commission for AIDS (TACAIDS), Zanzibar AIDS Commission (ZAC). Tanzania AIDS Indicator Survey 2011-2012. Fairfax, United States: ICF International, 2013.	77395
Tanzania	2017	Tanzania HIV Impact Survey 2016-2017	Survey report	Admin 1	Ministry of Health, Community Development, Gender, Elderly and Children (MoHCDGEC) and the Ministry of Health Zanzibar, Tanzania, Centers for Disease Control and Prevention (CDC), and ICAP at Columbia University. Tanzania HIV Impact Survey (THIS) 2015-16: Summary Sheet. Dodoma, Tanzania, Atlanta, Georgia and New York, New York, USA: Ministry of Health,	327591

Country	Years	Name	Type	Geographic Detail	Citation	NID*
					CDC and ICAP. December 2017.	
Tanzania, Uganda	2012	High prevalence of hypertension and of risk factors for non-communicable diseases (NCDs): a population based cross-sectional survey of NCDS and HIV infection in Northwestern Tanzania and Southern Uganda	Literature review	City	Kavishe B, Biraro S, Baisley K, Vanobberghen F, Kapiga S, Munderi P, Smeeth L, Peck R, Mghamba J, Mutungi G, Ikoona E, Levin J, Bou Monclús MA, Katende D, Kisanga E, Hayes R, Grosskurth H. High prevalence of hypertension and of risk factors for non-communicable diseases (NCDs): a population based cross-sectional survey of NCDS and HIV infection in Northwestern Tanzania and Southern Uganda. BMC Med. 2015; 13: 126.	247441
The Gambia	2013	Gambia Demographic and Health Survey 2013	Survey microdata	Admin 2	Gambia Bureau of Statistics (GBOS), ICF International, Ministry of Health and Social Welfare (Gambia). Gambia Demographic and Health Survey 2013. Fairfax, United States: ICF International, 2015.	77384
Togo	2013-14	Togo Demographic and Health Survey 2013-2014	Survey microdata	GPS	Directorate General of Statistics and National Accounts (Togo), ICF International, Ministry of Health (Togo), Ministry of Planning, Development and Zoning (Togo). Togo Demographic and Health Survey 2013-2014. Fairfax, United States: ICF International, 2015.	77515
Uganda	2000-04	HIV prevalence and incidence are no longer falling in southwest Uganda: evidence from a rural population cohort 1989-2005	Literature review	Site	Shafer LA, Biraro S, Nakiyingi-Miiro J, Kamali A, Ssematimba D, Ouma J, Ojwiya A, Hughes P, Van der Paal L, Whitworth J, Opio A, Grosskurth H. HIV prevalence and incidence are no longer falling in southwest Uganda: evidence from a rural population cohort 1989-2005. AIDS. 2008; 22(13): 1641-9.	353506

Country	Years	Name	Type	Geographic Detail	Citation	NID*
Uganda	2004	Sero-prevalence of herpes simplex type 2 virus (HSV-2) and HIV infection in Kampala, Uganda	Literature review	Admin 3	Nakku-Joloba E, Kambugu F, Wasubire J, Kimeze J, Salata R, Albert JM, Rimm A, Whalen C. Sero-prevalence of herpes simplex type 2 virus (HSV-2) and HIV infection in Kampala, Uganda. Afr Health Sci. 2014; 14(4): 782-9.	353493
Uganda	2004-05	Uganda AIDS Indicator Survey 2004-2005	Survey microdata	Admin 1	Division of Reproductive Health, Centers for Disease Control and Prevention (CDC), Ministry of Health (Uganda). Uganda AIDS Indicator Survey 2004-2005. Calverton, United States: Macro International, Inc.	13084†
Uganda	2008	High HIV Prevalence and Associated Factors in a Remote Community in the Rwenzori Region of Western Uganda	Literature review	Admin 2	Rubaihayo J, Akib S, Mughusu E, Abaasa A. High HIV Prevalence and Associated Factors in a Remote Community in the Rwenzori Region of Western Uganda. Infect Dis Rep. 2010; 2(2): e13.	353491
Uganda	2011	The Cango Lyec Project - Healing the Elephant : HIV related vulnerabilities of post-conflict affected populations aged 13-49 years living in three Mid-Northern Uganda districts	Literature review	Admin 2, Admin 3	Malamba SS, Muyinda H, Spittal PM, Ekwaru JP, Kiwanuka N, Ogwang MD, Odong P, Kitandwe PK, Katamba A, Jongbloed K, Sewankambo NK, Kinyanda E, Blair A, Schechter MT. The Cango Lyec Project - Healing the Elephant : HIV related vulnerabilities of post-conflict affected populations aged 13-49 years living in three Mid-Northern Uganda districts. BMC Infect Dis. 2016; 16(1): 690.	353487
Uganda	2011	Uganda AIDS Indicator Survey 2011	Survey microdata	GPS	Centers for Disease Control and Prevention (CDC), ICF Macro, Ministry of Health (Uganda), Uganda Bureau of Statistics, Uganda Virus Research Institute. Uganda AIDS Indicator Survey 2011. Calverton, United States: ICF Macro.	55973

Country	Years	Name	Type	Geographic Detail	Citation	NID*
Uganda	2012	Heterogeneity of the HIV epidemic in agrarian, trading, and fishing communities in Rakai, Uganda: an observational epidemiological study	Literature review	Village	Chang LW, Grabowski MK, Ssekubugu R, Nalugoda F, Kigozi G, Nantume B, Lessler J, Moore SM, Quinn TC, Reynolds SJ, Gray RH, Serwadda D, Wawer MJ. Heterogeneity of the HIV epidemic in agrarian, trading, and fishing communities in Rakai, Uganda: an observational epidemiological study. Lancet HIV. 2016; 3(8): e388-e396.	353518
Uganda	2016	Uganda Population-based HIV Impact Assessment 2016-2017	Survey report	Admin 1	Ministry of Health, Uganda, Centers for Disease Control and Prevention (CDC), and ICAP at Columbia University. Uganda Population-based HIV Impact Assessment (UPHIA) 2016-17: Summary Sheet. Kampala, Uganda, Atlanta, Georgia and New York, New York, USA: Ministry of Health, CDC and ICAP. April 2018.	327593
Zambia	2002	Zambia Demographic and Health Survey 2001-2002	Survey microdata	Admin 1	Central Board of Health (Zambia), Central Statistical Office (Zambia), Macro International, Inc. Zambia Demographic and Health Survey 2001-2002. Fairfax, United States: ICF International.	21102
Zambia	2003	Steep HIV prevalence declines among young people in selected Zambian communities: population-based observations (1995-2003)	Literature review	Town	Michelo C, Sandoy IF, Dzekedzeke K, Siziya S, Fylkesnes K. Steep HIV prevalence declines among young people in selected Zambian communities: population-based observations (1995-2003). BMC Public Health. 2006; 6: 279.	353508
Zambia	2007	Zambia Demographic and Health Survey 2007	Survey microdata	GPS	Central Statistical Office (Zambia), Macro International, Inc. Zambia Demographic and Health Survey 2007. Fairfax, United States: ICF International.	21117

Country	Years	Name	Type	Geographic Detail	Citation	NID*
Zambia	2013	The adult prevalence of HIV in Zambia: results from a population based mobile testing survey conducted in 2013-2014	Literature review	Admin 1	Chanda-Kapata P, Kapata N, Klinkenberg E, William N, Mazyanga L, Musukwa K, Kawesha EC, Masiye F, Mwaba P. The adult prevalence of HIV in Zambia: results from a population based mobile testing survey conducted in 2013-2014. AIDS Res Ther. 2016; 13: 4.	353512
Zambia	2013-14	Zambia Demographic and Health Survey 2013-2014	Survey microdata	GPS	Central Statistical Office (Zambia), ICF International, Ministry of Health (Zambia), Tropical Diseases Research Centre, University Teaching Hospital (Zambia), University of Zambia. Zambia Demographic and Health Survey 2013-2014. Fairfax, United States: ICF International.	77516
Zambia	2016	Zambia Population-based HIV Impact Assessment 2016	Survey report	Admin 1	Ministry of Health, Zambia, Centers for Disease Control and Prevention (CDC), and ICAP at Columbia University. Zambia Population-based HIV Impact Assessment (ZAMPHIA) 2015-16: First Report. Lusaka, Zambia, Atlanta, Georgia and New York, New York, USA: Ministry of Health, CDC and ICAP. December 2016.	287630
Zimbabwe	2005	Zimbabwe - Chimanimani Behavioral Risks and HIV Serostatus Survey 2005	Survey report	Admin 2	Biomedical Research and Training Institute (Zimbabwe), Human Sciences Research Council, Zimbabwe Central Statistics Office. Zimbabwe - Chimanimani Behavioral Risks and HIV Serostatus Survey 2005.	333662
Zimbabwe	2005-06	Zimbabwe Demographic and Health Survey 2005-2006	Survey microdata	GPS	Central Statistical Office (Zimbabwe), Macro International, Inc. Zimbabwe Demographic and Health Survey 2005-2006. Calverton, United States: Macro International, Inc.	21163
Zimbabwe	2010-11	Zimbabwe Demographic and Health	Survey microdata	GPS	ICF Macro, Zimbabwe National Statistics Agency. Zimbabwe Demographic and Health	55992

Country	Years	Name	Type	Geographic Detail	Citation	NID*
		Survey 2010-2011			Survey 2010-2011. Calverton, United States: ICF Macro, 2012.	
Zimbabwe	2015	Zimbabwe Demographic and Health Survey 2015	Survey microdata	GPS	ICF International, National Microbiology Reference Laboratory, Harare Central Hospital (NMRL) (Zimbabwe), Zimbabwe National Statistics Agency. Zimbabwe Demographic and Health Survey 2015. Fairfax, United States: ICF International, 2016.	157066
Zimbabwe	2016	Zimbabwe Population-based HIV Impact Assessment 2015-2016	Survey report	Admin 1	Ministry of Health and Child Care (MOHCC), Zimbabwe, Centers for Disease Control and Prevention (CDC), and ICAP at Columbia University. Zimbabwe Population-based HIV Impact Assessment (ZIMPHIA) 2015-16: First Report. Harare, Zimbabwe, Atlanta, Georgia and New York, New York, USA: Ministry of Health, CDC and ICAP. December 2016.	287631

*NID = Data source unique identifier in the Global Health Data Exchange (http://ghdx.healthdata.org/). Additional information about each data sources is available via the GHDx, including information about the data provider and links to where the data can be accessed or requested (where available). NIDs can be entered in the search bar to retrieve the record for a particular source.

†Data source is not publicly available due to restrictions by the data provider and was used under license for the current study.

Supplementary Table 3: Data requiring age cross-walk

Country	Years	Name	Age Group	HIV	Male Circumcision
Burkina Faso	2000	Epidemiology of HIV infection in urban Burkina Faso	13-49	X	
Burundi	2002	Burundi National HIV Infection Seroprevalence Survey 2001	12+	X	
Cameroon	2017	Cameroon Population-based HIV Impact Assessment 2017-2018	15-64	X	
Cote d'Ivoire	2017	Côte d'Ivoire Population-based HIV Impact Assessment 2017-2018	15-64	X	
Guinea-Bissau	2005	Changes in prevalence and incidence of HIV-1, HIV-2 and dual infections in urban areas of Bissau, Guinea-Bissau: is HIV-2 disappearing?	15+	X	
Guinea-Bissau	2006	Two distinct epidemics: the rise of HIV-1 and decline of HIV-2 infection between 1990 and 2007 in rural Guinea-Bissau	15+	X	
Guinea-Bissau	2010	Guinea-Bissau HIV Prevalence Survey 2010	0+	X	
Kenya	2003	HIV prevalence and associated risk factors among individuals aged 13-34 years in Rural Western Kenya	13-34	X	
Kenya	2006	The effect of participant nonresponse on HIV prevalence estimates in a population-based survey in two informal settlements in Nairobi city	15-59	X	
Kenya	2012	Cascade of HIV care and population viral suppression in a high-burden region of Kenya	15-59	X	
Kenya	2013	Population levels and geographical distribution of HIV RNA in rural Ugandan and Kenyan communities, including serodiscordant couples: a cross-sectional analysis	15+	X	
Lesotho	2017	Lesotho Population-based HIV Impact Assessment 2016-2017	15-59	X	
Malawi	2005, 2007-09	Underestimation of HIV prevalence in surveys when some people already know their status, and ways to reduce the bias	15+	X	
Malawi	2006	Sexual network structure and the spread of HIV in Africa: evidence from Likoma Island, Malawi	18-35	X	
Malawi	2016	Malawi Population-based HIV Impact Assessment 2015-2016	15-64		X
Mozambique	2010	HIV Incidence and Spatial Clustering in a Rural Area of Southern Mozambique	18-47	X	
Mozambique	2012	HIV Incidence and Spatial Clustering in a Rural Area of Southern Mozambique	18-50	X	
Namibia	2017	Namibia Population-based HIV Impact Assessment 2017	15-64	X	
Rwanda	2013	Rwanda AIDS Indicator and HIV Incidence Survey 2013	15-59	X	
Sierra Leone	2002	Sierra Leone HIV/AIDS Seroprevalence and Behavioral Risk Factor Survey 2002	12-49	X	

Country	Years	Name	Age Group	HIV	Male Circumcision
South Africa	2012	Level of viral suppression and the cascade of HIV care in a South African semi-urban setting in 2012	18+	X	
South Africa	2013	Socio-economic and demographic factors related to HIV status in urban informal settlements in the Eastern Cape, South Africa	18-49	X	
South Africa	2014	Attrition and Opportunities Along the HIV Care Continuum: Findings From a Population-based Sample, North West Province, South Africa	18-49	X	
Swaziland	2011	Swaziland HIV Incidence Measurement Survey (SHIMS): Descriptive Data Tables, August 2014	18-49	X	
Swaziland	2016	Swaziland HIV Incidence Measurement Survey Summary Sheet: Preliminary Findings November 2017	15+	X	
Tanzania	2003	HIV prevalence and incidence in rural Tanzania: results from 10 years of follow-up in an open-cohort study	15-44	X	
Tanzania	2005	Trends in HIV-1 prevalence and risk behaviours over 15 years in a rural population in Kilimanjaro region of Tanzania	15-44	X	
Tanzania	2010	Access to social capital and risk of HIV infection in Bukoba urban district, Kagera region, Tanzania	15-64	X	
Tanzania	2012	High prevalence of hypertension and of risk factors for non-communicable diseases (NCDs): a population based cross-sectional survey of NCDS and HIV infection in Northwestern Tanzania and Southern Uganda	18+	X	
Tanzania	2017	Tanzania HIV Impact Survey 2016-2017	15-64	X	
Uganda	2000-04	HIV prevalence and incidence are no longer falling in southwest Uganda: evidence from a rural population cohort 1989-2005	13+	X	
Uganda	2004	Sero-prevalence of herpes simplex type 2 virus (HSV-2) and HIV infection in Kampala, Uganda	15-65	X	
Uganda	2011	The Cango Lyec Project - Healing the Elephant : HIV related vulnerabilities of post-conflict affected populations aged 13-49 years living in three Mid-Northern Uganda districts	13-49	X	
Uganda	2012	High prevalence of hypertension and of risk factors for non-communicable diseases (NCDs): a population based cross-sectional survey of NCDS and HIV infection in Northwestern Tanzania and Southern Uganda	18+	X	
Uganda	2013	Population levels and geographical distribution of HIV RNA in rural Ugandan and Kenyan communities, including serodiscordant couples: a cross-sectional analysis	15+	X	
Uganda	2016	Uganda Population-based HIV Impact Assessment 2016-2017	15-64	X	
Zambia	2013	The adult prevalence of HIV in Zambia: results from a population based mobile testing survey conducted in 2013-2014	15+	X	

Country	Years	Name	Age Group	HIV	Male Circumcision
Zimbabwe	2005	Zimbabwe - Chimanimani Behavioral Risks and HIV Serostatus Survey 2005	25-89	X	
Zimbabwe	2005	Zimbabwe - Chimanimani Behavioral Risks and HIV Serostatus Survey 2005	15-24		X
Zimbabwe	2016	Zimbabwe Population-based HIV Impact Assessment 2015-2016	15-64		X

Supplementary Table 4: ANC sentinel surveillance data

Country	Years	Source	Geographic Detail	Citation	NID*
Angola	2001-02	Report	GPS	Angola Prevalence of HIV, Hepatitis B, and Syphilis, and HIV Prevalence Among Women Attending Antenatal Care.	347096
Angola	2002	Report	GPS	Presentation of HIV Surveillance Information and CAP Survey.	347109
Angola	2004-05, 2007, 2009, 2011, 2013	2017 UNAIDS file	GPS	Joint United Nations Program on HIV/AIDS (UNAIDS). UNAIDS Spectrum - National HIV Estimates 2017. Geneva, Switzerland: Joint United Nations Program on HIV/AIDS (UNAIDS), 2017.	317607
Benin	2000-16	2017 UNAIDS file	GPS	Joint United Nations Program on HIV/AIDS (UNAIDS). UNAIDS Spectrum - National HIV Estimates 2017. Geneva, Switzerland: Joint United Nations Program on HIV/AIDS (UNAIDS), 2017.	317607
Botswana	2000	Report	Admin 2	Botswana AIDS/STD Unit, Centers for Disease Control and Prevention (CDC), Government of Botswana, National AIDS Coordinating Agency (Botswana), World Health Organization (WHO). Botswana HIV Sentinel Survey 2000.	347350
Botswana	2000-03, 2005-07, 2009, 2011	2017 UNAIDS file	Admin 2, GPS	Joint United Nations Program on HIV/AIDS (UNAIDS). UNAIDS Spectrum - National HIV Estimates 2017. Geneva, Switzerland: Joint United Nations Program on HIV/AIDS (UNAIDS), 2017.	317607
Botswana	2001-03	Report	Admin 2	Botswana AIDS/STD Unit, Botswana Harvard AIDS Institute Partnership (BHP), Centers for Disease Control and Prevention (CDC), Government of Botswana, National AIDS Coordinating Agency (Botswana), World Health Organization (WHO). Botswana HIV Sentinel Survey 2003.	347264
Botswana	2005	Report	Admin 2	Botswana Harvard AIDS Institute Partnership (BHP), Centers for Disease Control and Prevention (CDC), Department of AIDS Prevention and Care, Ministry of Health (Botswana), Joint United Nations Program on HIV/AIDS (UNAIDS), Ministry of Local Government (Botswana), National AIDS Coordinating Agency (Botswana), National Health Laboratory (NHL) (Botswana), World Health Organization (WHO). Botswana HIV Sentinel Survey 2005.	347307

Country	Years	Source	Geographic Detail	Citation	NID*
Botswana	2007	Report	Admin 2	African Comprehensive HIV/AIDS Partnerships (ACHAP), Botswana Harvard AIDS Institute Partnership (BHP), Centers for Disease Control and Prevention (CDC), Central Statistics Office (Botswana), Department of AIDS Prevention and Care, Ministry of Health (Botswana), Government of Botswana, Health Statistics Unit, Ministry of Health (Botswana), Joint United Nations Program on HIV/AIDS (UNAIDS), Ministry of Local Government (Botswana), National AIDS Coordinating Agency (Botswana), National Health Laboratory (NHL) (Botswana), World Health Organization (WHO). Botswana HIV ANC Sentinel Survey 2007.	347145
Burkina Faso	2000-16	2017 UNAIDS file	GPS	Joint United Nations Program on HIV/AIDS (UNAIDS). UNAIDS Spectrum - National HIV Estimates 2017. Geneva, Switzerland: Joint United Nations Program on HIV/AIDS (UNAIDS), 2017.	317607
Burkina Faso	2017	2018 UNAIDS file	GPS	Joint United Nations Program on HIV/AIDS (UNAIDS). UNAIDS Spectrum - National HIV Estimates 2018. Geneva, Switzerland: Joint United Nations Program on HIV/AIDS (UNAIDS), 2018.	365412
Burundi	2000-10	2017 UNAIDS file	GPS	Joint United Nations Program on HIV/AIDS (UNAIDS). UNAIDS Spectrum - National HIV Estimates 2017. Geneva, Switzerland: Joint United Nations Program on HIV/AIDS (UNAIDS), 2017.	317607
Burundi	2002	Report	GPS	Ministry of Public Health (Burundi), National AIDS and STDs Program (Burundi). Burundi Annual HIV/AIDS/STIs Epidemiological Surveillance Report 2002. Burundi: National AIDS and STDs Program (Burundi), 2002.	352105
Cameroon	2000, 2002, 2007, 2009, 2012, 2016	2017 UNAIDS file	GPS	Joint United Nations Program on HIV/AIDS (UNAIDS). UNAIDS Spectrum - National HIV Estimates 2017. Geneva, Switzerland: Joint United Nations Program on HIV/AIDS (UNAIDS), 2017.	317607
Cameroon	2002	Report	GPS	Ministry of Public Health (Cameroon), National AIDS Control Committee (Cameroon). Cameroon National HIV Serosurvey 2002.	352128
Cape Verde	2001-02, 2006-08	2016 UNAIDS files	Admin 2	Joint United Nations Program on HIV/AIDS (UNAIDS). UNAIDS Spectrum -	306517

Country	Years	Source	Geographic Detail	Citation	NID*
				National HIV Estimates 2016. Geneva, Switzerland: Joint United Nations Program on HIV/AIDS (UNAIDS).	
Central African Republic	2000, 2002, 2006, 2011, 2015	2017 UNAIDS file	GPS	Joint United Nations Program on HIV/AIDS (UNAIDS). UNAIDS Spectrum - National HIV Estimates 2017. Geneva, Switzerland: Joint United Nations Program on HIV/AIDS (UNAIDS), 2017.	317607
Chad	2000	Report	GPS	Chad HIV prevalence among women in ANC per Sentinel SIte 2000.	356793
Chad	2000, 2002-03, 2009-11, 2013-14	2017 UNAIDS file	GPS	Joint United Nations Program on HIV/AIDS (UNAIDS). UNAIDS Spectrum - National HIV Estimates 2017. Geneva, Switzerland: Joint United Nations Program on HIV/AIDS (UNAIDS), 2017.	317607
Chad	2002	Report	GPS	Ministry of Public Health (Chad). Chad Annual Report of Epidemiology Service Activities 2002, Epidemiological Surveillance of Second Generation HIV. N'Djamena, Chad: Ministry of Public Health (Chad), 2003.	354940
Comoros	2007-08, 2011-16	2018 UNAIDS file	Admin 1	Joint United Nations Program on HIV/AIDS (UNAIDS). UNAIDS Spectrum - National HIV Estimates 2018. Geneva, Switzerland: Joint United Nations Program on HIV/AIDS (UNAIDS), 2018.	365412
Congo	2002, 2005-06, 2011	2017 UNAIDS file	Admin 1, Admin 2	Joint United Nations Program on HIV/AIDS (UNAIDS). UNAIDS Spectrum - National HIV Estimates 2017. Geneva, Switzerland: Joint United Nations Program on HIV/AIDS (UNAIDS), 2017.	317607
Cote d'Ivoire	2000-02, 2004-05, 2008, 2013	2017 UNAIDS file	GPS	Joint United Nations Program on HIV/AIDS (UNAIDS). UNAIDS Spectrum - National HIV Estimates 2017. Geneva, Switzerland: Joint United Nations Program on HIV/AIDS (UNAIDS), 2017.	317607
Cote d'Ivoire	2017	2018 UNAIDS file	GPS	Joint United Nations Program on HIV/AIDS (UNAIDS). UNAIDS Spectrum - National HIV Estimates 2018. Geneva, Switzerland: Joint United Nations Program on HIV/AIDS (UNAIDS), 2018.	365412
Democratic Republic of the Congo	2000, 2003-09, 2011, 2013, 2015	2017 UNAIDS file	GPS	Joint United Nations Program on HIV/AIDS (UNAIDS). UNAIDS Spectrum - National HIV Estimates 2017. Geneva, Switzerland: Joint United Nations Program on HIV/AIDS (UNAIDS), 2017.	317607
Djibouti	2002, 2006-10, 2013-14	2017 UNAIDS file	GPS	Joint United Nations Program on HIV/AIDS (UNAIDS). UNAIDS Spectrum - National HIV Estimates 2017. Geneva,	317607

Country	Years	Source	Geographic Detail	Citation	NID*
				Switzerland: Joint United Nations Program on HIV/AIDS (UNAIDS), 2017.	
Equatorial Guinea	2008	Report	Admin 1	Castroverde Laboratory (Equatorial Guinea), Institute of Health Carlos III (Spain), Ministry of Science and Innovation (Spain), Reference Center for Endemic Control (CRCE) (Equatorial Guinea), Spanish Agency for International Development Cooperation (AECID). Equatorial Guinea HIV Seroprevalence Survey 2008.	355263
Eritrea	2003	Report	GPS	Ministry of Health (Eritrea). Eritrea HIV Sentinel Surveillance Survey 2003.	355283
Eritrea	2003	Report	GPS	Eritrea HIV Prevalence by Health Unit, Site, and Zone 1999-2003.	355296
Eritrea	2003, 2005, 2007	Report	GPS	Ministry of Health (Eritrea). Eritrea HIV and Syphilis Sentinel Surveillance Survey in ANC Attendee Women 2007.	143203
Eritrea	2003, 2005, 2007, 2009, 2011, 2013, 2015	2017 UNAIDS file	GPS	Joint United Nations Program on HIV/AIDS (UNAIDS). UNAIDS Spectrum - National HIV Estimates 2017. Geneva, Switzerland: Joint United Nations Program on HIV/AIDS (UNAIDS), 2017.	317607
Eritrea	2017	2018 UNAIDS file	GPS	Joint United Nations Program on HIV/AIDS (UNAIDS). UNAIDS Spectrum - National HIV Estimates 2018. Geneva, Switzerland: Joint United Nations Program on HIV/AIDS (UNAIDS), 2018.	365412
Ethiopia	2001-03	Report	GPS	Centers for Disease Control and Prevention (CDC), Ministry of Health (Ethiopia). Ethiopia - AIDS in Ethiopia Fifth Report. Addis Ababa, Ethiopia: Ministry of Health (Ethiopia), 2004.	316592
Ethiopia	2001-03, 2005, 2007, 2009, 2012, 2014	2017 UNAIDS file	GPS	Joint United Nations Program on HIV/AIDS (UNAIDS). UNAIDS Spectrum - National HIV Estimates 2017. Geneva, Switzerland: Joint United Nations Program on HIV/AIDS (UNAIDS), 2017.	317607
Ethiopia	2005	Report	GPS	Ministry of Health (Ethiopia). AIDS in Ethiopia Sixth Report. Addis Ababa, Ethiopia: Ministry of Health (Ethiopia).	143235
Ethiopia	2007, 2009	Report	GPS	Centers for Disease Control and Prevention (CDC), Ethiopian Health and Nutrition Research Center (EHNRI). Ethiopia Antenatal Care Sentinel HIV Surveillance 2009.	355394
Gabon	2001	Report	GPS	National AIDS Control Program (Gabon). Gabon HIV/AIDS Incidence, Prevalence, and Mortality 2001-2003. 2003.	355441

Country	Years	Source	Geographic Detail	Citation	NID*
Gabon	2001-03, 2007, 2009	2017 UNAIDS file	GPS	Joint United Nations Program on HIV/AIDS (UNAIDS). UNAIDS Spectrum - National HIV Estimates 2017. Geneva, Switzerland: Joint United Nations Program on HIV/AIDS (UNAIDS), 2017.	317607
Ghana	2000	Report	GPS	Ghana Health Service, National AIDS Control Program (NACP) (Ghana), National Public Health and Reference Laboratory (NHPRL)(Ghana), United States Agency for International Development (USAID), World Health Organization (WHO). Ghana HIV Sentinel Survey 2000.	355496
Ghana	2000-16	2017 UNAIDS file	GPS	Joint United Nations Program on HIV/AIDS (UNAIDS). UNAIDS Spectrum - National HIV Estimates 2017. Geneva, Switzerland: Joint United Nations Program on HIV/AIDS (UNAIDS), 2017.	317607
Ghana	2001	Report	GPS	Danish International Development Agency (DANIDA), Ghana Health Service, National AIDS Control Program (NACP) (Ghana), National Public Health and Reference Laboratory (NHPRL)(Ghana), United States Agency for International Development (USAID), World Health Organization (WHO). Ghana HIV Sentinel Survey 2001.	355494
Ghana	2003	Report	GPS	Ghana Health Service, National AIDS Control Program (NACP) (Ghana), National Public Health and Reference Laboratory (NHPRL)(Ghana), Noguchi Memorial Institute for Medical Research (NMIMR) (Ghana), World Health Organization (WHO). Ghana HIV Sentinel Survey 2003.	355475
Ghana	2005	Report	GPS	Ghana Health Service, National AIDS Control Program (NACP) (Ghana), National Public Health and Reference Laboratory (NHPRL)(Ghana), Noguchi Memorial Institute for Medical Research (NMIMR) (Ghana), World Health Organization (WHO). Ghana HIV Sentinel Survey 2005.	355473
Ghana	2006	Report	GPS	Ghana Health Service, National AIDS Control Program (NACP) (Ghana), National Public Health and Reference Laboratory (NHPRL)(Ghana), Noguchi Memorial Institute for Medical Research (NMIMR) (Ghana), World	355458

Country	Years	Source	Geographic Detail	Citation	NID*
				Health Organization (WHO). Ghana HIV Sentinel Survey 2006.	
Ghana	2017	2018 UNAIDS file	GPS	Joint United Nations Program on HIV/AIDS (UNAIDS). UNAIDS Spectrum - National HIV Estimates 2018. Geneva, Switzerland: Joint United Nations Program on HIV/AIDS (UNAIDS), 2018.	365412
Guinea	2001, 2004, 2008, 2015	2017 UNAIDS file	GPS	Joint United Nations Program on HIV/AIDS (UNAIDS). UNAIDS Spectrum - National HIV Estimates 2017. Geneva, Switzerland: Joint United Nations Program on HIV/AIDS (UNAIDS), 2017.	317607
Guinea-Bissau	2000-05, 2010, 2014	2017 UNAIDS file	Admin 2	Joint United Nations Program on HIV/AIDS (UNAIDS). UNAIDS Spectrum - National HIV Estimates 2017. Geneva, Switzerland: Joint United Nations Program on HIV/AIDS (UNAIDS), 2017.	317607
Kenya	2000-11	2018 UNAIDS file	GPS	Joint United Nations Program on HIV/AIDS (UNAIDS). UNAIDS Spectrum - National HIV Estimates 2018. Geneva, Switzerland: Joint United Nations Program on HIV/AIDS (UNAIDS), 2018.	365412
Kenya	2010	Report	GPS	Centers for Disease Control and Prevention (CDC), Kenya Medical Research Institute (KEMRI), MEASURE Evaluation Project, Carolina Population Center, University of North Carolina, National AIDS and STI Control Program (Kenya), National Aids Control Council (NACC), National HIV Reference Laboratory (NHRL) (Kenya), National Public Health Laboratory Services, Ministry of Public Health and Sanitation (Kenya), University of California San Francisco, World Health Organization (WHO). Kenya ANC HIV Sentinel Survey 2010.	355605
Kenya	2011	Report	GPS	Centers for Disease Control and Prevention (CDC), Kenya Medical Research Institute (KEMRI), MEASURE Evaluation Project, Carolina Population Center, University of North Carolina, National AIDS and STI Control Program (Kenya), National HIV Reference Laboratory (NHRL) (Kenya), National Public Health Laboratory Services, Ministry of Public Health and Sanitation (Kenya), World Health Organization (WHO). Kenya ANC HIV Sentinel Survey 2011.	355607

Country	Years	Source	Geographic Detail	Citation	NID*
Lesotho	2000, 2003, 2005, 2009, 2011, 2013, 2016	2017 UNAIDS file	GPS	Joint United Nations Program on HIV/AIDS (UNAIDS). UNAIDS Spectrum - National HIV Estimates 2017. Geneva, Switzerland: Joint United Nations Program on HIV/AIDS (UNAIDS), 2017.	317607
Liberia	2006-08, 2011, 2013	2017 UNAIDS file	GPS	Joint United Nations Program on HIV/AIDS (UNAIDS). UNAIDS Spectrum - National HIV Estimates 2017. Geneva, Switzerland: Joint United Nations Program on HIV/AIDS (UNAIDS), 2017.	317607
Liberia	2007-08	Report	GPS	Global Fund to Fight Aids Tuberculosis and Malaria (GFATM), National AIDS and STI Control Program (NACP), Ministry of Health and Social Welfare (Liberia), National Public Health Reference Laboratory (Liberia), United Nations Development Programme (UNDP), World Health Organization (WHO). Liberia HIV Sentinel Survey 2008.	355669
Liberia	2017	2018 UNAIDS file	GPS	Joint United Nations Program on HIV/AIDS (UNAIDS). UNAIDS Spectrum - National HIV Estimates 2018. Geneva, Switzerland: Joint United Nations Program on HIV/AIDS (UNAIDS), 2018.	365412
Madagascar	2003	Report	GPS	Executive Secretariat of the National Committee for the Fight against HIV/AIDS (SE CNLS) (Madagascar), Joint United Nations Program on HIV/AIDS (UNAIDS), United States Agency for International Development (USAID), World Bank, World Health Organization (WHO). Madagascar Analysis of the Epidemiological Profile and Determinants of HIV Infection 2007. 2008.	355675
Madagascar	2005	Report	GPS	Centers for Disease Control and Prevention (CDC), French Cooperation, Joint United Nations Program on HIV/AIDS (UNAIDS), Ministry of Health and Family Planning (Madagascar), National Institute of Public and Community Health (INSPC) (Madagascar), Pasteur Institute of Madagascar (IPM), United Nations Children's Fund (UNICEF), United States Agency for International Development (USAID), World Health Organization	355671

Country	Years	Source	Geographic Detail	Citation	NID*
				(WHO). Madagascar Biological Surveillance Survey 2005.	
Madagascar	2005, 2007, 2010-16	2017 UNAIDS file	GPS	Joint United Nations Program on HIV/AIDS (UNAIDS). UNAIDS Spectrum - National HIV Estimates 2017. Geneva, Switzerland: Joint United Nations Program on HIV/AIDS (UNAIDS), 2017.	317607
Madagascar	2017	2018 UNAIDS file	GPS	Joint United Nations Program on HIV/AIDS (UNAIDS). UNAIDS Spectrum - National HIV Estimates 2018. Geneva, Switzerland: Joint United Nations Program on HIV/AIDS (UNAIDS), 2018.	365412
Malawi	2001	Report	GPS	Ministry of Health and Population (Malawi), National AIDS Commission (Malawi). HIV/AIDS in Malawi 2003. Lilongwe, Malawi: National AIDS Commission (Malawi).	355677
Malawi	2001, 2003, 2005, 2007, 2010	2017 UNAIDS file	GPS	Joint United Nations Program on HIV/AIDS (UNAIDS). UNAIDS Spectrum - National HIV Estimates 2017. Geneva, Switzerland: Joint United Nations Program on HIV/AIDS (UNAIDS), 2017.	317607
Malawi	2003	Report	GPS	Ministry of Health (Malawi), National AIDS Commission (Malawi). Malawi HIV and Syphilis Sentinel Surveillance of Antenatal Clinic Attendees 2003.	157140
Malawi	2007	Report	GPS	Ministry of Health (Malawi), National AIDS Commission (Malawi). Malawi HIV and Syphilis Sero-Survey of Antenatal Clinic Attendees 2007.	157132
Mali	2002-03, 2005, 2007, 2009, 2012	2017 UNAIDS file	GPS	Joint United Nations Program on HIV/AIDS (UNAIDS). UNAIDS Spectrum - National HIV Estimates 2017. Geneva, Switzerland: Joint United Nations Program on HIV/AIDS (UNAIDS), 2017.	317607
Mali	2007	Report	GPS	Centers for Disease Control and Prevention (CDC), INFO-STAT (Mali), Ministry of Health (Mali), National Institute of Public Health Research (INRSP) (Mali). Mali HIV and Syphilis Sentinel Surveillance Survey 2007.	355931
Mauritania	2001, 2003, 2005, 2007	2016 UNAIDS files	GPS	Joint United Nations Program on HIV/AIDS (UNAIDS). UNAIDS Spectrum - National HIV Estimates 2016. Geneva, Switzerland: Joint United Nations Program on HIV/AIDS (UNAIDS).	306517
Mauritania	2001, 2003, 2007	Report	GPS	Joint United Nations Program on HIV/AIDS (UNAIDS), Ministry of Health and Social Affairs (Mauritania), National Executive Secretariat for AIDS Control	356151

Country	Years	Source	Geographic Detail	Citation	NID*
				(SENLS) (Mauritania), National Institute of Public Health Research (INRSP) (Mauritania), United Nations Children's Fund (UNICEF). Mauritania HIV Sentinel Surveillance Survey 2007.	
Mozambique	2000	Report	GPS	Mozambique Results of Epidemiological Surveillance - Sentinel Posts 2000.	356796
Mozambique	2000-02, 2004, 2007, 2009, 2011	2017 UNAIDS file	GPS	Joint United Nations Program on HIV/AIDS (UNAIDS). UNAIDS Spectrum - National HIV Estimates 2017. Geneva, Switzerland: Joint United Nations Program on HIV/AIDS (UNAIDS), 2017.	317607
Namibia	2000	Report	Admin 2	National AIDS Coordination Program (Namibia). Namibia Epidemiological Report on HIV/AIDS 2000. Namibia: National AIDS Coordination Program (Namibia), 2001.	356159
Namibia	2000, 2002, 2004, 2006, 2008, 2010, 2012, 2014, 2016	2017 UNAIDS file	Admin 2, GPS	Joint United Nations Program on HIV/AIDS (UNAIDS). UNAIDS Spectrum - National HIV Estimates 2017. Geneva, Switzerland: Joint United Nations Program on HIV/AIDS (UNAIDS), 2017.	317607
Namibia	2002	Report	Admin 2, GPS	Ministry of Health and Social Services (Namibia), Namibia Institute of Pathology, National AIDS Coordination Program (Namibia). Namibia National HIV Sentinel Survey 2002.	356248
Namibia	2017	2018 UNAIDS file	Admin 2	Joint United Nations Program on HIV/AIDS (UNAIDS). UNAIDS Spectrum - National HIV Estimates 2018. Geneva, Switzerland: Joint United Nations Program on HIV/AIDS (UNAIDS), 2018.	365412
Niger	2000	Report	GPS	National Program for the Fight Against AIDS/STDs (PNLS/MST) (Niger). Niger HIV Epidemiological Surveillance Report 2000. Niger: National Program for the Fight Against AIDS/STDs (PNLS/MST) (Niger), 2001.	356296
Niger	2000, 2009, 2012, 2014	2017 UNAIDS file	GPS	Joint United Nations Program on HIV/AIDS (UNAIDS). UNAIDS Spectrum - National HIV Estimates 2017. Geneva, Switzerland: Joint United Nations Program on HIV/AIDS (UNAIDS), 2017.	317607
Nigeria	2000-03, 2005, 2008, 2010, 2014	2017 UNAIDS file	GPS	Joint United Nations Program on HIV/AIDS (UNAIDS). UNAIDS Spectrum - National HIV Estimates 2017. Geneva,	317607

Country	Years	Source	Geographic Detail	Citation	NID*
				Switzerland: Joint United Nations Program on HIV/AIDS (UNAIDS), 2017.	
Nigeria	2003	Report	GPS	Federal Ministry of Health (Nigeria). Nigeria National HIV Seroprevalence Sentinel Survey 2003.	152886
Nigeria	2005	Report	GPS	Federal Ministry of Health (Nigeria). Nigeria National HIV/Syphilis Seroprevalence Sentinel Survey 2005.	138348
Nigeria	2005, 2008, 2010	Report	GPS	Federal Ministry of Health (Nigeria). Nigeria National HIV Seroprevalence Sentinel Survey 2010.	152887
Rwanda	2002	Report	GPS	Centers for Disease Control and Prevention (CDC), Ministry of Health (Rwanda). Rwanda HIV Sentinel Surveillance Survey 2002.	356349
Rwanda	2002-03, 2005, 2007, 2011, 2013	2017 UNAIDS file	GPS	Joint United Nations Program on HIV/AIDS (UNAIDS). UNAIDS Spectrum - National HIV Estimates 2017. Geneva, Switzerland: Joint United Nations Program on HIV/AIDS (UNAIDS), 2017.	317607
Senegal	2000-01	Report	Admin 1, GPS	Senegal HIV Prevalence in Men and Pregnant Women by Region 1989-2002.	356379
Senegal	2002	Report	Admin 1, GPS	Ministry of Health and Prevention (Senegal), National Council for the Fight Against AIDS (Senegal). Senegal Sero-Epidemiological Bulletin on HIV Surveillance No. 10, July 2003. Dakar, Senegal: National Council for the Fight Against AIDS (Senegal), 2003.	356380
Senegal	2004	Report	Admin 1	Centers for Disease Control and Prevention (CDC), Family Health International, National Council for the Fight Against AIDS (Senegal), United States Agency for International Development (USAID). Senegal Epidemiological Data of HIV/AIDS 2004-2005. Dakar, Senegal: National Council for the Fight Against AIDS (Senegal), 2006.	356362
Senegal	2005-07	Report	Admin 1	Centers for Disease Control and Prevention (CDC), Family Health International, Global Fund to Fight Aids Tuberculosis and Malaria (GFATM), National Council for the Fight Against AIDS (Senegal), United States Agency for International Development (USAID), World Health Organization (WHO). Senegal Epidemiological Bulletin on Sentinel Surveillance of HIV and Syphilis in Pregnant Women No. 13, November	356395

Country	Years	Source	Geographic Detail	Citation	NID*
				2008. Dakar, Senegal: National Council for the Fight Against AIDS (Senegal), 2008.	
Senegal	2009, 2011, 2014	2017 UNAIDS file	Admin 1	Joint United Nations Program on HIV/AIDS (UNAIDS). UNAIDS Spectrum - National HIV Estimates 2017. Geneva, Switzerland: Joint United Nations Program on HIV/AIDS (UNAIDS), 2017.	317607
Sierra Leone	2003, 2006-08, 2010, 2015	2016 UNAIDS files	GPS	Joint United Nations Program on HIV/AIDS (UNAIDS). UNAIDS Spectrum - National HIV Estimates 2016. Geneva, Switzerland: Joint United Nations Program on HIV/AIDS (UNAIDS).	306517
Sierra Leone	2015	2017 UNAIDS file	GPS	Joint United Nations Program on HIV/AIDS (UNAIDS). UNAIDS Spectrum - National HIV Estimates 2017. Geneva, Switzerland: Joint United Nations Program on HIV/AIDS (UNAIDS), 2017.	317607
Somalia	2004, 2007-08, 2010-11, 2014, 2016	2017 UNAIDS file	GPS	Joint United Nations Program on HIV/AIDS (UNAIDS). UNAIDS Spectrum - National HIV Estimates 2017. Geneva, Switzerland: Joint United Nations Program on HIV/AIDS (UNAIDS), 2017.	317607
South Africa	2000-14	2017 UNAIDS file	Admin 1, Admin 2, Admin 3	Joint United Nations Program on HIV/AIDS (UNAIDS). UNAIDS Spectrum - National HIV Estimates 2017. Geneva, Switzerland: Joint United Nations Program on HIV/AIDS (UNAIDS), 2017.	317607
South Africa	2006-08	Report	Admin 2, Admin 3	Department of Health (South Africa), Human Sciences Research Council, JSI Research & Training Institute, Joint United Nations Program on HIV/AIDS (UNAIDS), National Institute for Communicable Diseases (South Africa), South African Center of Excellence in Epidemiology Modelling and Analysis (SACEMA), South African Medical Research Council, University of Cape Town. South Africa National Antenatal Sentinel HIV and Syphilis Prevalence Survey 2008.	356401
South Africa	2009	Report	Admin 2	Department of Health (South Africa). South Africa National Antenatal Sentinel HIV and Syphilis Prevalence Survey 2010.	238529
South Sudan	2007, 2009, 2012	2017 UNAIDS file	GPS	Joint United Nations Program on HIV/AIDS (UNAIDS). UNAIDS Spectrum - National HIV Estimates 2017. Geneva, Switzerland: Joint United Nations Program on HIV/AIDS (UNAIDS), 2017.	317607

Country	Years	Source	Geographic Detail	Citation	NID*
Sudan	2004-05, 2007, 2009-10	2017 UNAIDS file	GPS	Joint United Nations Program on HIV/AIDS (UNAIDS). UNAIDS Spectrum - National HIV Estimates 2017. Geneva, Switzerland: Joint United Nations Program on HIV/AIDS (UNAIDS), 2017.	317607
Swaziland	2000, 2002, 2004, 2006, 2008, 2010	2017 UNAIDS file	Admin 1, GPS	Joint United Nations Program on HIV/AIDS (UNAIDS). UNAIDS Spectrum - National HIV Estimates 2017. Geneva, Switzerland: Joint United Nations Program on HIV/AIDS (UNAIDS), 2017.	317607
Tanzania	2000, 2002, 2005, 2008	Report	GPS	Centers for Disease Control and Prevention (CDC), Ministry of Health and Social Welfare (Zanzibar), Zanzibar AIDS Control Program. Zanzibar HIV Sentinel Surveillance Survey 2008.	356752
Tanzania	2000-01, 2003, 2005, 2008, 2011, 2014-16	2017 UNAIDS file	GPS	Joint United Nations Program on HIV/AIDS (UNAIDS). UNAIDS Spectrum - National HIV Estimates 2017. Geneva, Switzerland: Joint United Nations Program on HIV/AIDS (UNAIDS), 2017.	317607
Tanzania	2005, 2008, 2017	2018 UNAIDS file	GPS	Joint United Nations Program on HIV/AIDS (UNAIDS). UNAIDS Spectrum - National HIV Estimates 2018. Geneva, Switzerland: Joint United Nations Program on HIV/AIDS (UNAIDS), 2018.	365412
The Gambia	2001-08, 2011-12, 2014, 2016	2017 UNAIDS file	GPS	Joint United Nations Program on HIV/AIDS (UNAIDS). UNAIDS Spectrum - National HIV Estimates 2017. Geneva, Switzerland: Joint United Nations Program on HIV/AIDS (UNAIDS), 2017.	317607
The Gambia	2017	2018 UNAIDS file	GPS	Joint United Nations Program on HIV/AIDS (UNAIDS). UNAIDS Spectrum - National HIV Estimates 2018. Geneva, Switzerland: Joint United Nations Program on HIV/AIDS (UNAIDS), 2018.	365412
Togo	2001, 2003-04, 2006-11, 2014, 2016	2017 UNAIDS file	GPS	Joint United Nations Program on HIV/AIDS (UNAIDS). UNAIDS Spectrum - National HIV Estimates 2017. Geneva, Switzerland: Joint United Nations Program on HIV/AIDS (UNAIDS), 2017.	317607
Togo	2011	Report	GPS	Directorate General of Health (DGS) (Togo), Joint United Nations Program on HIV/AIDS (UNAIDS), National Program for the Fight Against HIV/AIDS (PNLS) (Togo), World Health Organization (WHO). Togo National HIV and Syphilis Sentinel Surveillance Survey 2011-2012.	356774

Country	Years	Source	Geographic Detail	Citation	NID*
Uganda	2000-01, 2003-07, 2009	Report	GPS	Centers for Disease Control and Prevention (CDC), Medical Research Council (Uganda), Ministry of Health (Uganda), National Institute of Health (Italy), Rakai Health Sciences Program (Uganda), STD/AIDS Control Program, Ministry of Health (Uganda), Uganda Virus Research Institute. Uganda HIV/AIDS Epidemiological Surveillance Report 2010. Uganda: STD/AIDS Control Program, Ministry of Health (Uganda), 2010.	321955
Uganda	2000-05, 2007, 2009-12, 2014	2017 UNAIDS file	GPS	Joint United Nations Program on HIV/AIDS (UNAIDS). UNAIDS Spectrum - National HIV Estimates 2017. Geneva, Switzerland: Joint United Nations Program on HIV/AIDS (UNAIDS), 2017.	317607
Uganda	2005-07	Report	GPS	Centers for Disease Control and Prevention (CDC), Joint United Nations Program on HIV/AIDS (UNAIDS), Medical Research Council (Uganda), Ministry of Health (Uganda), National Institute of Health (Italy), Rakai Health Sciences Program (Uganda), STD/AIDS Control Program, Ministry of Health (Uganda), Uganda AIDS Commission, Uganda Virus Research Institute, United States Agency for International Development (USAID), World Health Organization (WHO). Uganda HIV/AIDS Epidemiological Surveillance Report 2005-2007. Uganda: STD/AIDS Control Program, Ministry of Health (Uganda), 2009.	356783
Zambia	2002, 2004, 2006, 2008, 2011	2017 UNAIDS file	GPS	Joint United Nations Program on HIV/AIDS (UNAIDS). UNAIDS Spectrum - National HIV Estimates 2017. Geneva, Switzerland: Joint United Nations Program on HIV/AIDS (UNAIDS), 2017.	317607
Zimbabwe	2000-02, 2004, 2006, 2009, 2012	2016 UNAIDS files	GPS	Joint United Nations Program on HIV/AIDS (UNAIDS). UNAIDS Spectrum - National HIV Estimates 2016. Geneva, Switzerland: Joint United Nations Program on HIV/AIDS (UNAIDS).	306517

*NID = Data source unique identifier in the Global Health Data Exchange (http://ghdx.healthdata.org/). Additional information about each data sources is available via the GHDx, including information about the data provider and links to where the data can be accessed or requested (where available). NIDs can be entered in the search bar to retrieve the record for a particular source.

Supplementary Table 5: Sources for pre-existing covariates

Covariate	Temporal resolution	Source	Reference
Travel time to nearest settlement >50,000 inhabitants	Static	Malaria Atlas Project, Big Data Institute, Nuffield Department of Medicine, University of Oxford	Weiss, D. J. et al. A global map of travel time to cities to assess inequalities in accessibility in 2015. Nature 533, 333-336 (2018).
Night-time lights	Annual, 2000-13	NOAA DMSP	P Savory et al. Intercalibration and Gaussian Process Modeling of Nighttime Lights Imagery for Measuring Urbanization Trends in Africa 2000–2013. Remote Sens. 9 (2017).
Urbanicity	Annual, 2000-16	European Commission/ GHS	Pesaresi, M. et al. Operating procedure for the production of the Global Human Settlement Layer from Landsat data of the epochs 1975, 1990, 2000, and 2014. Publications Office of the European Union (2016).
Malaria incidence	Annual, 2000-17	Malaria Atlas Project	Bhatt, S. et al. The effect of malaria control on Plasmodium falciparum in Africa between 2000 and 2015. Nature 526, 207–211 (2015)<br>*2016 In Press.*
Population	Annual, 2000-17	WorldPop	Lloyd, C. T., Sorichetta, A. & Tatem, A. J. High resolution global gridded data for use in population studies. Sci. Data 4, sdata20171 (2017).<br><br>World Pop. Get data. Available at: http://www.worldpop.org.uk/data/get_data/. (Accessed: 25th July 2017)

Supplementary Table 6: HIV covariate survey data

Country	Years	Name	Type	Geographic Detail	Male Circumcision	STI Symptoms	Had Intercourse (Women)	Partner Away	Condom Last Time	Multiple Partners in Year (Men)	Multiple Partners in Year (Women)	In Union	Citation	NID*
Angola	2001	Angola Multiple Indicator Cluster Survey 2001	Survey microdata	Admin 1								X	National Institute of Statistics (Angola), United Nations Children's Fund (UNICEF). Angola Multiple Indicator Cluster Survey 2001. New York, United States: United Nations Children's Fund (UNICEF).	687
Angola	2006	Angola Core Welfare Indicators Questionnaire Survey 2005	Survey microdata	Admin 1								X	National Institute of Statistics (Angola). Angola Core Welfare Indicators Questionnaire Survey 2005.	151566
Angola	2011	Angola Core Welfare Indicators Questionnaire Survey 2011	Survey microdata	Admin 1								X	Ministry of Planning and Territorial Development (Angola), National Institute of Statistics (Angola). Angola Core Welfare Indicators Questionnaire Survey 2011. Luanda, Angola: National Institute of Statistics (Angola).	151568
Angola	2015-16	Angola Demograp	Survey microdata	GPS	X	X	X	X	X	X	X	X	ICF International, Ministry of Health (Angola), National Institute of	218555

Country	Years	Name	Type	Geographic Detail	Male Circumcision	STI Symptoms	Had Intercourse (Women)	Partner Away	Condom Last Time	Multiple Partners in Year (Men)	Multiple Partners in Year (Women)	In Union	Citation	NID*
		hic and Health Survey 2015-2016											Statistics (Angola), United Nations Children's Fund (UNICEF). Angola Demographic and Health Survey 2015-2016. Fairfax, United States: ICF International, 2017.	
Benin	2001	Benin Demographic and Health Survey 2001	Survey microdata	GPS			X	X			X		National Institute of Statistics and Economic Analysis (INSAE) (Benin), ORC Macro. Benin Demographic and Health Survey 2001. Fairfax, United States: ICF International.	18950
Benin	2006	Benin Demographic and Health Survey 2006	Survey microdata	Admin 1	X	X	X	X	X	X		X	Macro International, Inc, National Institute of Statistics and Economic Analysis (INSAE) (Benin), National Program Against AIDS (PNLS) (Benin). Benin Demographic and Health Survey 2006. Fairfax, United States: ICF International.	18959
Benin	2011-12	Benin Demographic and Health Survey 2011-2012	Survey microdata	GPS	X	X	X	X	X	X	X	X	ICF International, National Institute of Statistics and Economic Analysis (INSAE) (Benin), National Program Against AIDS (PNLS) (Benin). Benin Demographic and Health Survey 2011-2012. Fairfax, United States: ICF International, 2014.	79839

Country	Years	Name	Type	Geographic Detail	Male Circumcision	STI Symptoms	Had Intercourse (Women)	Partner Away	Condom Last Time	Multiple Partners in Year (Men)	Multiple Partners in Year (Women)	In Union	Citation	NID*
Benin	2012	Benin Integrated Modular Survey on Household Living Conditions 2011-2012	Survey microdata	Admin 1								X	Danish International Development Agency (DANIDA), German Society for International Cooperation (GIZ), National Institute of Statistics and Economic Analysis (INSAE) (Benin). Benin Integrated Modular Survey on Household Living Conditions 2011-2012. Cotonou, Benin: National Institute of Statistics and Economic Analysis (INSAE) (Benin).	151805
Benin	2014	Benin Multiple Indicator Cluster Survey 2014	Survey microdata	Admin 1	X		X		X	X	X	X	National Institute of Statistics and Economic Analysis (INSAE) (Benin), United Nations Children's Fund (UNICEF). Benin Multiple Indicator Cluster Survey 2014. New York, United States: United Nations Children's Fund (UNICEF), 2017.	206075
Botswana	2000	Botswana Multiple Indicator Cluster Survey 2000	Survey microdata	Admin 1								X	Central Statistics Office (Botswana), United Nations Children's Fund (UNICEF). Botswana Multiple Indicator Cluster Survey 2000. New York, United States: United Nations Children's Fund (UNICEF), 2015.	1404
Botswana	2001	Botswana AIDS	Survey microdata	Admin <1	X	X	X		X			X	Central Statistics Office (Botswana), Ministry of Health (Botswana).	22112

Country	Years	Name	Type	Geographic Detail	Male Circumcision	STI Symptoms	Had Intercourse (Women)	Partner Away	Condom Last Time	Multiple Partners in Year (Men)	Multiple Partners in Year (Women)	In Union	Citation	NID*
		Impact Survey 2001											Botswana AIDS Impact Survey 2001. Gaborone, Botswana: Statistics Botswana.	
Botswana	2004	Botswana AIDS Impact Survey 2004	Survey microdata	GPS	X	X	X		X	X	X	X	Central Statistics Office (Botswana). Botswana AIDS Impact Survey 2004. Gaborone, Botswana: Statistics Botswana.	22114
Botswana	2007-08	Botswana Family Health Survey 2007-2008	Survey microdata	Admin 4			X	X				X	Central Statistics Office (Botswana). Botswana Family Health Survey 2007-2008. Gaborone, Botswana: Central Statistics Office (Botswana), 2009.	22125
Botswana	2008	Botswana AIDS Impact Survey 2008	Survey microdata	GPS	X	X	X	X	X	X	X	X	Central Statistics Office (Botswana), National AIDS Coordinating Agency (Botswana). Botswana AIDS Impact Survey 2008. Gaborone, Botswana: Statistics Botswana, 2015.	22116
Botswana	2013	Botswana AIDS Impact Survey 2013	Survey microdata	GPS	X	X	X	X	X	X	X	X	Ministry of Health (Botswana), National AIDS Coordinating Agency (Botswana), Statistics Botswana. Botswana AIDS Impact Survey 2013. Gaborone, Botswana: Statistics Botswana, 2015.	134753

Country	Years	Name	Type	Geographic Detail	Male Circumcision	STI Symptoms	Had Intercourse (Women)	Partner Away	Condom Last Time	Multiple Partners in Year (Men)	Multiple Partners in Year (Women)	In Union	Citation	NID*
Burkina Faso	2003	Burkina Faso Core Welfare Indicators Questionnaire Survey 2003	Survey microdata	Admin 3								X	National Institute of Statistics and Demography (Burkina Faso), World Bank. Burkina Faso Core Welfare Indicators Questionnaire Survey 2003. Ouagadougou, Burkina Faso: National Institute of Statistics and Demography (Burkina Faso).	1855†
Burkina Faso	2003	Burkina Faso Demographic and Health Survey 2003	Survey microdata	GPS	X	X	X	X	X	X	X	X	Macro International, Inc, National Institute of Statistics and Demography (Burkina Faso). Burkina Faso Demographic and Health Survey 2003. Fairfax, United States: ICF International.	19088
Burkina Faso	2006	Burkina Faso Multiple Indicator Cluster Survey 2006	Survey microdata	GPS			X				X		National Institute of Statistics and Demography (Burkina Faso), United Nations Children's Fund (UNICEF). Burkina Faso Multiple Indicator Cluster Survey 2006. New York, United States: United Nations Children's Fund (UNICEF).	1927
Burkina Faso	2007	Burkina Faso Core Welfare Indicators	Survey microdata	Admin 1								X	National Institute of Statistics and Demography (INSD). Burkina Faso Core Welfare Indicators Questionnaire Survey 2007. Ouagadougou, Burkina	18499†

Country	Years	Name	Type	Geographic Detail	Male Circumcision	STI Symptoms	Had Intercourse (Women)	Partner Away	Condom Last Time	Multiple Partners in Year (Men)	Multiple Partners in Year (Women)	In Union	Citation	NID*
		Questionnaire Survey 2007											Faso: National Institute of Statistics and Demography (INSD), 2008.	
Burkina Faso	2010	Burkina Faso Demographic and Health Survey 2010-2011	Survey microdata	GPS	X	X	X	X	X	X	X	X	ICF Macro, Ministry of Health (Burkina Faso), National Institute of Statistics and Demography (Burkina Faso). Burkina Faso Demographic and Health Survey 2010-2011. Fairfax, United States: ICF International.	19133
Burkina Faso	2014	Burkina Faso Performance Monitoring and Accountability 2020 Survey, Round 1 2014	Survey microdata	Admin 1			X	X				X	Burkina Faso Institut national de la statistique et de la demographie (National Institute of Statistics and Demography) and The Bill & Melinda Gates Institute for Population and Reproductive Health at The Johns Hopkins Bloomberg School of Public Health. Performance Monitoring and Accountability 2020 (PMA2020) Survey round 1, PMA2014/Burkina Faso-R1. 2014. Ouagadougou, Burkina Faso and Baltimore, Maryland, USA.	257044
Burkina Faso	2015	Burkina Faso Performan	Survey microdata	Admin 1			X	X				X	Burkina Faso Institut national de la statistique et de la demographie (National Institute of Statistics and	257045

Country	Years	Name	Type	Geographic Detail	Male Circumcision	STI Symptoms	Had Intercourse (Women)	Partner Away	Condom Last Time	Multiple Partners in Year (Men)	Multiple Partners in Year (Women)	In Union	Citation	NID*
		ce Monitoring and Accountability 2020 Survey, Round 2 2015											Demography), and The Bill & Melinda Gates Institute for Population and Reproductive Health at The Johns Hopkins Bloomberg School of Public Health. Performance Monitoring and Accountability 2020 (PMA2020) Survey round 2, PMA2015/Burkina Faso-R2. 2015. Ouagadougou, Burkina Faso and Baltimore, Maryland, USA.	
Burkina Faso	2016	Burkina Faso Performance Monitoring and Accountability 2020 Survey, Round 3 2016	Survey microdata	Admin 1			X	X				X	Burkina Faso Institut national de la statistique et de la demographie (National Institute of Statistics and Demography), and The Bill & Melinda Gates Institute for Population and Reproductive Health at The Johns Hopkins Bloomberg School of Public Health. Performance Monitoring and Accountability 2020 (PMA2020) Survey round 3, PMA2016/Burkina Faso-R3. 2016. Ouagadougou, Burkina Faso and Baltimore, Maryland, USA.	285993
Burkina Faso	2016	Burkina Faso Performance	Survey microdata	Admin 1			X	X				X	Burkina Faso Institut national de la statistique et de la demographie (National Institute of Statistics and Demography), and The Bill & Melinda	307751

Country	Years	Name	Type	Geographic Detail	Male Circumcision	STI Symptoms	Had Intercourse (Women)	Partner Away	Condom Last Time	Multiple Partners in Year (Men)	Multiple Partners in Year (Women)	In Union	Citation	NID*
		Monitoring and Accountability 2020 Survey, Round 4 2016-2017											Gates Institute for Population and Reproductive Health at The Johns Hopkins Bloomberg School of Public Health. Performance Monitoring and Accountability 2020 (PMA2020) Survey round 4, PMA2016/Burkina Faso-R4. 2016. Ouagadougou, Burkina Faso and Baltimore, Maryland, USA?.	
Burundi	2000	Burundi Multiple Indicator Cluster Survey 2000	Survey microdata	Admin 1								X	Burundi Institute of Statistics and Economic Studies, United Nations Children's Fund (UNICEF). Burundi Multiple Indicator Cluster Survey 2000. New York, United States: United Nations Children's Fund (UNICEF).	1994
Burundi	2005	Burundi Multiple Indicator Cluster Survey 2005	Survey microdata	Admin 1			X		X	X			United Nations Children's Fund (UNICEF), Burundi Institute of Statistics and Economic Studies, United Nations Population Fund (UNFPA). Burundi Multiple Indicator Cluster Survey 2005. New York, United States: United Nations Children's Fund (UNICEF).	1981
Burundi	2010-11	Burundi Demographic and	Survey microdata	GPS	X	X	X	X	X	X	X	X	Burundi Institute of Statistics and Economic Studies, ICF International, Ministry of Public Health and the Fight	30431

Country	Years	Name	Type	Geographic Detail	Male Circumcision	STI Symptoms	Had Intercourse (Women)	Partner Away	Condom Last Time	Multiple Partners in Year (Men)	Multiple Partners in Year (Women)	In Union	Citation	NID*
		Health Survey 2010-2011											Against AIDS (Burundi). Burundi Demographic and Health Survey 2010-2011. Fairfax, United States: ICF International, 2012.	
Burundi	2016-17	Burundi Demographic and Health Survey 2016-2017	Survey microdata	GPS	X	X	X	X	X	X	X	X	Burundi Institute of Statistics and Economic Studies, ICF International, Ministry of Public Health and the Fight Against AIDS (Burundi). Burundi Demographic and Health Survey 2016-2017. Fairfax, United States: ICF International, 2018.	286766
Cameroon	2000	Cameroon Multiple Indicator Cluster Survey 2000	Survey microdata	Admin 1								X	Directorate of Statistics and National Accounts, Ministry of Economics and Finance (Cameroon), United Nations Children's Fund (UNICEF). Cameroon Multiple Indicator Cluster Survey 2000. New York, United States: United Nations Children's Fund (UNICEF).	2053
Cameroon	2001	Cameroon Household Survey 2001	Survey microdata	Admin 1, Admin 7								X	National Institute of Statistics (Cameroon), Directorate of Statistics and National Accounts, Ministry of Economics and Finance (Cameroon), AFRISTAT. Cameroon Household Survey 2001. Yaounde, Cameroon:	2039

Country	Years	Name	Type	Geographic Detail	Male Circumcision	STI Symptoms	Had Intercourse (Women)	Partner Away	Condom Last Time	Multiple Partners in Year (Men)	Multiple Partners in Year (Women)	In Union	Citation	NID*
													National Institute of Statistics (Cameroon).	
Cameroon	2004	Cameroon Demographic and Health Survey 2004	Survey microdata	GPS	X	X	X	X	X	X	X	X	Macro International, Inc, National Institute of Statistics (Cameroon). Cameroon Demographic and Health Survey 2004. Fairfax, United States: ICF International.	19211
Cameroon	2006	Cameroon Multiple Indicator Cluster Survey 2006	Survey microdata	Admin 1, Admin 3							X	X	United Nations Children's Fund (UNICEF), National Institute of Statistics (Cameroon). Cameroon Multiple Indicator Cluster Survey 2006. New York, United States: United Nations Children's Fund (UNICEF).	2063
Cameroon	2011	Cameroon Demographic and Health Survey 2011	Survey microdata	GPS	X	X	X	X	X	X	X	X	ICF International, Ministry of Economy, Planning and Regional Development (Cameroon), Ministry of Public Health (Cameroon), National Institute of Statistics (Cameroon), Pasteur Center of Cameroon. Cameroon Demographic and Health Survey 2011. Fairfax, United States: ICF International.	19274
Cameroon	2014	Cameroon Multiple	Survey microdata	Admin 3			X		X	X	X	X	Ministry of Public Health (Cameroon), National Institute of Statistics	244455

Country	Years	Name	Type	Geographic Detail	Male Circumcision	STI Symptoms	Had Intercourse (Women)	Partner Away	Condom Last Time	Multiple Partners in Year (Men)	Multiple Partners in Year (Women)	In Union	Citation	NID*
		Indicator Cluster Survey 2014											(Cameroon), United Nations Children's Fund (UNICEF). Cameroon Multiple Indicator Cluster Survey 2014. New York, United States: United Nations Children's Fund (UNICEF), 2017.	
Central African Republic	2006	Central African Republic Multiple Indicator Cluster Survey 2006	Survey microdata	Admin 1	X		X		X	X	X	X	United Nations Children's Fund (UNICEF). Central African Republic Multiple Indicator Cluster Survey 2006. New York, United States: United Nations Children's Fund (UNICEF).	2223
Central African Republic	2010	Central African Republic Multiple Indicator Cluster Survey 2010-2011	Survey microdata	Admin 1			X		X	X	X	X	Central African Institute of Statistics, Economic and Social Studies (ICASEES) (Central African Republic), ICF International. Central African Republic Multiple Indicator Cluster Survey 2010-2011. Fairfax, United States: ICF International, 2013.	82832
Chad	2000	Chad Multiple Indicator	Survey microdata	Admin 1								X	United Nations Children's Fund (UNICEF), Census Bureau (Chad), National Institute of Statistical,	2244

Country	Years	Name	Type	Geographic Detail	Male Circumcision	STI Symptoms	Had Intercourse (Women)	Partner Away	Condom Last Time	Multiple Partners in Year (Men)	Multiple Partners in Year (Women)	In Union	Citation	NID*
		Cluster Survey 2000											Economic and Demographic Studies (Chad). Chad Multiple Indicator Cluster Survey 2000. New York, United States: United Nations Children's Fund (UNICEF).	
Chad	2004	Chad Demographic and Health Survey 2004	Survey microdata	Admin 1	X	X	X	X	X	X	X	X	Macro International, Inc, National Institute of Statistical, Economic and Demographic Studies (Chad). Chad Demographic and Health Survey 2004. Fairfax, United States: ICF International.	19315
Chad	2010	Chad Multiple Indicator Cluster Survey 2010	Survey microdata	Admin 2			X				X		Ministry of Planning, Economy, and International Cooperation (Chad), National Institute of Statistical, Economic and Demographic Studies (Chad), United Nations Children's Fund (UNICEF). Chad Multiple Indicator Cluster Survey 2010. New York, United States: United Nations Children's Fund (UNICEF), 2014.	76701
Chad	2014-15	Chad Demographic and Health	Survey microdata	GPS	X	X	X	X	X	X	X	X	ICF International, National Institute of Statistical, Economic and Demographic Studies (Chad). Chad Demographic and Health Survey 2014-	157025

Country	Years	Name	Type	Geographic Detail	Male Circumcision	STI Symptoms	Had Intercourse (Women)	Partner Away	Condom Last Time	Multiple Partners in Year (Men)	Multiple Partners in Year (Women)	In Union	Citation	NID*
		Survey 2014-2015											2015. Fairfax, United States: ICF International, 2016.	
Comoros	2000	Comoros Multiple Indicator Cluster Survey 2000	Survey microdata	Admin 1								X	United Nations Development Programme (UNDP), United Nations Children's Fund (UNICEF). Comoros Multiple Indicator Cluster Survey 2000. New York, United States: United Nations Children's Fund (UNICEF).	3114
Comoros	2012	Comoros Demographic and Health Survey 2012-2013	Survey microdata	GPS	X	X	X	X	X	X	X	X	General Directorate of Statistics and Forecasting (Comoros), ICF International. Comoros Demographic and Health Survey 2012-2013. Fairfax, United States: ICF International.	76850
Congo	2005	Congo Demographic and Health Survey 2005	Survey microdata	Admin 1		X	X	X	X	X	X	X	Macro International, Inc, National Center for Statistics and Economic Studies (Congo, Rep.). Congo Demographic and Health Survey 2005. Fairfax, United States: ICF International.	19391
Congo	2009	Congo AIDS Indicator Survey 2009	Survey microdata	Admin 1	X	X	X	X	X	X	X	X	ICF Macro, National Center for Statistics and Economic Studies (Congo, Rep.). Congo AIDS Indicator Survey 2009. Fairfax, United States: ICF International.	3133

Country	Years	Name	Type	Geographic Detail	Male Circumcision	STI Symptoms	Had Intercourse (Women)	Partner Away	Condom Last Time	Multiple Partners in Year (Men)	Multiple Partners in Year (Women)	In Union	Citation	NID*
Congo	2011	Congo Demographic and Health Survey 2011-2012	Survey microdata	Admin 1	X	X	X	X	X	X	X	X	ICF International, Ministry of Health (Congo, Rep.), National Center for Statistics and Economic Studies (Congo, Rep.). Congo Demographic and Health Survey 2011-2012. Fairfax, United States: ICF International.	56151
Congo	2014-15	Congo Multiple Indicator Cluster Survey 2014-2015	Survey microdata	Admin 1	X								National Institute of Statistics (INS) (Congo), United Nations Children's Fund (UNICEF). Congo Multiple Indicator Cluster Survey 2014-2015. New York, United States: United Nations Children's Fund (UNICEF), 2018.	234733
Cote d'Ivoire	2000	Côte d'Ivoire Multiple Indicator Cluster Survey 2000	Survey microdata	Admin >1								X	National School for Statistics and Economics Applied (ENSEA), United Nations Children's Fund (UNICEF), United Nations Educational, Scientific and Cultural Organization (UNESCO). Côte d'Ivoire Multiple Indicator Cluster Survey 2000. New York, United States: United Nations Children's Fund (UNICEF).	26444
Cote d'Ivoire	2005	Côte d'Ivoire AIDS	Survey microdata	Admin >1	X	X	X	X	X	X	X	X	CDC Retro-CI, Ministry of the Fight Against AIDS (Côte d'Ivoire), National Institute of Statistics (Côte d'Ivoire),	56148

Country	Years	Name	Type	Geographic Detail	Male Circumcision	STI Symptoms	Had Intercourse (Women)	Partner Away	Condom Last Time	Multiple Partners in Year (Men)	Multiple Partners in Year (Women)	In Union	Citation	NID*
		Indicator Survey 2005											ORC Macro. Côte d'Ivoire AIDS Indicator Survey 2005. Fairfax, United States: ICF International.	
Cote d'Ivoire	2011-12	Côte d'Ivoire Demographic and Health Survey 2011-2012	Survey microdata	GPS	X	X	X	X	X	X	X	X	ICF International, Ministry of the Fight Against AIDS (Côte d'Ivoire), National Institute of Statistics (Côte d'Ivoire). Côte d'Ivoire Demographic and Health Survey 2011-2012. Fairfax, United States: ICF International.	18533
Cote d'Ivoire	2016	Cote d'Ivoire Multiple Indicator Cluster Survey 2016	Survey microdata	Admin >1			X		X	X	X	X	National Institute of Statistics (Côte d'Ivoire), United Nations Children's Fund (UNICEF). Cote d'Ivoire Multiple Indicator Cluster Survey 2016. New York, United States: United Nations Children's Fund (UNICEF), 2018.	218611
Democratic Republic of the Congo	2001	Democratic Republic of the Congo Multiple Indicator Cluster Survey 2001	Survey microdata	Admin 1			X					X	Ministry of Planning and Reconstruction (Congo, DR), United Nations Children's Fund (UNICEF). Congo, DR Multiple Indicator Cluster Survey 2001. New York, United States: United Nations Children's Fund (UNICEF).	3161

Country	Years	Name	Type	Geographic Detail	Male Circumcision	STI Symptoms	Had Intercourse (Women)	Partner Away	Condom Last Time	Multiple Partners in Year (Men)	Multiple Partners in Year (Women)	In Union	Citation	NID*
Democratic Republic of the Congo	2007	Democratic Republic of the Congo Demographic and Health Survey 2007	Survey microdata	GPS	X	X	X	X	X	X	X	X	Macro International, Inc, Ministry of Planning (Congo, DR). Democratic Republic of the Congo Demographic and Health Survey 2007. Fairfax, United States: ICF International.	19381
Democratic Republic of the Congo	2010	Democratic Republic of the Congo Multiple Indicator Cluster Survey 2010	Survey microdata	GPS			X				X		National Statistical Institute (Congo, DR), Ministry of Planning (Congo, DR), United Nations Children's Fund (UNICEF). Congo, DR Multiple Indicator Cluster Survey 2010. New York, United States: United Nations Children's Fund (UNICEF).	26998
Democratic Republic of the Congo	2013	Democratic Republic of the Congo - Kinshasa Performance Monitoring and Accountabi	Survey microdata	Admin 1			X	X					Tulane University School of Public Health, University of Kinshasa School of Public Health and The Bill & Melinda Gates Institute for Population and Reproductive Health at The Johns Hopkins Bloomberg School of Public Health. Performance Monitoring and Accountability 2020 (PMA2020) Survey round 1, PMA2013/DRC-R1	257822

Country	Years	Name	Type	Geographic Detail	Male Circumcision	STI Symptoms	Had Intercourse (Women)	Partner Away	Condom Last Time	Multiple Partners in Year (Men)	Multiple Partners in Year (Women)	In Union	Citation	NID*
		lity 2020 Survey, Round 1 2013-2014											(Kinshasa). 2013. DRC and Baltimore, Maryland, USA.	
Democratic Republic of the Congo	2013-14	Democratic Republic of the Congo Demographic and Health Survey 2013-2014	Survey microdata	GPS		X	X	X	X	X	X	X	ICF International, Ministry of Planning and Monitoring Implementation of the Revolution of Modernity (Congo, DR), Ministry of Public Health (Congo, DR), National Institute of Statistics (Congo, DR). Democratic Republic of the Congo Demographic and Health Survey 2013-2014. Fairfax, United States: ICF International, 2014.	76878
Democratic Republic of the Congo	2014	Democratic Republic of the Congo - Kinshasa Performance Monitoring and Accountability 2020 Survey,	Survey microdata	GPS			X	X					Tulane University School of Public Health, University of Kinshasa School of Public Health and The Bill & Melinda Gates Institute for Population and Reproductive Health at The Johns Hopkins Bloomberg School of Public Health. Performance Monitoring and Accountability 2020 (PMA2020) Survey round 2, PMA2014/DRC-R2 (Kinshasa). 2014. Kinshasa, DRC and Baltimore, Maryland, USA.	257823

Country	Years	Name	Type	Geographic Detail	Male Circumcision	STI Symptoms	Had Intercourse (Women)	Partner Away	Condom Last Time	Multiple Partners in Year (Men)	Multiple Partners in Year (Women)	In Union	Citation	NID*
		Round 2 2014												
Democratic Republic of the Congo	2015	Democratic Republic of the Congo - Kinshasa Performance Monitoring and Accountability 2020 Survey, Round 3 2015	Survey microdata	GPS			X	X					Tulane University School of Public Health, University of Kinshasa School of Public Health and The Bill & Melinda Gates Institute for Population and Reproductive Health at The Johns Hopkins Bloomberg School of Public Health. Performance Monitoring and Accountability 2020 (PMA2020) Survey round 3, PMA2015/DRC-R3 (Kinshasa). 2015. Kinshasa, DRC and Baltimore, Maryland, USA.	257826
Democratic Republic of the Congo	2015	Democratic Republic of the Congo - Kinshasa and Kongo Central Performance Monitoring and	Survey microdata	GPS			X	X					Tulane University School of Public Health, University of Kinshasa School of Public Health and The Bill & Melinda Gates Institute for Population and Reproductive Health at The Johns Hopkins Bloomberg School of Public Health. Performance Monitoring and Accountability 2020 (PMA2020) Survey round 4, PMA2015/DRC-R4 (Kinshasa & Kongo Central). 2015.	286019

Country	Years	Name	Type	Geographic Detail	Male Circumcision	STI Symptoms	Had Intercourse (Women)	Partner Away	Condom Last Time	Multiple Partners in Year (Men)	Multiple Partners in Year (Women)	In Union	Citation	NID*
		Accountability 2020 Survey, Round 4 2015-2016											Kinshasa, DRC and Baltimore, Maryland, USA.	
Democratic Republic of the Congo	2016	Democratic Republic of the Congo - Kinshasa and Kongo Central Performance Monitoring and Accountability 2020 Survey, Round 5 2016	Survey microdata	GPS			X	X					Tulane University School of Public Health, University of Kinshasa School of Public Health and The Bill & Melinda Gates Institute for Population and Reproductive Health at The Johns Hopkins Bloomberg School of Public Health. Performance Monitoring and Accountability 2020 (PMA2020) Survey round 5, PMA2016/DRC-R5 (Kinshasa & Kongo Central). 2016. Kinshasa, DRC and Baltimore, Maryland, USA.	286054
Equatorial Guinea	2000	Equatorial Guinea Multiple Indicator Cluster	Survey microdata	Admin 1								X	Ministry of Planning, Economic Development and Public Investment (Equatorial Guinea), United Nations Children's Fund (UNICEF). Equatorial Guinea Multiple Indicator Cluster	3655

Country	Years	Name	Type	Geographic Detail	Male Circumcision	STI Symptoms	Had Intercourse (Women)	Partner Away	Condom Last Time	Multiple Partners in Year (Men)	Multiple Partners in Year (Women)	In Union	Citation	NID*
		Survey 2000											Survey 2000. New York, United States: United Nations Children's Fund (UNICEF).	
Eritrea	2002	Eritrea Demographic and Health Survey 2002	Survey microdata	Admin 1			X	X				X	Macro International, Inc, National Statistics and Evaluation Office (Eritrea). Eritrea Demographic and Health Survey 2002. Calverton, United States: Macro International, Inc.	19539†
Eritrea	2010	Eritrea Population and Health Survey 2010	Survey report	Admin 1	X								Kenya Medical Research Institute (KEMRI), National Statistics Office (Eritrea), The Fafo Research Foundation. Eritrea Population and Health Survey 2010.	249999
Ethiopia	2000	Ethiopia Demographic and Health Survey 2000	Survey microdata	GPS			X	X	X	X	X	X	Central Statistical Agency (Ethiopia), ORC Macro. Ethiopia Demographic and Health Survey 2000. Calverton, United States: ORC Macro, 2001.	19571
Ethiopia	2005	Ethiopia Demographic and Health	Survey microdata	GPS	X	X	X	X	X	X	X	X	Macro International, Inc, Population and Housing Census Commissions Office (PHCCO). Ethiopia Demographic and Health Survey 2005. Fairfax, United States: ICF International.	19557

Country	Years	Name	Type	Geographic Detail	Male Circumcision	STI Symptoms	Had Intercourse (Women)	Partner Away	Condom Last Time	Multiple Partners in Year (Men)	Multiple Partners in Year (Women)	In Union	Citation	NID*
		Survey 2005												
Ethiopia	2010-11	Ethiopia Demographic and Health Survey 2010-2011	Survey microdata	GPS	X	X	X	X	X	X	X	X	Central Statistical Agency (Ethiopia), ICF Macro, Ministry of Health (Ethiopia). Ethiopia Demographic and Health Survey 2010-2011. Fairfax, United States: ICF International.	21301
Ethiopia	2014	Ethiopia Performance Monitoring and Accountability 2020 Survey, Round 1 2014	Survey microdata	Admin 1			X	X				X	Addis Ababa University School of Public Health and The Bill & Melinda Gates Institute for Population and Reproductive Health at The Johns Hopkins Bloomberg School of Public Health. Performance Monitoring and Accountability 2020 (PMA2020) Survey round 1, PMA2014/Ethiopia-R1. 2014. Ethiopia and Baltimore, Maryland, USA.	153503
Ethiopia	2014	Ethiopia Performance Monitoring and Accountability 2020	Survey microdata	Admin 1			X	X				X	Addis Ababa University School of Public Health and The Bill & Melinda Gates Institute for Population and Reproductive Health at The Johns Hopkins Bloomberg School of Public Health. Performance Monitoring and Accountability 2020 (PMA2020)	256175

Country	Years	Name	Type	Geographic Detail	Male Circumcision	STI Symptoms	Had Intercourse (Women)	Partner Away	Condom Last Time	Multiple Partners in Year (Men)	Multiple Partners in Year (Women)	In Union	Citation	NID*
		Survey, Round 2 2014											Survey round 2, PMA2014/Ethiopia-R2. 2014. Ethiopia and Baltimore, Maryland, USA.	
Ethiopia	2015	Ethiopia Performance Monitoring and Accountability 2020 Survey, Round 3 2015	Survey microdata	Admin 1			X	X				X	Addis Ababa University School of Public Health and The Bill & Melinda Gates Institute for Population and Reproductive Health at The Johns Hopkins Bloomberg School of Public Health. Performance Monitoring and Accountability 2020 (PMA2020) Survey round 3, PMA2015/Ethiopia-R3. 2015. Ethiopia and Baltimore, Maryland, USA.	256176
Ethiopia	2016	Ethiopia Demographic and Health Survey 2016	Survey microdata	GPS	X	X	X	X	X	X	X	X	Central Statistical Agency (Ethiopia), ICF International. Ethiopia Demographic and Health Survey 2016. Fairfax, United States: ICF International, 2017.	218568
Ethiopia	2016	Ethiopia Performance Monitoring and Accountabi	Survey microdata	Admin 1			X	X				X	Addis Ababa University School of Public Health and The Bill & Melinda Gates Institute for Population and Reproductive Health at The Johns Hopkins Bloomberg School of Public Health. Performance Monitoring and	285891

Country	Years	Name	Type	Geographic Detail	Male Circumcision	STI Symptoms	Had Intercourse (Women)	Partner Away	Condom Last Time	Multiple Partners in Year (Men)	Multiple Partners in Year (Women)	In Union	Citation	NID*
		lity 2020 Survey, Round 4 2016											Accountability 2020 (PMA2020) Survey round 4, PMA2016/Ethiopia-R4. 2016. Ethiopia and Baltimore, Maryland, USA.PMA2020) Survey round 3, PMA2016/Burkina Faso-R3. 2016. Ouagadougou, Burkina Faso and Baltimore, Maryland, USA.	
Ethiopia	2017	Ethiopia Performance Monitoring and Accountability 2020 Survey, Round 5 2017	Survey microdata	Admin 1			X	X				X	Addis Ababa University School of Public Health and The Bill & Melinda Gates Institute for Population and Reproductive Health at The Johns Hopkins Bloomberg School of Public Health. Performance Monitoring and Accountability 2020 (PMA2020) Survey round 5, PMA2017/Ethiopia-R5. 2017. Ethiopia and Baltimore, Maryland, USA.	347050
Gabon	2000	Gabon Demographic and Health Survey 2000-2001	Survey microdata	Admin 2			X	X	X			X	General Directorate of Statistics and Economic Studies (Gabon), Macro International, Inc. Gabon Demographic and Health Survey 2000-2001. Fairfax, United States: ICF International.	19579
Gabon	2012	Gabon Demograp	Survey microdata	GPS	X	X	X	X	X	X	X	X	General Directorate of Statistics (Gabon), ICF International, Ministry of	76706

Country	Years	Name	Type	Geographic Detail	Male Circumcision	STI Symptoms	Had Intercourse (Women)	Partner Away	Condom Last Time	Multiple Partners in Year (Men)	Multiple Partners in Year (Women)	In Union	Citation	NID*
		hic and Health Survey 2012											Economy, Employment and Sustainable Development (Gabon), Ministry of Health (Gabon). Gabon Demographic and Health Survey 2012. Fairfax, United States: ICF International, 2013.	
Ghana	2003	Ghana Demographic and Health Survey 2003	Survey microdata	GPS	X	X	X	X	X	X	X	X	Ghana Statistical Service, Macro International, Inc. Ghana Demographic and Health Survey 2003. Fairfax, United States: ICF International.	19627
Ghana	2006	Ghana Multiple Indicator Cluster Survey 2006	Survey microdata	Admin 1			X		X	X	X	X	Ministry of Health (MOH) (Ghana), Ghana Statistical Service and United Nations Children's Fund (UNICEF). Ghana Multiple Indicator Cluster Survey 2006. New York, United States: United Nations Children's Fund (UNICEF).	4694
Ghana	2008	Ghana Demographic and Health Survey 2008	Survey microdata	GPS	X	X	X	X	X	X	X	X	Ghana Statistical Service, Macro International, Inc, Ministry of Health (Ghana). Ghana Demographic and Health Survey 2008. Fairfax, United States: ICF International.	21188

Country	Years	Name	Type	Geographic Detail	Male Circumcision	STI Symptoms	Had Intercourse (Women)	Partner Away	Condom Last Time	Multiple Partners in Year (Men)	Multiple Partners in Year (Women)	In Union	Citation	NID*
Ghana	2010	Ghana - Accra Multiple Indicator Cluster Survey 2010-2011	Survey microdata	GPS			X		X	X	X	X	Institute of Statistical, Social and Economic Research, University of Ghana, United Nations Children's Fund (UNICEF). Ghana - Accra Multiple Indicator Cluster Survey 2010-2011. New York, United States: United Nations Children's Fund (UNICEF), 2014.	56241
Ghana	2011	Ghana Multiple Indicator Cluster Survey 2011	Survey microdata	GPS			X		X	X	X	X	Centers for Disease Control and Prevention (CDC), Ghana Statistical Service, Government of Japan, ICF Macro, Ministry of Health (Ghana), Navrongo Health Research Centre, United Nations Children's Fund (UNICEF), United Nations Population Fund (UNFPA), United States Agency for International Development (USAID). Ghana Multiple Indicator Cluster Survey 2011. New York, United States: United Nations Children's Fund (UNICEF), 2013.	63993
Ghana	2013	Ghana Living Standards Measurem	Survey microdata	Admin 1								X	Ghana Statistical Service, World Bank. Ghana Living Standards Measurement Survey 2012-2013. Accra, Ghana: Ghana Statistical Service.	165101

Country	Years	Name	Type	Geographic Detail	Male Circumcision	STI Symptoms	Had Intercourse (Women)	Partner Away	Condom Last Time	Multiple Partners in Year (Men)	Multiple Partners in Year (Women)	In Union	Citation	NID*
		ent Survey 2012-2013												
Ghana	2013	Ghana Performance Monitoring and Accountability 2020 Survey, Round 1 2013	Survey microdata	Admin 1			X	X				X	Kwame Nkrumah University of Science & Technology School of Medicine and The Bill & Melinda Gates Institute for Population and Reproductive Health at The Johns Hopkins Bloomberg School of Public Health. Performance Monitoring and Accountability 2020 (PMA2020) Survey round 1, PMA2013/Ghana-R1. 2013. Ghana and Baltimore, Maryland, USA.	197904
Ghana	2014	Ghana Demographic and Health Survey 2014	Survey microdata	GPS	X	X	X	X	X	X	X	X	Ghana Health Service, Ghana Statistical Service, ICF International. Ghana Demographic and Health Survey 2014. Fairfax, United States: ICF International, 2016.	157027
Ghana	2014	Ghana Performance Monitoring and Accountability 2020	Survey microdata	Admin 1			X	X				X	Kwame Nkrumah University of Science & Technology School of Medicine and The Bill & Melinda Gates Institute for Population and Reproductive Health at The Johns Hopkins Bloomberg School of Public Health. Performance Monitoring and Accountability 2020	256241

Country	Years	Name	Type	Geographic Detail	Male Circumcision	STI Symptoms	Had Intercourse (Women)	Partner Away	Condom Last Time	Multiple Partners in Year (Men)	Multiple Partners in Year (Women)	In Union	Citation	NID*
		Survey, Round 2 2014											(PMA2020) Survey round 2, PMA2014/Ghana-R2. 2014. Ghana and Baltimore, Maryland, USA.	
Ghana	2014	Ghana Performance Monitoring and Accountability 2020 Survey, Round 3 2014	Survey microdata	Admin 1			X	X				X	Kwame Nkrumah University of Science & Technology School of Medicine and The Bill & Melinda Gates Institute for Population and Reproductive Health at The Johns Hopkins Bloomberg School of Public Health. Performance Monitoring and Accountability 2020 (PMA2020) Survey round 3, PMA2014/Ghana-R3. 2014. Ghana and Baltimore, Maryland, USA.	256243
Ghana	2015	Ghana Performance Monitoring and Accountability 2020 Survey, Round 4 2015	Survey microdata	Admin 1			X	X				X	Bill and Melinda Gates Institute for Population and Reproductive Health, Johns Hopkins Bloomberg School of Public Health, Ghana Health Service, Ghana Statistical Service, Kwame Nkrumah University of Science and Technology (KNUST), University for Development Studies (Ghana). Ghana Performance Monitoring and Accountability 2020 Survey, Round 4 2015. Baltimore, United States: Bill and Melinda Gates Institute for	256244

Country	Years	Name	Type	Geographic Detail	Male Circumcision	STI Symptoms	Had Intercourse (Women)	Partner Away	Condom Last Time	Multiple Partners in Year (Men)	Multiple Partners in Year (Women)	In Union	Citation	NID*
													Population and Reproductive Health, Johns Hopkins Bloomberg School of Public Health.	
Ghana	2016	Ghana Performance Monitoring and Accountability 2020 Survey, Round 5 2016	Survey microdata	Admin 1			X	X				X	Kwame Nkrumah University of Science & Technology School of Medicine and The Bill & Melinda Gates Institute for Population and Reproductive Health at The Johns Hopkins Bloomberg School of Public Health. Performance Monitoring and Accountability 2020 (PMA2020) Survey round 5, PMA2016/Ghana-R5. 2016. Ghana and Baltimore, Maryland, USA.	286146
Ghana	2017	Ghana Special Demographic and Health Survey 2017	Survey microdata	GPS				X					Ghana Health Service, Ghana Statistical Service, ICF International. Ghana Special Demographic and Health Survey 2017. Fairfax, United States: ICF International, 2018.	218572
Guinea	2005	Guinea Demographic and Health	Survey microdata	GPS	X	X	X	X	X	X	X	X	Macro International, Inc, National Statistics Directorate (Guinea). Guinea Demographic and Health Survey 2005. Fairfax, United States: ICF International.	19683

Country	Years	Name	Type	Geographic Detail	Male Circumcision	STI Symptoms	Had Intercourse (Women)	Partner Away	Condom Last Time	Multiple Partners in Year (Men)	Multiple Partners in Year (Women)	In Union	Citation	NID*
		Survey 2005												
Guinea	2012	Guinea Demographic and Health Survey 2012	Survey microdata	GPS		X	X	X	X	X	X	X	ICF Macro, Ministry of Health and Public Hygiene (Guinea), National Institute of Statistics (Guinea). Guinea Demographic and Health Survey 2012. Fairfax, United States: ICF International.	69761
Guinea	2016	Guinea Multiple Indicator Cluster Survey 2016	Survey microdata	Admin 1			X				X		National Institute of Public Health (NPHI) (Guinea), National Institute of Statistics (Guinea), National Malaria Control Program (Guinea), United Nations Children's Fund (UNICEF). Guinea Multiple Indicator Cluster Survey 2016. New York, United States: United Nations Children's Fund (UNICEF), 2018.	303458
Guinea-Bissau	2006	Guinea-Bissau Multiple Indicator Cluster Survey 2006	Survey microdata	Admin 1			X				X		United Nations Children's Fund (UNICEF), Government of Guinea-Bissau. Guinea-Bissau Multiple Indicator Cluster Survey 2006. New York, United States: United Nations Children's Fund (UNICEF).	4818

Country	Years	Name	Type	Geographic Detail	Male Circumcision	STI Symptoms	Had Intercourse (Women)	Partner Away	Condom Last Time	Multiple Partners in Year (Men)	Multiple Partners in Year (Women)	In Union	Citation	NID*
Guinea-Bissau	2014	Guinea-Bissau Multiple Indicator Cluster Survey 2014	Survey microdata	Admin 1	X		X		X	X	X	X	National Statistics Institute (Guinea-Bissau), United Nations Children's Fund (UNICEF). Guinea-Bissau Multiple Indicator Cluster Survey 2014. New York, United States: United Nations Children's Fund (UNICEF), 2016.	174049
Kenya	2000	Kenya Multiple Indicator Cluster Survey 2000	Survey microdata	GPS								X	Central Bureau of Statistics (Kenya), United Nations Children's Fund (UNICEF). Kenya Multiple Indicator Cluster Survey 2000. New York, United States: United Nations Children's Fund (UNICEF).	7387
Kenya	2003	Kenya Demographic and Health Survey 2003	Survey microdata	GPS	X	X	X	X	X	X	X	X	Centers for Disease Control and Prevention (CDC), Central Bureau of Statistics (Kenya), Macro International, Inc, Ministry of Health (Kenya), National Council for Population and Development (Kenya). Kenya Demographic and Health Survey 2003. Fairfax, United States: ICF International.	20145
Kenya	2007	Kenya AIDS Indicator	Survey microdata	GPS	X		X	X	X	X	X	X	Centers for Disease Control and Prevention (CDC), Kenya Medical Research Institute (KEMRI), Kenya	133219

Country	Years	Name	Type	Geographic Detail	Male Circumcision	STI Symptoms	Had Intercourse (Women)	Partner Away	Condom Last Time	Multiple Partners in Year (Men)	Multiple Partners in Year (Women)	In Union	Citation	NID*
		Survey 2007											National Bureau of Statistics, Ministry of Public Health and Sanitation (Kenya), National AIDS Control Council (Kenya), National AIDS and STI Control Program (Kenya), National Coordinating Agency for Population and Development (Kenya), National Public Health Laboratory Services, Ministry of Public Health and Sanitation (Kenya), United States Agency for International Development (USAID). Kenya AIDS Indicator Survey 2007. Nairobi, Kenya: Kenya National Bureau of Statistics.	
Kenya	2007	Kenya Household Health Expenditure and Utilization Survey 2007	Survey microdata	Admin 1								X	Abt Associates Inc., Kenya National Bureau of Statistics, Ministry of Health (Kenya). Kenya Household Health Expenditure and Utilization Survey 2007. Nairobi, Kenya: Kenya National Bureau of Statistics.	157635
Kenya	2008-09	Kenya Demographic and	Survey microdata	GPS	X	X	X	X	X	X	X	X	ICF Macro, Kenya Medical Research Institute (KEMRI), Kenya National Bureau of Statistics, Ministry of Public	21365

Country	Years	Name	Type	Geographic Detail	Male Circumcision	STI Symptoms	Had Intercourse (Women)	Partner Away	Condom Last Time	Multiple Partners in Year (Men)	Multiple Partners in Year (Women)	In Union	Citation	NID*
		Health Survey 2008-2009											Health and Sanitation (Kenya), National AIDS and STI Control Program (Kenya), National Aids Control Council (NACC), National Coordinating Agency for Population and Development (Kenya). Kenya Demographic and Health Survey 2008-2009. Fairfax, United States: ICF International.	
Kenya	2009	Kenya - Coast Multiple Indicator Cluster Survey 2009	Survey microdata	Admin 3			X				X		Kenya National Bureau of Statistics, United Nations Children's Fund (UNICEF). Kenya - Coast Multiple Indicator Cluster Survey 2009. New York, United States: United Nations Children's Fund (UNICEF), 2014.	56420
Kenya	2011	Kenya - Nyanza Province Multiple Indicator Cluster Survey 2011	Survey microdata	GPS			X				X		Kenya National Bureau of Statistics, United Nations Children's Fund (UNICEF). Kenya - Nyanza Province Multiple Indicator Cluster Survey 2011. Nairobi, Kenya: Kenya National Bureau of Statistics.	135416

Country	Years	Name	Type	Geographic Detail	Male Circumcision	STI Symptoms	Had Intercourse (Women)	Partner Away	Condom Last Time	Multiple Partners in Year (Men)	Multiple Partners in Year (Women)	In Union	Citation	NID*
Kenya	2013	Kenya AIDS Indicator Survey 2012-2013	Survey microdata	GPS	X	X	X	X		X	X	X	Kenya National Bureau of Statistics, Ministry of Devolution and Planning (Kenya), Ministry of Health (Kenya), National AIDS and STI Control Program (Kenya). Kenya AIDS Indicator Survey 2012-2013. Nairobi, Kenya: Kenya National Bureau of Statistics.	133304
Kenya	2013-14	Kenya - Bungoma County Multiple Indicator Survey 2013-2014	Survey microdata	GPS			X						Kenya National Bureau of Statistics, Population Studies and Research Institute, University of Nairobi (Kenya), United Nations Children's Fund (UNICEF). Kenya - Bungoma County Multiple Indicator Survey 2013-2014. New York, United States: United Nations Children's Fund (UNICEF), 2015.	203654
Kenya	2013-14	Kenya - Kakamega County Multiple Indicator Survey 2013-2014	Survey microdata	GPS			X				X		Kenya National Bureau of Statistics, Population Studies and Research Institute, University of Nairobi (Kenya), United Nations Children's Fund (UNICEF). Kenya - Kakamega County Multiple Indicator Survey 2013-2014. New York, United States:	203663

Country	Years	Name	Type	Geographic Detail	Male Circumcision	STI Symptoms	Had Intercourse (Women)	Partner Away	Condom Last Time	Multiple Partners in Year (Men)	Multiple Partners in Year (Women)	In Union	Citation	NID*
													United Nations Children's Fund (UNICEF), 2015.	
Kenya	2013-14	Kenya - Turkana County Multiple Indicator Survey 2013-2014	Survey microdata	GPS			X				X		Kenya National Bureau of Statistics, Population Studies and Research Institute, University of Nairobi (Kenya), United Nations Children's Fund (UNICEF). Kenya - Turkana County Multiple Indicator Survey 2013-2014. New York, United States: United Nations Children's Fund (UNICEF), 2015.	203664
Kenya	2014	Kenya Demographic and Health Survey 2014	Survey microdata	GPS	X	X	X	X	X	X	X	X	ICF International, Kenya Medical Research Institute (KEMRI), Kenya National Bureau of Statistics, Ministry of Health (Kenya), National AIDS Control Council (Kenya), National Council for Population and Development (Kenya). Kenya Demographic and Health Survey 2014. Fairfax, United States: ICF International.	157057
Kenya	2014	Kenya Performance Monitoring	Survey microdata	Admin 2			X	X				X	International Centre for Reproductive Health Kenya (ICRHK) and The Bill & Melinda Gates Institute for Population and Reproductive Health at The Johns	197910

Country	Years	Name	Type	Geographic Detail	Male Circumcision	STI Symptoms	Had Intercourse (Women)	Partner Away	Condom Last Time	Multiple Partners in Year (Men)	Multiple Partners in Year (Women)	In Union	Citation	NID*
		and Accountability 2020 Survey, Round 1 2014											Hopkins Bloomberg School of Public Health. Performance Monitoring and Accountability 2020 (PMA2020) Survey round 1, PMA2014/Kenya-R1. 2014. Kenya and Baltimore, Maryland, USA.	
Kenya	2014	Kenya Performance Monitoring and Accountability 2020 Survey, Round 2 2014	Survey microdata	Admin 2			X	X				X	Bill and Melinda Gates Institute for Population and Reproductive Health, Johns Hopkins Bloomberg School of Public Health, International Center for Reproductive Health (Kenya), Kenya National Bureau of Statistics, Ministry of Health (Kenya), National Council for Population and Development (Kenya). Kenya Performance Monitoring and Accountability 2020 Survey, Round 2 2014. Baltimore, United States: Johns Hopkins Bloomberg School of Public Health.	256338
Kenya	2015	Kenya Performance Monitoring and Accountabi	Survey microdata	Admin 2			X	X				X	International Centre for Reproductive Health Kenya (ICRHK) and The Bill & Melinda Gates Institute for Population and Reproductive Health at The Johns Hopkins Bloomberg School of Public Health. Performance Monitoring and	256365

Country	Years	Name	Type	Geographic Detail	Male Circumcision	STI Symptoms	Had Intercourse (Women)	Partner Away	Condom Last Time	Multiple Partners in Year (Men)	Multiple Partners in Year (Women)	In Union	Citation	NID*
		lity 2020 Survey, Round 3 2015											Accountability 2020 (PMA2020) Survey round 3, PMA2015/Kenya-R3. 2015. Kenya and Baltimore, Maryland, USA.	
Kenya	2015	Kenya Performance Monitoring and Accountability 2020 Survey, Round 4 2015	Survey microdata	Admin 2			X	X				X	International Centre for Reproductive Health Kenya (ICRHK) and The Bill & Melinda Gates Institute for Population and Reproductive Health at The Johns Hopkins Bloomberg School of Public Health. Performance Monitoring and Accountability 2020 (PMA2020) Survey round 4, PMA2015/Kenya-R4. 2015. Kenya and Baltimore, Maryland, USA.	256366
Kenya	2016	Kenya Performance Monitoring and Accountability 2020 Survey, Round 5 2016	Survey microdata	Admin 2			X	X				X	International Centre for Reproductive Health Kenya (ICRHK) and The Bill & Melinda Gates Institute for Population and Reproductive Health at The Johns Hopkins Bloomberg School of Public Health. Performance Monitoring and Accountability 2020 (PMA2020) Survey round 5, PMA2016/Kenya-R5. 2016. Kenya and Baltimore, Maryland, USA.	347047

Country	Years	Name	Type	Geographic Detail	Male Circumcision	STI Symptoms	Had Intercourse (Women)	Partner Away	Condom Last Time	Multiple Partners in Year (Men)	Multiple Partners in Year (Women)	In Union	Citation	NID*
Lesotho	2000	Lesotho Multiple Indicator Cluster Survey 2000	Survey microdata	Admin 1								X	Bureau of Statistics (Lesotho), United Nations Children's Fund (UNICEF). Lesotho Multiple Indicator Cluster Survey 2000. New York, United States: United Nations Children's Fund (UNICEF).	7721
Lesotho	2004-05	Lesotho Demographic and Health Survey 2004-2005	Survey microdata	GPS	X	X	X	X		X	X	X	Bureau of Statistics (Lesotho), Macro International, Inc, Ministry of Health and Social Welfare (Lesotho). Lesotho Demographic and Health Survey 2004-2005. Fairfax, United States: ICF International.	20167
Lesotho	2009-10	Lesotho Demographic and Health Survey 2009-2010	Survey microdata	GPS	X	X	X	X	X	X	X	X	ICF Macro, Ministry of Health and Social Welfare (Lesotho). Lesotho Demographic and Health Survey 2009-2010. Fairfax, United States: ICF International.	21382
Lesotho	2014	Lesotho Demographic and Health Survey 2014	Survey microdata	GPS	X	X	X	X	X	X	X	X	ICF International, Ministry of Health and Social Welfare (Lesotho). Lesotho Demographic and Health Survey 2014. Fairfax, United States: ICF International.	157058

Country	Years	Name	Type	Geographic Detail	Male Circumcision	STI Symptoms	Had Intercourse (Women)	Partner Away	Condom Last Time	Multiple Partners in Year (Men)	Multiple Partners in Year (Women)	In Union	Citation	NID*
Liberia	2006-07	Liberia Demographic and Health Survey 2006-2007	Survey microdata	GPS	X	X	X	X	X	X	X	X	Liberia Institute for Statistics and Geo-information Services (LISGIS), Macro International, Inc. Liberia Demographic and Health Survey 2006-2007. Fairfax, United States: ICF International.	20191
Liberia	2013	Liberia Demographic and Health Survey 2013	Survey microdata	GPS	X	X	X	X	X	X	X	X	ICF International, Liberia Institute for Statistics and Geo-information Services (LISGIS), National AIDS and STI Control Program (NACP), Ministry of Health and Social Welfare (Liberia). Liberia Demographic and Health Survey 2013. Fairfax, United States: ICF International.	77385
Madagascar	2000	Madagascar Multiple Indicator Cluster Survey 2000	Survey microdata	Admin 1			X						National Institute of Statistics (Madagascar), United Nations Children's Fund (UNICEF). Madagascar Multiple Indicator Cluster Survey 2000. New York, United States: United Nations Children's Fund (UNICEF).	27020
Madagascar	2003-04	Madagascar Demographic and Health	Survey microdata	Admin 2	X	X	X	X	X	X	X	X	Macro International, Inc, National Institute of Statistics (Madagascar). Madagascar Demographic and Health Survey 2003-2004. Fairfax, United States: ICF International.	20223

Country	Years	Name	Type	Geographic Detail	Male Circumcision	STI Symptoms	Had Intercourse (Women)	Partner Away	Condom Last Time	Multiple Partners in Year (Men)	Multiple Partners in Year (Women)	In Union	Citation	NID*
		Survey 2003-2004												
Madagascar	2008-09	Madagascar Demographic and Health Survey 2008-2009	Survey microdata	GPS	X	X	X	X	X	X	X	X	ICF Macro, National Institute of Statistics (Madagascar). Madagascar Demographic and Health Survey 2008-2009. Fairfax, United States: ICF International.	21409
Madagascar	2012	Madagascar - South Multiple Indicator Cluster Survey 2012	Survey microdata	GPS			X				X		National Institute of Statistics (Madagascar), United Nations Children's Fund (UNICEF). Madagascar - South Multiple Indicator Cluster Survey 2012. New York, United States: United Nations Children's Fund (UNICEF), 2015.	125594
Malawi	2000	Malawi Demographic and Health Survey 2000	Survey microdata	GPS		X	X	X	X	X	X	X	Macro International, Inc, National Statistical Office of Malawi. Malawi Demographic and Health Survey 2000. Fairfax, United States: ICF International.	20252
Malawi	2004-05	Malawi Demographic and	Survey microdata	GPS	X	X	X	X	X	X	X	X	Macro International, Inc, National Statistical Office of Malawi. Malawi Demographic and Health Survey 2004-	20263

Country	Years	Name	Type	Geographic Detail	Male Circumcision	STI Symptoms	Had Intercourse (Women)	Partner Away	Condom Last Time	Multiple Partners in Year (Men)	Multiple Partners in Year (Women)	In Union	Citation	NID*
		Health Survey 2004-2005											2005. Fairfax, United States: ICF International.	
Malawi	2006	Malawi Multiple Indicator Cluster Survey 2006	Survey microdata	Admin 2			X		X	X	X	X	United Nations Children's Fund (UNICEF), National Statistics Office (Malawi). Malawi Multiple Indicator Cluster Survey 2006. New York, United States: United Nations Children's Fund (UNICEF).	7919
Malawi	2010	Malawi Demograp hic and Health Survey 2010	Survey microdata	GPS	X	X	X	X	X	X	X	X	ICF Macro, National Statistical Office of Malawi. Malawi Demographic and Health Survey 2010. Fairfax, United States: ICF International.	21393
Malawi	2013-14	Malawi Multiple Indicator Cluster Survey 2013-2014	Survey microdata	Admin 2	X		X		X	X	X	X	National Statistical Office of Malawi, United Nations Children's Fund (UNICEF). Malawi Multiple Indicator Cluster Survey 2013-2014. New York, United States: United Nations Children's Fund (UNICEF), 2015.	161662
Malawi	2015-16	Malawi Demograp hic and Health	Survey microdata	GPS	X	X	X	X	X	X	X	X	Emory University and Centers for Disease Control & Prevention Collaboration, ICF International, Ministry of Health (Malawi), National	218581

Country	Years	Name	Type	Geographic Detail	Male Circumcision	STI Symptoms	Had Intercourse (Women)	Partner Away	Condom Last Time	Multiple Partners in Year (Men)	Multiple Partners in Year (Women)	In Union	Citation	NID*
		Survey 2015-2016											Statistical Office of Malawi. Malawi Demographic and Health Survey 2015-2016. Fairfax, United States: ICF International, 2017.	
Malawi	2016	Malawi Population-based HIV Impact Assessment 2015-2016	Survey report	Admin >1	X								Ministry of Health, Malawi, Centers for Disease Control and Prevention (CDC), and ICAP at Columbia University. Malawi Population-based HIV Impact Assessment (MPHIA) 2015-16: First Report. Lilongwe, Malawi, Atlanta, Georgia and New York, New York, USA: Ministry of Health, CDC and ICAP. December 2016.	287629
Malawi	2016-17	Malawi Integrated Household Survey 2016-2017	Survey microdata	Admin 2								X	National Statistical Office. Malawi - Fourth Integrated Household Survey 2016-2017, Ref. MWI_2016_IHS-IV_v02_M. Dataset downloaded from [http://microdata.worldbank.org/index.php/catalog/2936] on [December 18, 2017].	327852
Mali	2001	Mali Demographic and Health	Survey microdata	GPS		X	X	X	X	X	X	X	Macro International, Inc, National Directorate of Statistics and Informatics (DNSI) (Mali), Planning and Statistics Unit, Ministry of Health	20315

Country	Years	Name	Type	Geographic Detail	Male Circumcision	STI Symptoms	Had Intercourse (Women)	Partner Away	Condom Last Time	Multiple Partners in Year (Men)	Multiple Partners in Year (Women)	In Union	Citation	NID*
		Survey 2001											(Mali). Mali Demographic and Health Survey 2001. Fairfax, United States: ICF International.	
Mali	2006	Mali Demographic and Health Survey 2006	Survey microdata	GPS	X	X	X	X	X	X	X	X	Macro International, Inc, Ministry of Health (Mali), National Directorate of Statistics and Informatics (DNSI) (Mali). Mali Demographic and Health Survey 2006. Fairfax, United States: ICF International.	20274
Mali	2009-10	Mali Multiple Indicator Cluster Survey 2009-2010	Survey microdata	Admin 2			X				X		Ministry of Health (Mali), National Institute of Statistics (INSTAT) (Mali), United Nations Children's Fund (UNICEF). Mali Multiple Indicator Cluster Survey 2009-2010. New York, United States: United Nations Children's Fund (UNICEF), 2017.	270627
Mali	2012-13	Mali Demographic and Health Survey 2012-2013	Survey microdata	GPS	X	X	X	X	X	X	X	X	ICF International, INFO-STAT (Mali), Ministry of Health (Mali), National Institute of Statistics (INSTAT) (Mali), Planning and Statistics Unit, Ministry of Health (Mali). Mali Demographic and Health Survey 2012-2013. Fairfax, United States: ICF International, 2014.	77388
Mali	2015	Mali Multiple	Survey microdata	Admin 1			X		X	X	X	X	Ministry of Health (Mali), Ministry of Planning (Mali), National Institute of	248224

Country	Years	Name	Type	Geographic Detail	Male Circumcision	STI Symptoms	Had Intercourse (Women)	Partner Away	Condom Last Time	Multiple Partners in Year (Men)	Multiple Partners in Year (Women)	In Union	Citation	NID*
		Indicator Cluster Survey 2015											Statistics (INSTAT) (Mali), United Nations Children's Fund (UNICEF). Mali Multiple Indicator Cluster Survey 2015. New York, United States: United Nations Children's Fund (UNICEF), 2017.	
Mauritania	2000-01	Mauritania Demographic and Health Survey 2000-2001	Survey microdata	Admin 1			X	X		X		X	Macro International, Inc, National Office of Statistics (Mauritania). Mauritania Demographic and Health Survey 2000-2001. Calverton, United States: Macro International, Inc.	20322
Mauritania	2011	Mauritania Multiple Indicator Cluster Survey 2011	Survey microdata	Admin 3								X	National Office of Statistics (Mauritania), United Nations Children's Fund (UNICEF). Mauritania Multiple Indicator Cluster Survey 2011. New York, United States: United Nations Children's Fund (UNICEF), 2015.	152783
Mauritania	2015	Mauritania Multiple Indicator Cluster Survey 2015	Survey microdata	Admin 1								X	National Office of Statistics (Mauritania), United Nations Children's Fund (UNICEF). Mauritania Multiple Indicator Cluster Survey 2015. New York, United States: United	267343

Country	Years	Name	Type	Geographic Detail	Male Circumcision	STI Symptoms	Had Intercourse (Women)	Partner Away	Condom Last Time	Multiple Partners in Year (Men)	Multiple Partners in Year (Women)	In Union	Citation	NID*
													Nations Children's Fund (UNICEF), 2018.	
Mozambique	2001	Mozambique Young Adult Reproductive Health Survey 2001	Survey microdata	Admin 1			X						Mozambique National Institute of Statistics, Division of Reproductive Health-Centers for Disease Control and Prevention (CDC). (2003) Mozambique Young Adult Reproductive Health Survey 2001. Atlanta, United States: Centers for Disease Control and Prevention (CDC).	27519
Mozambique	2003	Mozambique Demographic and Health Survey 2003-2004	Survey microdata	Admin 1	X	X	X	X	X	X	X	X	Macro International, Inc, National Institute of Statistics (INE) (Mozambique). Mozambique Demographic and Health Survey 2003-2004. Fairfax, United States: ICF International.	20394
Mozambique	2008	Mozambique Multiple Indicator Cluster Survey 2008-2009	Survey microdata	Admin 1			X						United Nations Children's Fund (UNICEF), National Statistics Institute (Mozambique). Mozambique Multiple Indicator Cluster Survey 2008-2009. New York, United States: United Nations Children's Fund (UNICEF).	27031
Mozambique	2009	Mozambique AIDS	Survey microdata	GPS	X	X	X	X	X	X	X	X	ICF Macro, Ministry of Health (Mozambique), National Institute of	8906

Country	Years	Name	Type	Geographic Detail	Male Circumcision	STI Symptoms	Had Intercourse (Women)	Partner Away	Condom Last Time	Multiple Partners in Year (Men)	Multiple Partners in Year (Women)	In Union	Citation	NID*
		Indicator Survey 2009											Statistics (INE) (Mozambique). Mozambique AIDS Indicator Survey 2009. Fairfax, United States: ICF International, 2010.	
Mozambique	2011	Mozambique Demographic and Health Survey 2011	Survey microdata	GPS	X	X	X	X	X	X	X	X	ICF Macro, Manhica Health Research Center (CISM), Ministry of Health (Mozambique), National Institute of Statistics (INE) (Mozambique). Mozambique Demographic and Health Survey 2011. Fairfax, United States: ICF International.	55975
Mozambique	2015	Mozambique AIDS Indicator Survey 2015	Survey microdata	GPS	X	X	X	X	X	X	X	X	Centers for Disease Control and Prevention (CDC), ICF International, Ministry of Health (Mozambique), National Institute of Health (Mozambique), National Institute of Statistics (INE) (Mozambique). Mozambique AIDS Indicator Survey 2015. Fairfax, United States: ICF International, 2018.	157060
Namibia	2000	Namibia Demographic and Health	Survey microdata	GPS			X	X	X	X	X	X	Macro International, Inc, Ministry of Health and Social Services (Namibia), National Planning Commission (Namibia). Namibia Demographic and	20417

Country	Years	Name	Type	Geographic Detail	Male Circumcision	STI Symptoms	Had Intercourse (Women)	Partner Away	Condom Last Time	Multiple Partners in Year (Men)	Multiple Partners in Year (Women)	In Union	Citation	NID*
		Survey 2000											Health Survey 2000. Calverton, United States: Macro International, Inc.	
Namibia	2006-07	Namibia Demographic and Health Survey 2006-2007	Survey microdata	GPS	X	X	X	X	X	X	X	X	Macro International, Inc, Ministry of Health and Social Services (Namibia). Namibia Demographic and Health Survey 2006-2007. Fairfax, United States: ICF International.	20428
Namibia	2013	Namibia Demographic and Health Survey 2013	Survey microdata	GPS	X	X	X	X	X	X	X	X	ICF International, Ministry of Health and Social Services (Namibia), Namibia Institute of Pathology, Namibia Statistics Agency. Namibia Demographic and Health Survey 2013. Fairfax, United States: ICF International.	150382
Niger	2000	Niger Multiple Indicator Cluster Survey 2000	Survey microdata	Admin 1								X	Government of Niger, Macro International, Inc, United Nations Children's Fund (UNICEF). Niger Multiple Indicator Cluster Survey 2000. New York, United States: United Nations Children's Fund (UNICEF).	9439
Niger	2006	Niger Demographic and Health	Survey microdata	Admin 1	X	X	X	X	X	X	X	X	Department of Statistics and National Accounts (Niger), Macro International, Inc. Niger Demographic and Health	20499

Country	Years	Name	Type	Geographic Detail	Male Circumcision	STI Symptoms	Had Intercourse (Women)	Partner Away	Condom Last Time	Multiple Partners in Year (Men)	Multiple Partners in Year (Women)	In Union	Citation	NID*
		Survey 2006											Survey 2006. Fairfax, United States: ICF International.	
Niger	2012	Niger Demographic and Health Survey 2012	Survey microdata	Admin 1		X	X	X	X	X	X	X	ICF International, Ministry of Public Health (Niger), National Institute of Statistics (Niger). Niger Demographic and Health Survey 2012. Fairfax, United States: ICF International.	74393
Niger	2015	Niger - Niamey Performance Monitoring and Accountability 2020 Survey, Round 1 2015	Survey microdata	Admin 1			X	X				X	Niger/Niamey Institut National de la Statistique (National Institute of Statistics) and The Bill & Melinda Gates Institute for Population and Reproductive Health at The Johns Hopkins Bloomberg School of Public Health. Performance Monitoring and Accountability 2020 (PMA2020) Survey round 1, PMA2015/Niger-R1 (Niamey). 2015. Niamey, Niger and Baltimore, Maryland, USA.	256177
Niger	2016	Niger Performance Monitoring and Accountabi	Survey microdata	Admin 1			X	X				X	Niger/Niamey Institut National de la Statistique (National Institute of Statistics) and The Bill & Melinda Gates Institute for Population and Reproductive Health at The Johns Hopkins Bloomberg School of Public	286052

Country	Years	Name	Type	Geographic Detail	Male Circumcision	STI Symptoms	Had Intercourse (Women)	Partner Away	Condom Last Time	Multiple Partners in Year (Men)	Multiple Partners in Year (Women)	In Union	Citation	NID*
		lity 2020 Survey, Round 2 2016											Health. Performance Monitoring and Accountability 2020 (PMA2020) Survey round 2, PMA2016/Niger-R2 (National). 2016. Niamey, Niger and Baltimore, Maryland, USA.	
Nigeria	2003	Nigeria Demographic and Health Survey 2003	Survey microdata	GPS		X	X	X	X	X	X	X	Department for International Development (DFiD) (United Kingdom), National Population Commission of Nigeria, ORC Macro, United Nations Children's Fund (UNICEF), United Nations Population Fund (UNFPA). Nigeria Demographic and Health Survey 2003. Fairfax, United States: ICF International.	20567
Nigeria	2004	Nigeria Living Standards Survey 2003-2004	Survey microdata	Admin 1								X	Federal Office of Statistics (Nigeria). Nigeria Living Standards Survey 2003-2004.	25006
Nigeria	2006	Nigeria Core Welfare Indicators Questionna	Survey microdata	Admin 1	X							X	National Bureau of Statistics (Nigeria). Nigeria Core Welfare Indicators Questionnaire Survey 2006. Abuja, Nigeria: National Bureau of Statistics (Nigeria).	9522

Country	Years	Name	Type	Geographic Detail	Male Circumcision	STI Symptoms	Had Intercourse (Women)	Partner Away	Condom Last Time	Multiple Partners in Year (Men)	Multiple Partners in Year (Women)	In Union	Citation	NID*
		ire Survey 2006												
Nigeria	2007	Nigeria Multiple Indicator Cluster Survey 2007	Survey microdata	Admin 1			X				X		United Nations Children's Fund (UNICEF), National Bureau of Statistics (Nigeria). Nigeria Multiple Indicator Cluster Survey 2007. New York, United States: United Nations Children's Fund (UNICEF).	9516
Nigeria	2007	Nigeria National HIV/AIDS and Reproductive Health Survey 2007	Survey microdata	Admin 1		X	X			X	X	X	Federal Ministry of Health (Nigeria), National Bureau of Statistics (Nigeria), National Population Commission (NPC), Society for Family Health (Nigeria), University College Hospital, Ibadan. Nigeria National HIV/AIDS and Reproductive Health Survey 2007.	325046†
Nigeria	2008	Nigeria Demographic and Health Survey 2008	Survey microdata	GPS	X	X	X	X	X	X	X	X	Macro International, Inc, National Population Commission of Nigeria. Nigeria Demographic and Health Survey 2008. Fairfax, United States: ICF International, 2009.	21433
Nigeria	2008	Nigeria General Household	Survey microdata	Admin 1								X	National Bureau of Statistics (Nigeria), Minnesota Population Center. Nigeria General Household Survey 2008 from	151313

Country	Years	Name	Type	Geographic Detail	Male Circumcision	STI Symptoms	Had Intercourse (Women)	Partner Away	Condom Last Time	Multiple Partners in Year (Men)	Multiple Partners in Year (Women)	In Union	Citation	NID*
		Survey 2008 - IPUMS											the Integrated Public Use Microdata Series, International: [Machine-readable database]. Minneapolis: University of Minnesota.	
Nigeria	2009	Nigeria General Household Survey 2009 - IPUMS	Survey microdata	Admin 1								X	National Bureau of Statistics (Nigeria), Minnesota Population Center. Nigeria General Household Survey 2009 from the Integrated Public Use Microdata Series, International: [Machine-readable database]. Minneapolis: University of Minnesota.	151314
Nigeria	2009	Nigeria Living Standards Survey 2008-2010	Survey microdata	Admin 1, Admin 2								X	National Bureau of Statistics (Nigeria). Nigeria Living Standards Survey 2008-2010. Abuja, Nigeria: National Bureau of Statistics (Nigeria).	151719
Nigeria	2010	Nigeria General Household Survey 2010 - IPUMS	Survey microdata	Admin 2								X	National Bureau of Statistics (Nigeria), Minnesota Population Center. Nigeria General Household Survey 2010 from the Integrated Public Use Microdata Series, International: [Machine-readable database]. Minneapolis: University of Minnesota.	151315
Nigeria	2011	Nigeria General	Survey microdata	Admin 1								X	National Bureau of Statistics (Nigeria), Minnesota Population Center. Nigeria	151317

Country	Years	Name	Type	Geographic Detail	Male Circumcision	STI Symptoms	Had Intercourse (Women)	Partner Away	Condom Last Time	Multiple Partners in Year (Men)	Multiple Partners in Year (Women)	In Union	Citation	NID*
		Household Survey 2010-2011 - IPUMS											General Household Survey 2010-2011 from the Integrated Public Use Microdata Series, International: [Machine-readable database]. Minneapolis: University of Minnesota.	
Nigeria	2011	Nigeria Multiple Indicator Cluster Survey 2011	Survey microdata	Admin 1			X				X		National Bureau of Statistics (Nigeria), United Nations Children's Fund (UNICEF). Nigeria Multiple Indicator Cluster Survey 2011. New York, United States: United Nations Children's Fund (UNICEF), 2013.	76703
Nigeria	2012	Nigeria National HIV/AIDS and Reproductive Health Survey 2012	Survey microdata	Admin 2		X	X			X	X	X	Expanded Social Marketing Project in Nigeria (ESMPIN), Federal Ministry of Health (Nigeria), Joint United Nations Program on HIV/AIDS (UNAIDS), National Population Commission (NPC), Society for Family Health (Nigeria), University College Hospital, Ibadan, World Health Organization (WHO). Nigeria National HIV/AIDS and Reproductive Health Survey 2012.	324443†
Nigeria	2013	Nigeria Demographic and Health	Survey microdata	GPS	X	X	X	X	X	X	X	X	ICF International, National Population Commission of Nigeria. Nigeria Demographic and Health Survey 2013.	77390

Country	Years	Name	Type	Geographic Detail	Male Circumcision	STI Symptoms	Had Intercourse (Women)	Partner Away	Condom Last Time	Multiple Partners in Year (Men)	Multiple Partners in Year (Women)	In Union	Citation	NID*
		Survey 2013											Fairfax, United States: ICF International.	
Nigeria	2014	Nigeria - Kaduna and Lagos Performance Monitoring and Accountability 2020 Survey, Round 1 2014	Survey microdata	Admin 1			X	X				X	Centre for Population and Reproductive Health (CPRH), University of Ibadan; Centre for Research, Evaluation Resources and Development (CRERD); Population and Reproductive Health Program (PRHP), Obafemi Awolowo University (OAU); Bayero University Kano (BUK); and The Bill & Melinda Gates Institute for Population and Reproductive Health at The Johns Hopkins Bloomberg School of Public Health. Performance Monitoring and Accountability 2020 (PMA2020) Survey round 1, PMA2014/Nigeria-R1 (Kaduna & Lagos). 2014. Nigeria and Baltimore, Maryland, USA.	256263
Nigeria	2015	Nigeria - Kaduna and Lagos Performance Monitoring	Survey microdata	Admin 1			X	X				X	Centre for Research, Evaluation Resources and Development (CRERD), Bayero University Kano (BUK), and The Bill & Melinda Gates Institute for Population and Reproductive Health at The Johns Hopkins Bloomberg	256268

Country	Years	Name	Type	Geographic Detail	Male Circumcision	STI Symptoms	Had Intercourse (Women)	Partner Away	Condom Last Time	Multiple Partners in Year (Men)	Multiple Partners in Year (Women)	In Union	Citation	NID*
		and Accountability 2020 Survey, Round 2 2015											School of Public Health. Performance Monitoring and Accountability 2020 (PMA2020) Survey round 2, PMA2015/Nigeria-R2 (Kaduna & Lagos). 2015. Nigeria and Baltimore, Maryland, USA	
Nigeria	2016	Nigeria Performance Monitoring and Accountability 2020 Survey, Round 3 2016	Survey microdata	Admin 1			X					X	Centre for Research, Evaluation Resources and Development (CRERD), Bayero University Kano (BUK), and The Bill & Melinda Gates Institute for Population and Reproductive Health at The Johns Hopkins Bloomberg School of Public Health. Performance Monitoring and Accountability 2020 (PMA2020) Survey round 3, PMA2016/Nigeria-R3 (National). 2016. Nigeria and Baltimore, Maryland, USA.	286022
Nigeria	2016-17	Nigeria Multiple Indicator Cluster Survey with National Immunizati	Survey microdata	GPS	X		X		X	X	X	X	National Agency for the Control of AIDS (Nigeria), National Bureau of Statistics (Nigeria), National Primary Health Care Development Agency (NPHCDA) (Nigeria), United Nations Children's Fund (UNICEF). Nigeria Multiple Indicator Cluster Survey with National Immunization Coverage	218613

Country	Years	Name	Type	Geographic Detail	Male Circumcision	STI Symptoms	Had Intercourse (Women)	Partner Away	Condom Last Time	Multiple Partners in Year (Men)	Multiple Partners in Year (Women)	In Union	Citation	NID*
		on Coverage Survey Supplement 2016-2017											Survey Supplement 2016-2017. New York, United States: United Nations Children's Fund (UNICEF), 2018.	
Rwanda	2000	Rwanda Demographic and Health Survey 2000	Survey microdata	Admin 1		X	X	X	X	X	X	X	Macro International, Inc, National Office of Population (Rwanda). Rwanda Demographic and Health Survey 2000. Calverton, United States: Macro International, Inc.	20722
Rwanda	2000	Rwanda Multiple Indicator Cluster Survey 2000	Survey microdata	Admin 1								X	Department of Statistics (Rwanda), United Nations Children's Fund (UNICEF). Rwanda Multiple Indicator Cluster Survey 2000. New York, United States: United Nations Children's Fund (UNICEF).	26930
Rwanda	2005	Rwanda Demographic and Health Survey 2005	Survey microdata	GPS	X	X	X	X	X	X	X	X	Macro International, Inc, National Institute of Statistics of Rwanda. Rwanda Demographic and Health Survey 2005. Calverton, United States: Macro International, Inc.	20740

Country	Years	Name	Type	Geographic Detail	Male Circumcision	STI Symptoms	Had Intercourse (Women)	Partner Away	Condom Last Time	Multiple Partners in Year (Men)	Multiple Partners in Year (Women)	In Union	Citation	NID*
Rwanda	2007-08	Rwanda Interim Demographic and Health Survey 2007-2008	Survey microdata	GPS	X			X				X	Macro International, Inc, Ministry of Health (Rwanda), National Institute of Statistics of Rwanda. Rwanda Interim Demographic and Health Survey 2007-2008. Calverton, United States: Macro International, Inc.	21222
Rwanda	2010-11	Rwanda Demographic and Health Survey 2010-2011	Survey microdata	GPS	X	X	X	X	X	X	X	X	ICF Macro, Ministry of Health (Rwanda), National Institute of Statistics of Rwanda. Rwanda Demographic and Health Survey 2010-2011. Calverton, United States: ICF Macro.	56040
Rwanda	2011	Rwanda Integrated Household Living Conditions Survey 2010-2011	Survey microdata	Admin 1								X	National Institute of Statistics of Rwanda. Rwanda Integrated Household Living Conditions Survey 2010-2011. Kigali, Rwanda: National Institute of Statistics of Rwanda.	151437
Rwanda	2011	Rwanda Special Demographic and Health	Survey microdata	Admin 1								X	ICF International, Joint United Nations Program on HIV/AIDS (UNAIDS), National Institute of Statistics of Rwanda, Rwanda Biomedical Center/Institute of HIV/AIDS, Disease	56426

Country	Years	Name	Type	Geographic Detail	Male Circumcision	STI Symptoms	Had Intercourse (Women)	Partner Away	Condom Last Time	Multiple Partners in Year (Men)	Multiple Partners in Year (Women)	In Union	Citation	NID*
		Survey 2011											Control and Prevention Department, School of Public Health, University of Rwanda. Rwanda Special Demographic and Health Survey 2011. Fairfax, United States: ICF International, 2012.	
Rwanda	2014-15	Rwanda Demographic and Health Survey 2014-2015	Survey microdata	GPS	X	X	X	X	X	X	X	X	ICF International, Ministry of Health (Rwanda), National Institute of Statistics of Rwanda. Rwanda Demographic and Health Survey 2014-2015. Fairfax, United States: ICF International, 2016.	157063
Rwanda	2017	Rwanda Malaria Indicator Survey 2017	Survey microdata	Admin 2								X	ICF International, Ministry of Health (Rwanda), Rwanda Biomedical Center. Rwanda Malaria Indicator Survey 2017. Fairfax, United States: ICF International, 2018.	350836
Sao Tome and Principe	2000	Sao Tome and Principe Multiple Indicator Cluster Survey 2000	Survey microdata	Admin 1								X	National Institute of Statistics (Sao Tome and Principe), United Nations Children's Fund (UNICEF). Sao Tome and Principe Multiple Indicator Cluster Survey 2000. New York, United States: United Nations Children's Fund (UNICEF).	27055

Country	Years	Name	Type	Geographic Detail	Male Circumcision	STI Symptoms	Had Intercourse (Women)	Partner Away	Condom Last Time	Multiple Partners in Year (Men)	Multiple Partners in Year (Women)	In Union	Citation	NID*
Sao Tome and Principe	2008	Sao Tome and Principe Demographic and Health Survey 2008-2009	Survey microdata	Admin 2		X	X	X	X	X	X	X	ICF Macro, Ministry of Health (Sao Tome and Principe), National Institute of Statistics (Sao Tome and Principe). Sao Tome and Principe Demographic and Health Survey 2008-2009. Calverton, United States: ICF Macro.	26866
Sao Tome and Principe	2014	Sao Tome and Principe Multiple Indicator Cluster Survey 2014	Survey microdata	Admin 2	X		X		X	X	X	X	Global Fund to Fight Aids Tuberculosis and Malaria (GFATM), ICF International, National Center for Endemic Diseases (CNE) (Sao Tome and Principe), National Institute of Statistics (Sao Tome and Principe), United Nations Children's Fund (UNICEF), United Nations Development Programme (UNDP). Sao Tome and Principe Multiple Indicator Cluster Survey 2014. New York, United States: United Nations Children's Fund (UNICEF), 2016.	214640
Senegal	2000	Senegal Multiple Indicator Cluster	Survey microdata	Admin 1								X	Directorate of Forecasting and Statistics, Ministry of the Economy, Finance and Planning (Senegal), Ministry of Economy and Finance	27044

Country	Years	Name	Type	Geographic Detail	Male Circumcision	STI Symptoms	Had Intercourse (Women)	Partner Away	Condom Last Time	Multiple Partners in Year (Men)	Multiple Partners in Year (Women)	In Union	Citation	NID*
		Survey 2000											(Senegal), United Nations Children's Fund (UNICEF). Senegal Multiple Indicator Cluster Survey 2000. New York, United States: United Nations Children's Fund (UNICEF).	
Senegal	2005	Senegal Demographic and Health Survey 2005	Survey microdata	GPS	X	X	X	X	X	X	X	X	Ministry of Health and Prevention (Senegal), Research Center for Human Development (Senegal). Senegal Demographic and Health Survey 2005. Calverton, United States: Macro International, Inc.	26855
Senegal	2010-11	Senegal Demographic and Health Survey 2010-2011	Survey microdata	GPS		X	X	X	X	X	X	X	Center for Research in Human Development (CRDH), Cheikh Anta Diop University, Hospital Aristide Le Dantec, ICF Macro, National Agency of Statistics and Demography (Senegal). Senegal Demographic and Health Survey 2010-2011. Fairfax, United States: ICF International.	56063
Senegal	2012-13	Senegal Continuous Demographic and Health	Survey microdata	GPS			X	X			X	X	ICF International, Ministry of Health and Social Action (Senegal), National Agency of Statistics and Demography (Senegal). Senegal Continuous Demographic and Health Survey 2012-	111432

Country	Years	Name	Type	Geographic Detail	Male Circumcision	STI Symptoms	Had Intercourse (Women)	Partner Away	Condom Last Time	Multiple Partners in Year (Men)	Multiple Partners in Year (Women)	In Union	Citation	NID*
		Survey 2012-2013											2013. Fairfax, United States: ICF International, 2014.	
Senegal	2014	Senegal Continuous Demographic and Health Survey 2014	Survey microdata	GPS		X	X	X	X	X	X	X	Cheikh Anta Diop University, ICF International, National Agency of Statistics and Demography (Senegal). Senegal Continuous Demographic and Health Survey 2014. Fairfax, United States: ICF International, 2015.	191270
Senegal	2015	Senegal - Dakar Urban Multiple Indicator Cluster Survey 2015-2016	Survey microdata	Admin 2			X		X	X	X	X	National Agency of Statistics and Demography (Senegal), United Nations Children's Fund (UNICEF). Senegal - Dakar Urban Multiple Indicator Cluster Survey 2015-2016. New York, United States: United Nations Children's Fund (UNICEF), 2018.	287639
Senegal	2015	Senegal Continuous Demographic and Health Survey 2015	Survey microdata	GPS		X	X	X	X	X	X	X	Cheikh Anta Diop University, ICF International, National Agency of Statistics and Demography (Senegal). Senegal Continuous Demographic and Health Survey 2015. Fairfax, United States: ICF International, 2016.	218592

Country	Years	Name	Type	Geographic Detail	Male Circumcision	STI Symptoms	Had Intercourse (Women)	Partner Away	Condom Last Time	Multiple Partners in Year (Men)	Multiple Partners in Year (Women)	In Union	Citation	NID*
Senegal	2015	Senegal Global Adult Tobacco Survey 2015	Survey microdata	Admin 1								X	Centers for Disease Control and Prevention (CDC), Johns Hopkins Bloomberg School of Public Health, Ministry of Health and Social Action (Senegal), National Agency of Statistics and Demography (Senegal), Research Triangle Institute, Inc. (RTI), World Health Organization (WHO). Senegal Global Adult Tobacco Survey 2015.	237375
Senegal	2016	Senegal Continuous Demographic and Health Survey 2016	Survey microdata	GPS		X	X	X	X	X	X	X	ICF International, Ministry of Health and Social Action (Senegal), National Agency of Statistics and Demography (Senegal). Senegal Continuous Demographic and Health Survey 2016. Fairfax, United States: ICF International, 2017.	286772
Senegal	2017	Senegal Continuous Demographic and Health Survey 2017	Survey microdata	Admin 1		X	X	X	X	X	X	X	ICF International, Ministry of Health and Social Action (Senegal), National Agency of Statistics and Demography (Senegal), Unit for the Fight Against Malnutrition (Senegal). Senegal Continuous Demographic and Health Survey 2017. Fairfax, United States: ICF International, 2018.	353526

Country	Years	Name	Type	Geographic Detail	Male Circumcision	STI Symptoms	Had Intercourse (Women)	Partner Away	Condom Last Time	Multiple Partners in Year (Men)	Multiple Partners in Year (Women)	In Union	Citation	NID*
Sierra Leone	2000	Sierra Leone Multiple Indicator Cluster Survey 2000	Survey microdata	Admin 1								X	Central Statistics Office (Sierra Leone), United Nations Children's Fund (UNICEF). Sierra Leone Multiple Indicator Cluster Survey 2000. New York, United States: United Nations Children's Fund (UNICEF).	11639
Sierra Leone	2005	Sierra Leone Multiple Indicator Cluster Survey 2005	Survey microdata	Admin 2			X				X		United Nations Children's Fund (UNICEF), Statistics Sierra Leone. Sierra Leone Multiple Indicator Cluster Survey 2005. New York, United States: United Nations Children's Fund (UNICEF).	11649
Sierra Leone	2008	Sierra Leone Demographic and Health Survey 2008	Survey microdata	GPS	X	X	X	X	X	X	X	X	Macro International, Inc, Statistics Sierra Leone. Sierra Leone Demographic and Health Survey 2008. Fairfax, United States: ICF International.	21258
Sierra Leone	2010	Sierra Leone Multiple Indicator	Survey microdata	Admin 2			X				X		Statistics Sierra Leone, United Nations Children's Fund (UNICEF). Sierra Leone Multiple Indicator Cluster Survey 2010. New York, United States:	76700

Country	Years	Name	Type	Geographic Detail	Male Circumcision	STI Symptoms	Had Intercourse (Women)	Partner Away	Condom Last Time	Multiple Partners in Year (Men)	Multiple Partners in Year (Women)	In Union	Citation	NID*
		Cluster Survey 2010											United Nations Children's Fund (UNICEF).	
Sierra Leone	2013	Sierra Leone Demographic and Health Survey 2013	Survey microdata	GPS	X	X	X	X	X	X	X	X	ICF International, Ministry of Health and Sanitation (Sierra Leone), Statistics Sierra Leone. Sierra Leone Demographic and Health Survey 2013. Fairfax, United States: ICF International, 2014.	131467
Sierra Leone	2014	Sierra Leone Labor Force Survey 2014	Survey microdata	Admin 2								X	German Society for International Cooperation (GIZ), International Labour Organization (ILO), Statistics Sierra Leone, World Bank. Sierra Leone Labor Force Survey 2014.	286094
South Africa	2002	South Africa - Agincourt Integrated Family Survey 2002	Survey microdata	Admin 4								X	Case, A. 2003. Agincourt Integrated Family Survey 2002. [dataset] Version 1. Cape Town: DataFirst [distributor].	135825
South Africa	2002	South Africa	Survey microdata	Admin 1								X	Statistics South Africa. South Africa General Household Survey 2002.	115481

Country	Years	Name	Type	Geographic Detail	Male Circumcision	STI Symptoms	Had Intercourse (Women)	Partner Away	Condom Last Time	Multiple Partners in Year (Men)	Multiple Partners in Year (Women)	In Union	Citation	NID*
		General Household Survey 2002											Pretoria, South Africa: Statistics South Africa.	
South Africa	2002	South Africa HIV/AIDS Behavioral Risks, Sero-Status, and Mass Media Impact Survey 2002	Survey microdata	Admin 3	X		X		X	X	X	X	Center for AIDS Development, Research and Evaluation (CADRE) (South Africa), Department of Social Development (South Africa), Family Health International, Geospace International (South Africa), Human Sciences Research Council, Joint United Nations Program on HIV/AIDS (UNAIDS), Medical University of Southern Africa (MEDUNSA), National Agency for AIDS Research (ANRS) (France), National Health Laboratory Service (NHLS) (South Africa), South African Medical Research Council, University of Natal, Wits Health Consortium. South Africa HIV/AIDS Behavioral Risks, Sero-Status, and Mass Media Impact Survey 2002. Pretoria, South Africa: Human Sciences Research Council, 2011.	12102

Country	Years	Name	Type	Geographic Detail	Male Circumcision	STI Symptoms	Had Intercourse (Women)	Partner Away	Condom Last Time	Multiple Partners in Year (Men)	Multiple Partners in Year (Women)	In Union	Citation	NID*
South Africa	2003	South Africa General Household Survey 2003	Survey microdata	Admin 1								X	Statistics South Africa. South Africa General Household Survey 2003. Pretoria, South Africa: Statistics South Africa.	11787
South Africa	2004	South Africa General Household Survey 2004	Survey microdata	Admin 1								X	Statistics South Africa. South Africa General Household Survey 2004. Pretoria, South Africa: Statistics South Africa.	11788
South Africa	2005	South Africa General Household Survey 2005	Survey microdata	Admin 1								X	Statistics South Africa. South Africa General Household Survey 2005. Pretoria, South Africa: Statistics South Africa.	11789
South Africa	2005	South Africa National HIV Prevalence, Incidence, Behavior	Survey microdata	Admin 3			X		X	X	X	X	Center for AIDS Development, Research and Evaluation (CADRE) (South Africa), Centers for Disease Control and Prevention (CDC), Global Clinical and Viral Laboratory (South Africa), Human Sciences Research Council, Maphume Research Services,	313074

Country	Years	Name	Type	Geographic Detail	Male Circumcision	STI Symptoms	Had Intercourse (Women)	Partner Away	Condom Last Time	Multiple Partners in Year (Men)	Multiple Partners in Year (Women)	In Union	Citation	NID*
		and Communication Survey 2004-2005											National Institute for Communicable Diseases (South Africa). South Africa National HIV Prevalence, Incidence, Behavior and Communication Survey 2004-2005. Pretoria, South Africa: Human Sciences Research Council, 2011.	
South Africa	2006	South Africa General Household Survey 2006	Survey microdata	Admin 1								X	Statistics South Africa. South Africa General Household Survey 2006. Pretoria, South Africa: Statistics South Africa.	115486
South Africa	2007	South Africa General Household Survey 2007	Survey microdata	Admin 1								X	Statistics South Africa. South Africa General Household Survey 2007. Cape Town, South Africa: DataFirst.	11790
South Africa	2008	South Africa General Household Survey 2008	Survey microdata	Admin 1								X	Statistics South Africa. South Africa General Household Survey 2008. Pretoria, South Africa: Statistics South Africa.	115488

Country	Years	Name	Type	Geographic Detail	Male Circumcision	STI Symptoms	Had Intercourse (Women)	Partner Away	Condom Last Time	Multiple Partners in Year (Men)	Multiple Partners in Year (Women)	In Union	Citation	NID*
South Africa	2008-09	South Africa National HIV Prevalence, Incidence, Behavior and Communication Survey 2008-2009	Survey microdata	Admin 5, GPS	X	X	X		X	X	X	X	Center for AIDS Development, Research and Evaluation (CADRE) (South Africa), Geospace International (South Africa), Global Clinical and Viral Laboratory (South Africa), Human Sciences Research Council, Maphume Research Services, National Institute for Communicable Diseases (South Africa), South African Medical Research Council. South Africa National HIV Prevalence, Incidence, Behavior and Communication Survey 2008-2009. Pretoria, South Africa: Human Sciences Research Council, 2014.	228102
South Africa	2009	South Africa General Household Survey 2009	Survey microdata	Admin 1								X	Statistics South Africa. South Africa General Household Survey 2009. Pretoria, South Africa: Statistics South Africa.	115489
South Africa	2009	South Africa Living Conditions	Survey microdata	Admin 1								X	Statistics South Africa. South Africa Living Conditions Survey 2008-2009. Pretoria, South Africa: Statistics South Africa.	152021

Country	Years	Name	Type	Geographic Detail	Male Circumcision	STI Symptoms	Had Intercourse (Women)	Partner Away	Condom Last Time	Multiple Partners in Year (Men)	Multiple Partners in Year (Women)	In Union	Citation	NID*
		Survey 2008-2009												
South Africa	2010-11	South Africa National Income Dynamics Study - Wave 2 2010-2011	Survey microdata	Admin 1, Admin 2, Admin 3								X	Southern Africa Labour and Development Research Unit. National Income Dynamics Study 2010-2011, Wave 2. Version 1.0. Cape Town: Southern Africa Labour and Development Research Unit [producer], 2012. Cape Town: DataFirst [distributor], 2013.	133731
South Africa	2011-12	South Africa National HIV Prevalence, Incidence, and Behavior Survey 2011-2012	Survey microdata	Admin 5, GPS	X		X		X	X	X	X	Centers for Disease Control and Prevention (CDC), Global Clinical and Viral Laboratory (South Africa), Human Sciences Research Council, National Institute for Communicable Diseases (South Africa), South African Medical Research Council, University of Cape Town. South Africa National HIV Prevalence, Incidence, and Behavior Survey 2011-2012. Pretoria, South Africa: Human Sciences Research Council, 2016.	313076
South Africa	2013	South Africa General	Survey microdata	Admin 1								X	Statistics South Africa. South Africa General Household Survey 2013. Cape Town, South Africa: DataFirst.	238483

Country	Years	Name	Type	Geographic Detail	Male Circumcision	STI Symptoms	Had Intercourse (Women)	Partner Away	Condom Last Time	Multiple Partners in Year (Men)	Multiple Partners in Year (Women)	In Union	Citation	NID*
		Household Survey 2013												
South Africa	2014	South Africa General Household Survey 2014	Survey microdata	Admin 1								X	Statistics South Africa. South Africa General Household Survey 2014. Cape Town, South Africa: DataFirst, 2015.	238485
South Africa	2015	South Africa Living Conditions Survey 2014-2015	Survey microdata	Admin 1								X	Statistics South Africa. South Africa Living Conditions Survey 2014-2015. Pretoria, South Africa: Statistics South Africa.	317239
South Africa	2016	South Africa Community Survey 2016	Survey microdata	Admin 3								X	Statistics South Africa. South Africa Community Survey 2016. Pretoria, South Africa: Statistics South Africa, 2016.	280803
South Africa	2016	South Africa Demograp hic and Health	Survey microdata	GPS	X	X	X	X	X	X	X	X	Department of Health (South Africa), ICF International, South African Medical Research Council, Statistics South Africa. South Africa Demographic and Health Survey 2016.	157064

Country	Years	Name	Type	Geographic Detail	Male Circumcision	STI Symptoms	Had Intercourse (Women)	Partner Away	Condom Last Time	Multiple Partners in Year (Men)	Multiple Partners in Year (Women)	In Union	Citation	NID*
		Survey 2016											Fairfax, United States: ICF International, 2019.	
South Africa	2016	South Africa General Household Survey 2016	Survey microdata	Admin 1								X	Statistics South Africa. South Africa General Household Survey 2016. Cape Town, South Africa: DataFirst, 2015.	317089
South Sudan	2010	Sudan - South Multiple Indicator Cluster Survey 2010	Survey microdata	Admin 1			X				X	X	Central Bureau of Statistics (Sudan), Federal Ministry of Health (Sudan), Government of Sudan, Ministry of Health (South Sudan), Southern Sudan Centre for Census, Statistics and Evaluation. Sudan - South Multiple Indicator Cluster Survey 2010. New York, United States: United Nations Children's Fund (UNICEF), 2015.	32189
Sudan	2000	Sudan Multiple Indicator Cluster Survey 2000	Survey microdata	Admin 1								X	Central Bureau of Statistics (Sudan), Federal Ministry of Health (Sudan), United Nations Children's Fund (UNICEF). Sudan Multiple Indicator Cluster Survey 2000. New York, United States: United Nations Children's Fund (UNICEF).	12243

Country	Years	Name	Type	Geographic Detail	Male Circumcision	STI Symptoms	Had Intercourse (Women)	Partner Away	Condom Last Time	Multiple Partners in Year (Men)	Multiple Partners in Year (Women)	In Union	Citation	NID*
Sudan	2010	Sudan - North Multiple Indicator Cluster Survey 2010	Survey microdata	Admin 1								X	Central Bureau of Statistics (Sudan), Ministry of Health (South Sudan). Sudan - North Multiple Indicator Cluster Survey 2010. New York, United States: United Nations Children's Fund (UNICEF), 2015.	153643
Swaziland	2000	Swaziland Multiple Indicator Cluster Survey 2000	Survey microdata	Admin 1								X	Central Statistical Office (Swaziland), United Nations Children's Fund (UNICEF). Swaziland Multiple Indicator Cluster Survey 2000. New York, United States: United Nations Children's Fund (UNICEF).	12320
Swaziland	2006-07	Swaziland Demographic and Health Survey 2006-2007	Survey microdata	GPS	X	X	X	X	X	X	X	X	Central Statistical Office (Swaziland), Macro International, Inc. Swaziland Demographic and Health Survey 2006-2007. Fairfax, United States: ICF International.	20829
Swaziland	2010	Swaziland Multiple Indicator Cluster Survey 2010	Survey microdata	Admin 1	X	X	X		X	X	X	X	Central Statistical Office (Swaziland), United Nations Children's Fund (UNICEF). Swaziland Multiple Indicator Cluster Survey 2010. New York, United States: United Nations Children's Fund (UNICEF).	30325

Country	Years	Name	Type	Geographic Detail	Male Circumcision	STI Symptoms	Had Intercourse (Women)	Partner Away	Condom Last Time	Multiple Partners in Year (Men)	Multiple Partners in Year (Women)	In Union	Citation	NID*
Swaziland	2014	Swaziland Multiple Indicator Cluster Survey 2014	Survey microdata	Admin 1	X		X		X	X	X	X	Central Statistical Office (Swaziland), United Nations Children's Fund (UNICEF), United Nations Educational, Scientific and Cultural Organization (UNESCO), United Nations Population Fund (UNFPA). Swaziland Multiple Indicator Cluster Survey 2014. New York, United States: United Nations Children's Fund (UNICEF), 2016.	200707
Tanzania	2001	Tanzania Household Budget Survey 2000-2001	Survey microdata	Admin 1								X	National Bureau of Statistics (Tanzania), Oxford Policy Management. Tanzania Household Budget Survey 2000-2001. Dar es Salaam, Tanzania: National Bureau of Statistics (Tanzania).	31740
Tanzania	2003-04	Tanzania AIDS Indicator Survey 2003-2004	Survey microdata	GPS	X	X	X		X	X	X	X	National Bureau of Statistics (Tanzania), ORC Macro, Tanzania Commission for AIDS (TACAIDS). Tanzania AIDS Indicator Survey 2003-2004. Calverton, United States: ORC Macro.	12630
Tanzania	2004	Tanzania - Shinyanga Core Welfare	Survey microdata	Admin 2								X	Economic Development Initiatives (EDI), National Bureau of Statistics (Tanzania), Institute of Finance Management (Tanzania), World Bank	31786

Country	Years	Name	Type	Geographic Detail	Male Circumcision	STI Symptoms	Had Intercourse (Women)	Partner Away	Condom Last Time	Multiple Partners in Year (Men)	Multiple Partners in Year (Women)	In Union	Citation	NID*
		Indicators Questionnaire Survey 2004											(WB). Tanzania - Shinyanga Core Welfare Indicators Questionnaire Survey 2004. Bukoba, Tanzania: Economic Development Initiatives (EDI).	
Tanzania	2004-05	Tanzania Demographic and Health Survey 2004-2005	Survey microdata	Admin 1	X	X	X	X	X	X	X	X	Macro International, Inc, National Bureau of Statistics (Tanzania). Tanzania Demographic and Health Survey 2004-2005. Fairfax, United States: ICF International.	20875
Tanzania	2005	Tanzania Core Welfare Indicators Questionnaire Survey 2005	Survey microdata	Admin 2								X	Economic Development Initiatives (EDI), World Bank (WB). Tanzania Core Welfare Indicators Questionnaire Survey 2005. Bukoba, Tanzania: Economic Development Initiatives (EDI).	31797
Tanzania	2006-07	Tanzania Core Welfare Indicators Questionnaire Survey 2006-2007	Survey microdata	Admin 2								X	Economic Development Initiatives (EDI), World Bank (WB). Tanzania Core Welfare Indicators Questionnaire Survey 2006-2007. Bukoba, Tanzania: Economic Development Initiatives (EDI).	31831

Country	Years	Name	Type	Geographic Detail	Male Circumcision	STI Symptoms	Had Intercourse (Women)	Partner Away	Condom Last Time	Multiple Partners in Year (Men)	Multiple Partners in Year (Women)	In Union	Citation	NID*
Tanzania	2007	Tanzania Household Budget Survey 2007	Survey microdata	Admin 1								X	National Bureau of Statistics (Tanzania). Tanzania Household Budget Survey 2007. Dar es Salaam, Tanzania: National Bureau of Statistics (Tanzania).	31887
Tanzania	2007-08	Tanzania HIV/AIDS and Malaria Indicator Survey 2007-2008	Survey microdata	GPS	X	X	X	X	X	X	X	X	Macro International, Inc, National Bureau of Statistics (Tanzania), Office of Chief Government Statistician (OCGS-Zanzibar), Tanzania Commission for AIDS (TACAIDS), Zanzibar AIDS Commission (ZAC). Tanzania HIV/AIDS and Malaria Indicator Survey 2007-2008. Calverton, United States: Macro International, Inc.	12644
Tanzania	2009-10	Tanzania Demographic and Health Survey 2009-2010	Survey microdata	GPS	X	X	X	X	X	X	X	X	ICF Macro, National Bureau of Statistics (Tanzania). Tanzania Demographic and Health Survey 2009-2010. Calverton, United States: ICF Macro.	21331
Tanzania	2011-12	Tanzania AIDS Indicator	Survey microdata	GPS	X	X	X	X	X	X	X	X	ICF International, National Bureau of Statistics (Tanzania), Office of Chief Government Statistician (OCGS-Zanzibar), Tanzania Commission for	77395

Country	Years	Name	Type	Geographic Detail	Male Circumcision	STI Symptoms	Had Intercourse (Women)	Partner Away	Condom Last Time	Multiple Partners in Year (Men)	Multiple Partners in Year (Women)	In Union	Citation	NID*
		Survey 2011-2012											AIDS (TACAIDS), Zanzibar AIDS Commission (ZAC). Tanzania AIDS Indicator Survey 2011-2012. Fairfax, United States: ICF International, 2013.	
Tanzania	2012	Tanzania Household Budget Survey 2011-2012	Survey microdata	Admin 1								X	National Bureau of Statistics (Tanzania). Tanzania Household Budget Survey 2011-2012. Dar es Salaam, Tanzania: National Bureau of Statistics (Tanzania), 2014.	243012
Tanzania	2015-16	Tanzania Demographic and Health Survey 2015-2016	Survey microdata	GPS	X		X	X	X			X	ICF International, Ministry of Health (Zanzibar), Ministry of Health, Community Development, Gender, Elderly and Children (MoHCDEC) (Tanzania), National Bureau of Statistics (Tanzania), Office of Chief Government Statistician (OCGS-Zanzibar). Tanzania Demographic and Health Survey 2015-2016. Fairfax, United States: ICF International, 2016.	218593
The Gambia	2010	Gambia Multiple Indicator Cluster Survey 2010	Survey microdata	Admin 1			X				X		Gambia Bureau of Statistics (GBOS), United Nations Children's Fund (UNICEF). Gambia Multiple Indicator Cluster Survey 2010. New York, United States: United Nations Children's Fund (UNICEF), 2018.	91506

Country	Years	Name	Type	Geographic Detail	Male Circumcision	STI Symptoms	Had Intercourse (Women)	Partner Away	Condom Last Time	Multiple Partners in Year (Men)	Multiple Partners in Year (Women)	In Union	Citation	NID*
The Gambia	2013	Gambia Demographic and Health Survey 2013	Survey microdata	Admin 2		X	X	X	X	X	X	X	Gambia Bureau of Statistics (GBOS), ICF International, Ministry of Health and Social Welfare (Gambia). Gambia Demographic and Health Survey 2013. Fairfax, United States: ICF International, 2015.	77384
Togo	2006	Togo Multiple Indicator Cluster Survey 2006	Survey microdata	Admin 1			X				X		Directorate General of Statistics and National Accounting (Togo), United Nations Children's Fund (UNICEF). Togo Multiple Indicator Cluster Survey 2006. New York, United States: United Nations Children's Fund (UNICEF).	12896
Togo	2010	Togo Multiple Indicator Cluster Survey 2010	Survey microdata	Admin 1			X		X	X	X	X	Directorate General of Statistics and National Accounting (Togo), United Nations Children's Fund (UNICEF). Togo Multiple Indicator Cluster Survey 2010. New York, United States: United Nations Children's Fund (UNICEF).	40021
Togo	2013-14	Togo Demographic and Health Survey 2013-2014	Survey microdata	GPS	X	X	X	X	X	X	X	X	Directorate General of Statistics and National Accounts (Togo), ICF International, Ministry of Health (Togo), Ministry of Planning, Development and Zoning (Togo). Togo Demographic and Health Survey 2013-	77515

Country	Years	Name	Type	Geographic Detail	Male Circumcision	STI Symptoms	Had Intercourse (Women)	Partner Away	Condom Last Time	Multiple Partners in Year (Men)	Multiple Partners in Year (Women)	In Union	Citation	NID*
													2014. Fairfax, United States: ICF International, 2015.	
Togo	2017	Togo Malaria Indicator Survey 2017	Survey microdata	GPS								X	ICF International, National Institute of Hygiene, Ministry of Health (Togo), National Institute of Statistics and Economic and Demographic Studies (INSEED) (Togo). Togo Malaria Indicator Survey 2017. Fairfax, United States: ICF International, 2018.	359318
Uganda	2000-01	Uganda Demographic and Health Survey 2000-2001	Survey microdata	GPS		X	X	X	X	X	X	X	Macro International, Inc, Uganda Bureau of Statistics. Uganda Demographic and Health Survey 2000-2001. Calverton, United States: Macro International, Inc.	20993
Uganda	2004	Uganda National Service Delivery Survey 2004	Survey microdata	Admin 1								X	Ministry of Public Service (Uganda), Uganda Bureau of Statistics. Uganda National Service Delivery Survey 2004. OpenMicroData.	13114
Uganda	2004-05	Uganda AIDS Indicator	Survey microdata	Admin 1	X	X	X			X	X	X	Division of Reproductive Health, Centers for Disease Control and Prevention (CDC), Ministry of Health (Uganda). Uganda AIDS Indicator	13084†

Country	Years	Name	Type	Geographic Detail	Male Circumcision	STI Symptoms	Had Intercourse (Women)	Partner Away	Condom Last Time	Multiple Partners in Year (Men)	Multiple Partners in Year (Women)	In Union	Citation	NID*
		Survey 2004-2005											Survey 2004-2005. Calverton, United States: Macro International, Inc.	
Uganda	2006	Uganda Demographic and Health Survey 2006	Survey microdata	GPS	X	X	X	X	X	X	X	X	Macro International, Inc, Uganda Bureau of Statistics. Uganda Demographic and Health Survey 2006. Calverton, United States: Macro International, Inc.	21014
Uganda	2008	Uganda National Service Delivery Survey 2008	Survey microdata	Admin 1								X	Ministry of Public Service (Uganda), Uganda Bureau of Statistics. Uganda National Service Delivery Survey 2008.	93320
Uganda	2011	Uganda AIDS Indicator Survey 2011	Survey microdata	GPS	X	X	X	X	X	X	X	X	Centers for Disease Control and Prevention (CDC), ICF Macro, Ministry of Health (Uganda), Uganda Bureau of Statistics, Uganda Virus Research Institute. Uganda AIDS Indicator Survey 2011. Calverton, United States: ICF Macro.	55973
Uganda	2011	Uganda Demographic and Health	Survey microdata	GPS	X	X	X	X	X	X	X	X	ICF Macro, Uganda Bureau of Statistics. Uganda Demographic and Health Survey 2011. Fairfax, United States: ICF International.	56021

Country	Years	Name	Type	Geographic Detail	Male Circumcision	STI Symptoms	Had Intercourse (Women)	Partner Away	Condom Last Time	Multiple Partners in Year (Men)	Multiple Partners in Year (Women)	In Union	Citation	NID*
		Survey 2011												
Uganda	2013	Uganda National Household Survey 2012-2013	Survey microdata	Admin 1								X	Uganda Bureau of Statistics. Uganda National Household Survey 2012-2013. Kampala, Uganda: Uganda Bureau of Statistics.	165406
Uganda	2014	Uganda Performance Monitoring and Accountability 2020 Survey, Round 1 2014	Survey microdata	Admin 1			X	X				X	Makerere University, School of Public Health at the College of Health Sciences and The Bill & Melinda Gates Institute for Population and Reproductive Health at The Johns Hopkins Bloomberg School of Public Health. Performance Monitoring and Accountability 2020 (PMA2020) Survey round 1, PMA2014/Uganda-R1. 2014. Uganda and Baltimore, Maryland, USA.	256199
Uganda	2015	Uganda Performance Monitoring and Accountability 2020	Survey microdata	Admin 1			X	X				X	Makerere University, School of Public Health at the College of Health Sciences and The Bill & Melinda Gates Institute for Population and Reproductive Health at The Johns Hopkins Bloomberg School of Public Health. Performance Monitoring and	256200

Country	Years	Name	Type	Geographic Detail	Male Circumcision	STI Symptoms	Had Intercourse (Women)	Partner Away	Condom Last Time	Multiple Partners in Year (Men)	Multiple Partners in Year (Women)	In Union	Citation	NID*
		Survey, Round 2 2015											Accountability 2020 (PMA2020) Survey round 2, PMA2015/Uganda-R2. 2015. Uganda and Baltimore, Maryland, USA.	
Uganda	2015	Uganda Performance Monitoring and Accountability 2020 Survey, Round 3 2015	Survey microdata	Admin 1			X	X				X	Makerere University, School of Public Health at the College of Health Sciences and The Bill & Melinda Gates Institute for Population and Reproductive Health at The Johns Hopkins Bloomberg School of Public Health. Performance Monitoring and Accountability 2020 (PMA2020) Survey round 3, PMA2015/Uganda-R3. 2015. Uganda and Baltimore, Maryland, USA.	256201
Uganda	2016	Uganda Demographic and Health Survey 2016	Survey microdata	GPS	X	X	X	X	X	X	X	X	ICF International, Uganda Bureau of Statistics. Uganda Demographic and Health Survey 2016. Fairfax, United States: ICF International, 2018.	286780
Uganda	2016	Uganda Performance Monitoring	Survey microdata	Admin 1			X	X				X	Makerere University, School of Public Health at the College of Health Sciences and The Bill & Melinda Gates Institute for Population and	285893

Country	Years	Name	Type	Geographic Detail	Male Circumcision	STI Symptoms	Had Intercourse (Women)	Partner Away	Condom Last Time	Multiple Partners in Year (Men)	Multiple Partners in Year (Women)	In Union	Citation	NID*
		and Accountability 2020 Survey, Round 4 2016											Reproductive Health at The Johns Hopkins Bloomberg School of Public Health. Performance Monitoring and Accountability 2020 (PMA2020) Survey round 4, PMA2016/Uganda-R4. 2016. Uganda and Baltimore, Maryland, USA.	
Uganda	2017	Uganda Performance Monitoring and Accountability 2020 Survey, Round 5 2017	Survey microdata	Admin >1			X	X				X	Makerere University, School of Public Health at the College of Health Sciences and The Bill & Melinda Gates Institute for Population and Reproductive Health at The Johns Hopkins Bloomberg School of Public Health. Performance Monitoring and Accountability 2020 (PMA2020) Survey round 5, PMA2017/Uganda-R5. 2017. Uganda and Baltimore, Maryland, USA.	347043
Zambia	2000	Zambia Sexual Behavior Survey 2000	Survey microdata	Admin 2	X	X	X	X		X	X	X	Central Board of Health (Zambia), Central Statistical Office (Zambia), MEASURE Evaluation Project, Carolina Population Center, University of North Carolina, University of Zambia, Zambian National AIDS Council. Zambia Sexual Behavior Survey 2000.	27924

Country	Years	Name	Type	Geographic Detail	Male Circumcision	STI Symptoms	Had Intercourse (Women)	Partner Away	Condom Last Time	Multiple Partners in Year (Men)	Multiple Partners in Year (Women)	In Union	Citation	NID*
													Lusaka, Zambia: Central Statistical Office (Zambia).	
Zambia	2001-02	Zambia Demographic and Health Survey 2001-2002	Survey microdata	Admin 2		X	X	X	X	X	X	X	Central Board of Health (Zambia), Central Statistical Office (Zambia), Macro International, Inc. Zambia Demographic and Health Survey 2001-2002. Fairfax, United States: ICF International.	21102
Zambia	2003	Zambia Living Conditions Monitoring Survey 2002-2003	Survey microdata	Admin 2								X	Central Statistical Office (Zambia). Zambia Living Conditions Monitoring Survey 2002-2003. Lusaka, Zambia: Central Statistical Office (Zambia).	14027†
Zambia	2003	Zambia Sexual Behavior Survey 2003	Survey microdata	Admin 2	X	X	X	X				X	Central Board of Health (Zambia), Central Statistical Office (Zambia), MEASURE Evaluation Project, Carolina Population Center, University of North Carolina, Zambian National AIDS Council. Zambia Sexual Behavior Survey 2003. Lusaka, Zambia: Central Statistical Office (Zambia).	27952
Zambia	2005	Zambia Sexual Behavior	Survey microdata	Admin 2	X	X	X	X		X	X	X	Central Statistical Office (Zambia), Department for International Development (DFiD) (United	27987

Country	Years	Name	Type	Geographic Detail	Male Circumcision	STI Symptoms	Had Intercourse (Women)	Partner Away	Condom Last Time	Multiple Partners in Year (Men)	Multiple Partners in Year (Women)	In Union	Citation	NID*
		Survey 2005											Kingdom), Family Health International, MEASURE Evaluation Project, Carolina Population Center, University of North Carolina, Ministry of Health (Zambia), University of Zambia, Zambian National AIDS Council. Zambia Sexual Behavior Survey 2005. Lusaka, Zambia: Central Statistical Office (Zambia).	
Zambia	2007	Zambia Demographic and Health Survey 2007	Survey microdata	GPS	X	X	X	X	X	X	X	X	Central Statistical Office (Zambia), Macro International, Inc. Zambia Demographic and Health Survey 2007. Fairfax, United States: ICF International.	21117
Zambia	2008	Zambia Global Fund Household Health Coverage Survey 2008	Survey microdata	Admin 3			X	X					Central Statistical Office (Zambia). Zambia Global Fund Household Health Coverage Survey 2008. Lusaka, Zambia: Central Statistical Office (Zambia).	26702
Zambia	2009	Zambia Sexual	Survey microdata	Admin 2	X	X	X	X		X	X	X	Central Statistical Office (Zambia), MEASURE Evaluation Project, Carolina	59339

Country	Years	Name	Type	Geographic Detail	Male Circumcision	STI Symptoms	Had Intercourse (Women)	Partner Away	Condom Last Time	Multiple Partners in Year (Men)	Multiple Partners in Year (Women)	In Union	Citation	NID*
		Behavior Survey 2009											Population Center, University of North Carolina, Ministry of Health (Zambia), University of Zambia, Zambian National AIDS Council. Zambia Sexual Behavior Survey 2009.	
Zambia	2010	Zambia Living Conditions Monitoring Survey 2010	Survey microdata	Admin 2								X	Central Statistical Office (Zambia). Zambia Living Conditions Monitoring Survey 2010.	58660†
Zambia	2013-14	Zambia Demographic and Health Survey 2013-2014	Survey microdata	GPS	X	X	X	X	X	X	X	X	Central Statistical Office (Zambia), ICF International, Ministry of Health (Zambia), Tropical Diseases Research Centre, University Teaching Hospital (Zambia), University of Zambia. Zambia Demographic and Health Survey 2013-2014. Fairfax, United States: ICF International.	77516
Zimbabwe	2005	Zimbabwe-Chimanimani Behavioral Risks and HIV	Survey report	Admin 2	X								Biomedical Research and Training Institute (Zimbabwe), Human Sciences Research Council, Zimbabwe Central Statistics Office. Zimbabwe - Chimanimani Behavioral Risks and HIV Serostatus Survey 2005.	333662

Country	Years	Name	Type	Geographic Detail	Male Circumcision	STI Symptoms	Had Intercourse (Women)	Partner Away	Condom Last Time	Multiple Partners in Year (Men)	Multiple Partners in Year (Women)	In Union	Citation	NID*
		Serostatus Survey 2005												
Zimbabwe	2005-06	Zimbabwe Demographic and Health Survey 2005-2006	Survey microdata	GPS	X	X	X	X	X	X	X	X	Central Statistical Office (Zimbabwe), Macro International, Inc. Zimbabwe Demographic and Health Survey 2005-2006. Calverton, United States: Macro International, Inc.	21163
Zimbabwe	2009	Zimbabwe Multiple Indicator Monitoring Survey 2009	Survey microdata	Admin 1								X	Central Statistical Office (Zimbabwe). Zimbabwe Multiple Indicator Monitoring Survey 2009. New York, United States: United Nations Children's Fund (UNICEF).	35493
Zimbabwe	2010-11	Zimbabwe Demographic and Health Survey 2010-2011	Survey microdata	GPS	X	X	X	X	X	X	X	X	ICF Macro, Zimbabwe National Statistics Agency. Zimbabwe Demographic and Health Survey 2010-2011. Calverton, United States: ICF Macro, 2012.	55992
Zimbabwe	2014	Zimbabwe Multiple Indicator Cluster	Survey microdata	Admin 1	X		X		X	X	X	X	United Nations Children's Fund (UNICEF), Zimbabwe National Statistics Agency. Zimbabwe Multiple Indicator Cluster Survey 2014. New	152720

Country	Years	Name	Type	Geographic Detail	Male Circumcision	STI Symptoms	Had Intercourse (Women)	Partner Away	Condom Last Time	Multiple Partners in Year (Men)	Multiple Partners in Year (Women)	In Union	Citation	NID*
		Survey 2014											York, United States: United Nations Children's Fund (UNICEF), 2015.	
Zimbabwe	2015	Zimbabwe Demographic and Health Survey 2015	Survey microdata	GPS	X	X	X	X	X	X	X	X	ICF International, National Microbiology Reference Laboratory, Harare Central Hospital (NMRL) (Zimbabwe), Zimbabwe National Statistics Agency. Zimbabwe Demographic and Health Survey 2015. Fairfax, United States: ICF International, 2016.	157066
Zimbabwe	2016	Zimbabwe Population-based HIV Impact Assessment 2015-2016	Survey report	Admin 1	X								Ministry of Health and Child Care (MOHCC), Zimbabwe, Centers for Disease Control and Prevention (CDC), and ICAP at Columbia University. Zimbabwe Population-based HIV Impact Assessment (ZIMPHIA) 2015-16: First Report. Harare, Zimbabwe, Atlanta, Georgia and New York, New York, USA: Ministry of Health, CDC and ICAP. December 2016.	287631

*NID = Data source unique identifier in the Global Health Data Exchange (http://ghdx.healthdata.org/). Additional information about each data sources is available via the GHDx, including information about the data provider and links to where the data can be accessed or requested (where available). NIDs can be entered in the search bar to retrieve the record for a particular source.

†Data source is not publicly available due to restrictions by the data provider and was used under license for the current study.

Supplementary Table 7: Surveys excluded from this analysis

Country	Year(s)	Name	Citation	Excluded from	Rationale
All countries (Burkina Faso, Chad, Comoros, Congo, Côte d'Ivoire, Ethiopia, Ghana, Kenya, Malawi, Mali, Mauritania, Namibia, Senegal, South Africa, Swaziland, Zambia, Zimbabwe)	All years (2002-04)	World Health Surveys (WHS)	World Health Organization (WHO). Geneva, Switzerland: World Health Organization (WHO), 2005.	All	Geographic patterns of cluster locations in available microdata are inconsistent with reported maps. Additionally, estimates were considered outliers compared to other surveys in the same countries.
Benin	2009	Benin Household Living Conditions Survey	Benin Household Living Conditions Survey 2009.	'in union'	Estimates for 'in union' were implausibly high compared to estimates from other surveys (2006 and 2011 DHS).
Botswana	2001 and 2004	Botswana AIDS Impact Surveys	Central Statistics Office (Botswana), Ministry of Health (Botswana). Botswana AIDS Impact Survey 2001. Gaborone, Botswana: Statistics Botswana.<br><br>Central Statistics Office (Botswana). Botswana AIDS Impact Survey 2004. Gaborone, Botswana: Statistics Botswana.	'partner away'	These surveys did not ask the 'partner away' question for respondents who had previously indicated they were living with a partner and survey estimates were implausibly low compared to estimates from the same survey series in later years (2008 and 2013).
Burkina Faso	2005	Burkina Faso CWIQ Survey	National Institute of Statistics and Demography (Burkina Faso), World Bank. Burkina Faso Core Welfare Indicators Questionnaire Survey 2005. Ouagadougou, Burkina Faso: National Institute of Statistics and Demography (Burkina Faso)	'in union'	Implausibly low estimates as compared to DHS 2005

Country	Year(s)	Name	Citation	Excluded from	Rationale
Burundi	2005	Multiple Indicator Cluster Survey (MICS)	United Nations Children's Fund (UNICEF), Burundi Institute of Statistics and Economic Studies, United Nations Population Fund (UNFPA). Burundi Multiple Indicator Cluster Survey 2005. New York, United States: United Nations Children's Fund (UNICEF)	'in union'	Implausibly low estimates as compared to other surveys
Cameroon	2006	Multiple Indicator Cluster Survey (MICS)	United Nations Children's Fund (UNICEF), National Institute of Statistics (Cameroon). Cameroon Multiple Indicator Cluster Survey 2006. New York, United States: United Nations Children's Fund (UNICEF).	'had intercourse'	Estimates for 'had intercourse' were implausibly lower than estimates from other surveys (2004 DHS and 2011 DHS) and, in comparison with published reports for this survey, it appears missing responses were marked as false values for this indicator in reports with no explanation.
Central African Republic	2000	Multiple Indicator Cluster Survey (MICS)	Division of Statistics and Economic Studies (Central African Republic), Ministry of Economy, Planning and International Cooperation (Central African Republic), United Nations Children's Fund (UNICEF). Central African Republic Multiple Indicator Cluster Survey 2000. New York, United States: United Nations Children's Fund (UNICEF)	'in union'	Implausibly low estimates as compared to other surveys in later years.
Democratic Republic of the Congo	2013-14, 2014, 2015,	Performance, Monitoring, and	Performance Monitoring and Accountability 2020 (PMA2020) Project,	'in union'	Estimates for 'in union' were implausibly high compared to estimates from other surveys

Country	Year(s)	Name	Citation	Excluded from	Rationale
	2015-16, and 2016	Accountability Survey (PMA)	University of Kinshasa School of Public Health, Tulane University School of Public Health and Tropical Medicine. 2013, 2014, 2015, 2016. Democratic Republic of the Congo. Baltimore, MD: PMA2020, Bill & Melinda Gates Institute for Population and Reproductive Health, Johns Hopkins Bloomberg School of Public Health.		(2001 and 2010 MICS, 2007 and 2013-2014 DHS).
Ghana	2007-08	Multiple Indicator Cluster Survey (MICS)	Ghana Statistical Service, Ministry of Health (Ghana), United Nations Children's Fund (UNICEF). Ghana District Multiple Indicator Cluster Survey 2007-2008.	'had intercourse'	Survey data had zero explicit 'never had intercourse' responses, so survey estimates are implausibly low compared to estimates from other surveys (2003, 2008, and 2014 DHS and 2006 MICS).
Kenya	2005-2006	Kenya Integrated HH Budget Survey	Kenya National Bureau of Statistics. Kenya Integrated Household Budget Survey Tabular Data 2005-2006. Nairobi, Kenya: Kenya National Bureau of Statistics.	'in union'	Implausibly low estimates as compared to other surveys.
Kenya	2015	STEPS Noncommunicable Disease Risk Factors Survey	Kenya National Bureau of Statistics, Ministry of Health (Kenya), World Health Organization (WHO). Kenya STEPS Noncommunicable Disease Risk Factors Survey 2015.	'in union'	Estimates for 'in union' were implausibly high compared to estimates from other surveys (2008 DHS and 2014 and 2015 PMA).
Malawi	2013	Malawi Integrated HH Survey	National Statistical Office of Malawi. Malawi Integrated Household Survey 2013 (IHS3 - Year: 2). Dataset downloaded from	'in union'	Implausibly low estimates as compared to other surveys in neighbouring years.

Country	Year(s)	Name	Citation	Excluded from	Rationale
			[http://go.worldbank.org/NOXNI9YDS0] on [September 15, 2015]		
Mali	2014-15	Living Standards Measurement Study – Integrated Survey on Agriculture (LSMS – ISA)	Ministry of Rural Development (Mali), National Institute of Statistics (INSTAT) (Mali), World Bank. Mali Agricultural Integrated Economic Survey 2014-2015. Washington DC, United States: World Bank.	'in union'	Estimates for 'in union' were implausibly low compared to other surveys (2012 DHS, 2015 MICS, and 2015 MIS).
Namibia	2009-2010	Namibia Household Income and Expenditure Survey	Namibia Statistics Agency. Namibia Household Income and Expenditure Survey 2009-2010. Windhoek, Namibia: Namibia Statistics Agency.	'in union'	Estimates for 'in union' were implausibly low compared to other surveys (2006 and 2013 DHS).
Nigeria	2007 and 2012	National HIV/AIDS and Reproductive Health Survey	Federal Ministry of Health (Nigeria), National Bureau of Statistics (Nigeria), National Population Commission (NPC), Society for Family Health (Nigeria), University College Hospital, Ibadan. Nigeria National HIV/AIDS and Reproductive Health Surveys 2007 and 2012.	'male circumcision'	Estimates for 'male circumcision' were implausibly lower than estimates from other survey series (2008 DHS, 2013 DHS, and 2016-2017 MICS).
Nigeria	2008-10	Living Standards Survey	National Bureau of Statistics (Nigeria). Nigeria Living Standards Survey 2008-2010. Abuja, Nigeria: National Bureau of Statistics (Nigeria).	'male circumcision'	Estimates for 'male circumcision' were implausibly lower than estimates from other survey series (2008 DHS, 2013 DHS, and 2016-2017 MICS).
Nigeria	2012-2013	Nigeria General HH Survey	National Bureau of Statistics (Nigeria). Nigeria General Household Survey 2012-2013. Washington DC,	'in union'	Implausibly low estimates as compared to other surveys in neighbouring years.

Country	Year(s)	Name	Citation	Excluded from	Rationale
			United States: World Bank		
South Africa	All years	International Social Science Survey Programme (ISSP)	ISSP Research Group (2009): International Social Survey Programme: Leisure Time and Sports - ISSP 2007. GESIS Data Archive, Cologne. ZA4850 Data file version 2.0.0, doi:10.4231/1.10079.<br><br>ISSP Research Group (2008): International Social Survey Programme: Role of Government IV - ISSP 2006. GESIS Data Archive, Cologne. ZA4700 Data file Version 1.0.0, doi:10.4232/1.4700.<br><br>ISSP Research Group (2012): International Social Survey Programme: Religion III - ISSP 2008. GESIS Data Archive, Cologne. ZA4950 Data file Version 2.2.0, doi:10.4232/1.11334.<br><br>ISSP Research Group (2017): International Social Survey Programme: Social Inequality IV - ISSP 2009. GESIS Data Archive, Cologne. ZA5400 Data file Version 4.0.0, doi:10.4232/1.12777.<br><br>ISSP Research Group (2012): International Social Survey Programme: Environment III - ISSP 2010. GESIS Data	'in union'	Estimates for 'in union' were consistently low for this survey series compared to all other surveys in country.

Country	Year(s)	Name	Citation	Excluded from	Rationale
			Archive, Cologne. ZA5500 Data file Version 2.0.0, doi:10.4232/1.11418.<br><br>ISSP Research Group (2016): International Social Survey Programme: Family and Changing Gender Roles IV - ISSP 2012. GESIS Data Archive, Cologne. ZA5900 Data file Version 4.0.0, doi:10.4232/1.12661.<br><br>ISSP Research Group (2012): International Social Survey Programme: Citizenship - ISSP 2004. GESIS Data Archive, Cologne. ZA3950 Data file Version 1.3.0, doi:10.4232/1.11372.<br><br>ISSP Research Group (2013): International Social Survey Programme: Work Orientation III - ISSP 2005. GESIS Data Archive, Cologne. ZA4350 Data file Version 2.0.0, doi:10.4232/1.11648.<br><br>ISSP Research Group (2015): International Social Survey Programme: National Identity III - ISSP 2013. GESIS Data Archive, Cologne. ZA5950 Data file Version 2.0.0, doi:10.4232/1.12312.<br><br>ISSP Research Group (2016): International Social Survey		

Country	Year(s)	Name	Citation	Excluded from	Rationale
			Programme: Citizenship II - ISSP 2014. GESIS Data Archive, Cologne. ZA6670 Data file Version 2.0.0, doi:10.4232/1.12590.		
Uganda	2009-10	Uganda Living Standards Measurement Survey – Integrated Survey on Agriculture (LSMS – ISA)	Uganda Bureau of Statistics. Uganda Living Standards Measurement Survey - Integrated Survey on Agriculture 2009-2010. Washington DC, United States: World Bank.	‘in union’	Estimates for ‘in union’ were implausibly low compared to other surveys (2006 and 2011 DHS, 2011 AIS).

Supplementary Table 8: Model prior sensitivity analyses

Indicator	Model results compared	Correlation			Mean absolute error (PPT)			90th percentile absolute difference (PPT)			Percent of overlapping uncertainty intervals		
		Grid cell	Admin 1	Admin 2	Grid cell	Admin 1	Admin 2	Grid cell	Admin 1	Admin 2	Grid cell	Admin 1	Admin 2
HIV Prevalence	Model 1	0.998	0.999	0.999	0.171	0.148	0.126	0.41	0.363	0.32	100	100	100
	Model 2	0.998	0.999	0.999	0.17	0.146	0.119	0.397	0.336	0.284	100	100	100
	Model 3	0.998	0.999	0.999	0.174	0.157	0.133	0.417	0.364	0.324	100	100	100
	Model 4	0.999	0.999	0.999	0.161	0.147	0.119	0.386	0.349	0.299	100	100	100
	Model 5	0.998	0.999	0.999	0.175	0.156	0.123	0.428	0.367	0.307	100	100	100
Prevalence of Male Circumcision (male circumcision)	Model 1	0.999	0.999	0.999	0.7	0.662	0.647	1.878	1.866	1.909	100	100	100
	Model 2	0.998	0.998	0.999	1.062	0.811	0.897	3.381	2.357	2.869	100	100	100
	Model 3	0.999	0.999	0.999	0.821	0.702	0.72	2.349	2.012	2.227	100	100	100
	Model 4	0.999	0.999	0.999	0.767	0.631	0.678	2.11	1.794	1.835	100	99.99	100
	Model 5	0.999	0.999	1	0.56	0.536	0.474	1.47	1.401	1.205	100	100	100
Prevalence of self-reported STI symptoms (STI symptoms)	Model 1	0.99	0.994	0.995	0.503	0.44	0.489	1.082	1.026	1.144	100	100	100
	Model 2	0.994	0.996	0.996	0.399	0.334	0.401	0.898	0.8	0.954	100	100	100
	Model 3	0.995	0.997	0.998	0.346	0.303	0.307	0.809	0.727	0.753	100	100	100
	Model 4	0.995	0.997	0.998	0.328	0.315	0.307	0.765	0.766	0.754	100	100	100
	Model 5	0.997	0.998	0.999	0.288	0.256	0.254	0.639	0.593	0.597	100	100	100
Prevalence of marriage or living with a partner as married (in union)	Model 1	0.998	0.999	0.999	0.497	0.418	0.327	1.081	0.928	0.754	100	100	100
	Model 2	0.997	0.998	0.998	0.693	0.538	0.459	1.562	1.219	1.075	100	100	100
	Model 3	0.999	0.999	0.999	0.37	0.336	0.244	0.79	0.75	0.554	100	100	100
	Model 4	0.999	0.999	0.999	0.357	0.322	0.226	0.757	0.716	0.504	100	100	100
	Model 5	0.999	0.999	1	0.353	0.32	0.215	0.75	0.717	0.491	100	100	100
Prevalence of one's current partner living elsewhere (partner away)	Model 1	*	*	*	*	*	*	*	*	*	*	*	*
	Model 2	0.99	0.997	0.997	0.79	0.56	0.452	1.995	1.313	1.021	100	100	100
	Model 3	0.998	0.999	0.999	0.423	0.36	0.251	0.945	0.824	0.601	100	100	100
	Model 4	0.998	0.999	0.999	0.418	0.381	0.269	0.929	0.89	0.618	100	100	100
	Model 5	0.998	0.999	0.999	0.385	0.363	0.262	0.853	0.839	0.639	100	100	100

Prevalence of condom use at last sexual encounter with the last 12 months (condom use)	Model 1	*	*	*	*	*	*	*	*	*	*	*	*
	Model 2	*	*	*	*	*	*	*	*	*	*	*	*
	Model 3	0.998	0.998	0.999	0.482	0.44	0.35	1.251	1.139	0.961	100	100	100
	Model 4	0.998	0.998	0.998	0.569	0.464	0.402	1.573	1.196	1.019	100	100	100
	Model 5	0.999	0.999	0.999	0.376	0.381	0.279	0.936	0.97	0.695	100	100	100
Prevalence of reporting ever had sexual intercourse among young adults (had intercourse)	Model 1	*	*	*	*	*	*	*	*	*	*	*	*
	Model 2	*	*	*	*	*	*	*	*	*	*	*	*
	Model 3	0.997	0.998	0.998	0.626	0.536	0.442	1.464	1.194	0.914	100	100	100
	Model 4	0.997	0.999	0.999	0.601	0.484	0.349	1.43	1.086	0.822	100	100	100
	Model 5	0.999	0.999	0.999	0.375	0.393	0.262	0.805	0.878	0.575	100	100	100
Prevalence of men reporting multiple sexual partners within the last year (Multiple partners among men)	Model 1	0.997	0.997	0.997	0.408	0.411	0.355	0.888	0.94	0.854	100	100	100
	Model 2	0.995	0.997	0.997	0.487	0.372	0.341	1.083	0.807	0.749	100	100	100
	Model 3	0.998	0.998	0.998	0.365	0.336	0.303	0.813	0.755	0.695	100	100	100
	Model 4	0.998	0.998	0.998	0.405	0.352	0.311	0.91	0.787	0.715	100	100	100
	Model 5	0.998	0.998	0.998	0.375	0.354	0.301	0.818	0.814	0.705	100	100	100
Prevalence of women reporting multiple sexual partners within the last year (Multiple partners among women)	Model 1	0.99	0.989	0.988	0.204	0.188	0.191	0.528	0.518	0.535	100	100	100
	Model 2	0.992	0.995	0.993	0.179	0.136	0.149	0.463	0.358	0.405	100	100	100
	Model 3	0.997	0.998	0.998	0.113	0.089	0.09	0.285	0.229	0.244	100	100	100
	Model 4	0.996	0.997	0.997	0.136	0.108	0.105	0.329	0.281	0.28	100	100	100
	Model 5	0.997	0.998	0.998	0.105	0.082	0.075	0.258	0.204	0.187	100	100	100

*Indicates model failed to converge

Supplementary Table 8 presents metrics of model comparison for the sensitivity analyses on hyper-prior selection. For each indicator, it presents a comparison between all given models described in section 4.2 with the selected model, computing the correlation, mean absolute error, 90th percentile of observed difference and percent of uncertainty interval overlap at the raster, first administrative level, and second administrative level.

Supplementary Table 9: Fitted model parameters

	Central sub-Saharan Africa			Eastern sub-Saharan Africa			Southern sub-Saharan Africa			Western sub-Saharan Africa		
**Parameter**	**0.025th quantile**	**0.500th quantile**	**0.975th quantile**	**0.025th quantile**	**0.500th quantile**	**0.975th quantile**	**0.025th quantile**	**0.500th quantile**	**0.975th quantile**	**0.025th quantile**	**0.500th quantile**	**0.975th quantile**
β_0	-0.7226	-0.3067	0.1089	-1.2724	-0.6943	-0.1167	-2.2557	-1.904	-1.5527	-1.7297	-1.1536	-0.5785
β_1 (GAM)	0.0346	0.1423	0.25	0.1093	0.1775	0.2457	-0.0696	0.0012	0.0719	0.1832	0.3066	0.4301
β_1 (BRT)	0.5211	0.6166	0.712	0.5867	0.6368	0.6867	0.0759	0.1094	0.143	0.299	0.3757	0.4522
β_1 (Lasso)	0.0166	0.1585	0.3004	0.1167	0.2272	0.3377	-0.2843	-0.126	0.0324	-0.0642	0.1224	0.3087
Standard Dev. for $\gamma_{c[i]}$	0.0964	0.1698	0.287	0.9595	1.2255	1.5408	0.2143	0.362	0.6453	0.2267	0.301	0.3911
Range for $Z_{i,t}$	4.8846	6.7006	9.0044	4.4059	5.5519	7.4679	3.6984	4.1858	4.9569	4.2435	6.0883	8.1669
Standard Dev. for $Z_{i,t}$	0.3323	0.446	0.559	0.5127	0.6183	0.7428	0.3751	0.4146	0.4686	0.4056	0.5192	0.6329
ρ for $Z_{i,t}$	0.8689	0.9404	0.9659	0.9572	0.9732	0.9831	0.9686	0.9743	0.9799	0.9271	0.9573	0.9725
Standard Dev. for $\epsilon_{i,t}$	0.2428	0.2626	0.2863	0.3026	0.315	0.333	0.0007	0.0038	0.0107	0.3083	0.3288	0.3463
β_2	0.139	0.2193	0.2997	0.089	0.1478	0.2066	0.2389	0.4019	0.5648	0.2545	0.3141	0.3737
Range for U_i	0.1585	0.3022	0.5099	0.2022	0.3642	0.5366	3.6465	5.3005	8.5695	0.0851	0.1503	0.2385
Standard Dev. for U_i	0.4306	0.5642	0.7816	0.4729	0.5505	0.6883	0.229	0.3025	0.427	0.5842	0.8399	1.3074
